# Model-Based Simulation of Maintenance Therapy of Childhood Acute Lymphoblastic Leukemia

**DOI:** 10.3389/fphys.2020.00217

**Published:** 2020-03-18

**Authors:** Felix Jost, Jakob Zierk, Thuy T. T. Le, Thomas Raupach, Manfred Rauh, Meinolf Suttorp, Martin Stanulla, Markus Metzler, Sebastian Sager

**Affiliations:** ^1^Department of Mathematics, Institute of Mathematical Optimization, Otto-von-Guericke University Magdeburg, Magdeburg, Germany; ^2^Department of Paediatrics and Adolescent Medicine, University Hospital Erlangen, Erlangen, Germany; ^3^Pediatric Hematology and Oncology, University Hospital “Carl Gustav Carus”, Dresden, Germany; ^4^Department of Pediatric Hemato-Oncology, Hannover Medical School, Hanover, Germany; ^5^Health Campus “Immunology, Infectiology and Inflammation (GC-I^3^)”, Otto-von-Guericke University, Magdeburg, Germany

**Keywords:** childhood acute lymphoblastic leukemia, maintenance therapy, 6-mercaptopurine, methotrexate, neutropenia, non-linear mixed-effects modeling, population pharmacokinetics/pharmacodynamics

## Abstract

Acute lymphoblastic leukemia is the most common malignancy in childhood. Successful treatment requires initial high-intensity chemotherapy, followed by low-intensity oral maintenance therapy with oral 6-mercaptopurine (6MP) and methotrexate (MTX) until 2–3 years after disease onset. However, intra- and inter-individual variability in the pharmacokinetics (PK) and pharmacodynamics (PD) of 6MP and MTX make it challenging to balance the desired antileukemic effects with undesired excessive myelosuppression during maintenance therapy. A model to simulate the dynamics of different cell types, especially neutrophils, would be a valuable contribution to improving treatment protocols (6MP and MTX dosing regimens) and a further step to understanding the heterogeneity in treatment efficacy and toxicity. We applied and modified a recently developed semi-mechanistic PK/PD model to neutrophils and analyzed their behavior using a non-linear mixed-effects modeling approach and clinical data obtained from 116 patients. The PK model of 6MP influenced the accuracy of absolute neutrophil count (ANC) predictions, whereas the PD effect of MTX did not. Predictions based on ANC were more accurate than those based on white blood cell counts. Using the new cross-validated mathematical model, simulations of different treatment protocols showed a linear dose-effect relationship and reduced ANC variability for constant dosages. Advanced modeling allows the identification of optimized control criteria and the weighting of specific influencing factors for protocol design and individually adapted therapy to exploit the optimal effect of maintenance therapy on survival.

## 1. Introduction

Acute lymphoblastic leukemia (ALL), characterized by malignant white blood cells (WBCs) and displacement of normal hematopoiesis, is the most common childhood malignancy (Hoffbrand et al., 2016). The treatment of childhood ALL is based on combination chemotherapy and begins with intensive, high-dose treatment for approximately 6 months (the so-called induction and consolidation therapy) followed by less-intensive, low-dose treatment [so-called maintenance therapy (MT)] that lasts for 2–3 years after disease onset. The goal of induction and consolidation therapy is to achieve remission via lymphoblast elimination below the limit of detection, and the high intensity of these therapy elements limits further therapy intensification using conventional chemotherapy. Subsequent MT is essential to prevent disease relapse, and aims to maintain prolonged antileukemic activity against residual lymphoblasts, with minimal adverse events. MT includes daily oral 6-mercaptopurine (6MP) and weekly oral methotrexate (MTX). Both drugs cause myelosuppression through their metabolized active forms (Schmiegelow et al., 2014). Blood count tests are performed regularly to ensure adequate WBC and absolute neutrophil count (ANC) suppression as a surrogate marker for antileukemic activity, without unintended excessive myelotoxicity. However, there exists no international consensus for MT dosing strategies and target levels for WBC and ANC suppression (i.e., what dose to start with, and when and how to increase or decrease chemotherapy). Empirical evaluation of different MT strategies using randomized clinical trials would be extremely challenging, due to the probably moderate effect size, the length of MT, the latency of clinically relevant endpoints, and the risk of compromising the current overall favorable outcome of childhood ALL. However, certain levels of WBC and ANC are established factors for survival, relapse or death, and other adverse events (e.g., infection), respectively. Therefore, a simulation model of childhood ALL MT could support the development of future MT strategies by identifying those strategies that achieve established survival factors best while avoiding established risk factors. Mathematical models describing the pharmacokinetics (PK) of 6MP and MTX and their pharmacodynamic (PD) effects on neutrophils may help clarify the drug-exposure relationship, predict the ANC dynamics, adapt subsequent dosing amounts, and stratify patients into groups with different drug responses. Several PK models for 6MP (Hawwa et al., 2008; Jayachandran et al., 2014, 2015) and MTX (Godfrey et al., 1998; Panetta et al., 2002, 2010; Nagulu et al., 2010; Rühs et al., 2012; Korell et al., 2013; Hui et al., 2019) have been published, but not all have been developed with low-dosage treatments and validated in the pediatric population. To the best of our knowledge, there are only three publications (Jayachandran et al., 2014; Le et al., 2018; Karppinen et al., 2019) in which some of the PK models or their simplifications were linked to transient PD compartment models (Upton and Mould, 2014). The models were individually fitted to WBC counts and different prediction and optimization studies were conducted.

Here, we developed a population PK/PD model for maintenance treatment of ALL in children based on the approach used by Le et al. (2018) with a modified underlying PK model. As ANCs are the best established risk and survival factors, we adapted the model to predict ANCs instead of WBCs. The model was fitted to and validated on a dataset consisting of weekly ANC measurements obtained from 116 patients treated with daily oral 6MP and weekly oral MTX over an average of 459 (range, 200–581) days. We started our investigations with a PK/PD model considering 6MP and MTX but the constant administration ratio hampered the identification of separate PD effects. Further, the PK of MTX had no significant impact on the improvement of the model fitting, similar to the mathematical approach in Karppinen et al. (2019) and the clinical findings of *NUDT15* genetics conferring 6MP but no MTX sensitivities (Tsujimoto et al., 2018). Thus, the final model only contains the PK of 6MP. We come back to this issue in the discussion. Then, for each patient, we simulated different therapy protocols (6MP dosing regimens), and compared the resulting predictions.

## 2. Patients and Methods

### 2.1. Data

The data used in this study were obtained retrospectively from 116 children who were diagnosed with de novo ALL at university hospitals in Erlangen and Dresden and treated according to the AIEOP-BFM 2000 and 2009 protocols. A subset of this data set (WBC counts from nine patients) was used and described similarly in a previous study (Le et al., 2018). Patients were eligible if they were diagnosed with precursor B-cell or T-cell ALL, negative for the BCR-ABL- and MLL-AF4 translocations, and started MT (i.e., did not experience relapse before the end of consolidation therapy and did not undergo stem cell transplantation). During MT administered according to the AIEOP-BFM 2000 and 2009 protocols, patients received oral chemotherapy with daily 6MP and once-weekly MTX until 2 years after ALL diagnosis. During MT, chemotherapy was applied to achieve antileukemic activity against lymphoblasts below the limit of detection. As a surrogate for antileukemic activity, WBC and ANC were measured regularly, with ANC <2 G/L, being correlated to a significantly better relapse-free survival (Schmiegelow et al., 2014), and ANC <0.5 G/L being an indicator of excessive myelosuppression. The target range for the WBC count was 1.5–3 G/L. The chemotherapeutic dose was reduced when cell counts fell below the lower limits (WBC count <1.5 G/L, ANC <0.5 G/L, lymphocyte count <0.3 G/L, and platelet count <0.05 G/L) or liver toxicity was suspected. For each patient included in the analysis, data regarding the following variables were recorded: gender, age, weight, height, body surface area (BSA), prescribed 6MP and MTX dosages (absolute and per BSA), WBC count, platelet count, lymphocyte and neutrophil counts, and therapy interruptions. In this study, we focused on 5897 ANCs and 6640 WBC counts, disregarding measurements of other cell types. We used both WBC counts and ANC separately and compared the accuracy of the resulting mathematical models. In all, 1150 ANC and 1289 WBC count measurements were excluded due to concurrent high C-reactive protein (CRP) levels indicating periods in which patients probably suffered from an infection. More precisely, we excluded measurements in the interval from 2 weeks before until 2 weeks after CRP levels of >5 mg/Lwere recorded. Among the remaining 4747 ANC measurements 56% were below the ANC threshold of 2 G/L, only 2% were below 0.5 G/L, and 54% were in the ANC target range 0.5–2 G/L. The demographic and clinical characteristics of the pediatric ALL population are shown in Table 1.

**Table 1:** Characteristics (median and range) of the pediatric ALL population consisting of 116 (64 male and 52 female) patients.

**Characteristic**	**Unit**	**Median**	**Range**
Age	Year	4.75	1.1–17.1
Weight	kg	22	10–90
Height	cm	112.45	80–182.7
Body surface area	m^2^	0.82	0.47–1.98
6MP daily dose	mg	40	5–150
MTX weekly dose	mg	15	1.25–60
ANC	G/L	1.8	0.0–19.9

*The body surface area was calculated using the Mosteller formula*.

### 2.2. Non-linear Mixed-Effects Modeling and Parameter Estimation

The non-linear mixed-effects (NLME) modeling (Bonate and Steimer, 2011) was based on the PK/PD model of Le et al. (2018). It describes the absorption of both drugs, 6MP and MTX, through the gastrointestinal (GI) tract into the plasma after oral administration and their metabolization to their active forms. The MTX metabolites MTXPG_2_ to MTXGP_7_ inhibit several enzymes responsible for DNA synthesis (Panetta et al., 2002). The active form of 6MP, 6-thioguanine nucleotides (6-TGNs), is incorporated into the DNA (Hawwa et al., 2008). Thus, both drugs negatively affect the hematopoiesis of neutrophils. During the model development, we replaced the 6MP PK model of Jayachandran et al. (2014) with the PK model described by Hawwa et al. (2008) to obtain a better response to 6MP dosage. The PK model of Jayachandran et al. (2014) was validated on concentration data of eight patients (adults) from Hindorf et al. (2006). However, the simulated 6-TGN concentrations coincided with data from pediatric patients reported by Hawwa et al. (2008); hence, it was a priori unclear which would give better results. Both compartment models have a comparable representation of the absorption and metabolic pathway of 6MP but the model of Hawwa et al. (2008) describes the metabolic transformations by first order kinetics instead of Michaelis–Menten kinetics. Further, the clearance is described by a BSA-dependent term, thus providing individualized PK profiles through patient characteristics. We also tested the influence of weekly MTX administration by either ignoring or considering the administrations and their resulting concentrations through the MTX PK model with a second PD parameter during model fitting. Additionally, we tested the myelosuppression model from Jayachandran et al. (2014), which contained a different feedback term for ANC recovery, but the accuracy decreased and this line of research was not further investigated.

**Figure 1:**
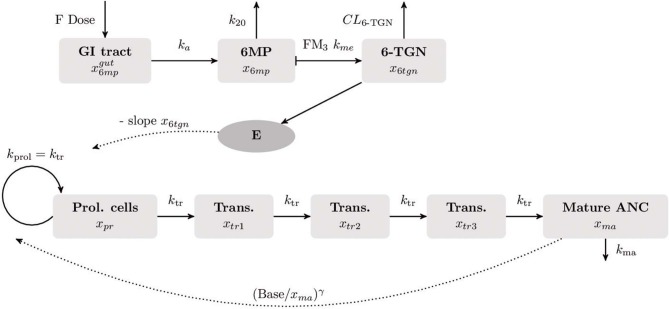
Visualization of the final compartment model used for the population PK/PD analysis. The underlying mathematical models for the PK of 6MP and the myelosuppression were published by Hawwa et al. (2008), respectively (Le et al., 2018). The PK of orally administered 6MP is described as a three compartment model. A fraction of the 6MP dosage (6MP dose multiplied with the bioavailability factor F) enters the gastrointestinal (GI) tract where bioavailable 6MP is absorbed into the central compartment by the rate *k*_*a*_. In the central compartment, 6MP is eliminated with the first order kinetics *k*_20_. The elimination rate also comprises metabolization of 6MP to its active form 6-thioguanine nucleotide (6-TGN) with the rate FM_3_ *k*_*m*_. The hematopoiesis of neutrophils is described by a chain of five compartments. The first compartment represents the hematopoietic stem cells proliferating with the rate *k*_*prol*_. The maturation process with equivalent transition rates *k*_*tr*_ is represented by three intermediate compartments after which matured cells enter the circulating blood (last compartment). Matured cells die by the process of apoptosis with the rate *k*_*ma*_. The neutrophil baseline *Base* is maintained by the feedback term ${\left(B/x_{ma}\right)}^{\gamma }$. As 6-TGN is incorporated into the DNA leading to cell apoptosis, the proliferation process is negatively affected by a linear PD function E.

As a result, we identified one PK/PD model which described the clinical data best. This model was formulated as a system of ordinary differential equations (ODEs):

(1) $$\begin{array}{l} {\dot{x}}_{6mp}^{gut}\left(t\right)=-k_{a}\;x_{6mp}^{gut}\left(t\right)+F\;u\left(t\right),\\ {\dot{x}}_{6mp}\left(t\right)=k_{a}\;x_{6mp}^{gut}\left(t\right)-k_{20}\;x_{6mp}\left(t\right),\\ {\dot{x}}_{6tgn}\left(t\right)={\text{FM}}_{3}\;k_{me}\;x_{6mp}\left(t\right)-{\text{CL}}_{6tgn}\left(\text{BSA}\right)\;x_{6tgn}\left(t\right)\\ {\dot{x}}_{pr}\left(t\right)=k_{tr}\;x_{pr}\left(t\right)\;\left(1-E_{drug}\right)\;{\left(\frac{Base}{x_{ma}\left(t\right)}\right)}^{\gamma }-k_{tr}\;x_{pr}\left(t\right),\\ {\dot{x}}_{tr1}\left(t\right)=k_{tr}\;\left(x_{pr}\left(t\right)-x_{tr1}\left(t\right)\right),\\ {\dot{x}}_{tr2}\left(t\right)=k_{tr}\;\left(x_{tr1}\left(t\right)-x_{tr2}\left(t\right)\right),\\ {\dot{x}}_{tr3}\left(t\right)=k_{tr}\;\left(x_{tr2}\left(t\right)-x_{tr3}\left(t\right)\right),\\ {\dot{x}}_{ma}\left(t\right)=k_{tr}\;x_{tr3}\left(t\right)-k_{ma}\;x_{ma}\left(t\right) \end{array}$$

with the BSA-dependent clearance

(2) $$\begin{array}{ll} {\text{CL}}_{6tgn}\left(\text{BSA}\right) & =0.00914\;{\left(\text{BSA}\right)}^{1.16}, \end{array}$$

the linear pharmacodynamic effect

(3) $$\begin{array}{ll} E_{drug} & =\text{slope }x_{6tgn}, \end{array}$$

and the patient-specific bioavailable 6MP amount *F u*(*t*) of 6MP (implemented as point administration in NONMEM). The PK of 6MP is described by a three compartment model altered from Hawwa et al. (2008). A fraction of the orally administered 6MP dosage enters the GI tract where bioavailable 6MP is absorbed to the central compartment with the first order rate *k*_*a*_. In the central compartment, 6MP is eliminated by *k*_20_. The elimination also comprises metabolization of 6MP with the rate *k*_*me*_ out of which a fraction FM_3_ is metabolized to the active form 6-TGN. 6-TGN is then cleared by the BSA-dependent clearance term CL_6*tgn*_. The hematopoiesis of neutrophils is described via a chain of five compartments with equivalent transition rates *k*_*tr*_ representing the mean maturation time of the neutrophils (De Souza et al., 2018). The proliferation rate of hematopoietic stem cells *k*_*prol*_ is equivalent to the transition rate *k*_*tr*_ guaranteeing homeostasis (De Souza et al., 2018). Deviations from the neutrophil baseline *Base* are compensated by the feedback regulation ${\left(Base/x_{ma}\right)}^{\gamma }$ reflecting the granulocyte colony-stimulating factor (GCSF) controlled proliferation of neutrophils (Friberg et al., 2002; Quartino et al., 2012; Henrich et al., 2017; Jost et al., 2019). As the active forms of both drugs affect the proliferation process, the PD effect is modeled via a linear term with one joint parameter *slope* multiplied to the feedback-regulated first order proliferation rate constant. Other modeling approaches for the incorporation of the PD effect previously showed worst results in model fitting such that we focused on the described term which is additionally more plausible regarding the PD effect, i.g. an impaired proliferation through the incorporation of the metabolized drug into the DNA (Jost et al., 2019). Matured neutrophils die by the process of apoptosis with the rate *k*_*ma*_. A schematic representation of the model is shown in Figure 1 and model constants are listed in Table 2. As no PK biomarkers were measured in the examined dataset, we relied on published PK models and individualized the PD models with respect to individual sets of PD parameters.

**Table 2:** Model constants of the pharmacokinetic model of 6MP and its metabolite 6-TGN from Hawwa et al. (2008), death rate constant of matured neutrophils, and initial conditions of the model (1).

**Constant**	**Value**	**Unit**	**Description/comment**
*F*	0.22		Bioavailability factor
*k*_*a*_	31.2	1/day	Absorption rate constant of 6MP
*k*_20_	12.72	1/day	Elimination rate constant of 6MP
FM_3_	0.019		Fractional metabolic transformation into 6TGN
*k*_*me*_	9.9216	1/day	Metabolic transformation rate constant of 6MP
			into either 6TGN or 6MPN
CL_6*tgn*_(BSA)	0.219 (BSA)^1.16^	L/day	Body surface area (BSA) dependent clearance of
			metabolite 6-TGN
*k*_*ma*_	2.3765	1/day	Death rate of matured neutrophils/leukocytes
*u*(*t*_*i*_)		mg	6MP amount at time point *t*_*i*_
$x_{6mp}^{gut}\left(0\right)$	0	mg	Same initial value for *x*_6*mp*_(0)
*x*_6*tgn*_(0)	0	mg/L	
*x*_*pr*_(0)	(*Base k*_*ma*_)/*k*_*tr*_	G/L	Same initial value for *x*_*tr*1_(0) = *x*_*tr*2_(0) = *x*_*tr*3_(0)
*x*_*ma*_	*Base*	G/L	

In the following, we describe the NLME parameter estimation approach. Therefore, we summarize model (1) for patient *i* as

$$\begin{array}{l} {\dot{x}}_{i}\left(t\right)=f\left(x_{i}\left(t\right),\theta_{i},u_{i}\left(t\right)\right) \end{array}$$

with *u*_*i*_(*t*) the individual treatment schedule and $\theta_{i}={\left({Base}_{i},k_{tr,i},\gamma_{i},{slope}_{i}\right)}^{T}$ the patient specific parameter values of the steady state of neutrophils *Base*, the transition rate *k*_*tr*_, the feedback term γ, and the PD effect *slope*. The vector θ_*i*_ contains the fixed effect parameters *Base, k*_*tr*_, γ and *slope* for all patients and the individual realizations $\eta_{i}\in {\mathbb{R}}^{4},i=1,\ldots ,116$ of the random variable

(4) $$\begin{array}{l} \eta ={\left(\eta_{Base},\eta_{k_{tr}},\eta_{\gamma },\eta_{slope}\right)}^{T}\sim N\left(0,\Omega \right) \end{array}$$

with the mean 0 ∈ ℝ^4^ and the diagonal variance matrix Ω ∈ ℝ^4 × 4^ with the diagonal vector $\omega^{2}={\left(\omega_{1}^{2},\omega_{2}^{2},\omega_{3}^{2},\omega_{4}^{2}\right)}^{T}$. Interindividual variability (IIV) was assumed as log-normally distributed for all four parameters resulting in the following relation between fixed and random effects:

(5) $$\begin{array}{l} {Base}_{i}=Base\;exp\left(\eta_{i,Base}\right) \end{array}$$

(6) $$\begin{array}{l} k_{tr,i}=k_{tr}\;exp\left(\eta_{i,k_{tr}}\right) \end{array}$$

(7) $$\begin{array}{l} \gamma_{i}=\gamma \;exp\left(\eta_{i,\gamma }\right) \end{array}$$

(8) $$\begin{array}{l} {slope}_{i}=slope\;exp\left(\eta_{i,slope}\right) \end{array}$$

summarized as θ_*i*_ = *g*(θ, η_*i*_) and for the description of the residual variability a proportional error model was used

(9) $$\begin{array}{l} y_{ij}=x_{ma}\left(t_{ij}\right)+x_{ma}\left(t_{ij}\right)\;\varepsilon \;i=1,\ldots ,116,\;j=1,\ldots ,n_{i} \end{array}$$

with a normally distributed measurement error $\varepsilon \sim N\left(0,\sigma^{2}\right)$ and ANC count measurements *y*_*ij*_.

The parameters were estimated using the first order conditional estimation (FOCE) method with η-ε interaction. This approximation method results in the parameter estimation method

(10) $$\begin{array}{l} \underset{x_{i}\left(t\right),\theta ,\omega^{2},\sigma^{2},\eta_{i}}{\min}\;\overset{116}{\underset{i=1}{\sum }}L_{i,outer}^{\text{FOCEi}}\left(x_{i}\left(t\right),u_{i}\left(t\right),\theta ,\omega^{2},\sigma^{2},\eta_{1}^{*},\ldots ,\eta_{N}^{*}\right)\\ \;\forall \;t\in \left[0,{t_{\text{f}}}^{i}\right],\;i=1,\ldots ,116\\ \;\text{s.t. }\eta_{i}^{*}={\text{argmin}}_{\eta_{i}}=L_{MAP}^{\text{FOCEi}}\left(x_{i}\left(t\right),u_{i}\left(t\right),\theta ,\omega^{2},\sigma^{2},\eta_{i}\right)\\ \;\forall \;t\in \left[0,{t_{\text{f}}}^{i}\right],\;i=1,\ldots ,116\\ \;\text{s.t. }{\dot{x}}_{i}\left(t\right)=f\left(t,x_{i}\left(t\right),u_{i}\left(t\right),\theta_{i}\right)\\ \;\forall \;t\in \left[0,{t_{\text{f}}}^{i}\right],\;i=1,\ldots ,116\\ \;x_{i}\left(t_{0,i};\theta_{i}\right)=x_{0,i}\left(\theta_{i}\right)\\ \;\forall \;t\in \left[0,{t_{\text{f}}}^{i}\right],\;i=1,\ldots ,116\\ \;\theta_{i}=g\left(\theta ,\eta_{i}\right)\\ \;\forall \;t\in \left[0,{t_{\text{f}}}^{i}\right],\;i=1,\ldots ,116 \end{array}$$

consisting of two nested optimization problems and ${t_{\text{f}}}^{i}$ being the time point of patient *i*'s last ANC measurement. The two parameter estimation problems (estimating θ, ω^2^, σ^2^ with fixed η_*i*_ and vice versa) are iteratively solved until a convergence criterion is fulfilled (Bae and Yim, 2016). For the detailed derivation of the FOCE method with η-ε interaction and the formulation of the resulting objective functions $L_{i,outer}^{\text{FOCEi}}$ and $L_{MAP}^{\text{FOCEi}}$ we refer the interested reader to Wang (2007) and Demidenko (2013) as we confine our analysis on the application of the parameter estimation method.

### 2.3. Out-of-Sample Validation

The reliability of the final population PK/PD model was tested via out-of-sample cross-validation. For each patient, the first 70% of ANC measurements were used for parameter estimation and the final 30% were used to evaluate the model predictions. Model accuracy and predictability were evaluated using the root mean squared error (RMSE) and the mean absolute error (MAE).

### 2.4. Simulation Study

We compared individual simulated minimal, median, and maximal ANCs resulting from the application of different dosing regimens (MT dosage over time). The choice of the different doses described in Table 3 was based on ALL treatment protocols (AIEOP-BFM 2009 with EudraCT number 2007-004270-43, NOPHO-ALL 2008-003235-20, and UKALL 2010-020924-22). In particular, we sought to investigate the relationship between an increased total amount of chemotherapy (higher dosage) and plausibly reduced ANC in the *in silico* simulations. Throughout, we used the fitted models (estimated model parameters) from section 2.2 and only varied the chemotherapy dosage. The simulated ANC values were obtained from the individual actual measurement time points.

**Table 3:** Different dosing protocols for our *in silico* simulation study.

**Nr**	**Description**	**Short**
1	Collected clinical data	(ClinicalData)
2	Fitted model based on patient's actual dosing	(FittedModels)
3	Daily 6MP administration of 25 mg/m^2^ (50% of AIEOP dosis)	(25 mg/m^2^)
4	Daily 6MP administration of 50 mg/m^2^ (AIEOP dosis)	(50 mg/m^2^)
5	Daily 6MP administration of 75 mg/m^2^ (NOPHO/UK dosis)	(75 mg/m^2^)
6	Daily 6MP administration of 100 mg/m^2^ (200% of AIEOP dosis)	(100 mg/m^2^)

*Identical protocols for the administration of 6MP for ClinicalData and FittedModels with a median of the patient-individual average daily dosages of 43.15 ± 10.5 mg/m^2^ (minimum 15.8 mg/m^2^, maximum 72.9 mg/m^2^)*.

### 2.5. Software

The population PK/PD analysis was performed with the NLME modeling program NONMEM 7.4 (ICON Plc., Dublin, Irland) (Beal et al., 2009). There exist several other software packages for parameter estimation of NLME models providing the same or similar algorithms. A variety of algorithms are provided in R Core Team (2019, version 3.6.1). The software Monolix (version 2019R1. Antony, France: Lixoft SAS, 2019) and Diffmem (see https://bitbucket.org/tomhaber/diffmem/src/master/, Melicher et al., 2017) are based on the stochastic approximation expectation maximization algorithm and the recently published package Pumas (based on Julia, see https://pumas.ai/) contains several deterministic and stochastic algorithms. Standard errors were computed with the $COVARIANCE step in NONMEM. Pirana (Certara, Princeton, USA) was used for the generation of the visual predictive check with *auto_bin* option. The simulations in section *out-of-sample validation* and *simulation study* were performed with the ODE integrator CVodes (Sundials, Lawrence Livermore National Laboratory, Livermore) (Hindmarsh et al., 2005) interfaced to CasADi (Optimization in Engineering Center [OPTEC], K.U. Leuven) (Andersson et al., 2019).

## 3. Results

### 3.1. Mathematical Model

Table 4 shows RMSEs, MAEs, and final objective function values for four different parameter estimations. Here, we compared the usage of different PK/PD models and parameter estimations based on either WBC counts or ANCs. First, the explicit consideration of MTX within the PK/PD model of Le et al. (2018) only had a minimal/non-significant effect on the model accuracy, so we fixed it to the ratio 2.5:1 between 6MP and MTX and neglected the PK of MTX in the following. Second, our results showed that the use of the PK model of Hawwa et al. (2008) increased the sensitivity of the PD effect and the model accuracy compared to the 6MP PK model of Jayachandran et al. (2014). Third, ANC measurements resulted in higher accuracy than did WBC measurements.

**Table 4:** Results of parameter estimations for different models.

	**Model 1**	**Model 2**	**Model 3**	**Model 3**
Data	ANC	ANC	ANC	WBC
PK 6MP	Jayachandra	Jayachandra	Hawwa	Hawwa
PK MTX	Panetta	–	–	–
MAE	1.068 (1.65)	1.045 (1.92)	0.9571 (4.31)	1.315 (2.92)
RMSE	1.033 (0.492)	1.022 (0.539)	0.9783 (0.678)	1.147 (0.579)
FinalOBJ	7003	7094	6550	9746^*^

Shown are model characteristics [data based on absolute neutrophil count (ANC) or white blood cell (WBC) count and PK models for 6MP and MTX], median and standard deviation in parentheses of individual root mean squared errors (RMSE), mean absolute errors (MAE), and final objective function values (FinalOBJ). Medians and final objective function values are rounded off to four and the standard deviations in parentheses to three significant figures. *Objective value is not comparable to first three values due to different dataset.

### 3.2. Parameter Estimation

**Figure 2:**
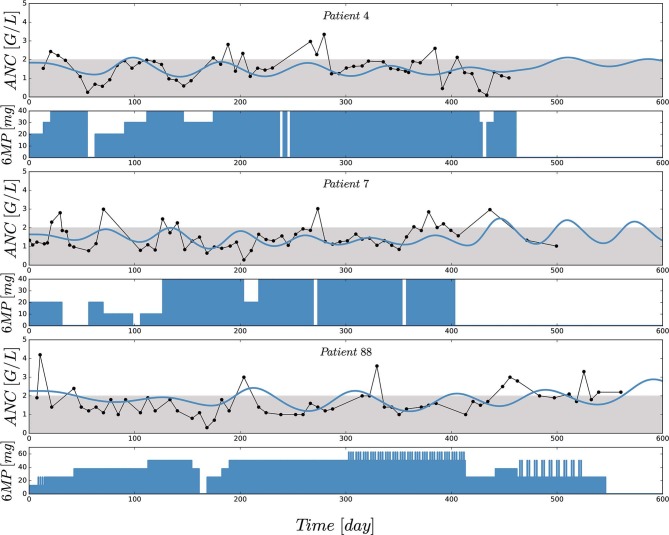
Comparisons of observed (black) and individually simulated (blue) absolute neutrophil counts (ANCs) [G/L] for three exemplary chosen patients presented in rows 1,3 and 5. Simulations of the ANCs (*x*_*ma*_) were performed with the newly proposed mathematical model (1) after nonlinear mixed-effects parameter estimation. Based on a visual assessment, the model captures the trends of the chemotherapy induced myelosuppression (compared with the indicators in Table 4). For each patient, the individual 6-mercaptopurine (6MP [mg]) dosing protocol is presented in rows 2, 4 and 6, indicating dose changes for efficacy adjustments. The daily oral 6MP administration, ranging from 10 to 60 mg for the three patients, are presented as filled areas and corresponds to the control function *u*(*t*) in model (1).

**Figure 3:**
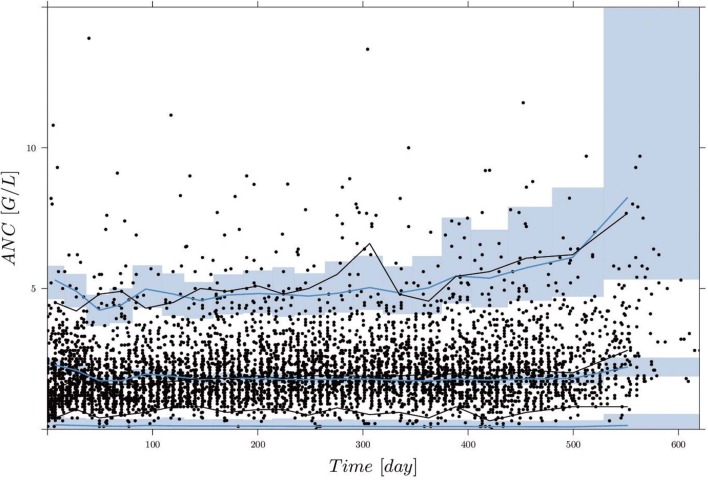
Visual predictive check (VPC), derived by 1,000 simulations with the final parameter estimates from the first column of Table 5, for circulating ANCs (G/L) vs. time (days). Black dots are the measured ANCs. Black and blue lines show the median and 2.5th and 97.5th percentiles of measurements and model predictions, respectively. The shaded areas represent the 95% confidence intervals around the 2.5, 50, and 97.5th percentiles of the model predictions. Two ANC outliers (19.9 and 17.8) at time points 285.42 and 340.42 days are not shown.

Figure 2 shows the comparisons of observed clinical and simulated ANCs derived from the final PK/PD model (1) after parameter estimation for three exemplary chosen patients presented in rows 1,3 and 5. For each patient, the individual 6-mercaptopurine (6MP [mg]) dosing protocol is presented in rows 2, 4 and 6, indicating dose changes for efficacy adjustments. The model simulations represented the clinical ANCs quite well in the average and captured trends toward larger or smaller ANC values. However, they did not oscillate as strongly as the measured values. Persistent oscillations of neutrophils often occur in chemotherapy-treated hematopoietic diseases inducing cyclic myelosuppression (see Knauer et al., 2019 and references therein). Several othter reasons were responsible for the observed ANC oscillations such as aberrant hematopoiesis, chemotherapeutic dose adaptations, infections or measurement errors. This exemplary behavior was representative of the entire data set of 116 patients. The visual predictive check plot in Figure 3 shows the good agreement of model response and measurements for the median (solid line) and 97.5th percentile (dashed line) with a slight underprediction of the model for low ANC values. The 95% confidence interval of the model simulation median was very thin, indicative of high prediction accuracy. The fixed effect estimate for the ANC steady state was slightly higher than the target range limit of 2 G/L. The estimated transition rate of 0.148 resulted in a mean maturation time (MMT = *n*_*tr*_/*k*_*tr*_) of 487 h (20.3 days) (De Souza et al., 2018). The interindividual variability and residual error were within reasonable ranges.

The goodness-of-fit plot in Supplemental Data shows the results of out-of-sample cross-validation. It reflected reasonable model accuracy for fitted (blue) and predicted (red) ANC measurements with spreading around the line of identity because the model was not able (and not intended) to hit the lower and upper peaks of the measurements. The values of estimated model parameters both for the in-sample and out-of-sample calculations are shown in Table 5. The parameter values for *slope* and *Base* were reduced and the value of γ was slightly increased for the estimates based on 70% of the ANC. The interindividual variability (IIV) for the *slope* was significantly larger whereas the IIV of *k*_*tr*_ was smaller. To evaluate the model accuracy, we calculated the median and standard deviation of the individual MAEs and RMSEs, showing the expected decrease in accuracy for out-of-sample predictions.

**Table 5:** Results of parameter estimations of the final model using all (in-sample) or 70% (out-of-sample) of the ANC values.

**Data**	**In-sample**	**Out-of-sample**
**Fixed effect parameters**		
*Base*	2.34 (1)	2.06 (0.1)
k_*tr*_	0.148 (0.4)	0.146 (0.2)
*slope*	0.242 (0.2)	0.103 (0.2)
γ	0.769 (0.1)	0.866 (0.2)
**Interindividual variability as coefficients of variation**		
*Base*	23.1 (20)	27.5 (10)
k_*tr*_	16.5 (30)	7.19 (3)
*slope*	44.9 (5)	67.8 (1)
γ	10.7 (0.5)	16.5 (0.4)
Proportional additive error	0.226 (2)	0.226 (FIXED)
**Parameter estimation errors**		
Mean absolute error	0.957 (4)	1.47 (500)
Root mean squared error	0.978 (0.7)	1.21 (7)

*Shown are parameter estimates of fixed effects, interindividual variability as coefficient of variation, proportional additive error as variance, and median errors of the parameter estimations rounded off to three significant figures. For the mean absolute and root mean squared errors all ANC measurements are used. Relative standard errors are shown in parentheses rounded off to one significant figure*.

### 3.3. Simulation

**Figure 4:**
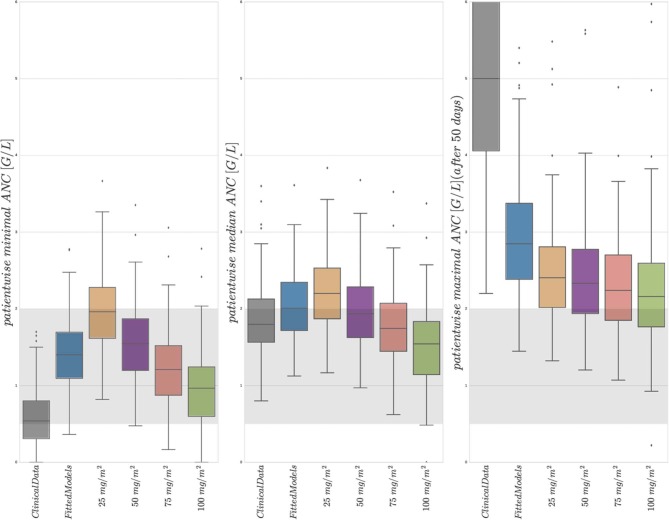
Boxplots of minimal, median, and maximal (from left to right) individual ANCs for all 116 patients. Shown are values for the 6 different protocols from Table 3, observed for the first column and simulated for protocols 2–6. The target range (0.5–2.0 G/L) of the NOPHO/UK treatment protocol is shown as the gray background. Horizontal lines within the boxes are the medians, the upper and lower box limits are the first and third quartiles of the data, respectively. The whiskers indicate an even larger confidence region of these quartiles plus/minus 1.5-times the interquartile range. Beyond the whiskers, data are considered as outliers and are plotted as individual points. For the columns representing 25 mg/m^2^ to 100 mg/m^2^, the total amount of 6MP administered is increasing. The median average individual daily doses actually administered for protocols 1 and 2 were 43.15±10.5 mg/m^2^.

Figure 4 shows boxplot results for an *in silico* simulation study based on the 6 different treatment protocols (including the real clinical data) from Table 3. We want to stress three main observations.

First, a comparison of the first two entries of the three boxplots confirmed an already known result. The personalized models could reproduce the clinical ANC data on average quite well, with the exception of extreme values quantitatively confirming the observation made in Figure 2. Given the similarity of simulated and observed median values, we continued with an objective comparison only of the simulated results (protocols 2–6).

Second, a comparison of the protocols 3–6 (25, 50, 75, and 100 mg/m^2^ BSA 6MP) showed a significant and linear dosage-effect relationship with respect to the total amount of 6MP administered, which is, of course, proportional to the daily dose. All (minimal, median and maximal) ANC values decreased linearly, when daily dosing was increased linearly.

**Figure 5:**
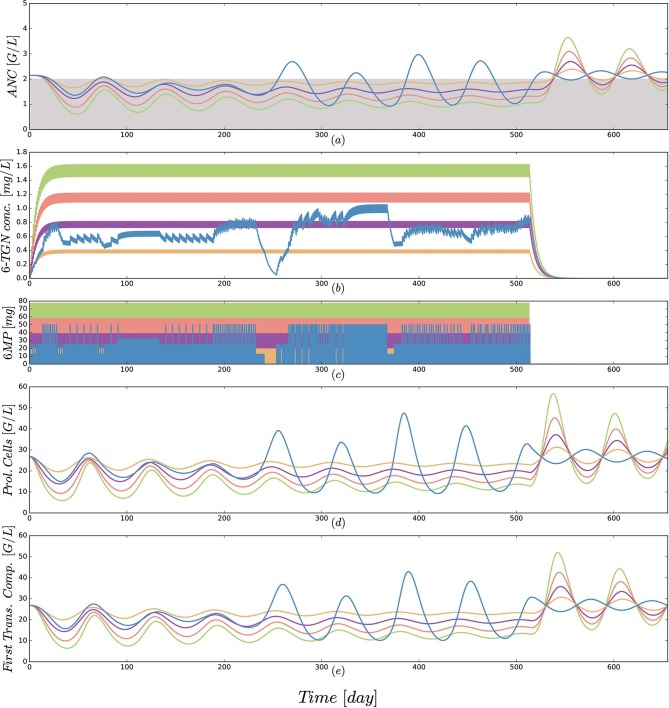
**(a)** Simulated absolute neutrophil count (ANC) dynamics [equal to *x*_*ma*_ in Equation (1)], **(b)** concentration of the active form 6-TGN [equal to *x*_6*tgn*_ in Equation (1)], **(c)** 6MP dosing amount [control function *u*(*t*) in Equation (1)], **(d)** dynamics of proliferating cells [equal to *x*_*pr*_ in Equation (1)] and **(e)** cells of the first transition compartment [equal to *x*_*tr*1_ in Equation (1)] for 5 different protocols from Table 3 and an exemplary patient. Colors of the trajectories are identical to those used in Figure 4. The linear increase in dosing from 25 to 100 mg/m^2^ forces the neutrophils (ANC) to lower peak values and a smaller drug-induced steady state value at the end of treatment. The actual dosage administered to the patient (blue) ranged between the 25 mg/m^2^ and 50 mg/m^2^ protocols and resulted in similar ANC dynamics. At approximately day 240, the actual dosing was stopped for a short period, inducing stronger ANC oscillations in the subsequent treatment period. This observation is even stronger regarding the proliferating cells as well as cells in the first transit compartment. Interestingly, these oscillations also continued for some time after the end of treatment. Due to the long simulation horizon, the 6-TGN dynamics are squeezed such that the concentrations between two administrations are not visible.

Third, a comparison of protocol 2 (the simulation of the real treatment) and protocols 3 and 4 (which gave lower and upper bounds on the total amount of administered 6MP in protocol 2, respectively) showed that the median ANC value of protocol 2 was indeed bounded by the two other values, however, for significantly lower minimal and higher maximal ANC values. Figure 5 shows an exemplary comparison of protocols 2–6 for one patient, highlighting lower peak values and smaller drug-induced steady state values when the dosing is linearly increased from 25 mg/m^2^ to 100 mg/m^2^. The actual dosage administered to the patient (blue) ranged between the 25 mg/m^2^ and 50 mg/m^2^ protocols and resulted in similar ANC dynamics. At approximately day 240, the actual dosing was stopped for a short period, inducing stronger ANC oscillations in the subsequent treatment period and revealing a significant impact of the dosing regimen on the ANCs. This observation is even stronger regarding the proliferating cells as well as cells in the first transit compartment. Similar plots for all 116 patients are provided in the Supplemental Data.

## 4. Discussion

### 4.1. Mathematical Model

We developed and fitted a population PK/PD model to assess the ANC dynamics during 6MP/MTX treatment, get a better understanding of dose adjustments, and identify solutions to the challenges that arise throughout MT. During the model development process we also fitted the model to WBC measurements. The resulting MAEs and RMSEs were worse compared to the values resulting from ANC measurements. This is probably due to the fact that WBCs comprise different cell lineages, with additional physiological effects that are not accounted for in the mathematical model. In future studies, the current model might be extended to further cell lineages. The models brought forth by Quartino et al. (2012) and Fornari et al. (2019) might serve as a basis and drive the modeling process from a semi-mechanistic approach toward a more mechanistic one.

In addition to using a population estimation approach and applying it to ANC instead of WBC, two modifications brought forth by Le et al. (2018) were shown to yield better results. First, the 6MP PK model of Jayachandran et al. (2014) was replaced by that of Hawwa et al. (2008). The first order kinetics in the PK model of Hawwa et al. (2008) compared to the Michaelis–Menten terms in the PK model of Jayachandran et al. (2014) resulted in more significant concentration changes with altered drug amounts consequently in a more sensitive PD effect. Second, the MTX PK model was completely omitted as the constant ratio of administered 6MP and MTX prevents a differentiation of separate PD effects. Further studies with measurements of drug concentrations, metabolites and clinical effects as cell counts would push forward the development of a mathematical model additionally including the PK of MTX to provide two distinct PD effects and to account for varying ratios of 6MP to MTX. For the currently available data, our new model, which indirectly agglomerates the effects of 6MP and MTX, appears to be a good choice (compare for Table 4).

### 4.2. Model Parameter Estimates

Looking at the resulting model parameter estimates listed in Table 5, the question arises as to how these values relate to known biological properties of hematopoiesis and myelosuppression and to other values from the literature. The estimated ANC steady state value *Base* was below the normal ANC range for children, but still higher than the desired ANC range of 0.5–2 G/L. Without treatment, the model-based ANCs would increase to normal patient-specific steady states. Thus, low ANC values were induced via MT or some of the aforementioned external events.

The estimated fixed-effects parameter value of the transition rate *k*_*tr*_ = 0.148 was comparable with the published mean value ($\bar{k_{tr}}=0.1431$) obtained from eight pediatric ALL patients from Riley Hospital for Children in Indianapolis (Jayachandran et al., 2014). For better interpretability, the transition rate parameter *k*_*tr*_ can be transformed to the MMT (*n*_*tr*_/*k*_*tr*_) of the neutrophils. The estimated MMT in our study, as well as the MMT from Jayachandran et al., are extremely high and do not coincide with biological findings of 3.9 days obtained by Hearn et al. (1998). This mismatch is a large disadvantage of the model as it fails to comply with biological properties, leading to falsely characterized physiological mechanisms and thus reduced model reliability. Jayachandran et al. did not discuss this issue, but a similar observation was made by Craig et al. (2016) who determined an estimated proliferation time of 26 days (Craig et al., 2016). In their work, the authors further presented model modifications to obtain a more realistic maturation time of 3.9 days. For this value we performed two parameter estimations with either *Base* as a parameter or fixed to 5 resulting in promising dynamics but worse RMSEs and MAEs. In future studies, the falsely determined MMT and possible model limitations for continuous low-dose treatments should be further investigated.

The feedback parameter (γ) is significantly higher compared with published values (Friberg et al., 2002), indicating a stronger feedback mechanism during the daily chemotherapy over a long period. This is the first time estimated *slope* values of the linear PD function from the PK model of Hawwa et al. (2008) are presented; thus there are no available comparisons.

### 4.3. Simulation Results

The newly developed mathematical model enables us to perform a virtual comparison of different treatment protocols. The boxplots in Figure 4 show several interesting results.

First, the median and standard deviation of actual ANC measurements were very accurately matched by the simulation using the estimated parameters (compare the first two entries in the middle boxplot of Figure 4). Concerning the patientwise observed and simulated minimal and maximal ANC values, the model demonstrates a corresponding weakened chemotherapy-induced myelosuppression, respectively overproduction of ANCs compared to the high measured variability. This variability is biologically and clinically very plausible due to the aforementioned external events and uncertainties, although periods of severe infections were already excluded. The reproducibility of the median and avoidance of over-fitting of the extreme values are in our opinion good properties of a mathematical model. Given this good correspondence between cross-validated data and simulations, we felt encouraged to compare simulations of different treatment protocols as specified in Table 3. Note, however, that generalizations of mathematical models personalized for data from one protocol to another have to be considered with extreme care (compare the discussion for acute myeloid leukemia models by Jost et al., 2019). Further, we want to highlight that the current model is not intended to describe the ANC extrema such that the results of the simulation study have to be treated with caution. The results shall serve as a preliminary assessment of the dose-effect relationship which has to be confirmed in future studies. The relationship might be stronger compared to the current model predictions and demonstrated by the clinical data in Figure 5. The impact of model variations on the outcome of simulation studies is usually significant. We tested the value of fixing the *k*_*tr*_ parameter to represent a biologically plausible MMT of 3.9 days. This decreased the model accuracy (which is why the results are not included here), but still led qualitatively to the same subsequent effects.

Second, an approximately linear decrease in minimal, median and maximal values could be observed as the dosage increased linearly from 25 to 100 mg/m^2^ with a slightly reduced decrease of the maximal ANC values. Again, this linear dose-effect relationship seems biologically plausible. For most of the simulations such as those shown in Figure 5, the maximal ANC value decreased. However, for other simulations (see Supplementary Material) stronger myelosuppression led to identical maximal ANC values. This effect is due to a feedback mechanism that may lead to increased proliferation for reduced ANC which leads to larger ANC values after some delay.

Third, a tendency for higher oscillations for treatments with pauses and changes in dosage was seen in a comparison of the simulated actual treatment protocol 2 and the constant administrations of protocols 3 and 4, which used lower/higher total amounts of 6MP. Again, an example of this can be seen in Figure 5. We believe that in the future adapted dosing schedules might take advantage of the chemotherapy-induced oscillations for an optimized dosing regimen. In the consolidation therapy of acute myeloid leukemia it was shown *in silico* that the timing of the treatment start can have a beneficial influence on the reduction of myelosuppression (Jost et al., 2019). However, high dose chemotherapy administered every 3 to 4 weeks provokes stronger periodic oscillations compared to the daily oral dosing which makes it more challenging to identify and capture the oscillations. For high dosage, previously a multi-compartment hematopoietic model was analayzed regarding Hopf bifurcation and an explicit analytical expression for the bifurcation point was provided depending on model parameters (Knauer et al., 2019). Oscillations of various blood cell populations have been observed in clinical data and partly investigated for different hematological disorders (Haurie et al., 1998; Colijn et al., 2006). The exact mechanisms and interaction between (1) stem cell cycling, (2) hematological disorder, and (3) drug exposure are still not fully understood. In our case, for all 116 patients *in silico* simulations showed that the oscillations were damped (in 84 cases into a steady state) once the chemotherapy was stopped, albeit with long time ranges of up to one year (see Supplemental Data for examples). Therefore, we assume that the oscillations in the ANCs observed in our simulations could be attributed to the influence of chemotherapy on the nonlinear dynamics of hematopoiesis. The connection between *model-intrinsic* and *chemotherapy-induced* oscillations should be assessed in detail in future studies. A stability analysis (Edelstein-Keshet, 2005) of the steady state could be performed (e.g., similar to Stiehl, 2014; Tetschke et al., 2018) to assess the theoretical properties of the model and relate them to the physiological behavior of neutrophils.

## 5. Conclusion

We presented a novel NLME model describing myelosuppression for ALL MT among children who received 6MP and MTX and was cross-validated on a data set of 4747 ANC measurements obtained from 116 patients. A comparison with alternative modeling approaches and using WBC counts instead of ANCs showed the benefit of this model. We could show a linear dose-effect relationship superimposed with fluctuations of varying magnitude. Mathematical simulations and more mechanistic modeling approaches will allow to improve the understanding of intrinsic and extrinsic influence factors on the aberrant hematopoiesis and chemotherapy-induced myelosuppression of pediatric ALL patients. Therefore, the monitoring of individual PK profiles and a subsequent analysis of the PK/PD relationship are mandatory next steps for a better dose-effect correlation.

In the future, based on the conduction of further PK and PD experiments driving the development of more advanced mathematical models together with the individual determination of response-related genotyping (Tsujimoto et al., 2018), MT protocols might be developed *in silico*, leading to individualized treatment protocols with better clinical outcomes.

## Data Availability Statement

The datasets generated from the parameter estimation process can be found in the Supplemental Material.

## Author Contributions

FJ developed the population PK/PD models, performed the numerical computations, and wrote the first draft of the manuscript. JZ, TL, TR, MR, MM, and SS contributed to the model development, the study designs, and the interpretation of the results. MSu and MSt provided clinical data. All authors contributed to writing the final manuscript.

### Conflict of Interest

The authors declare that the research was conducted in the absence of any commercial or financial relationships that could be construed as a potential conflict of interest.

## Supplementary Material

Goodness-of-fit plot presenting observed vs. individually calculated absolute neutrophil counts for 116 patients (see Supplemental Data 1).

Simulations of protocols 2–6 for all 116 patients, similar to Figure 5 (see Supplemental Data 1).

Simulations of protocols 2 for all 116 patients to analyze the steady state behavior (see Supplemental Data 2).

The individual parameter estimates of the final PK/PD model are recorded in the file *finalParameterEstimates191221.csv*.

The individual 6-mercaptopurine (6MP) treatment schedules, the observed and calculated absolute neutrophil counts (ANCs) and the patient's weight, height and body surface area are recorded in the file *NONMEMresultsALL191221.csv*. The dataset contains the columns ID, TIME, DV, CMT, AMT, MDV, EVID, IPRED, WEIGHT, HEIGHT and BSA. ID serves as an identifier for the appropriate patient. TIME [day] either specifies the measurement times of ANCs or the time of oral 6MP administrations. DV [G/L] is the dependent variable, in our case the individual ANC measurements. The column CMT specifies the compartment in which a dosing or observation event occurs. AMT [mg] defines the amount of oral 6MP administration. The column MDV allows the user to inform NONMEM whether or not the value in the DV field is missing, but in our case the datasets do not contain missing measurements. The column EVID explicitly declares to NONMEM the type of the current record. EVID=0 defines the record as an observation event and EVID=1 defines the record as a dose event. The column IPRED contains the calculated ANCs derived by the PK/PD model after parameter estimation.
