# Supplementary material for: Model-Based Simulation of Maintenance Therapy of Childhood Acute Lymphoblastic Leukemia
Source: Front Physiol. 2020 Mar 18;11:217. doi: 10.3389/fphys.2020.00217 (PMC7093595; doi:10.3389/fphys.2020.00217)
Supplement: Supplementary file 3 [file Data_Sheet_3.PDF]

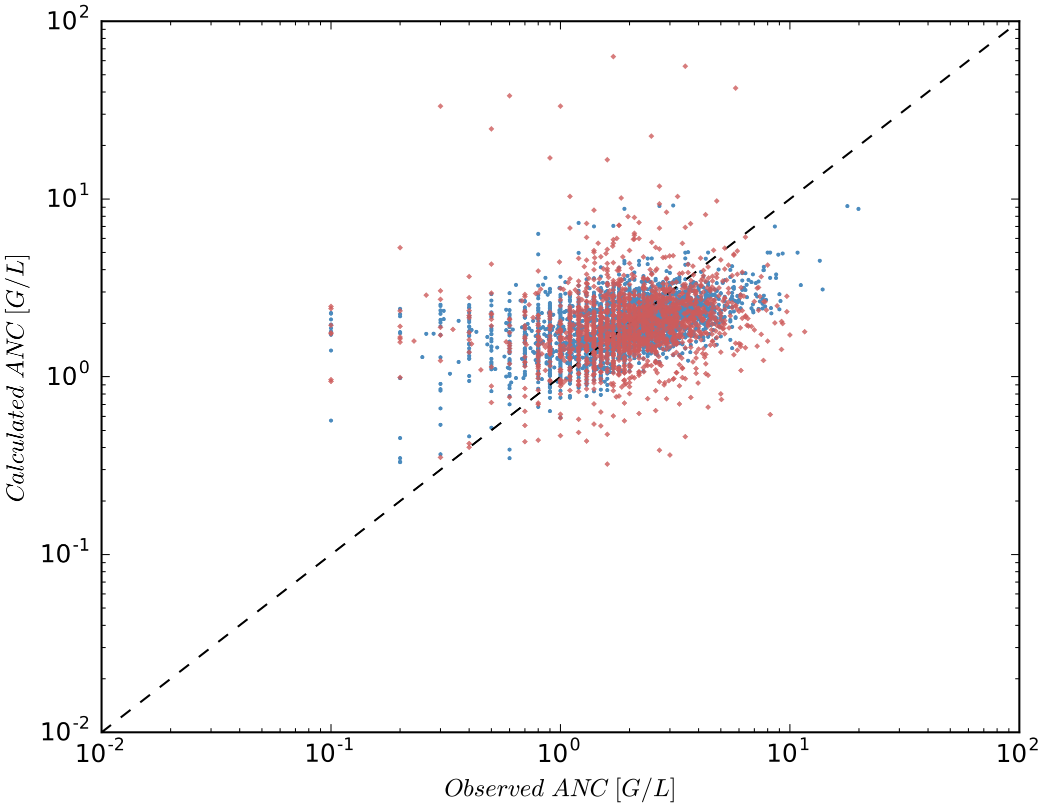

Figure 1: Goodness-of-fit plot depicting observed versus individually calculated absolute neutrophil counts (ANCs) for 116 patients. The calculated ANCs were derived from individual simulations of the final PK/PD model (1) with estimated parameters using the first 70% of measured ANCs (compare column *in-sample* in Table 5). Blue markers show the comparison between the first 70% of observed/measured (x-axis) and calculated (y-axis) ANCs used for parameter estimation. Models were cross-validated using the last 30% of observed ANCs (red markers) which were not considered during parameter estimation (out-of-sample). The goodness-of-fit plot provides a visual assessment of the model capability to describe and predict the clinical data with increasing accuracy the closer the markers come to the line of identity. The in-sample as well as out-of-sample markers quantify the obersation from Figure 2 that in general the model captures the trends of the ANC dynamics, but a perfect match could not be achieved.

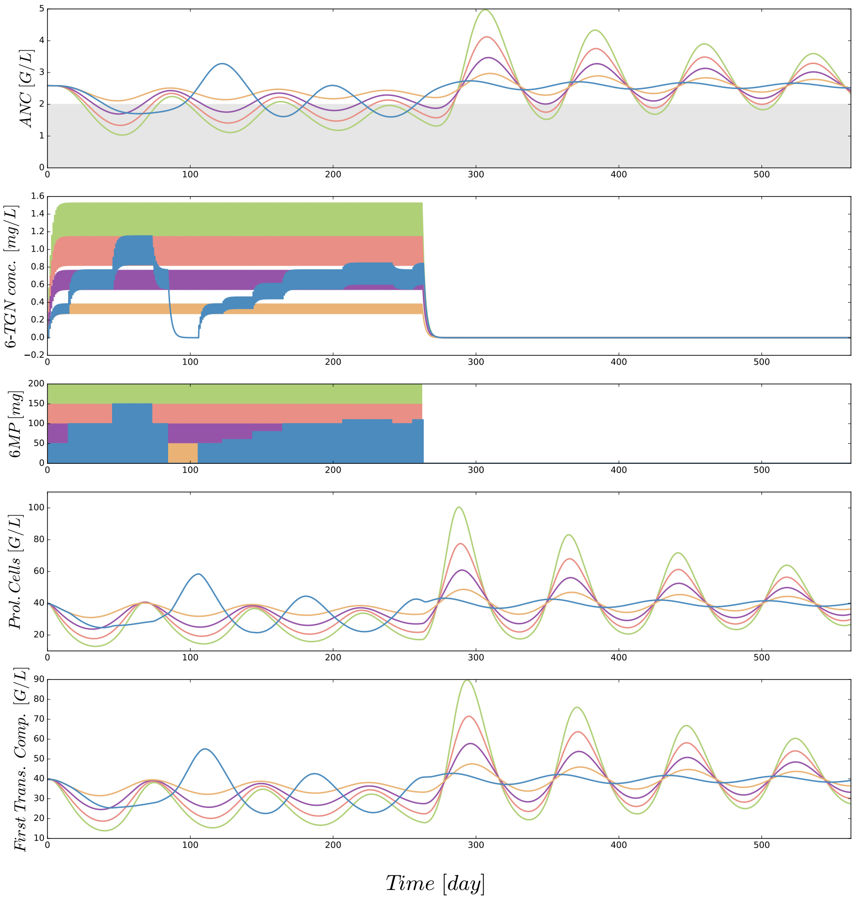

Figure 2: Simulated trajectories of the first patient for 5 different protocols from Table 2. Colors of the trajectories are identical to those used in Figure 4.

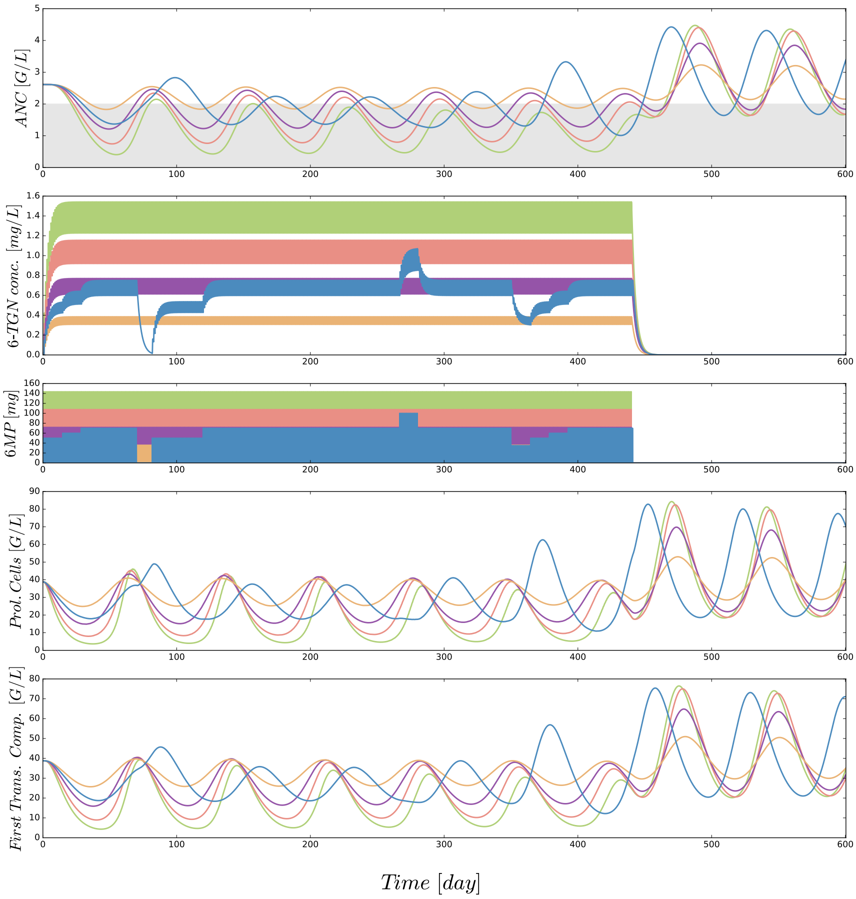

Figure 3: As Figure 2, but for another patient out of 116 patients.

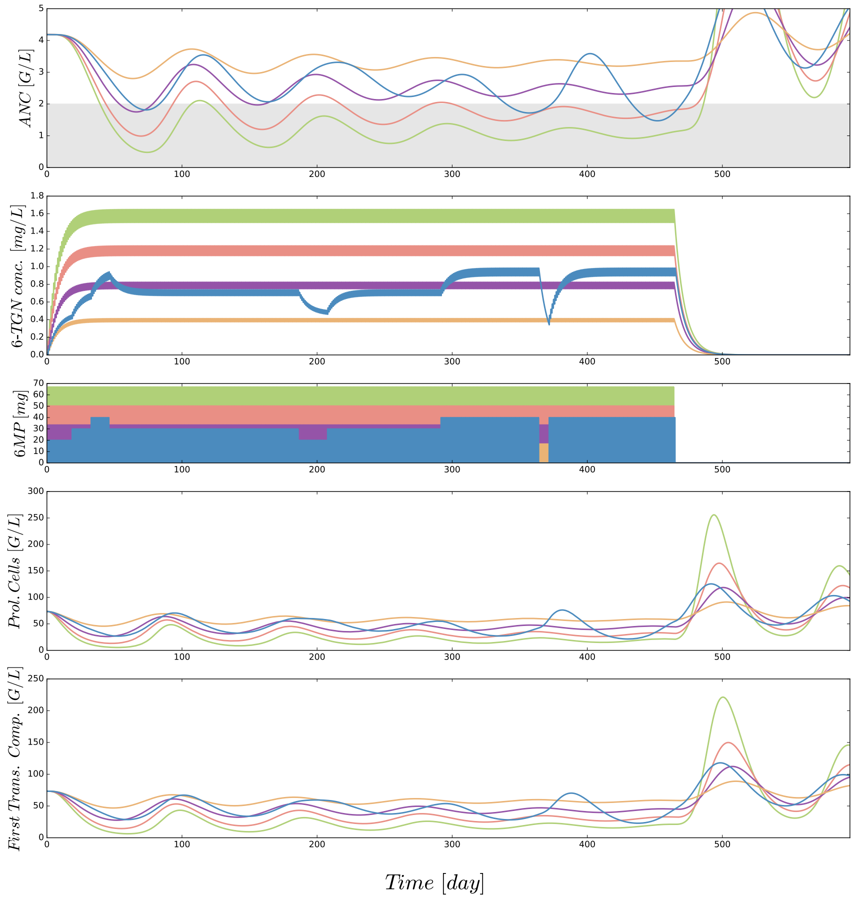

Figure 4: As Figure 2, but for another patient out of 116 patients.

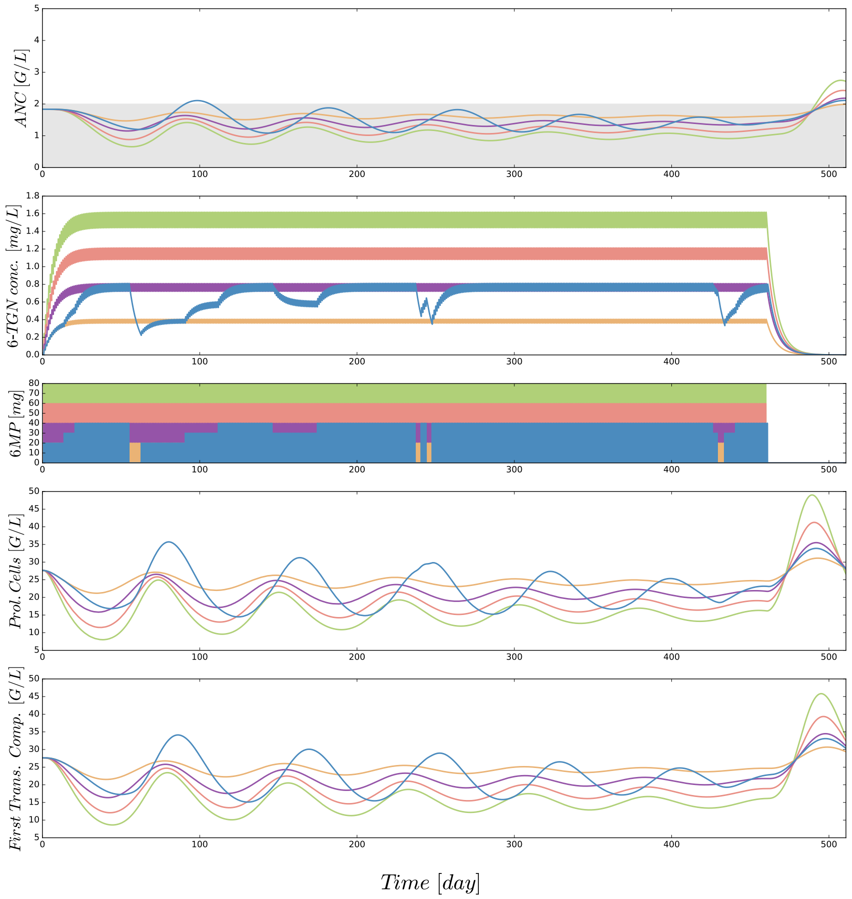

Figure 5: As Figure 2, but for another patient out of 116 patients.

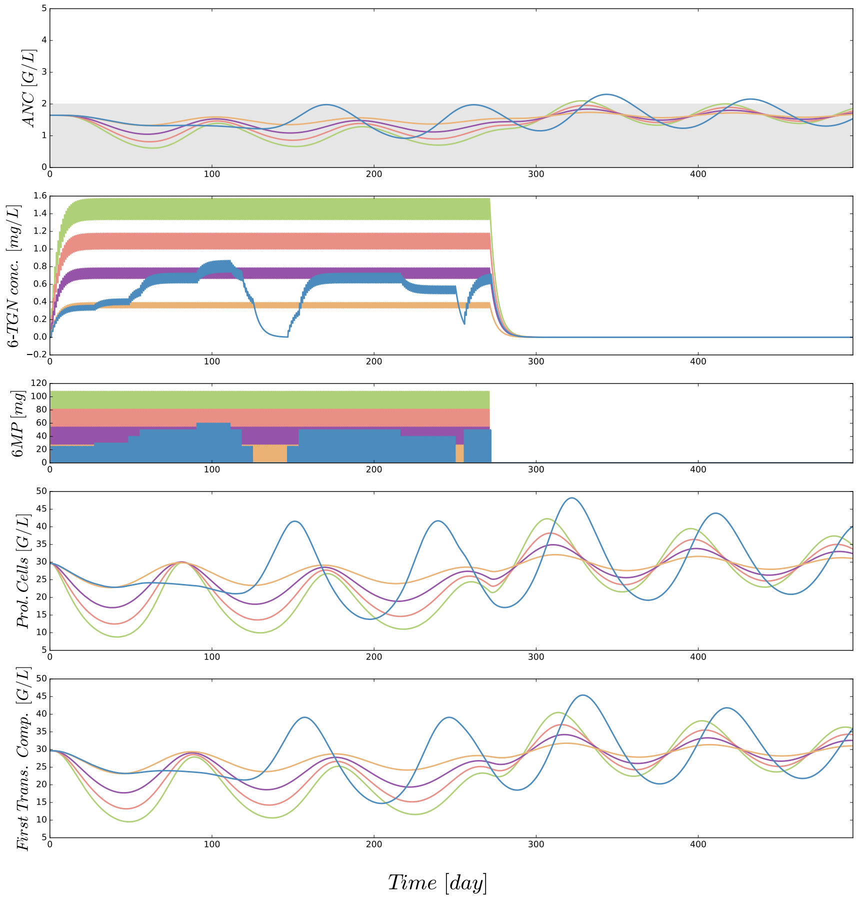

Figure 6: As Figure 2, but for another patient out of 116 patients.

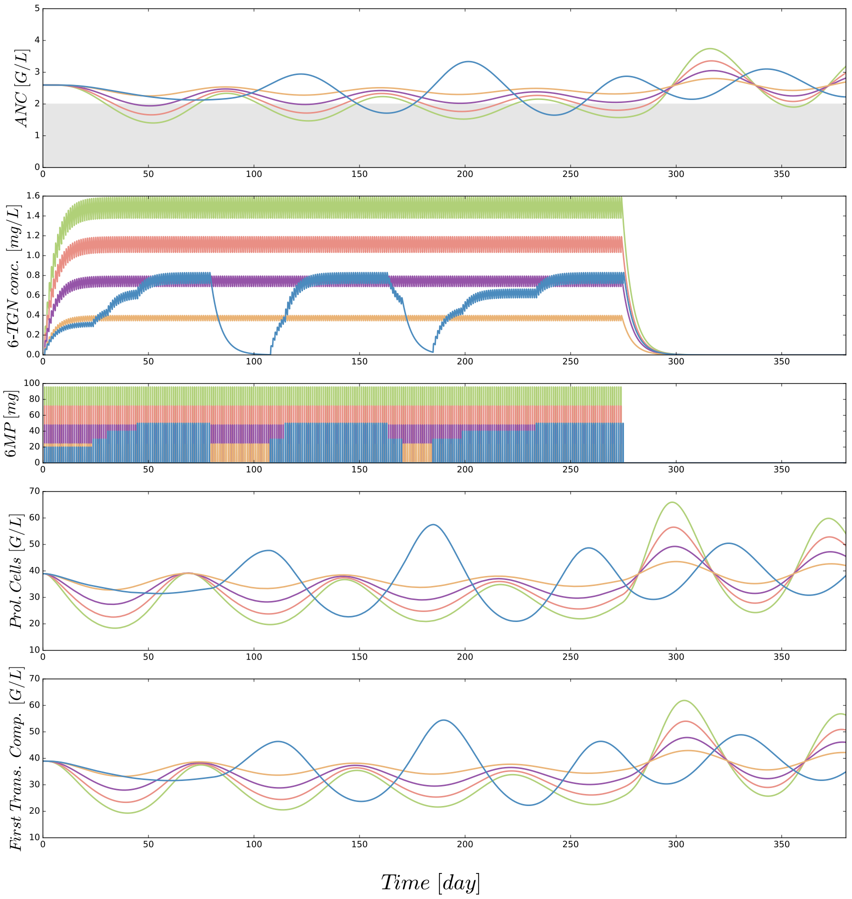

Figure 7: As Figure 2, but for another patient out of 116 patients.

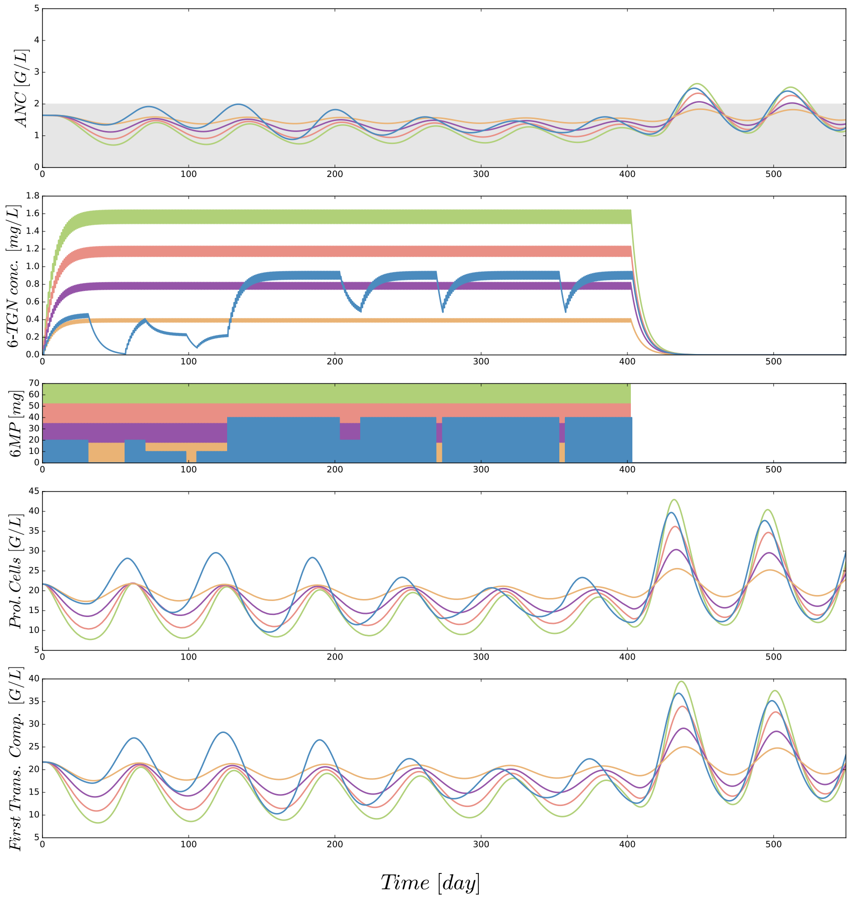

Figure 8: As Figure 2, but for another patient out of 116 patients.

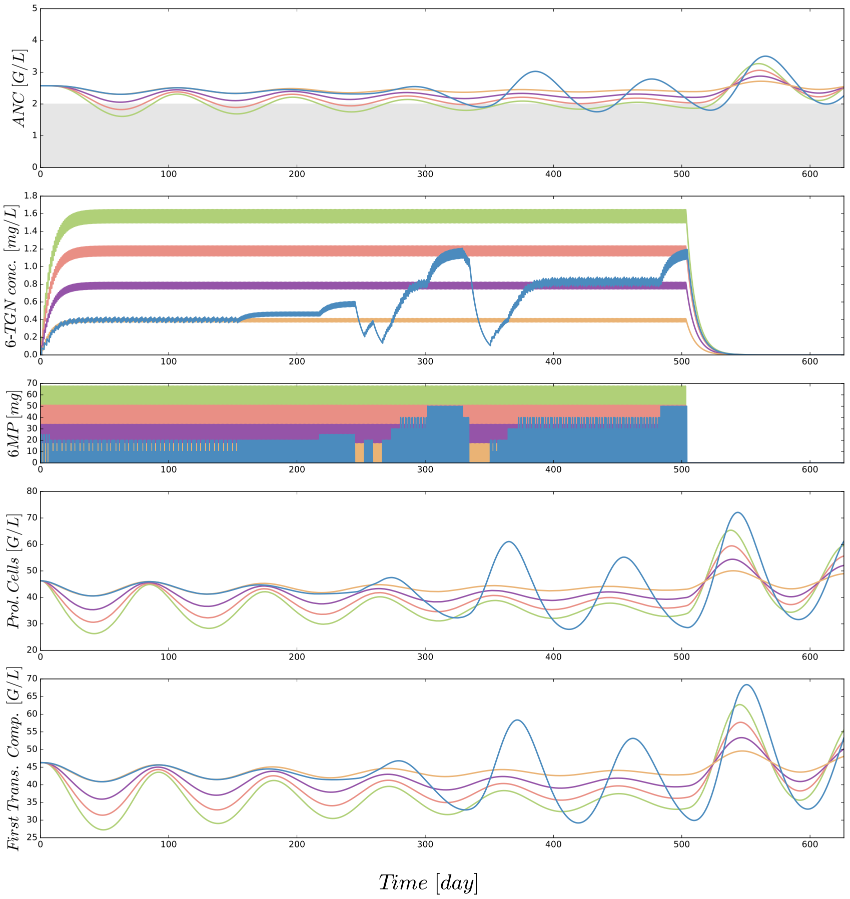

Figure 9: As Figure 2, but for another patient out of 116 patients.

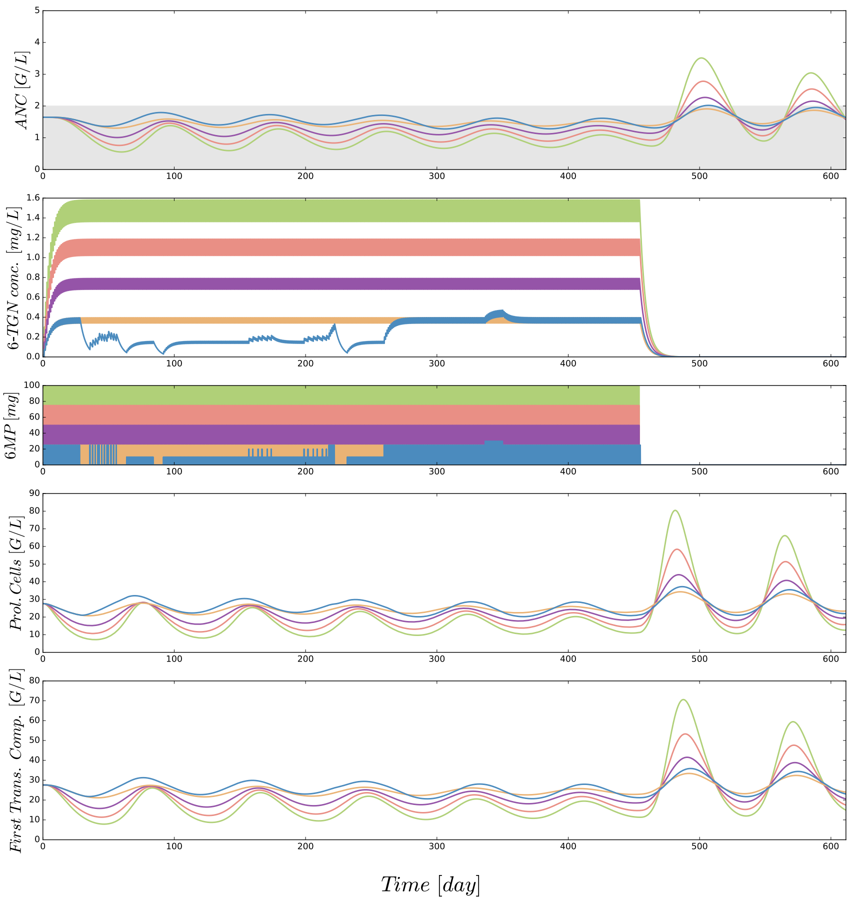

Figure 10: As Figure 2, but for another patient out of 116 patients.

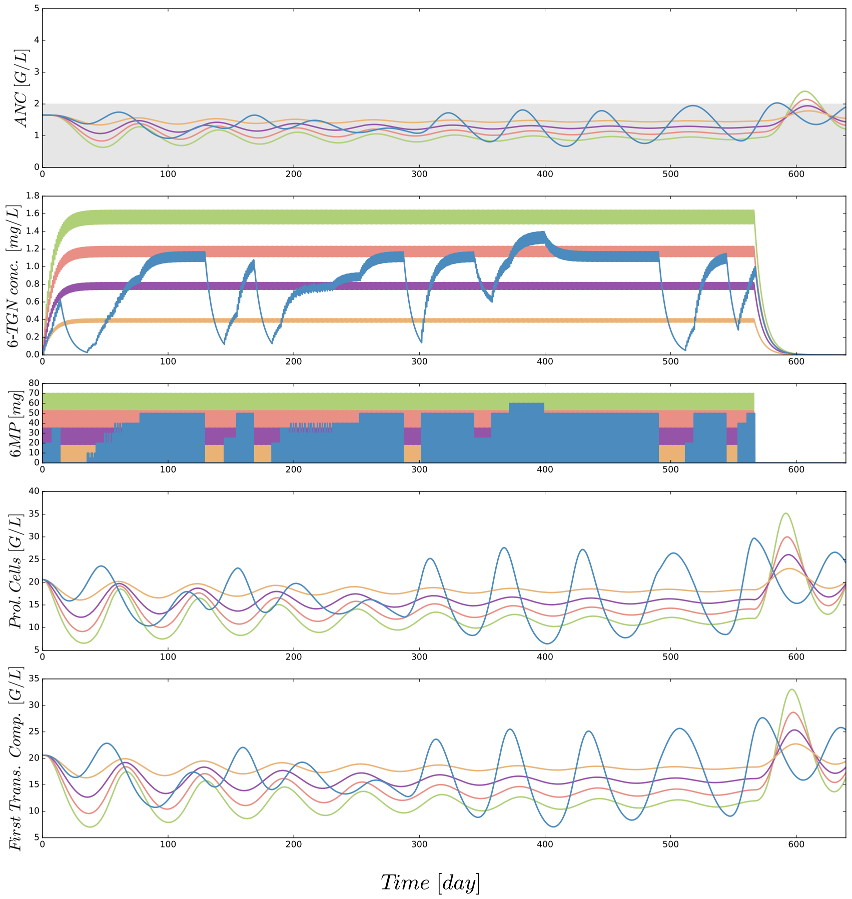

Figure 11: As Figure 2, but for another patient out of 116 patients.

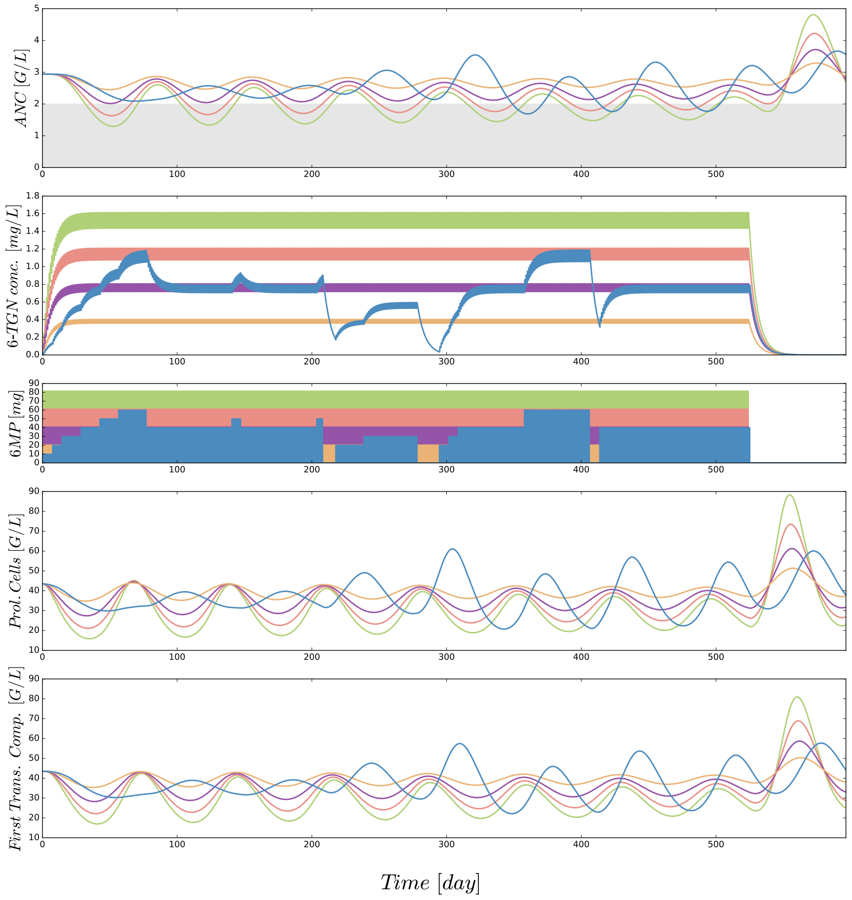

Figure 12: As Figure 2, but for another patient out of 116 patients.

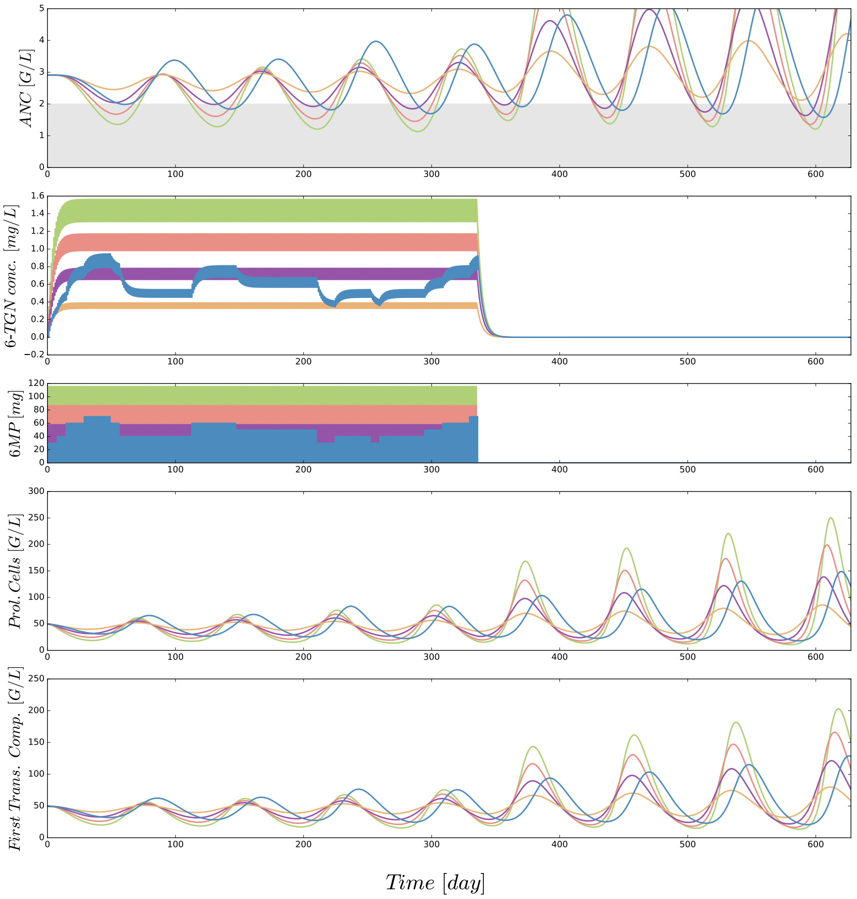

Figure 13: As Figure 2, but for another patient out of 116 patients.

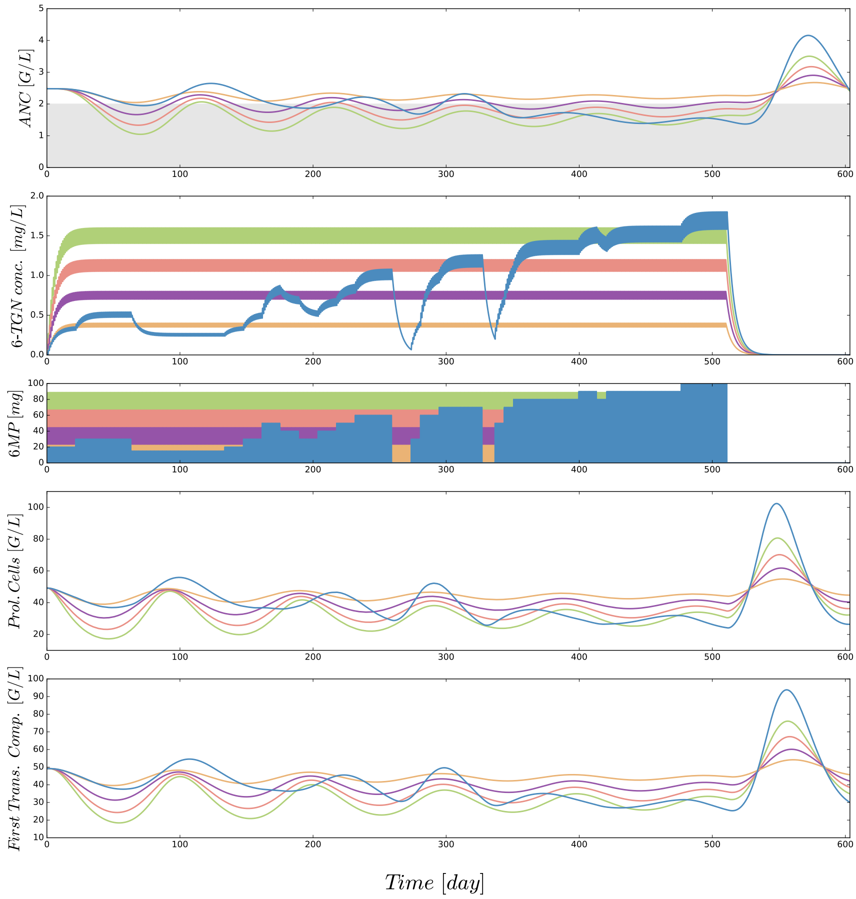

Figure 14: As Figure 2, but for another patient out of 116 patients.

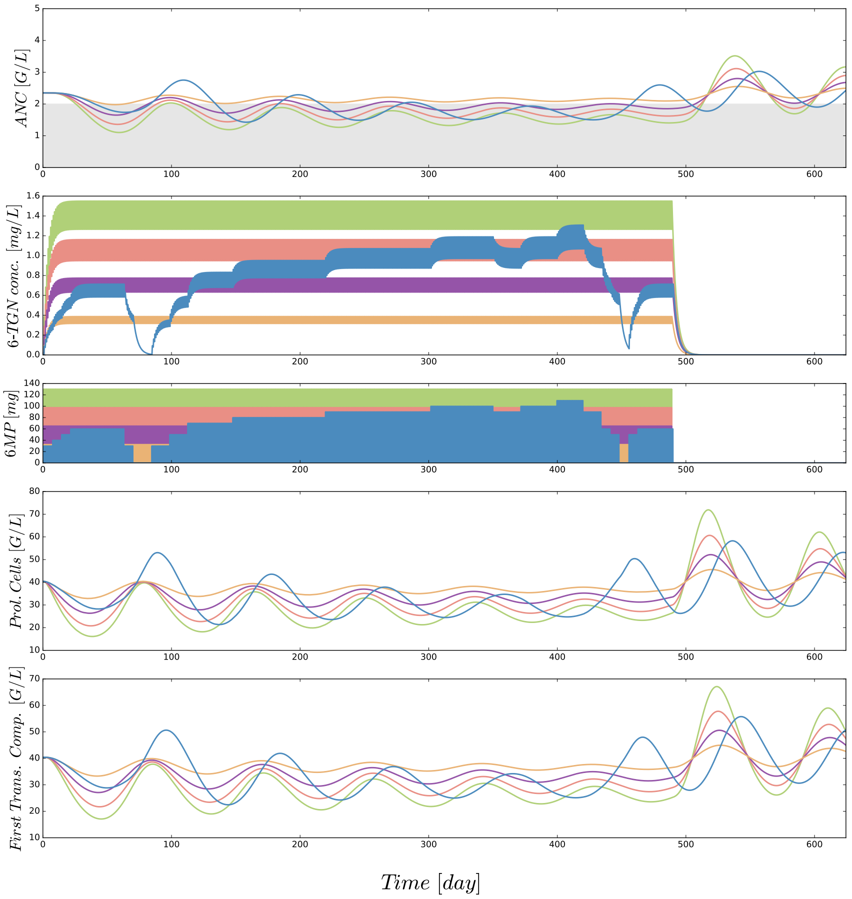

Figure 15: As Figure 2, but for another patient out of 116 patients.

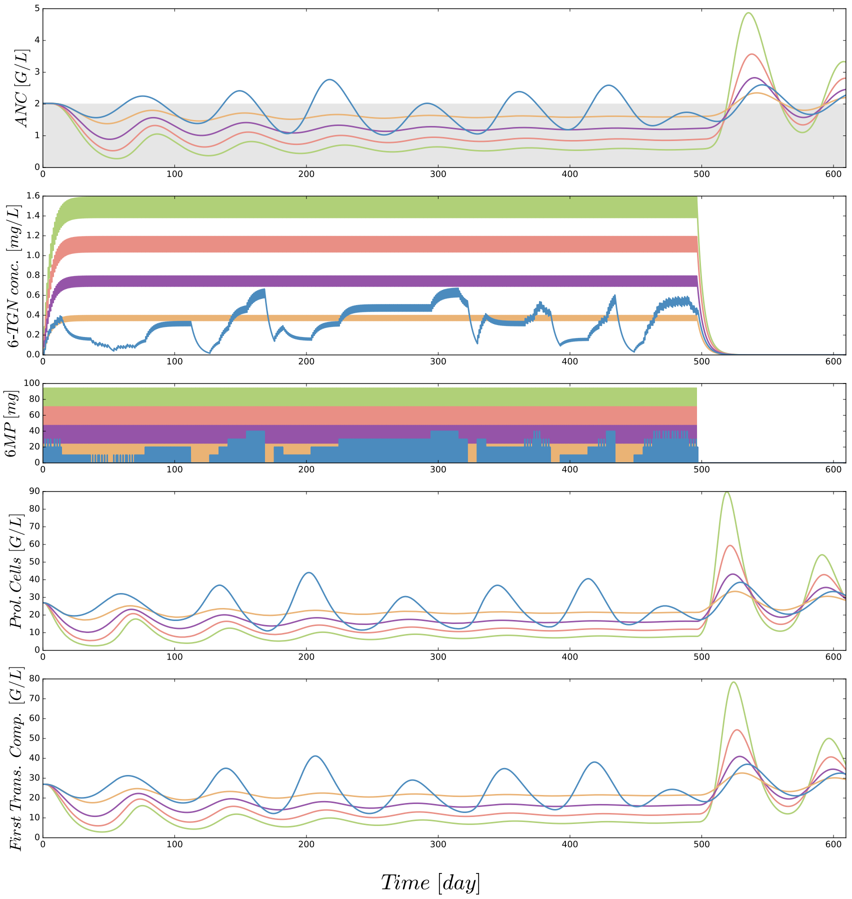

Figure 16: As Figure 2, but for another patient out of 116 patients.

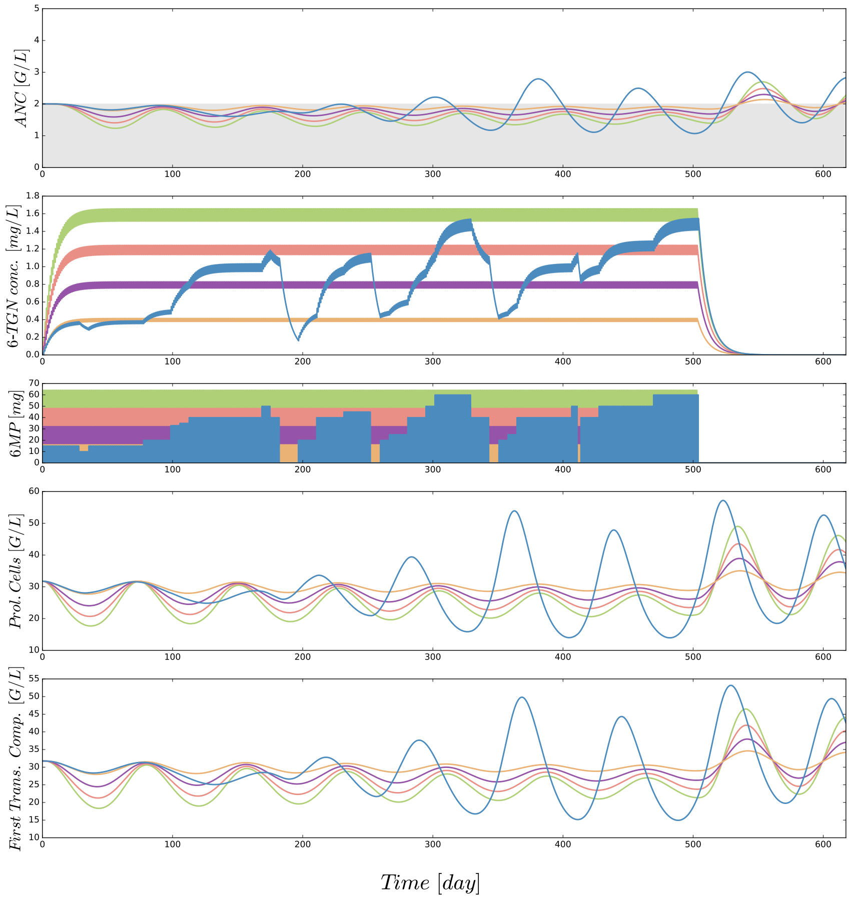

Figure 17: As Figure 2, but for another patient out of 116 patients.

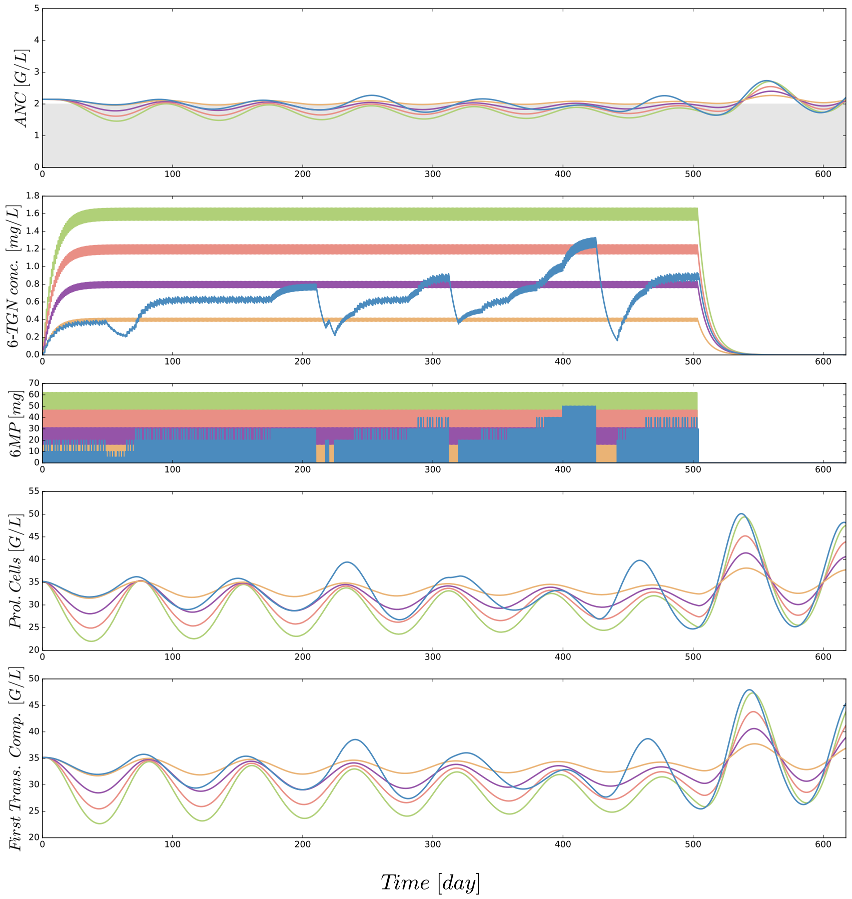

Figure 18: As Figure 2, but for another patient out of 116 patients.

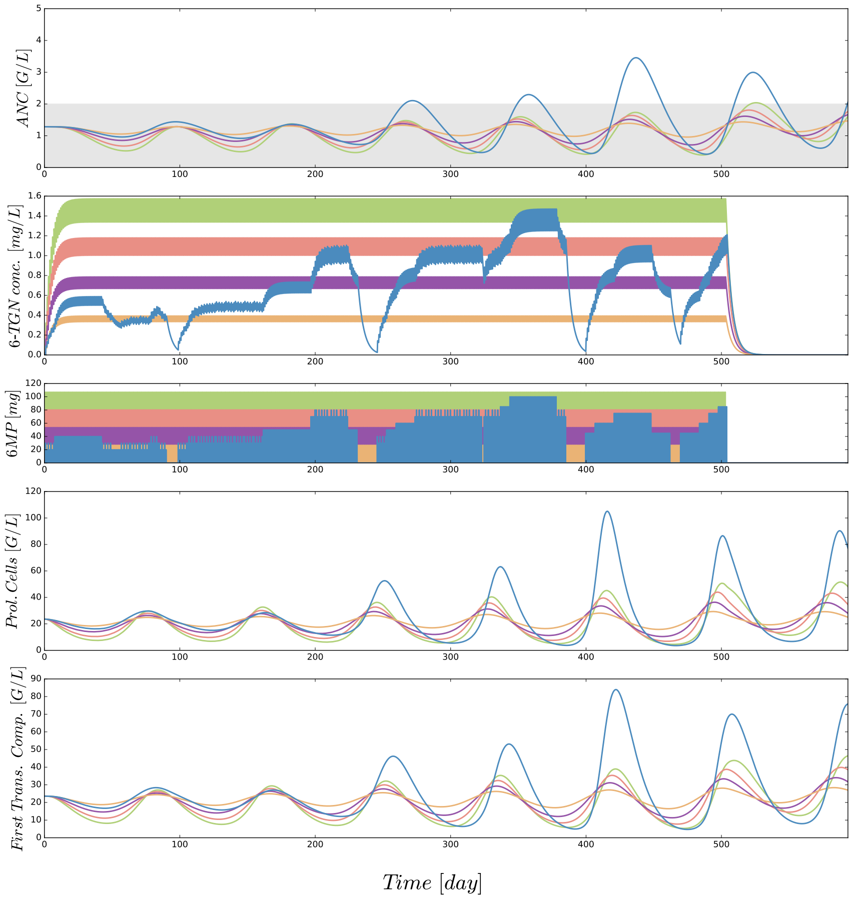

Figure 19: As Figure 2, but for another patient out of 116 patients.

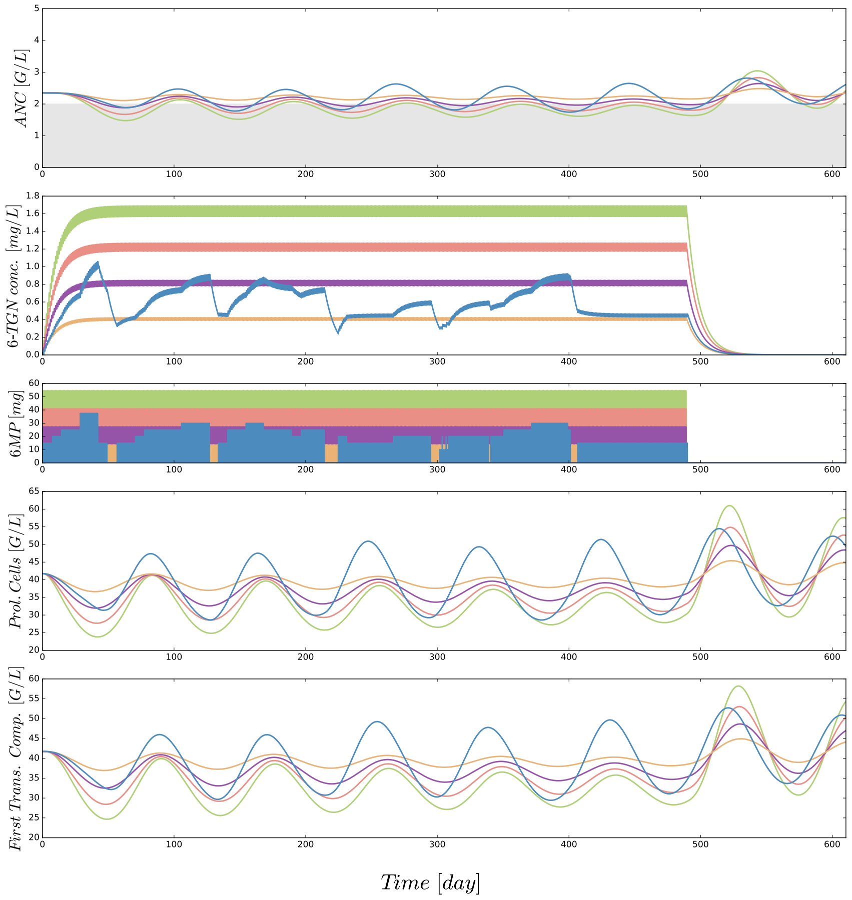

Figure 20: As Figure 2, but for another patient out of 116 patients.

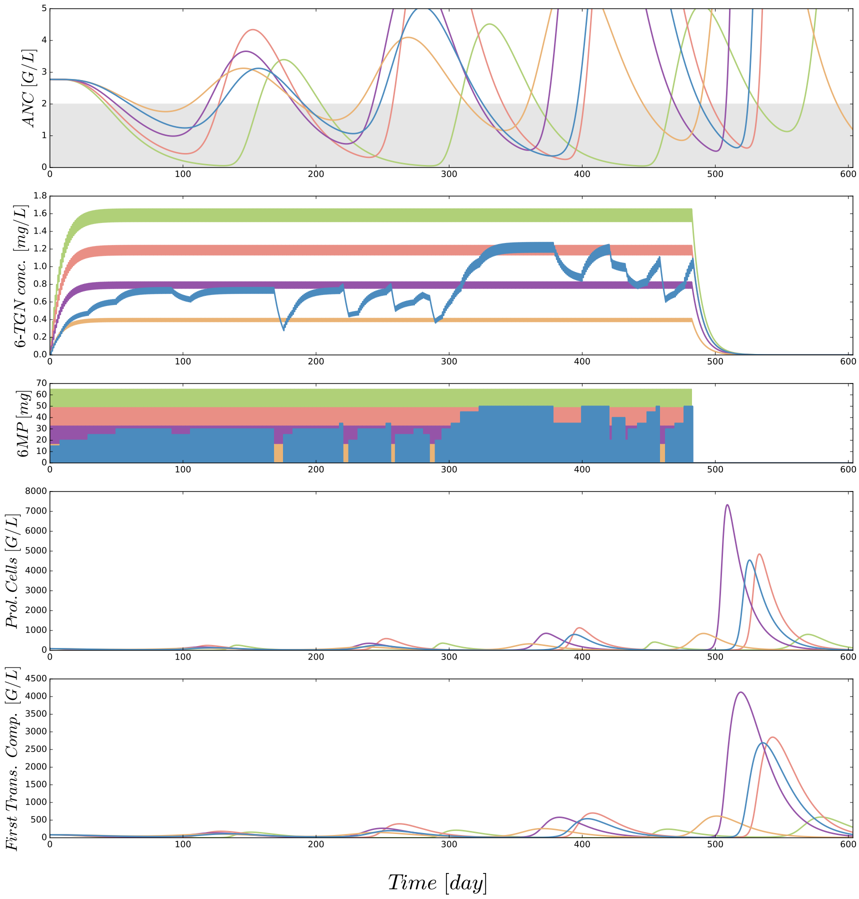

Figure 21: As Figure 2, but for another patient out of 116 patients.

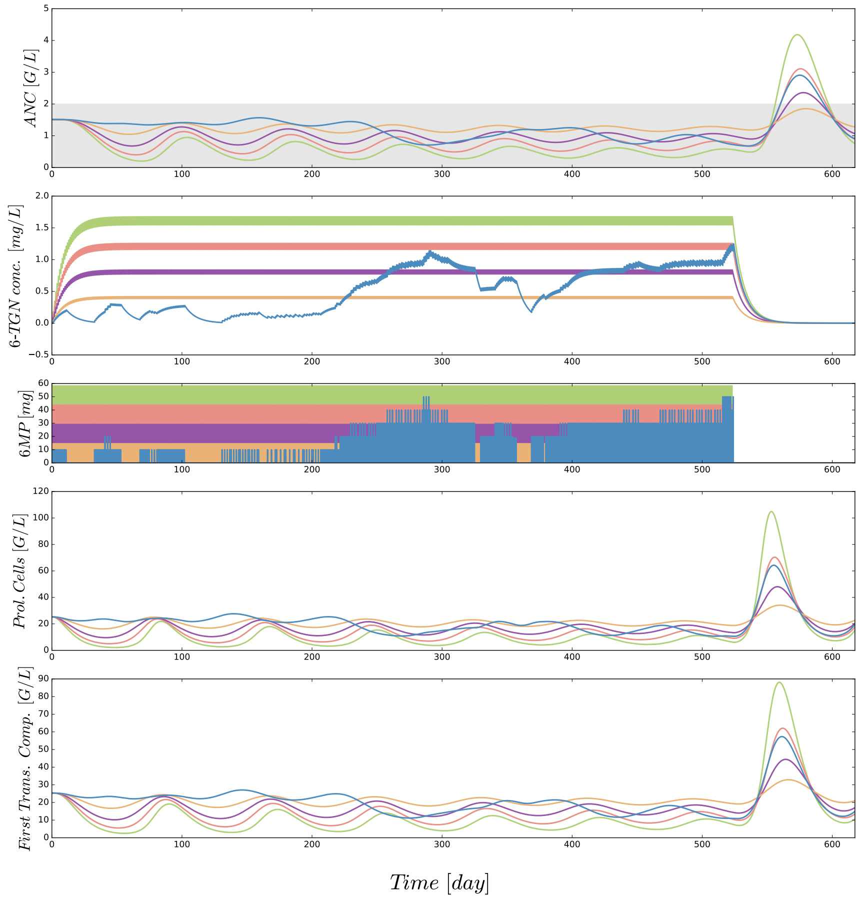

Figure 22: As Figure 2, but for another patient out of 116 patients.

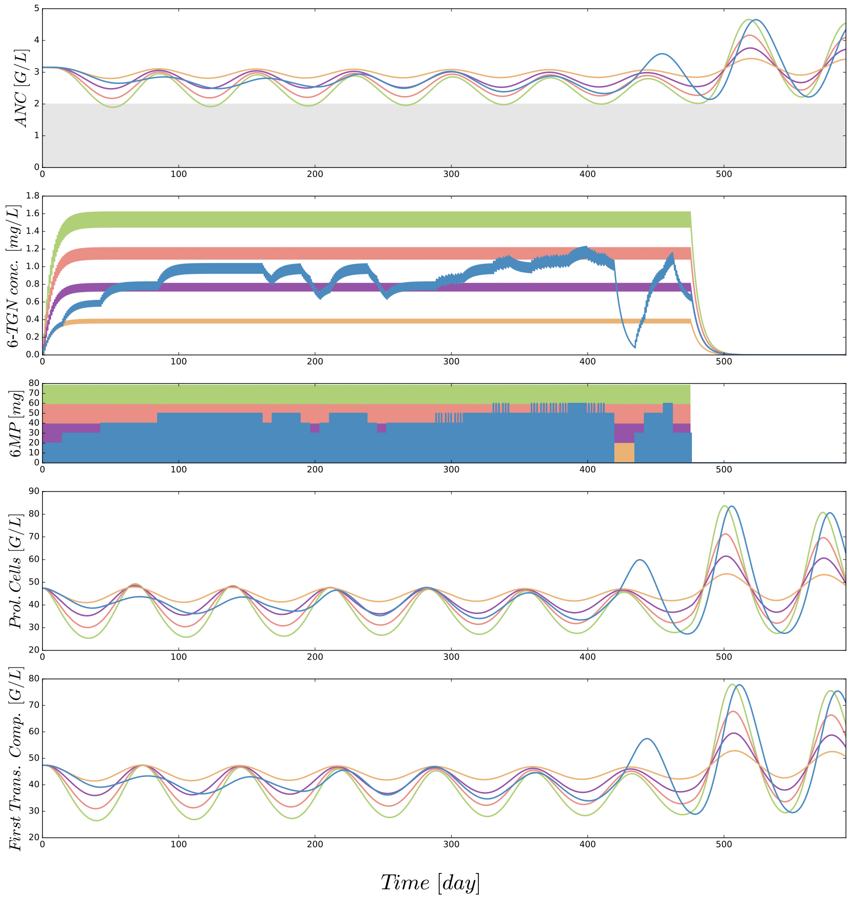

Figure 23: As Figure 2, but for another patient out of 116 patients.

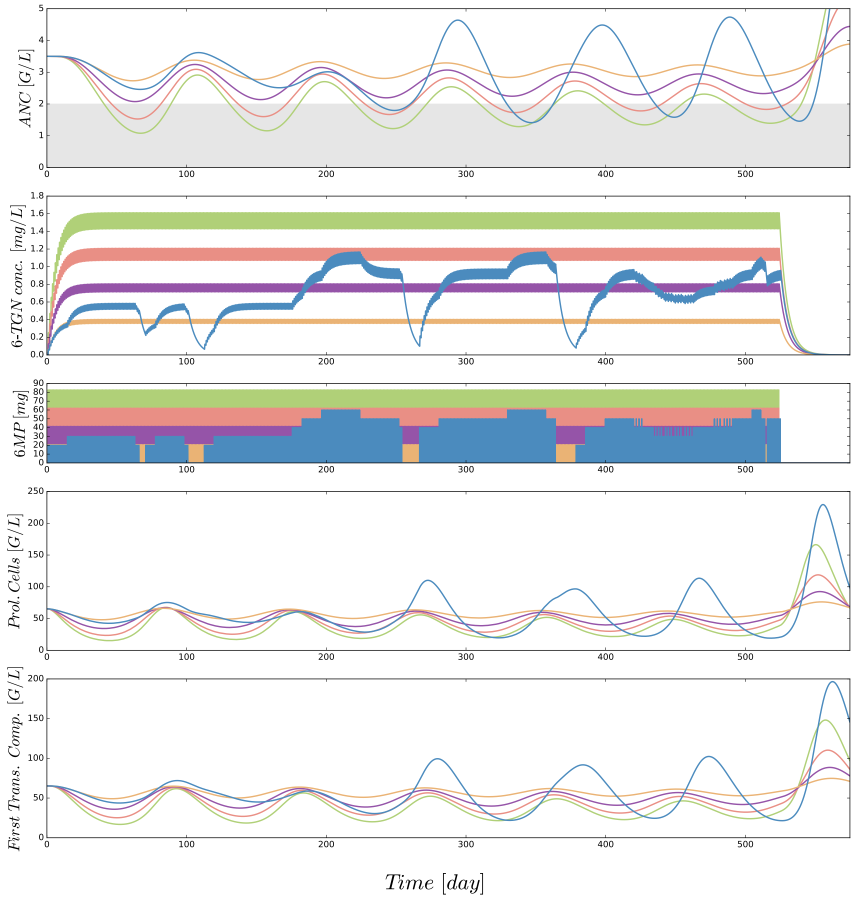

Figure 24: As Figure 2, but for another patient out of 116 patients.

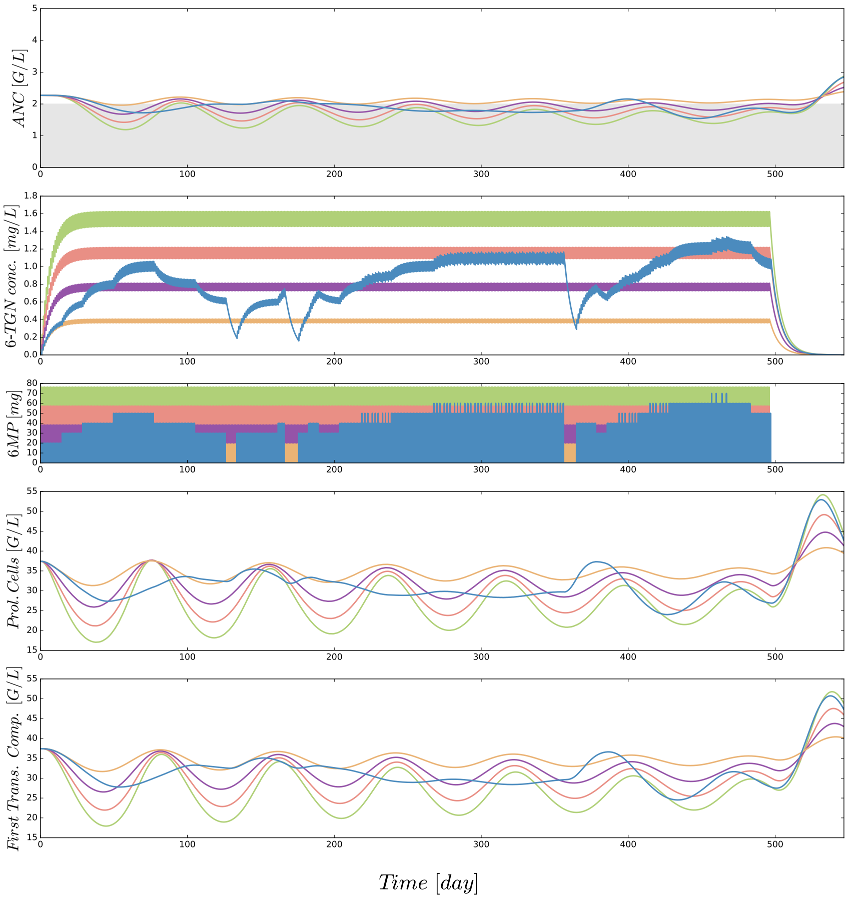

Figure 25: As Figure 2, but for another patient out of 116 patients.

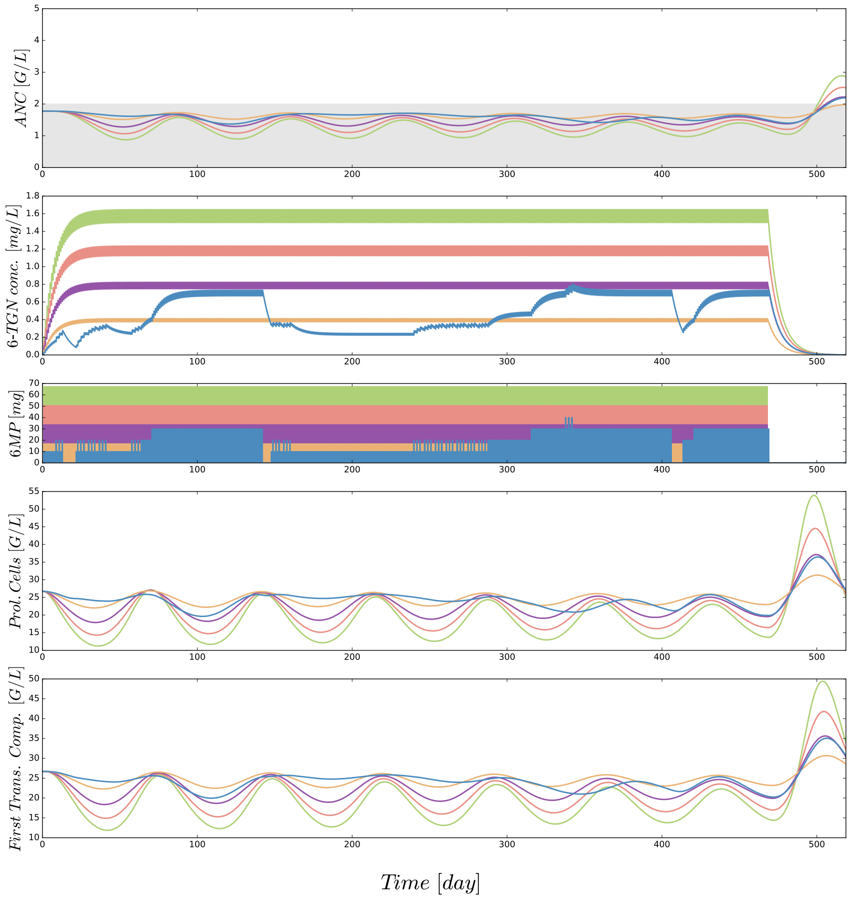

Figure 26: As Figure 2, but for another patient out of 116 patients.

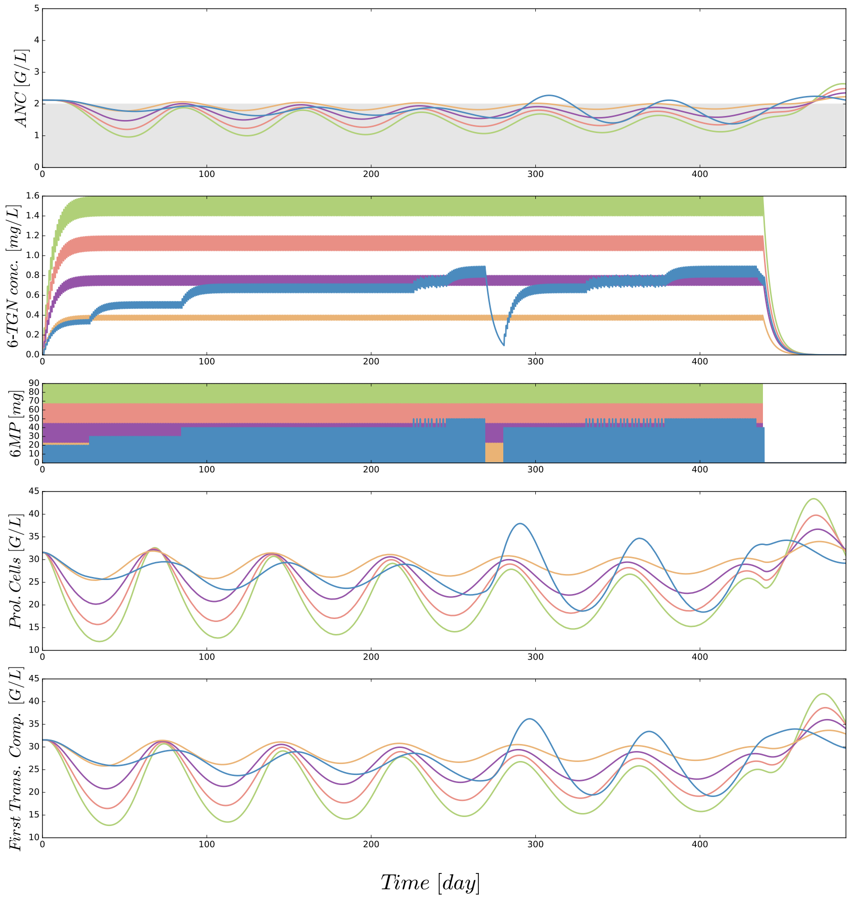

Figure 27: As Figure 2, but for another patient out of 116 patients.

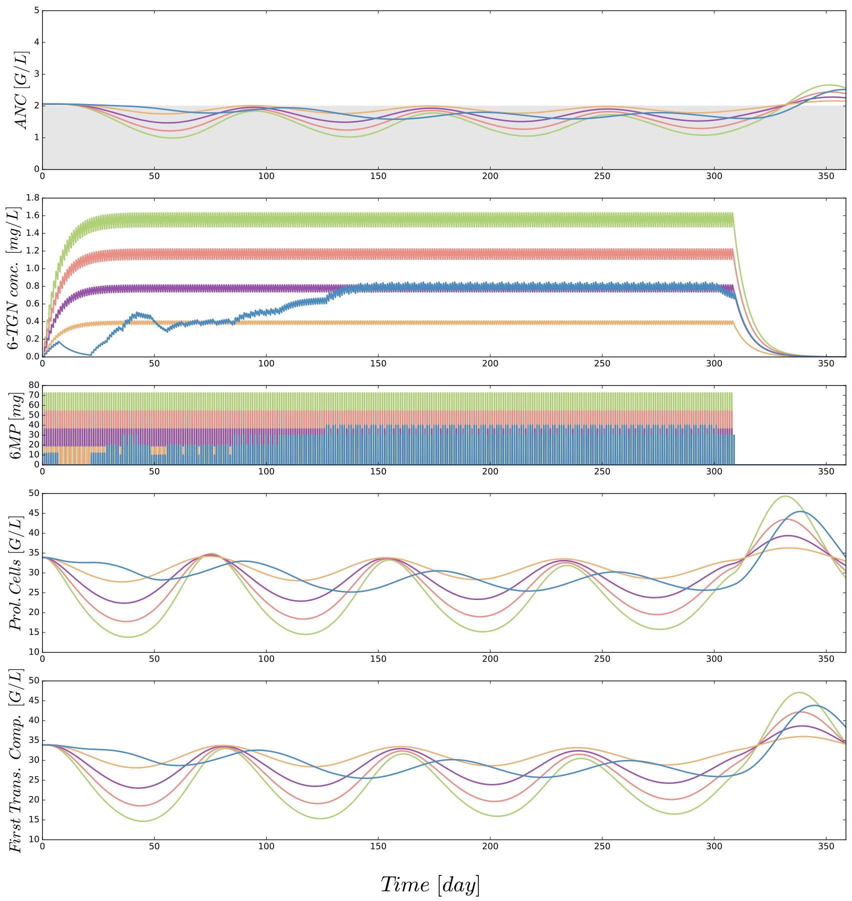

Figure 28: As Figure 2, but for another patient out of 116 patients.

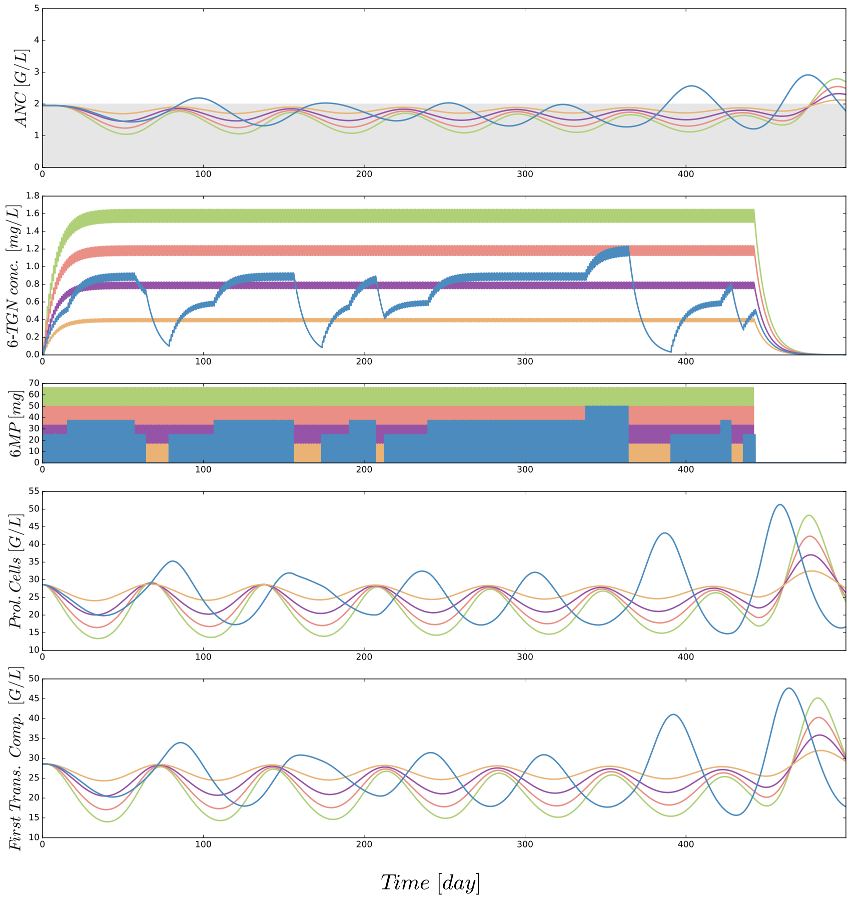

Figure 29: As Figure 2, but for another patient out of 116 patients.

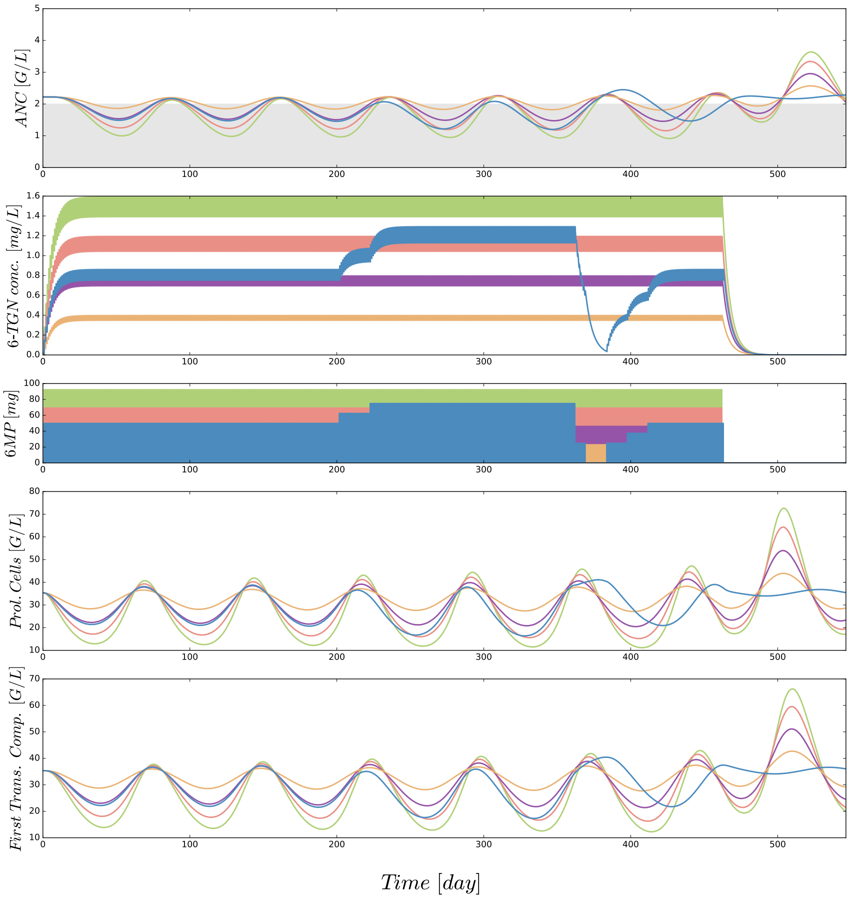

Figure 30: As Figure 2, but for another patient out of 116 patients.

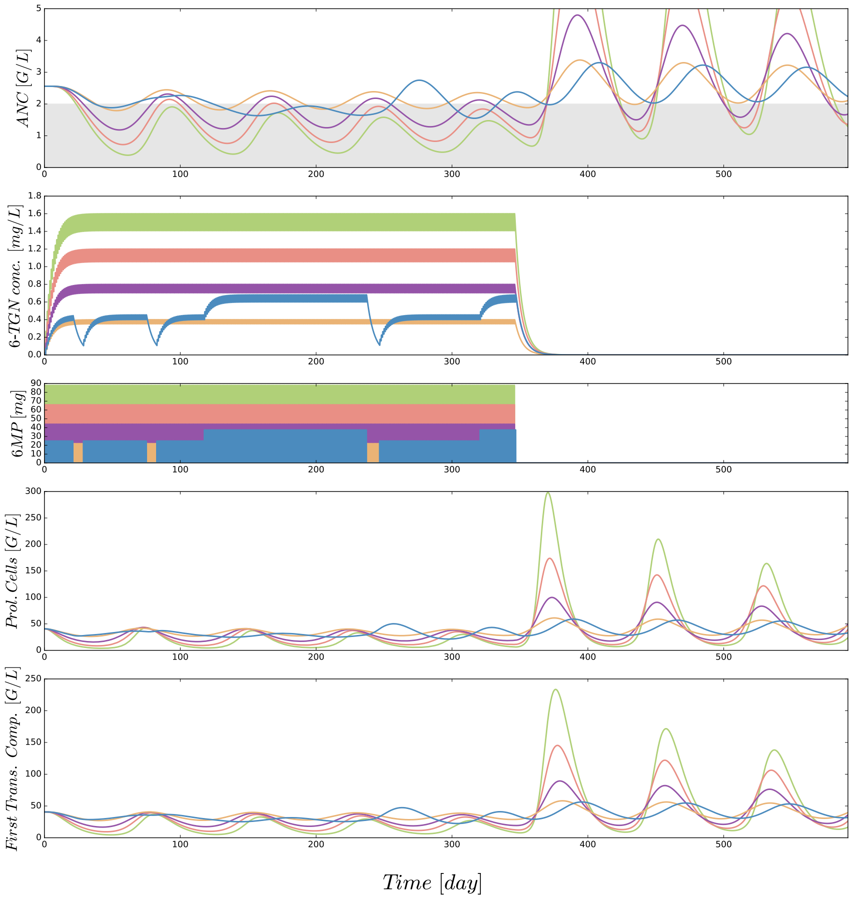

Figure 31: As Figure 2, but for another patient out of 116 patients.

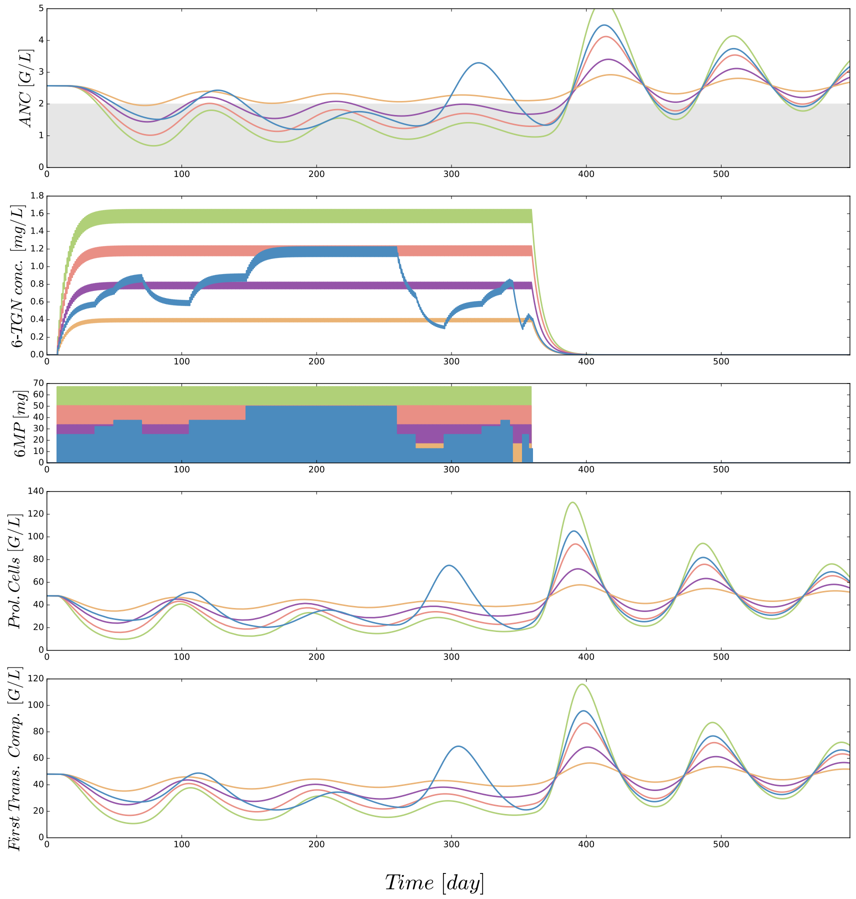

Figure 32: As Figure 2, but for another patient out of 116 patients.

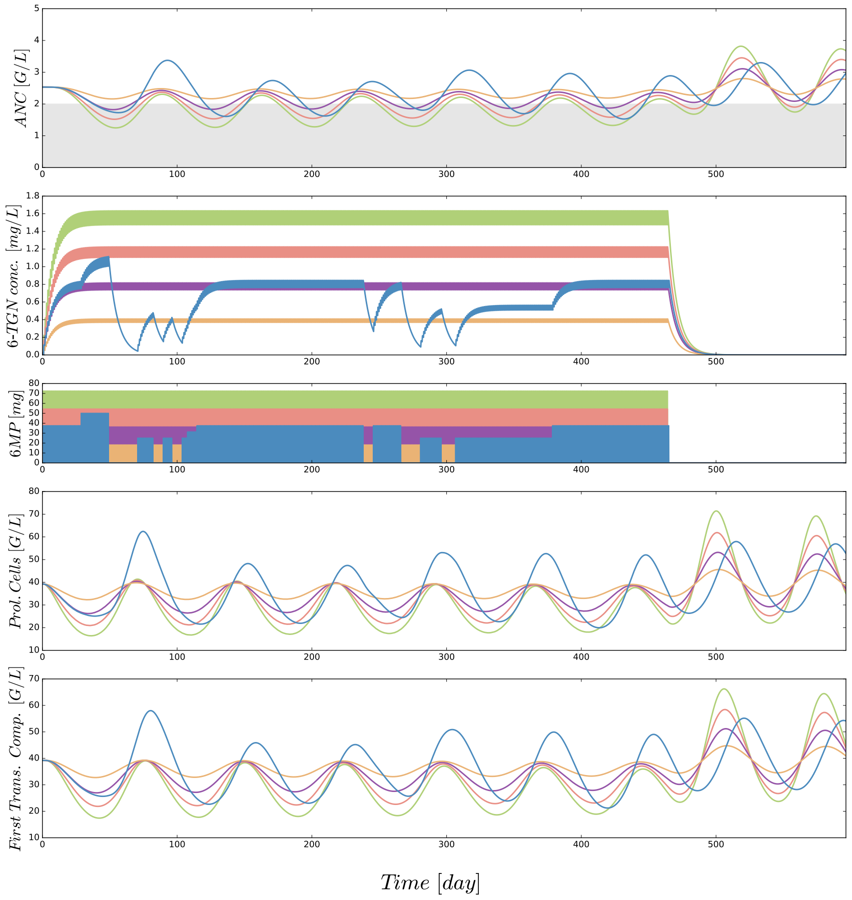

Figure 33: As Figure 2, but for another patient out of 116 patients.

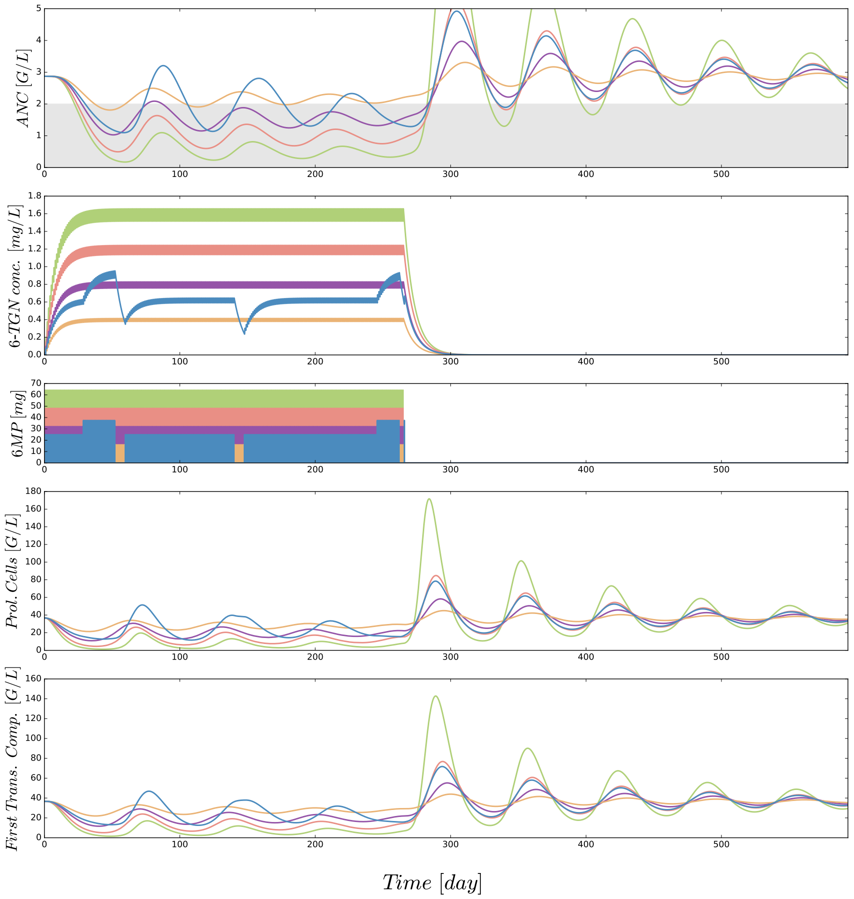

Figure 34: As Figure 2, but for another patient out of 116 patients.

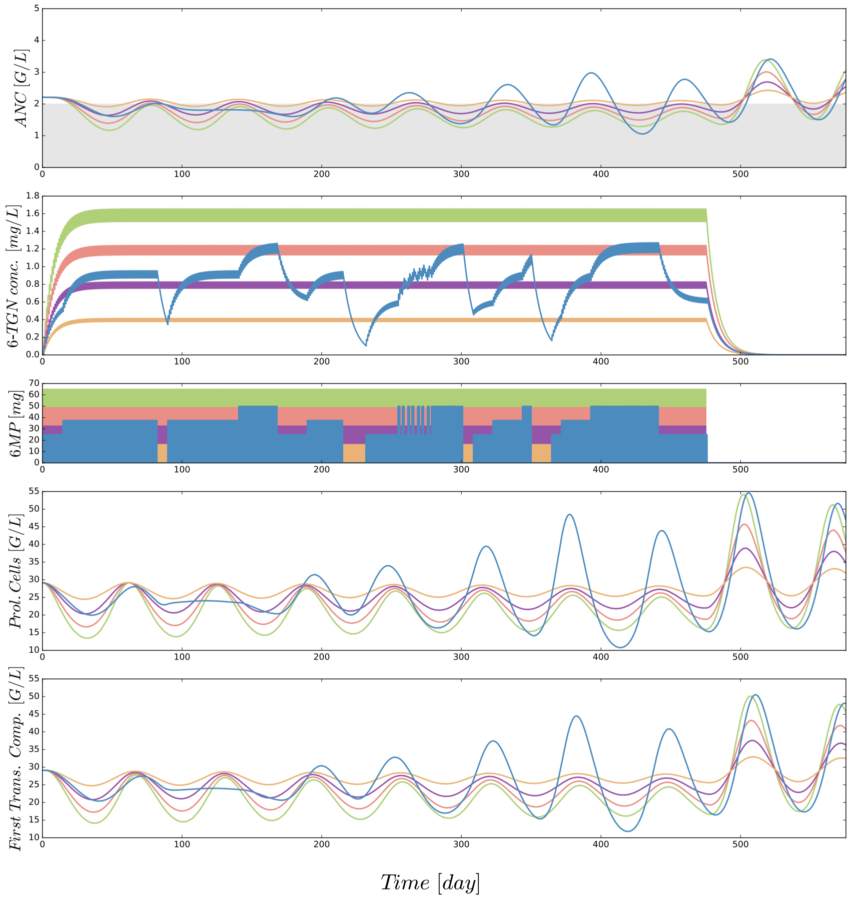

Figure 35: As Figure 2, but for another patient out of 116 patients.

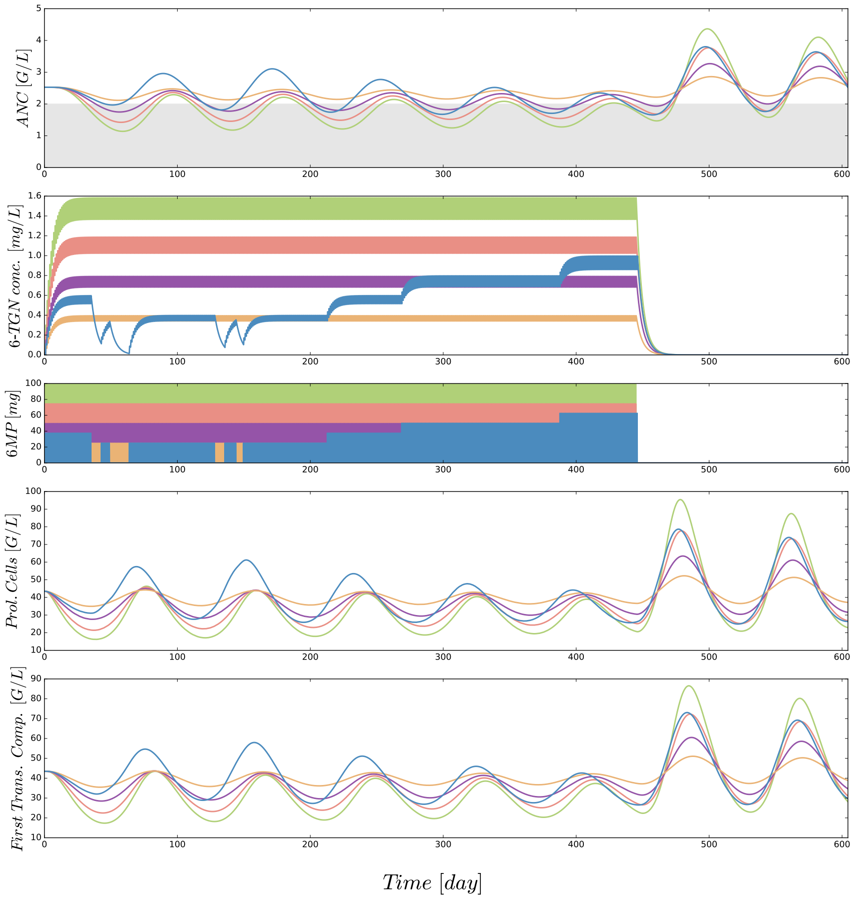

Figure 36: As Figure 2, but for another patient out of 116 patients.

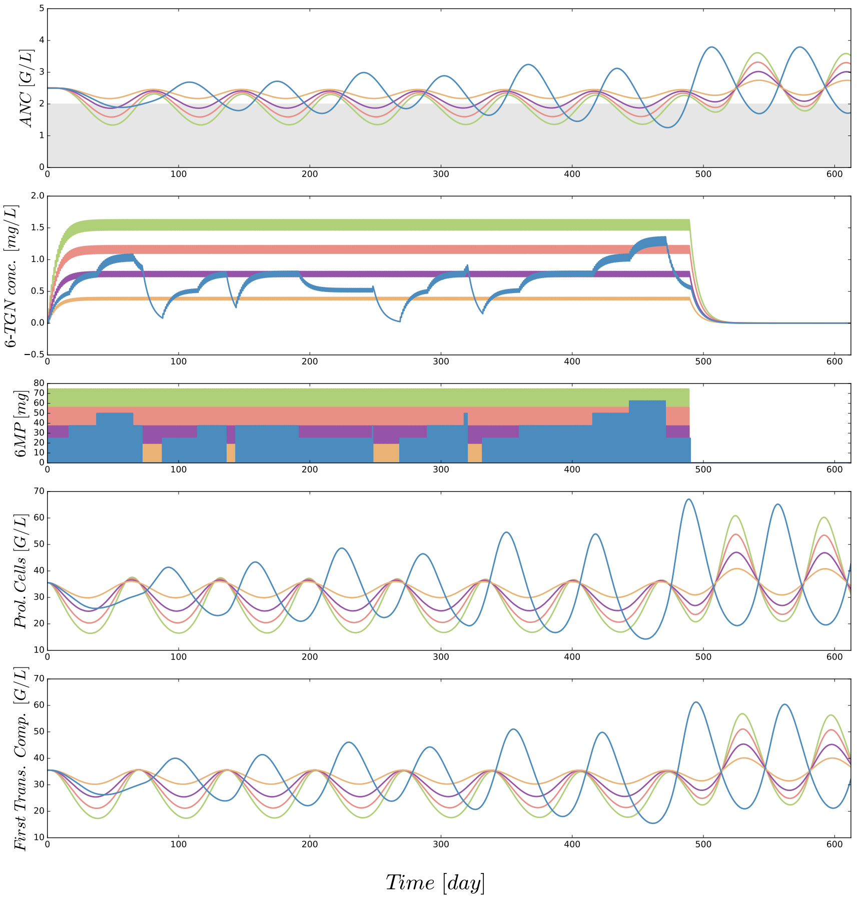

Figure 37: As Figure 2, but for another patient out of 116 patients.

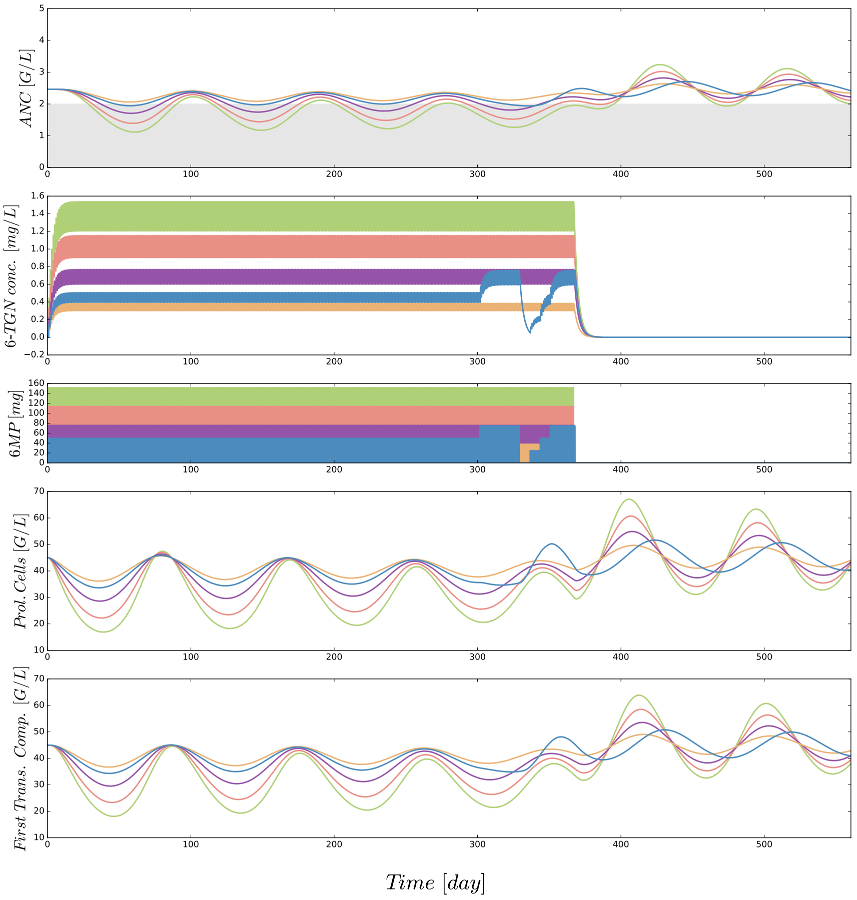

Figure 38: As Figure 2, but for another patient out of 116 patients.

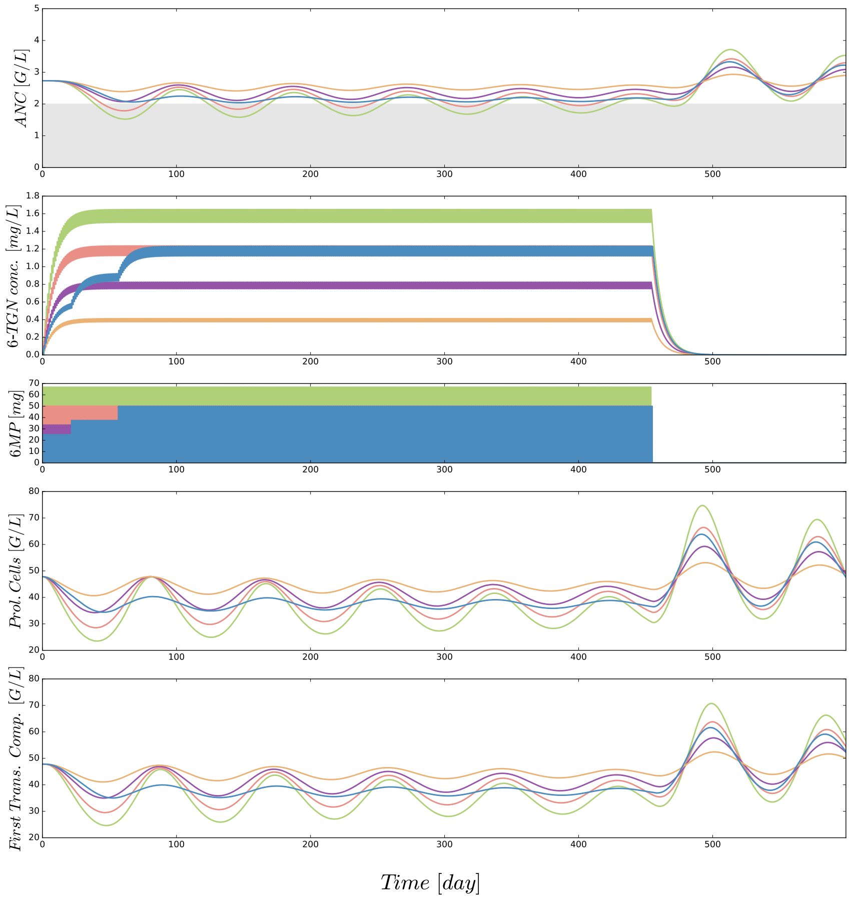

Figure 39: As Figure 2, but for another patient out of 116 patients.

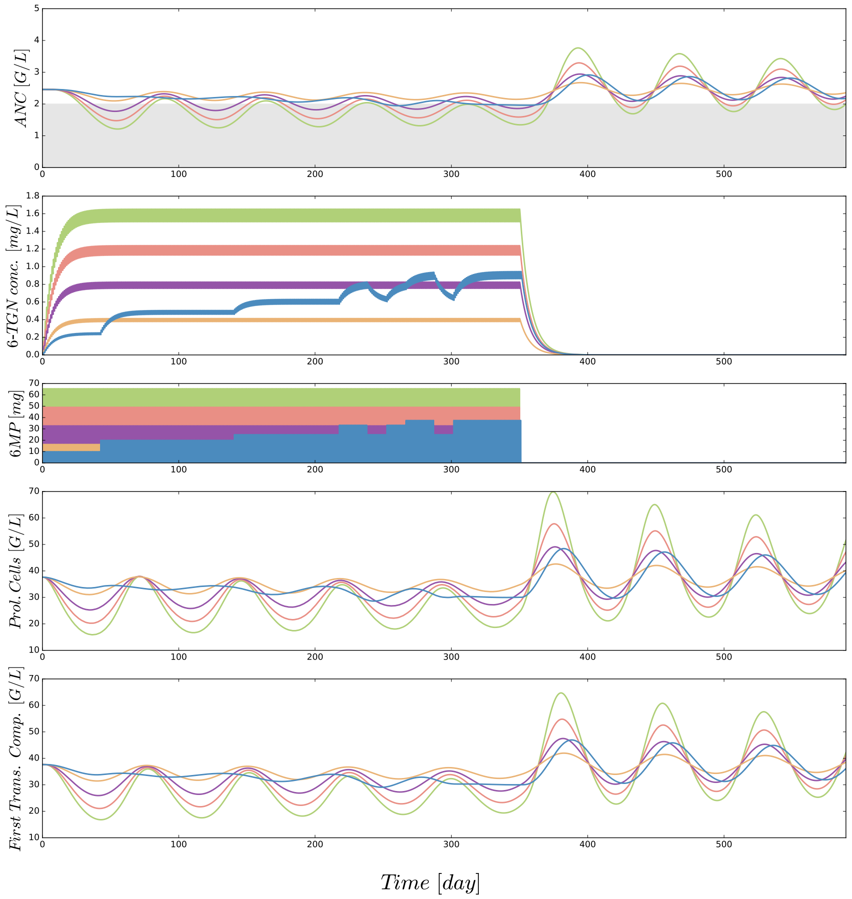

Figure 40: As Figure 2, but for another patient out of 116 patients.

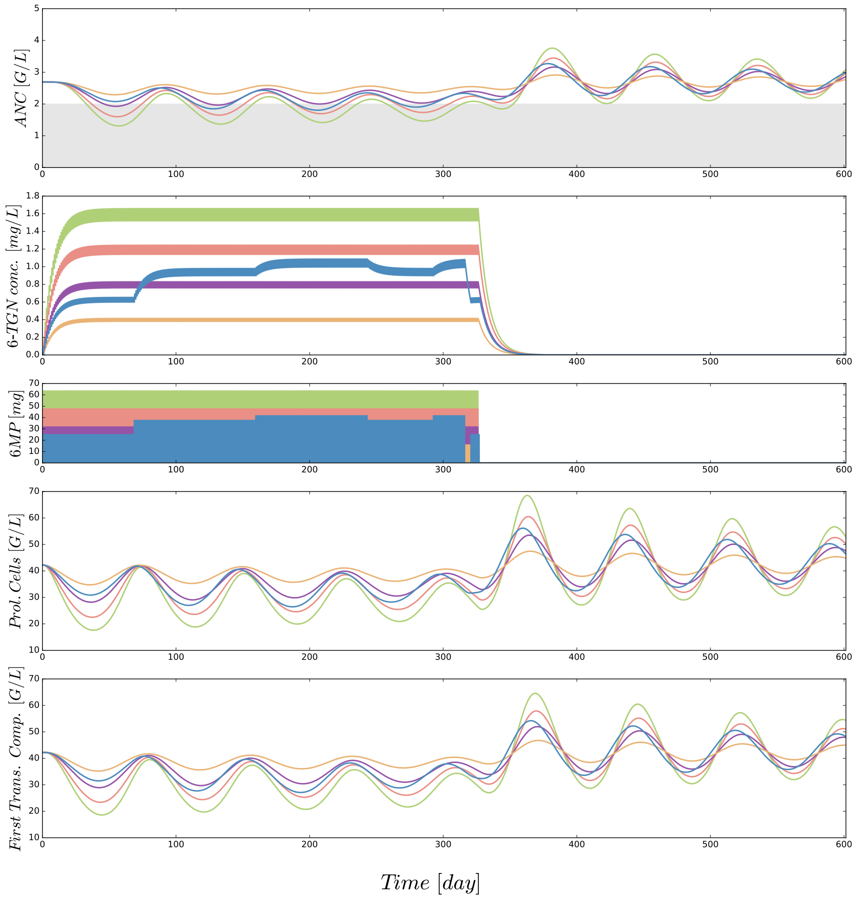

Figure 41: As Figure 2, but for another patient out of 116 patients.

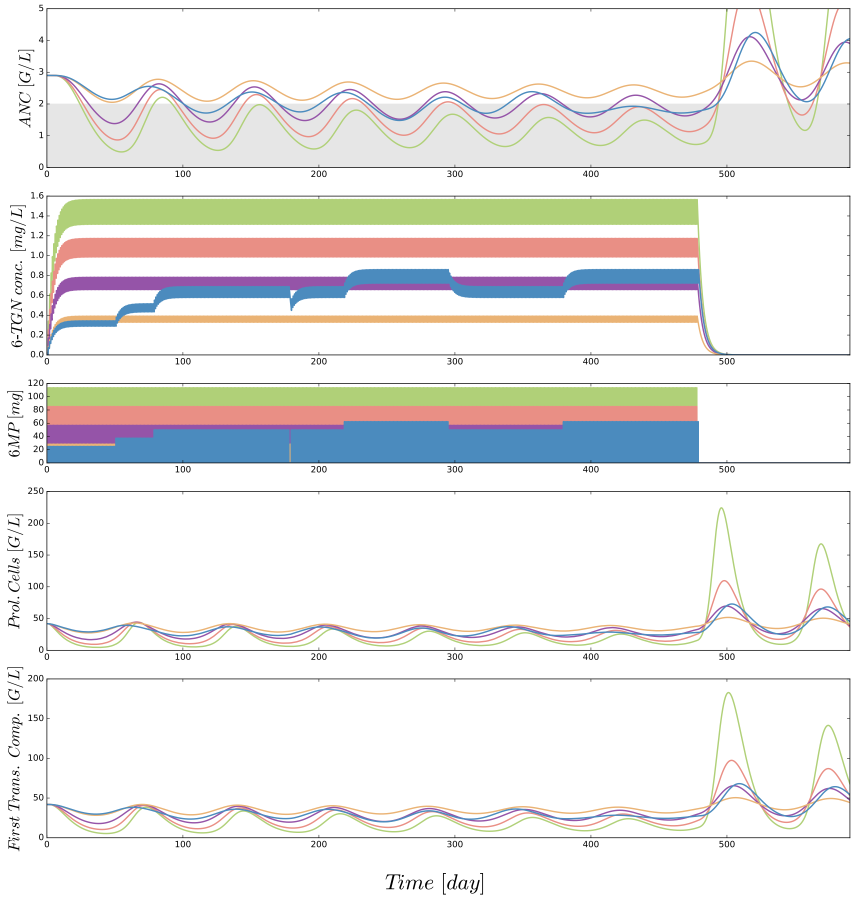

Figure 42: As Figure 2, but for another patient out of 116 patients.

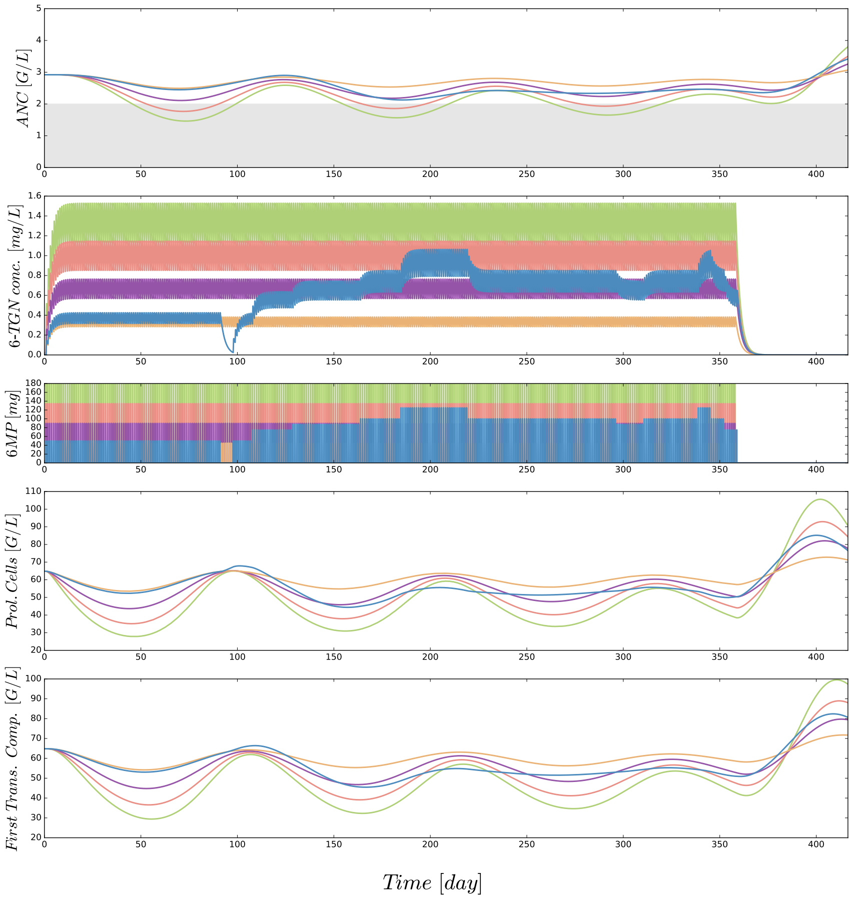

Figure 43: As Figure 2, but for another patient out of 116 patients.

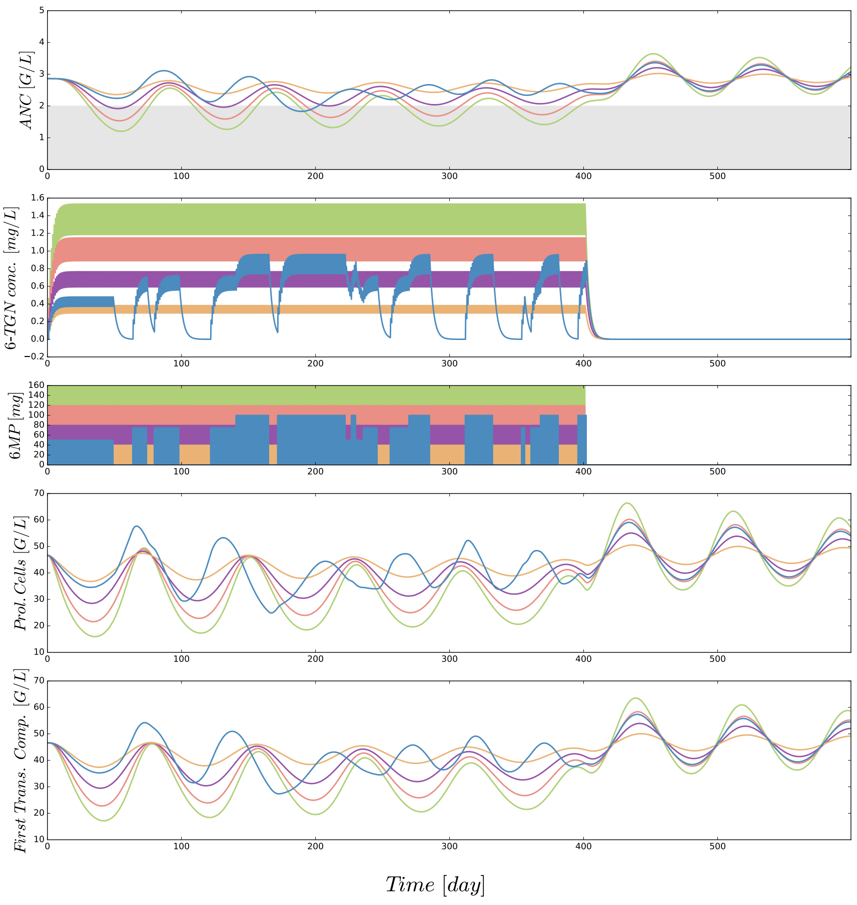

Figure 44: As Figure 2, but for another patient out of 116 patients.

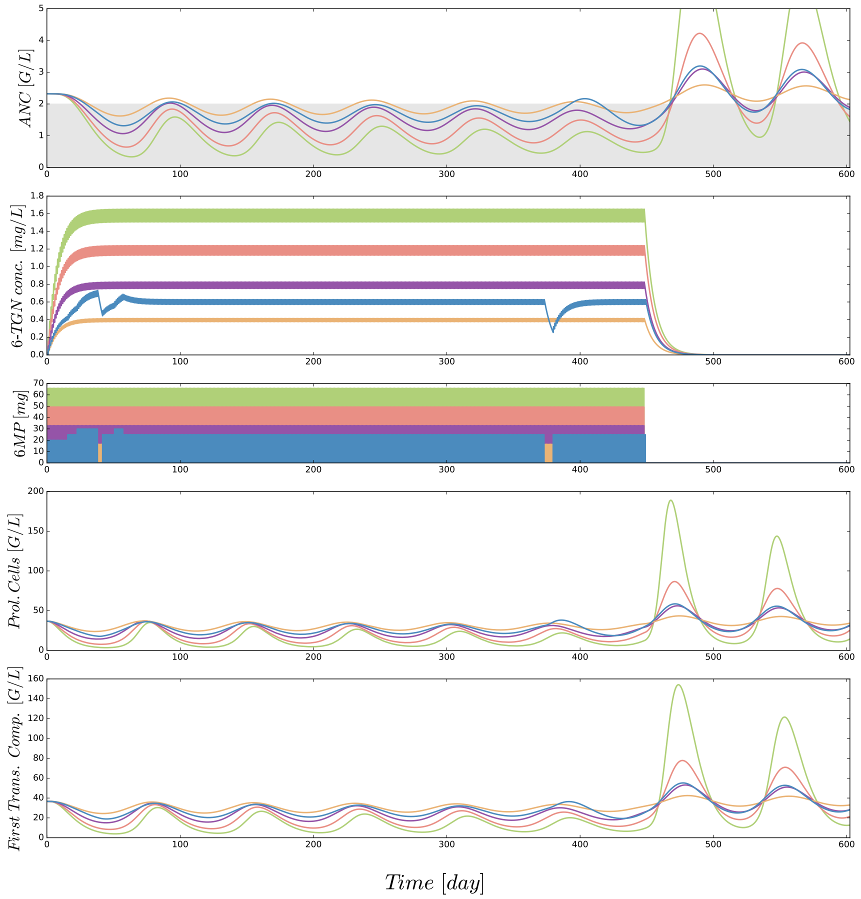

Figure 45: As Figure 2, but for another patient out of 116 patients.

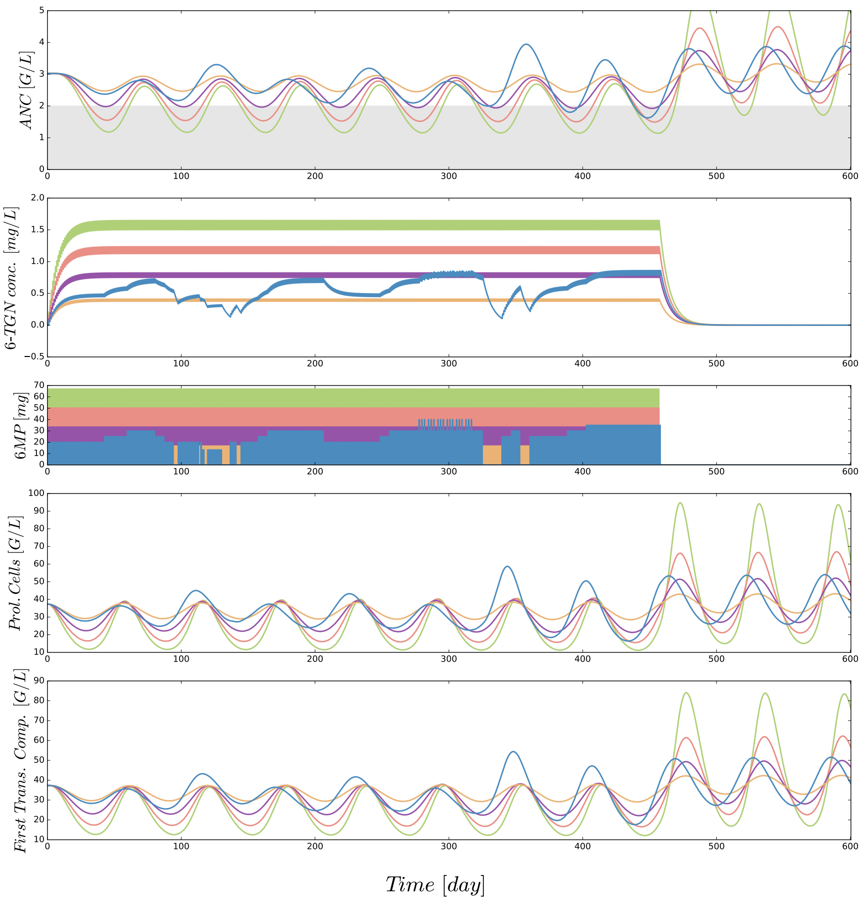

Figure 46: As Figure 2, but for another patient out of 116 patients.

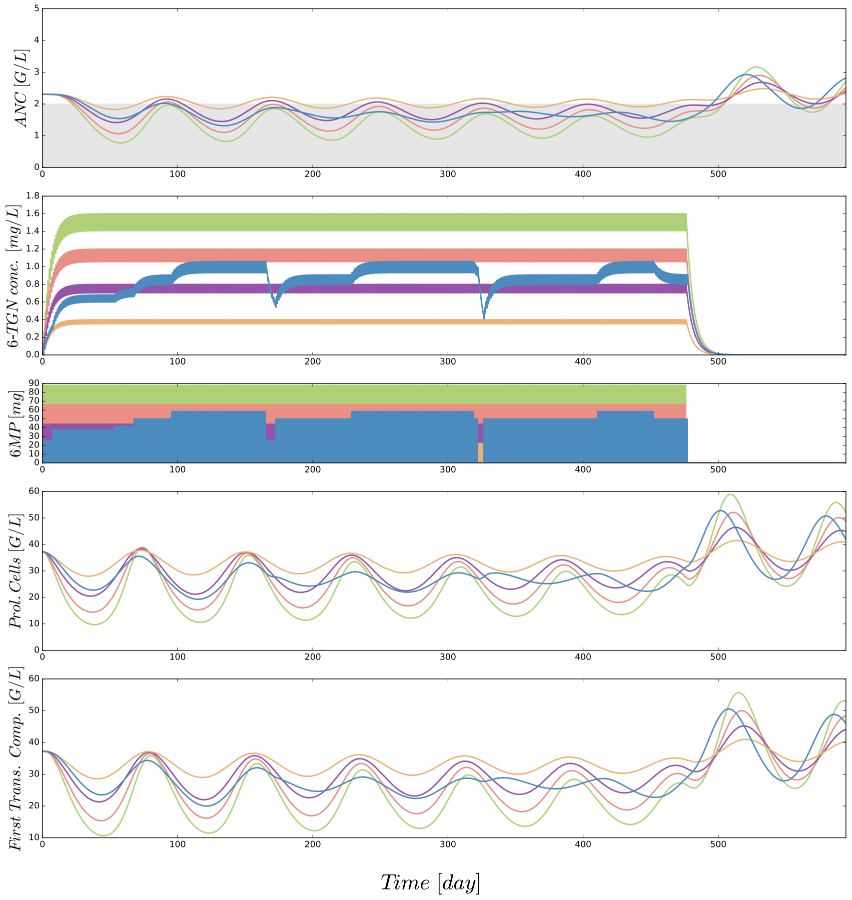

Figure 47: As Figure 2, but for another patient out of 116 patients.

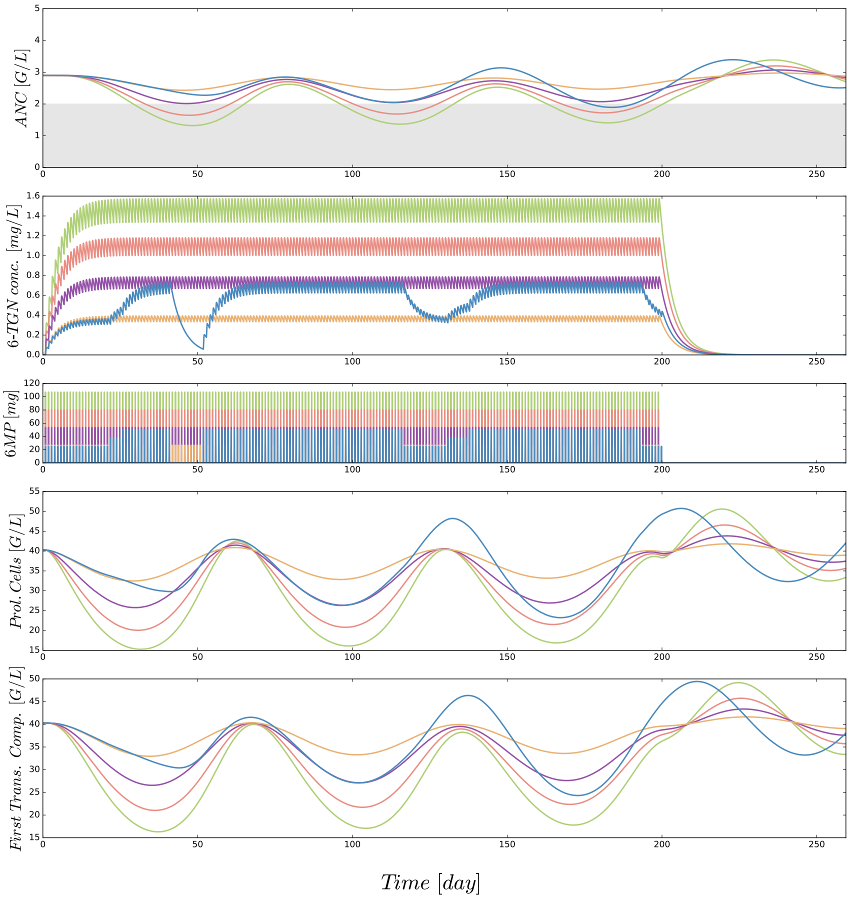

Figure 48: As Figure 2, but for another patient out of 116 patients.

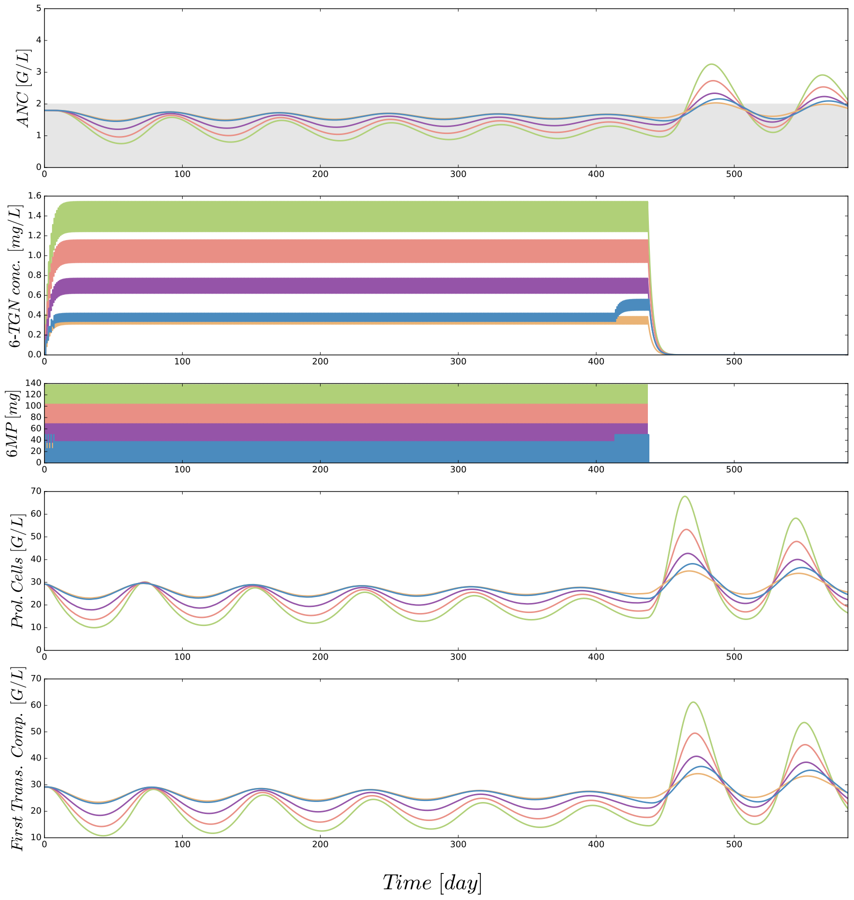

Figure 49: As Figure 2, but for another patient out of 116 patients.

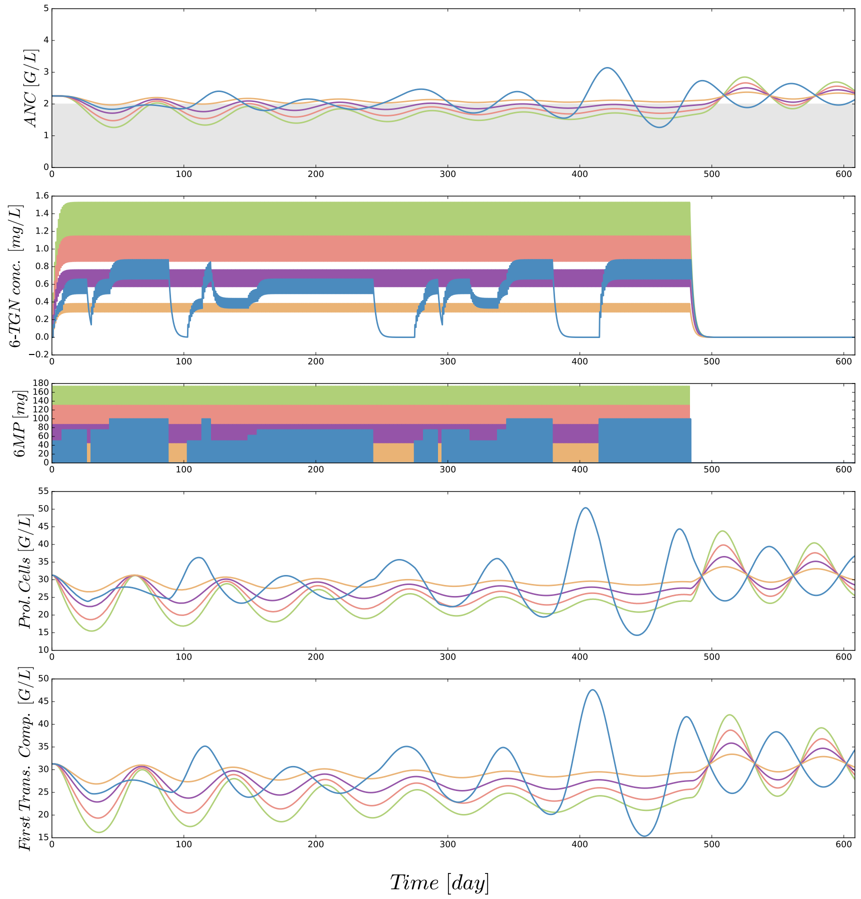

Figure 50: As Figure 2, but for another patient out of 116 patients.

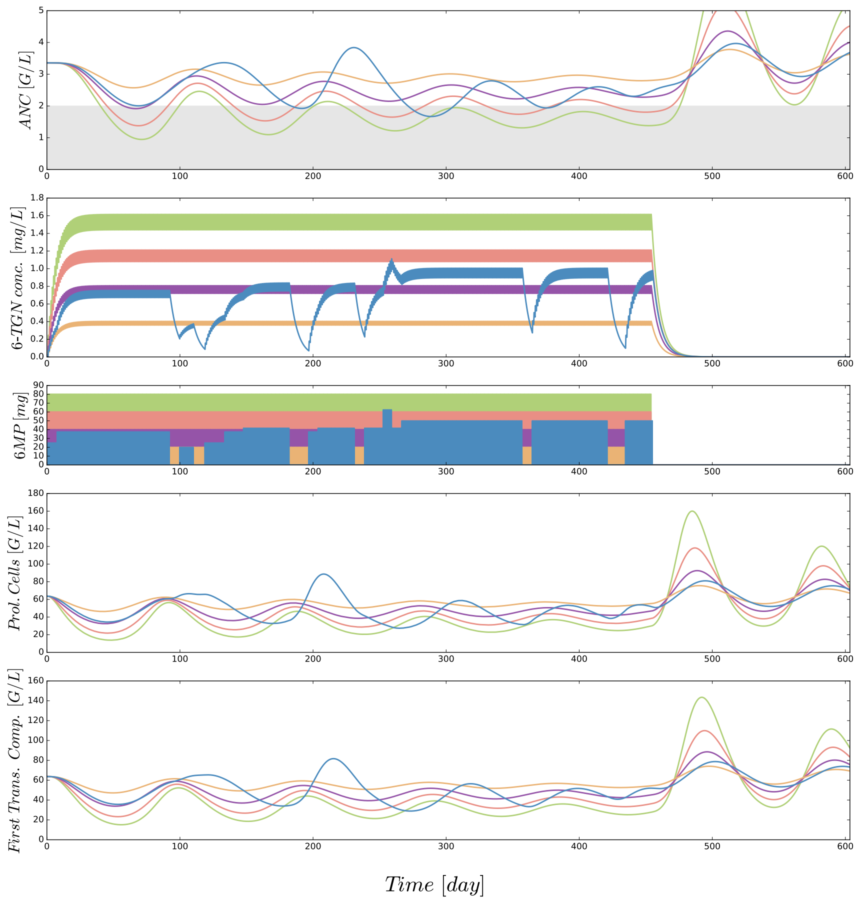

Figure 51: As Figure 2, but for another patient out of 116 patients.

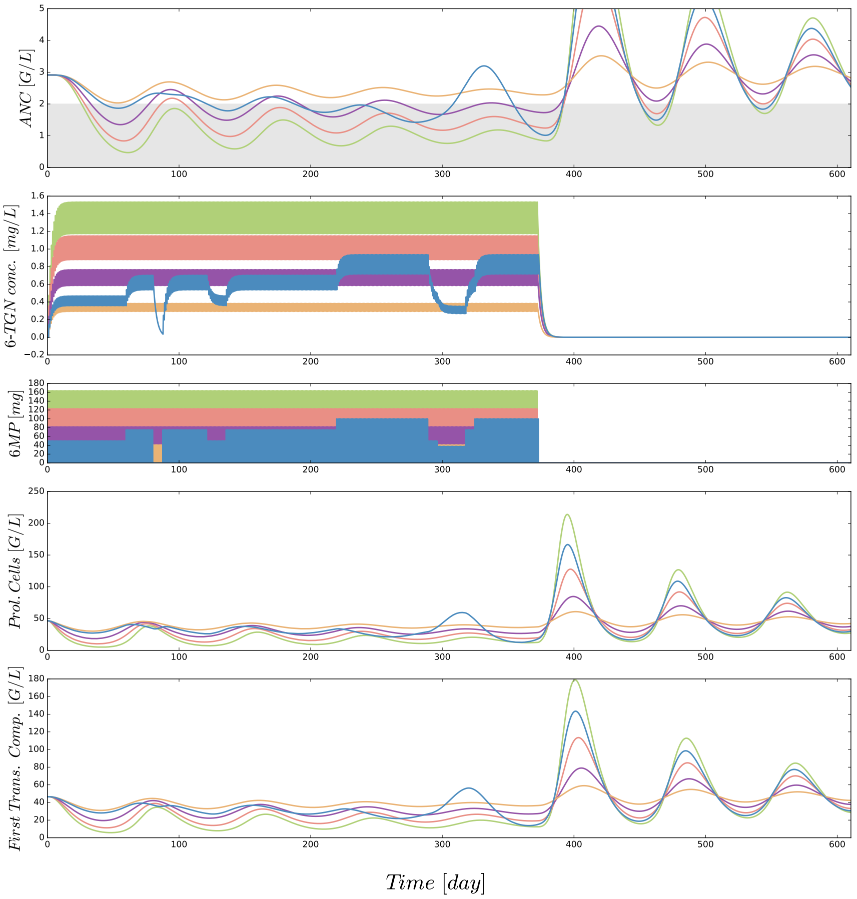

Figure 52: As Figure 2, but for another patient out of 116 patients.

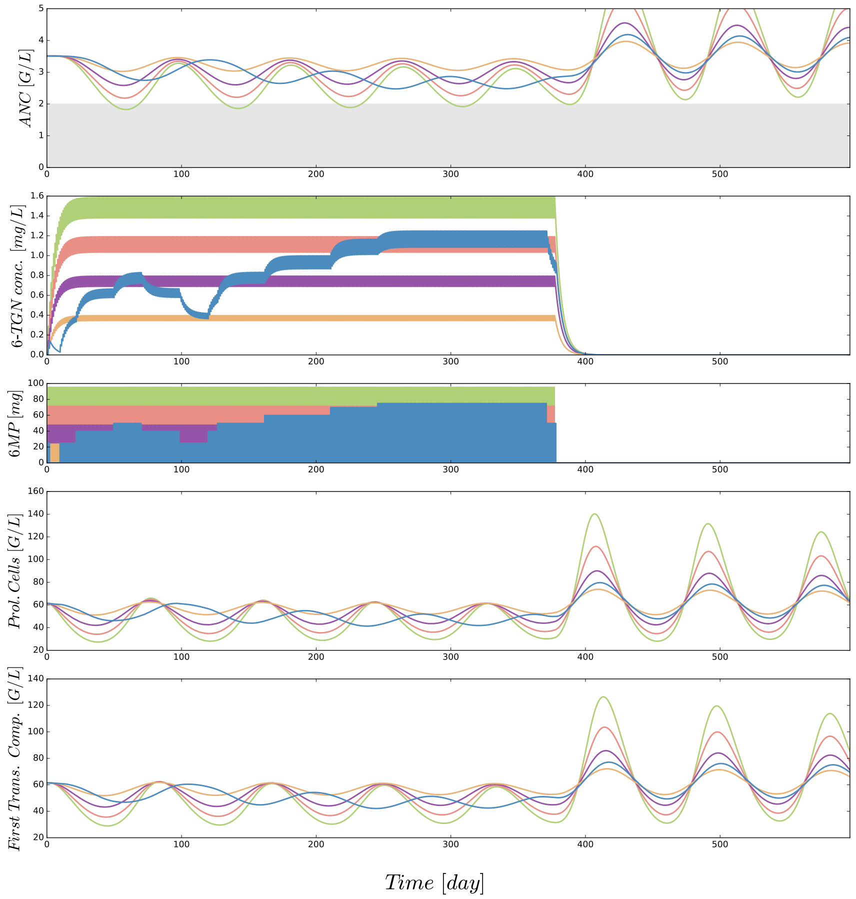

Figure 53: As Figure 2, but for another patient out of 116 patients.

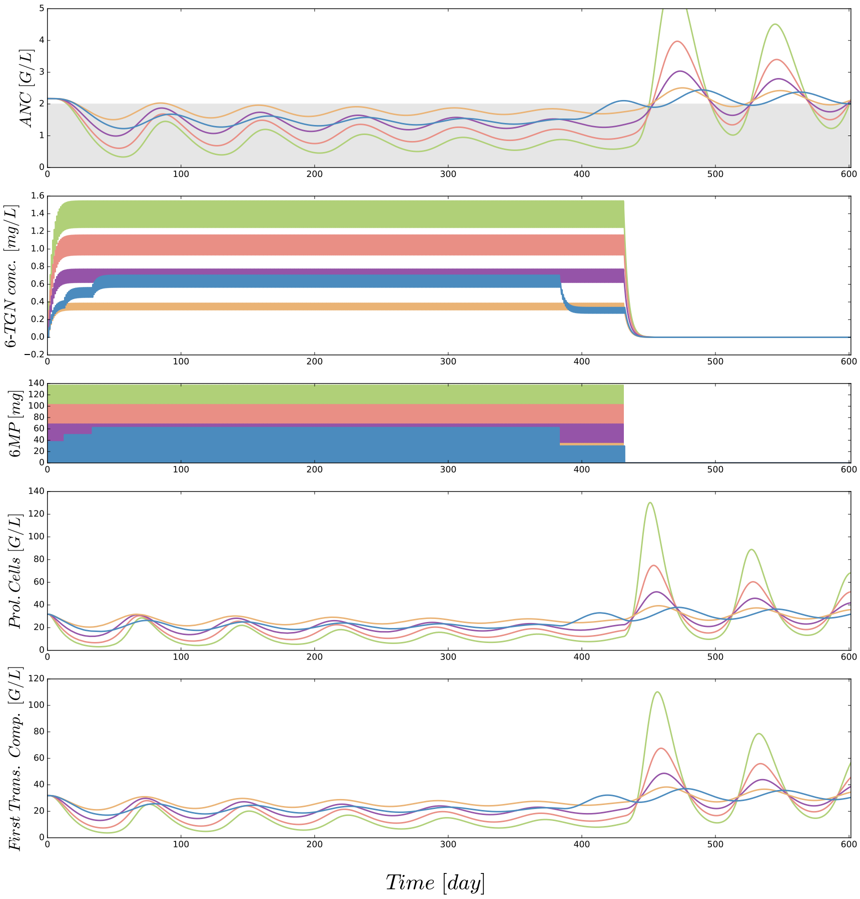

Figure 54: As Figure 2, but for another patient out of 116 patients.

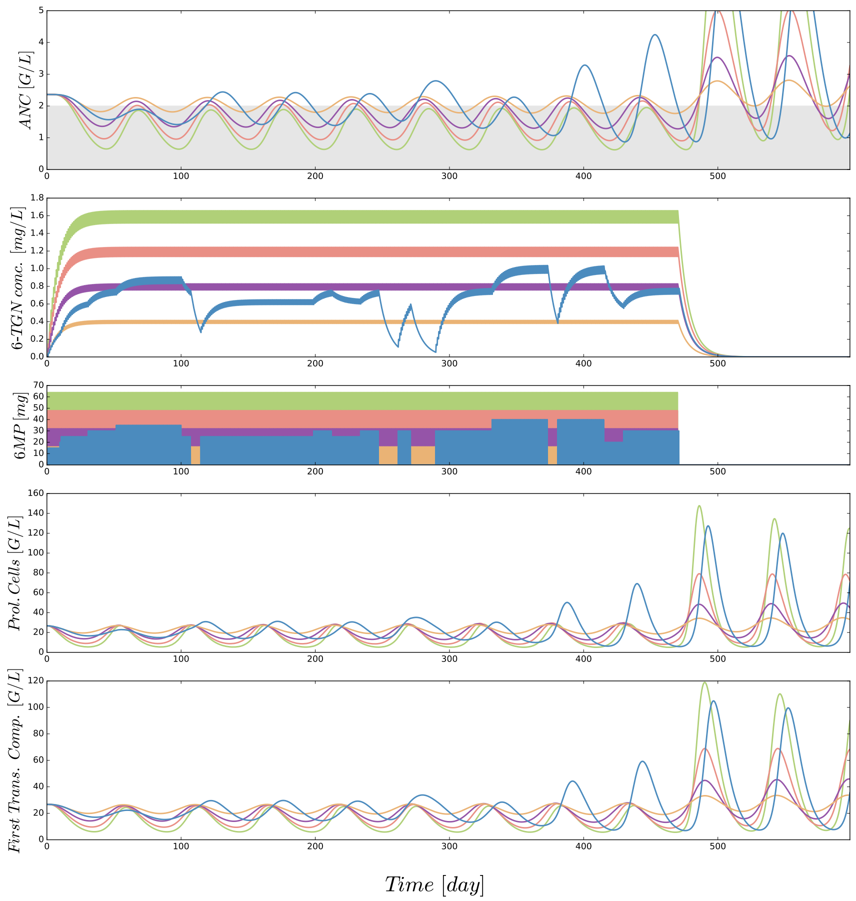

Figure 55: As Figure 2, but for another patient out of 116 patients.

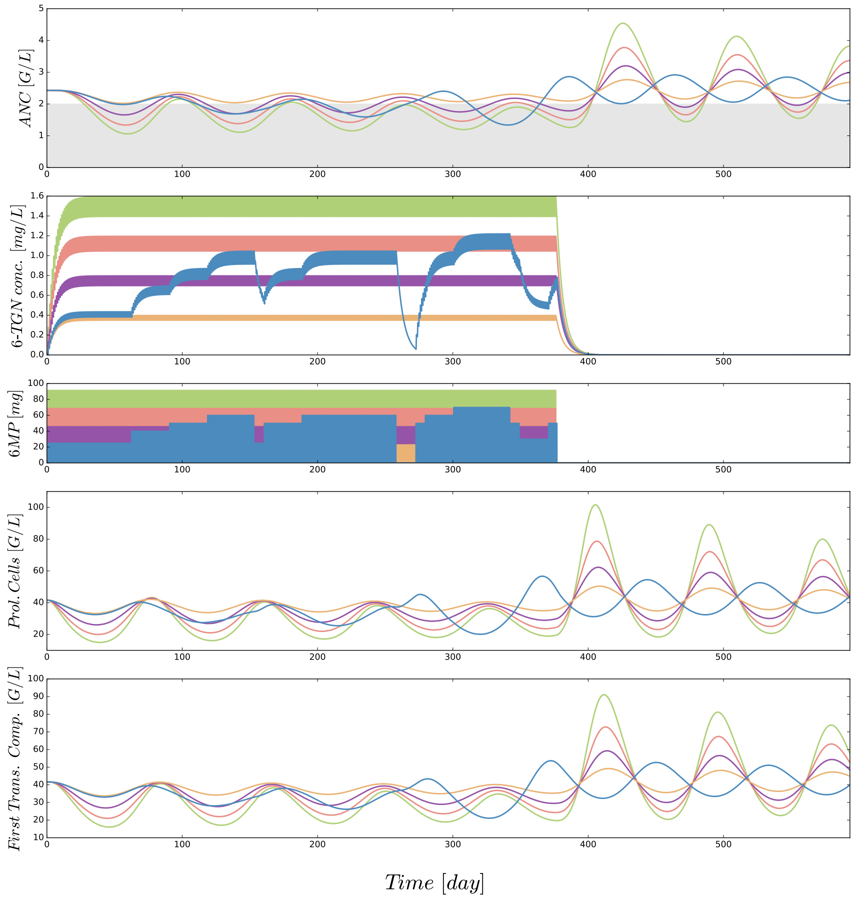

Figure 56: As Figure 2, but for another patient out of 116 patients.

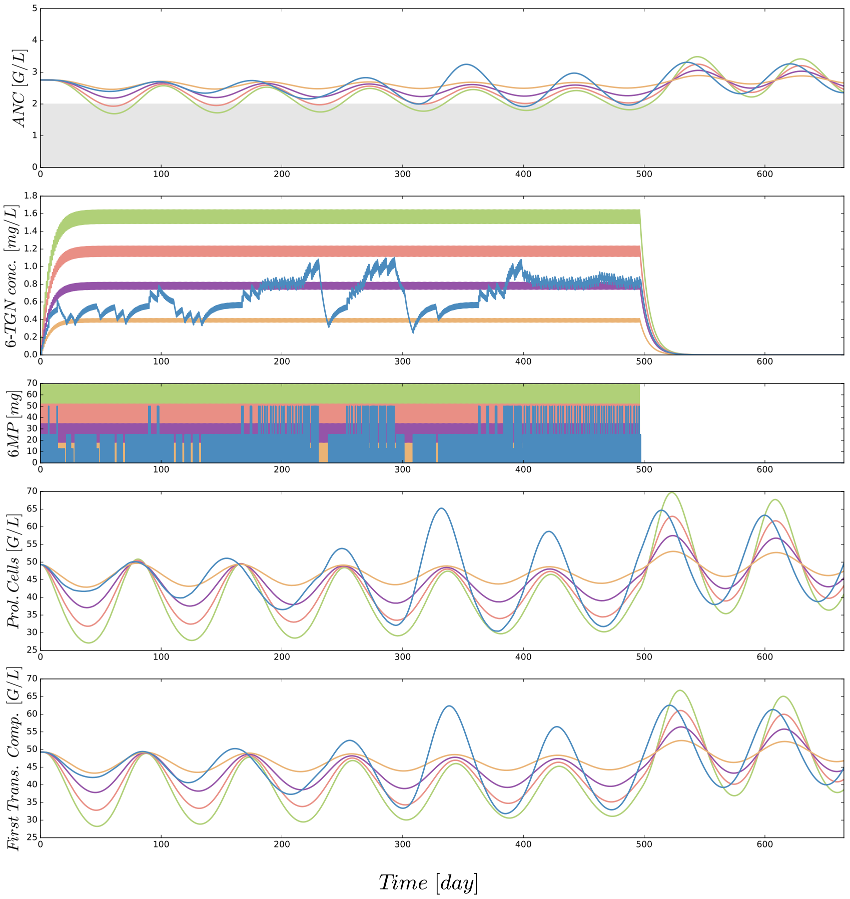

Figure 57: As Figure 2, but for another patient out of 116 patients.

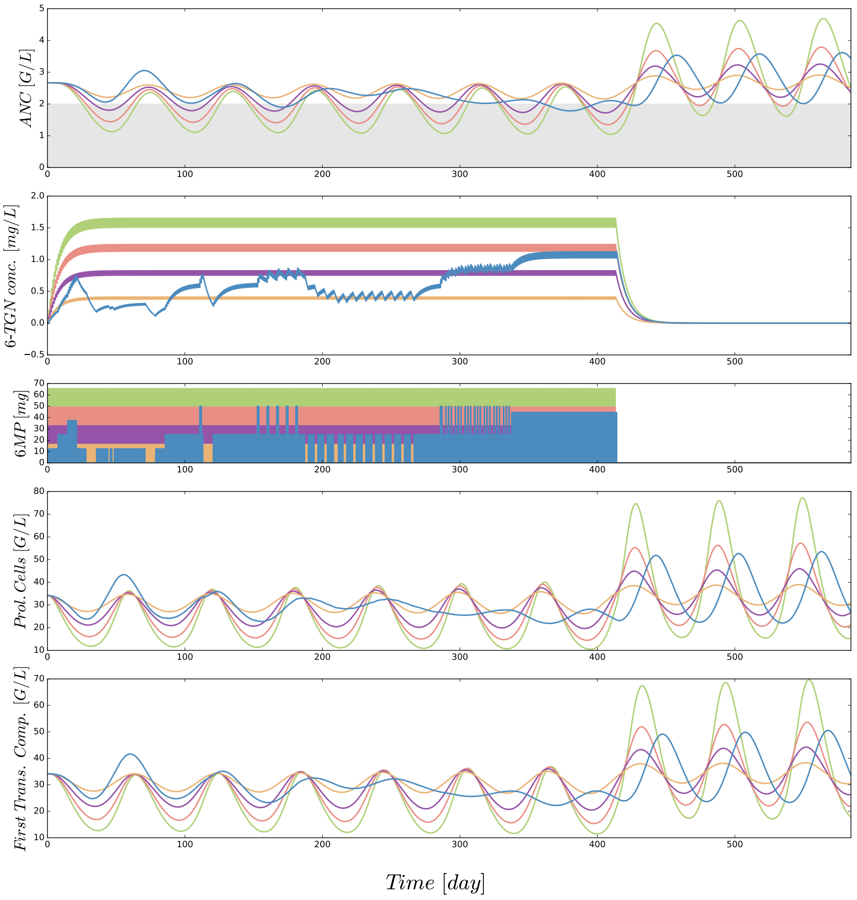

Figure 58: As Figure 2, but for another patient out of 116 patients.

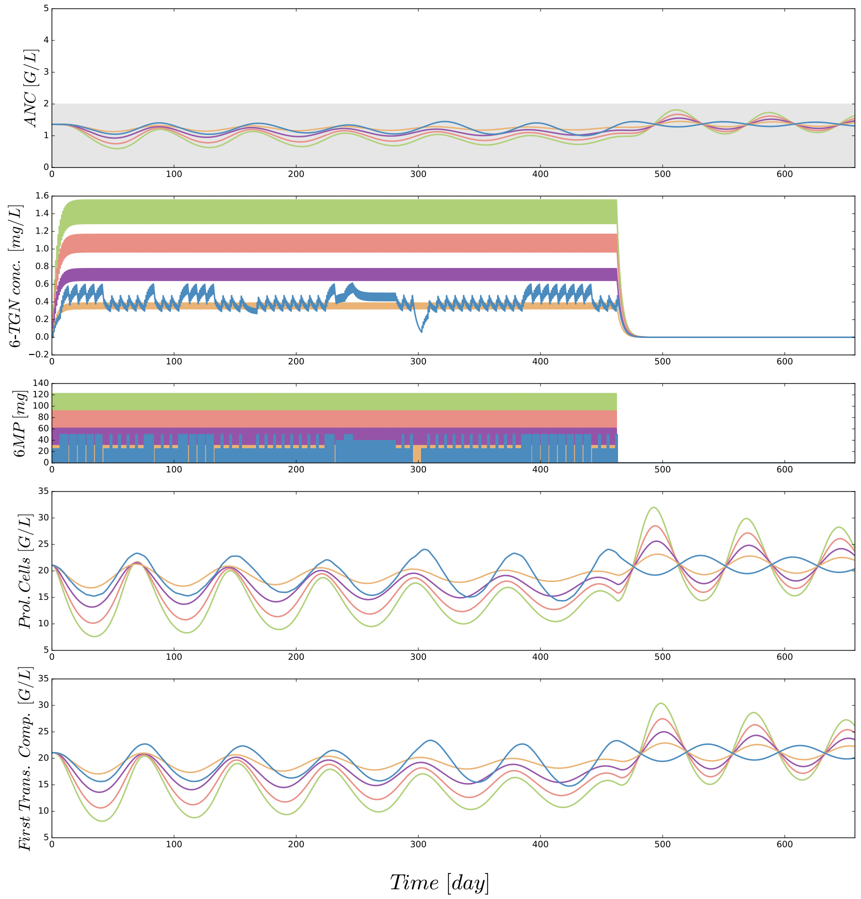

Figure 59: As Figure 2, but for another patient out of 116 patients.

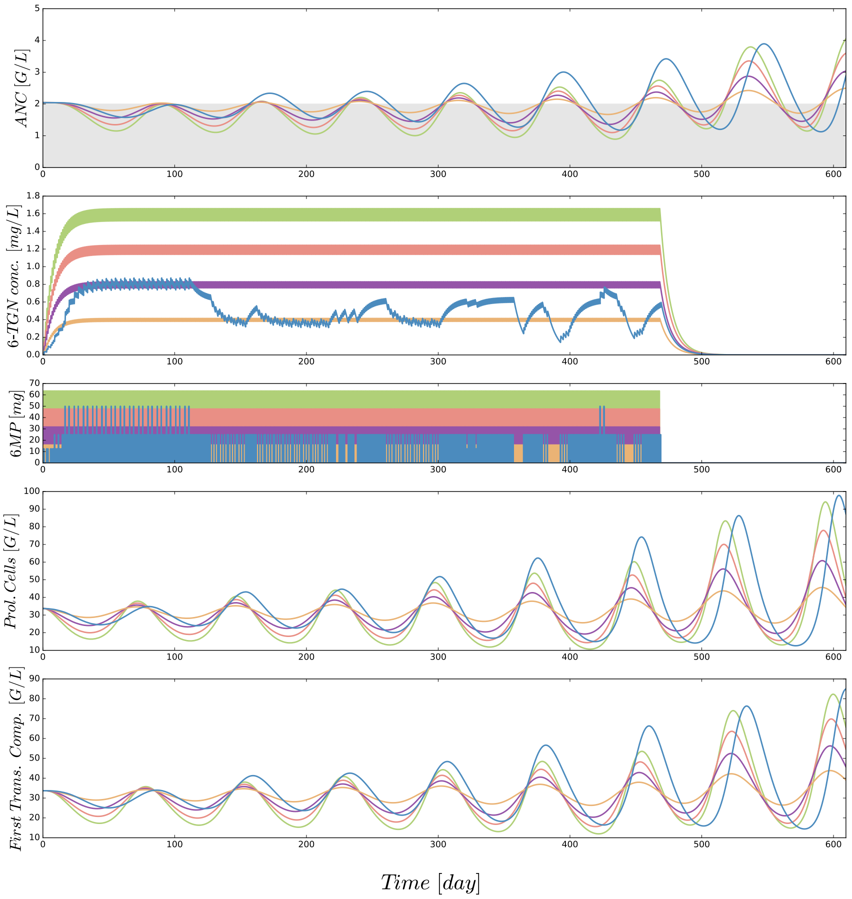

Figure 60: As Figure 2, but for another patient out of 116 patients.

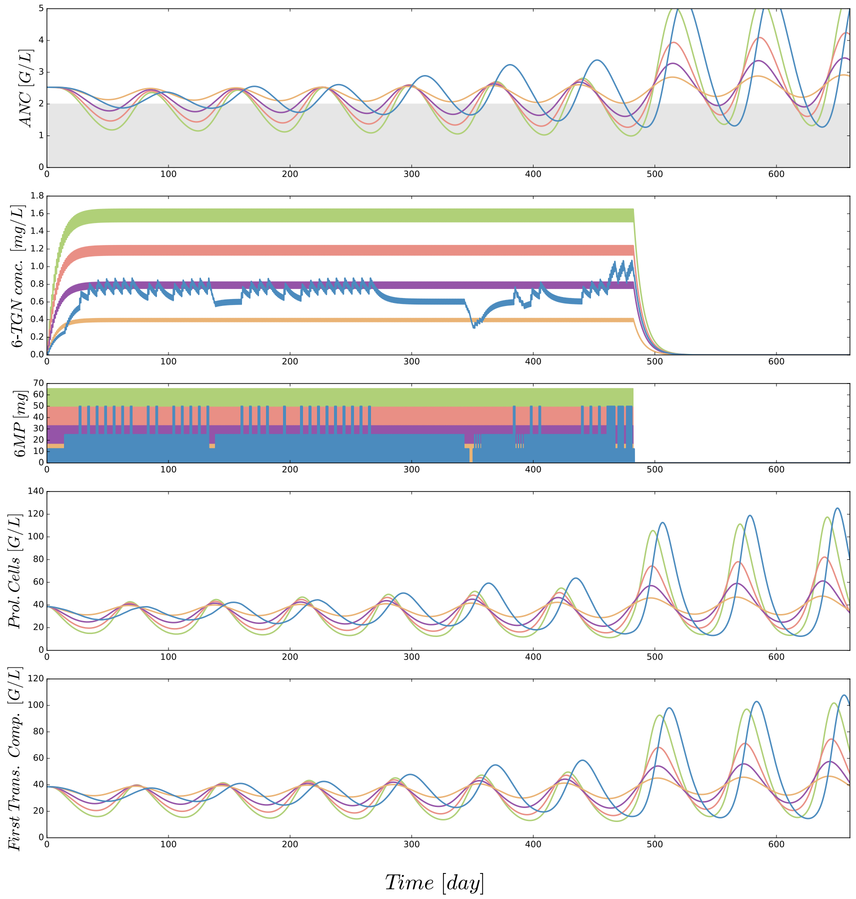

Figure 61: As Figure 2, but for another patient out of 116 patients.

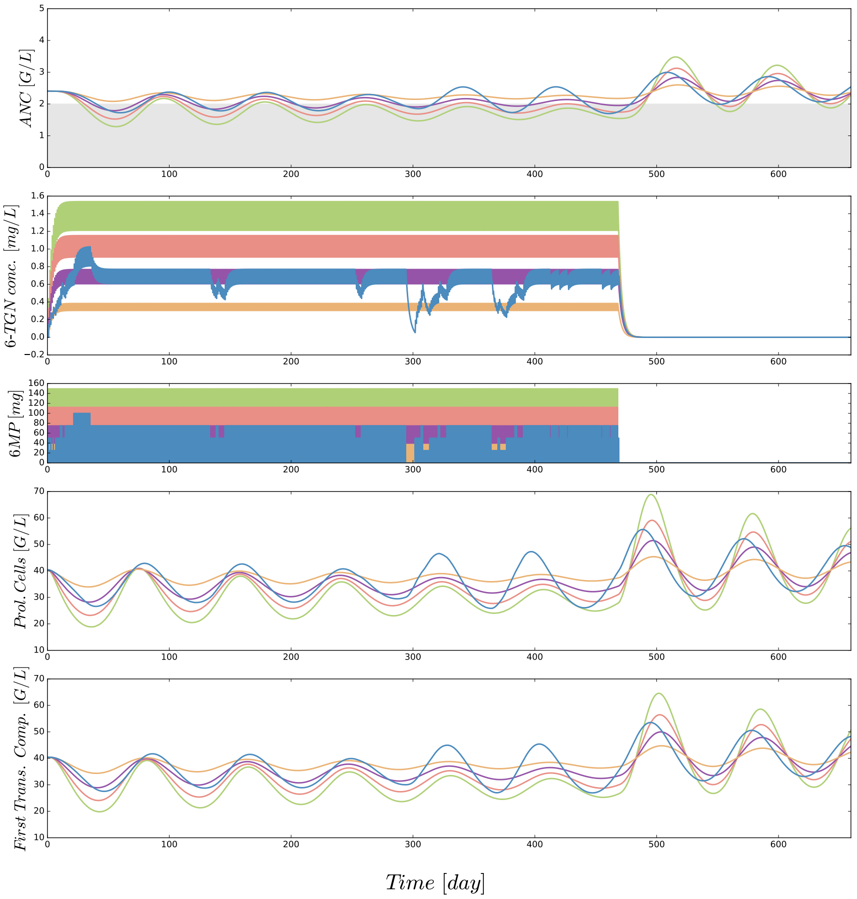

Figure 62: As Figure 2, but for another patient out of 116 patients.

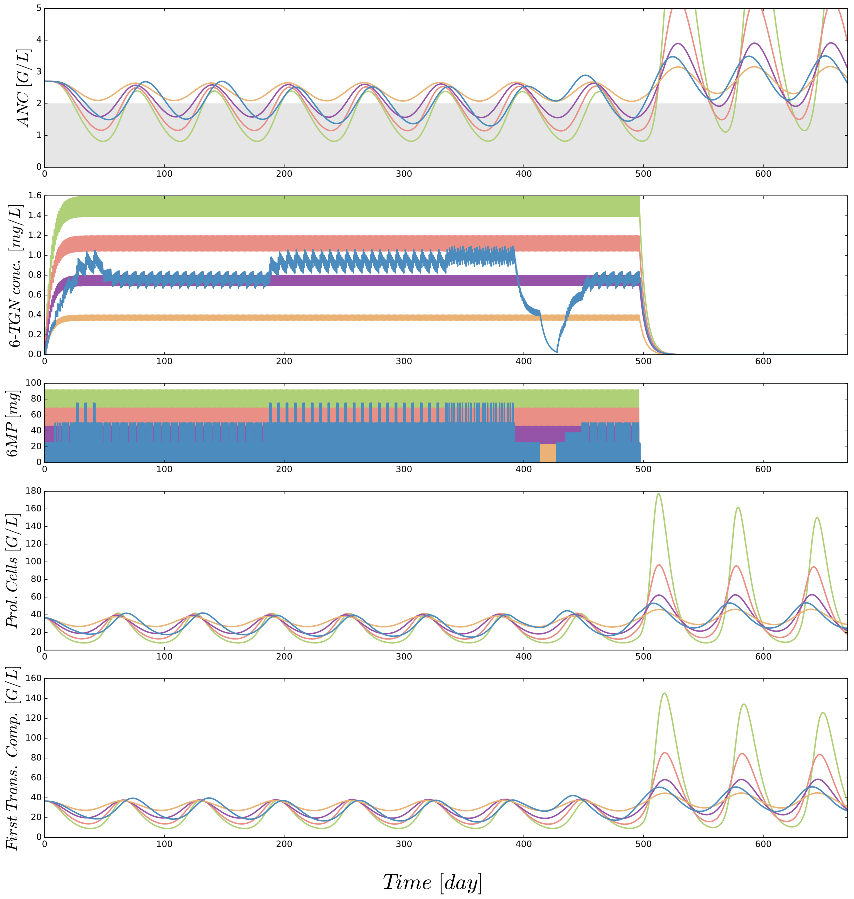

Figure 63: As Figure 2, but for another patient out of 116 patients.

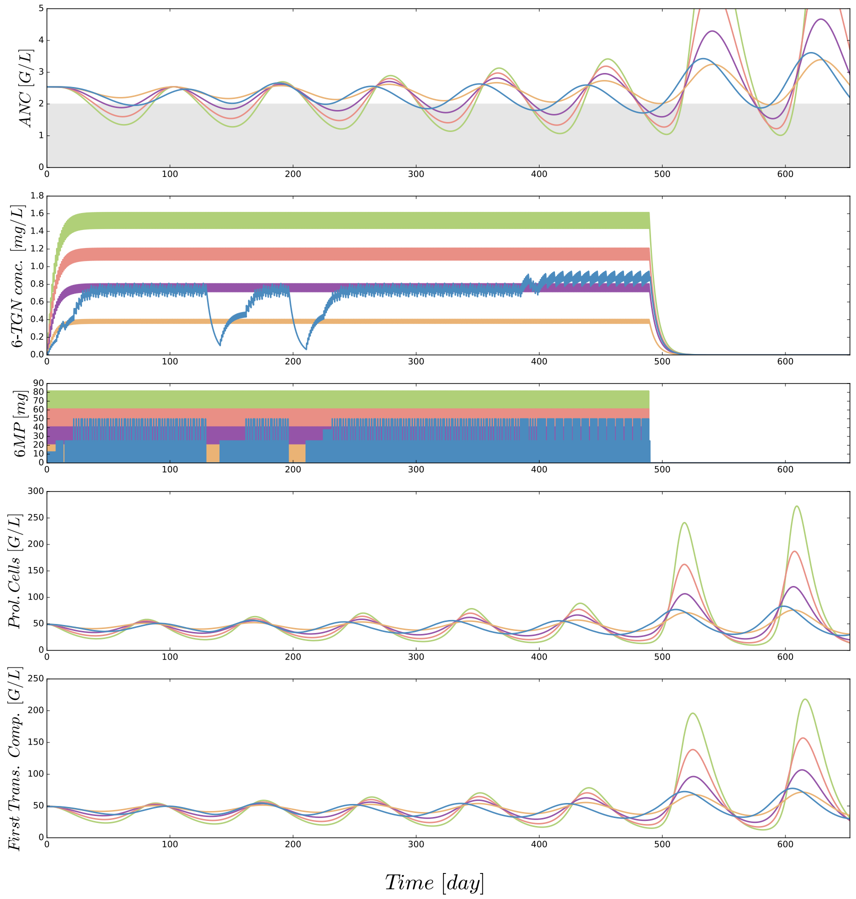

Figure 64: As Figure 2, but for another patient out of 116 patients.

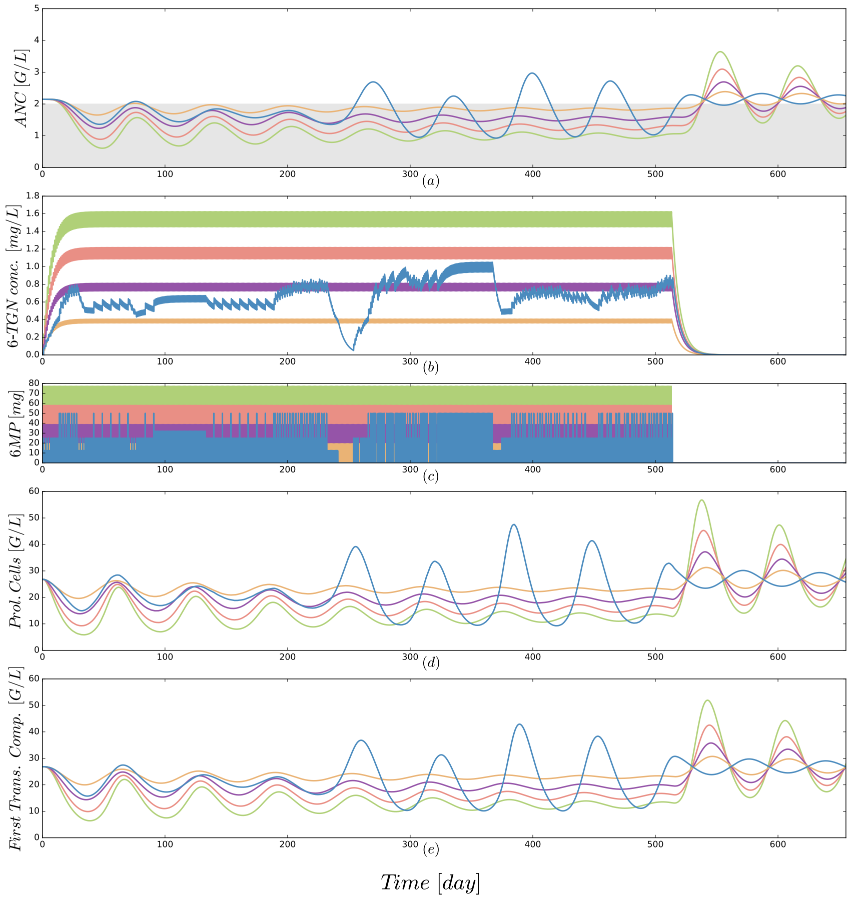

Figure 65: As Figure 2, but for another patient out of 116 patients.

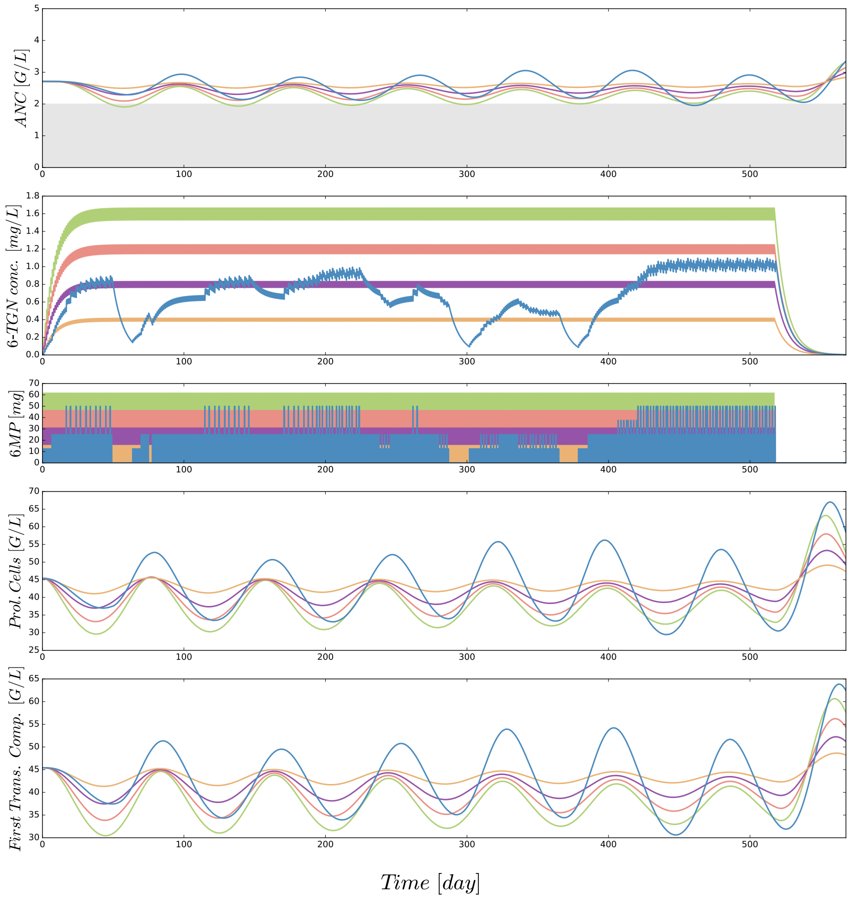

Figure 66: As Figure 2, but for another patient out of 116 patients.

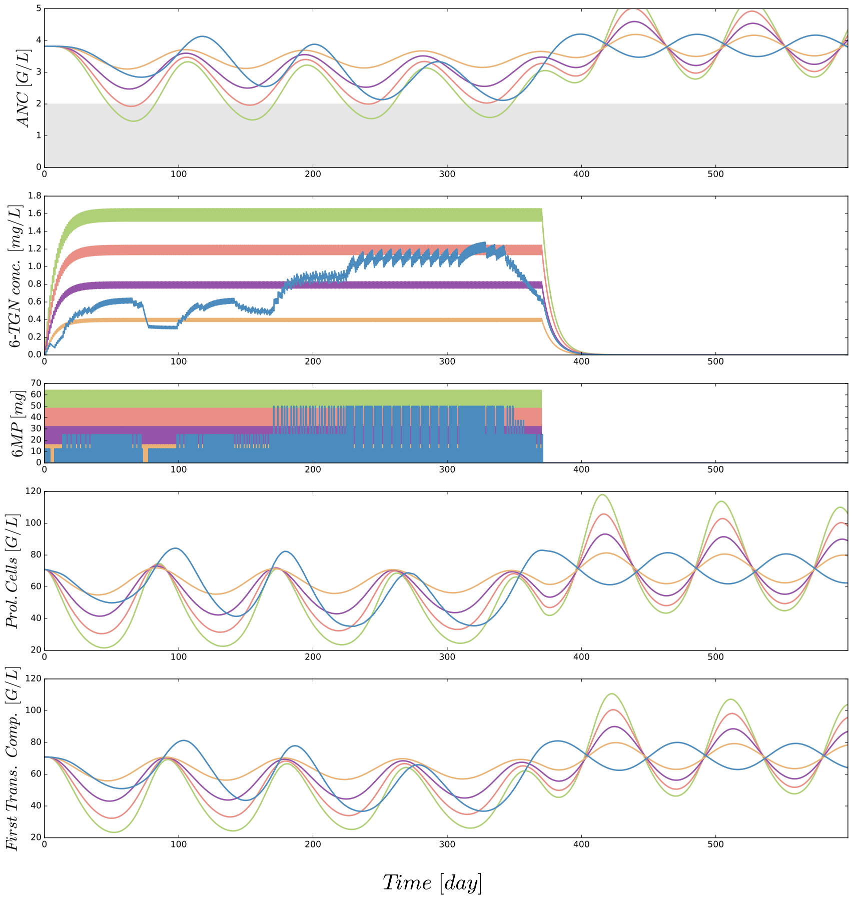

Figure 67: As Figure 2, but for another patient out of 116 patients.

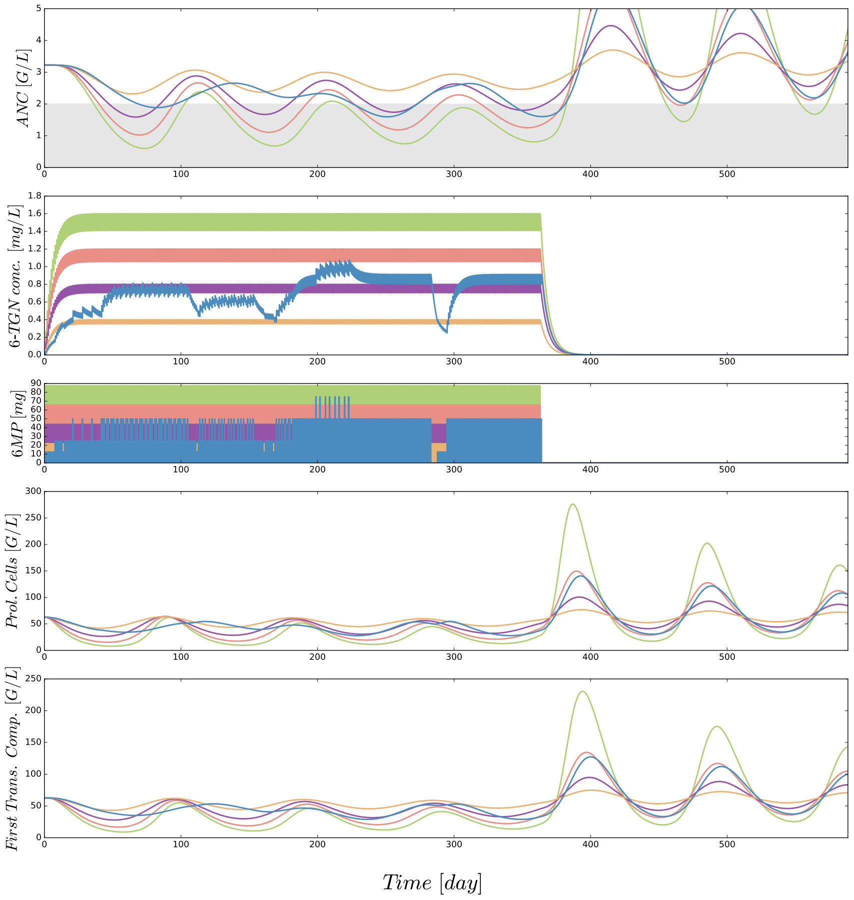

Figure 68: As Figure 2, but for another patient out of 116 patients.

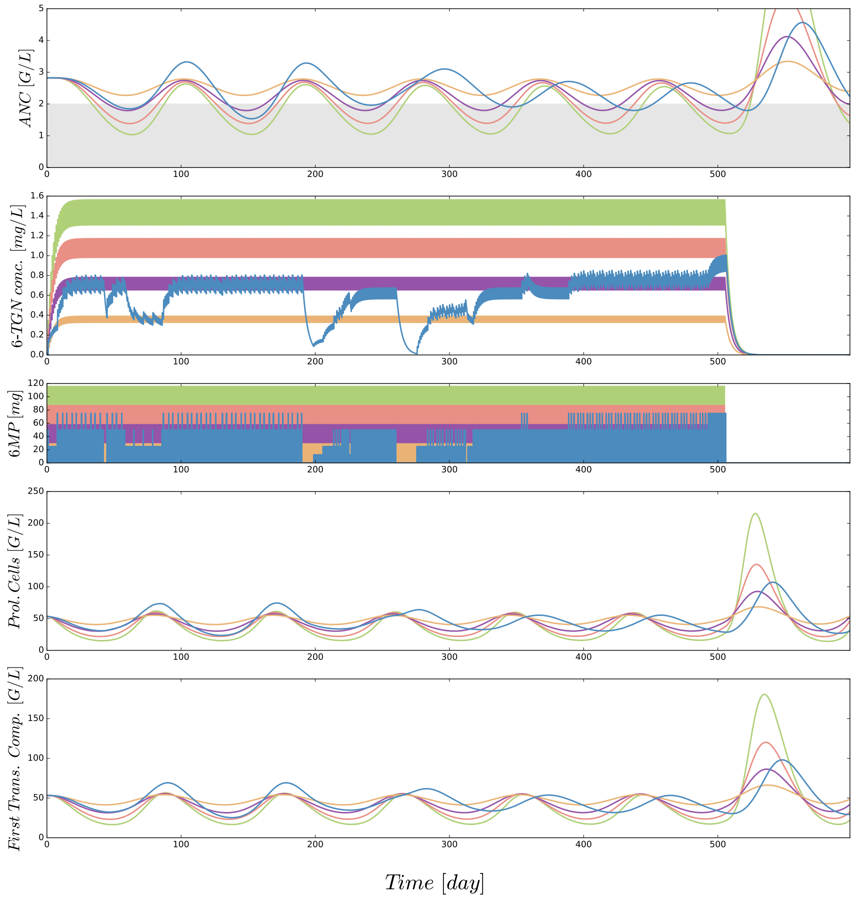

Figure 69: As Figure 2, but for another patient out of 116 patients.

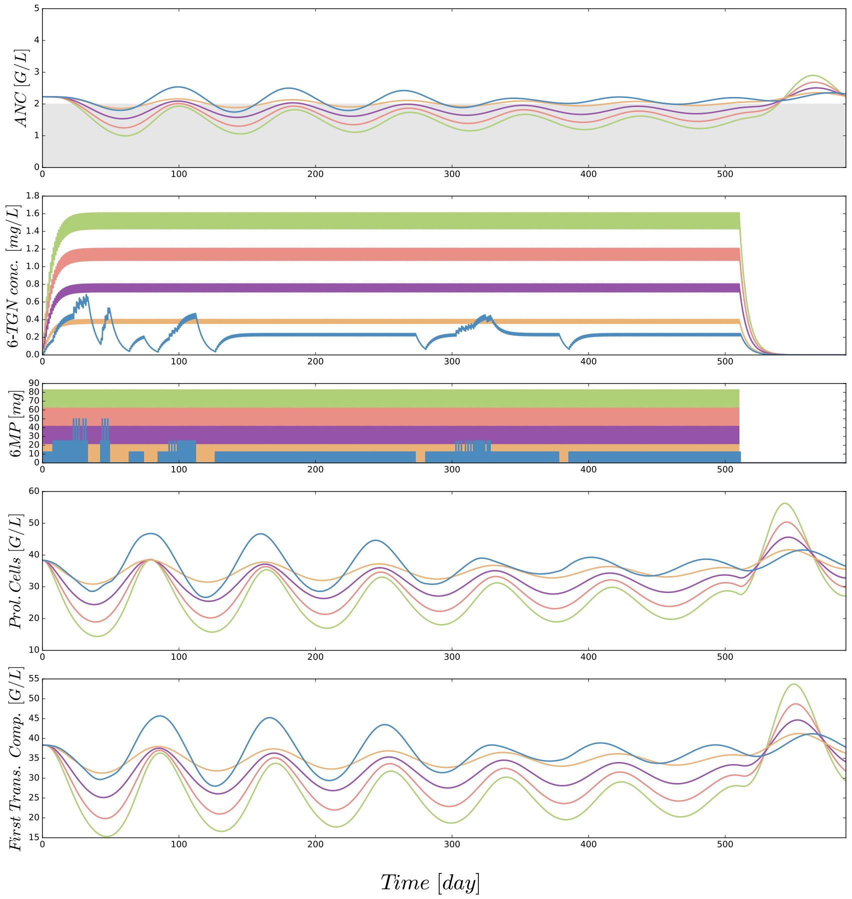

Figure 70: As Figure 2, but for another patient out of 116 patients.

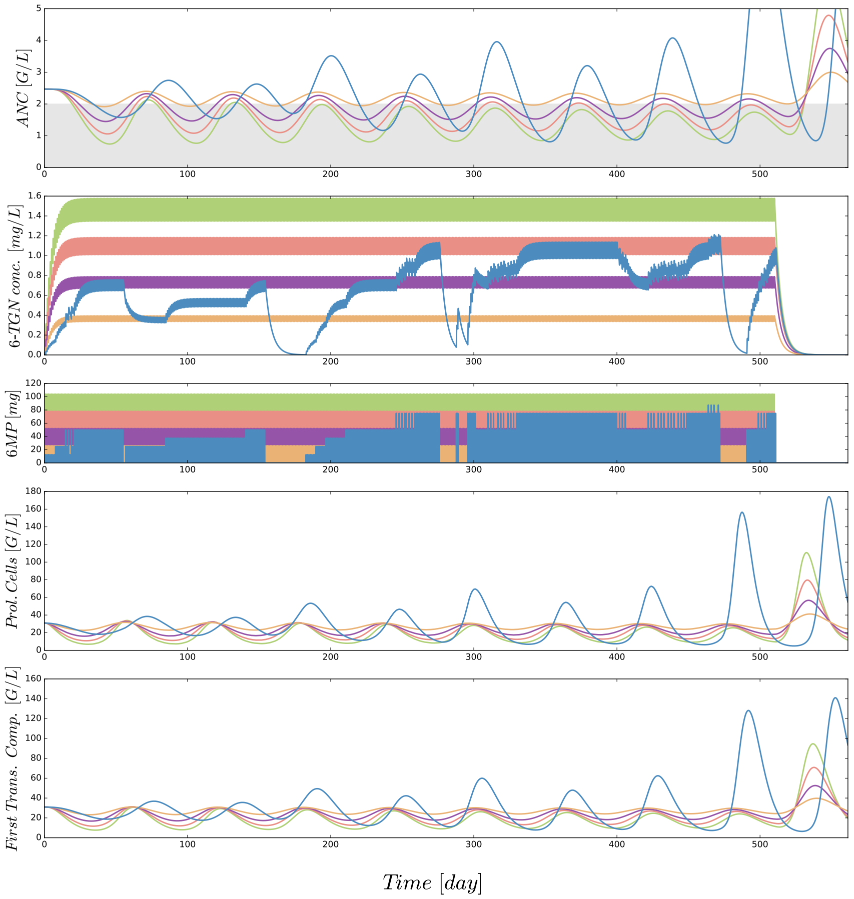

Figure 71: As Figure 2, but for another patient out of 116 patients.

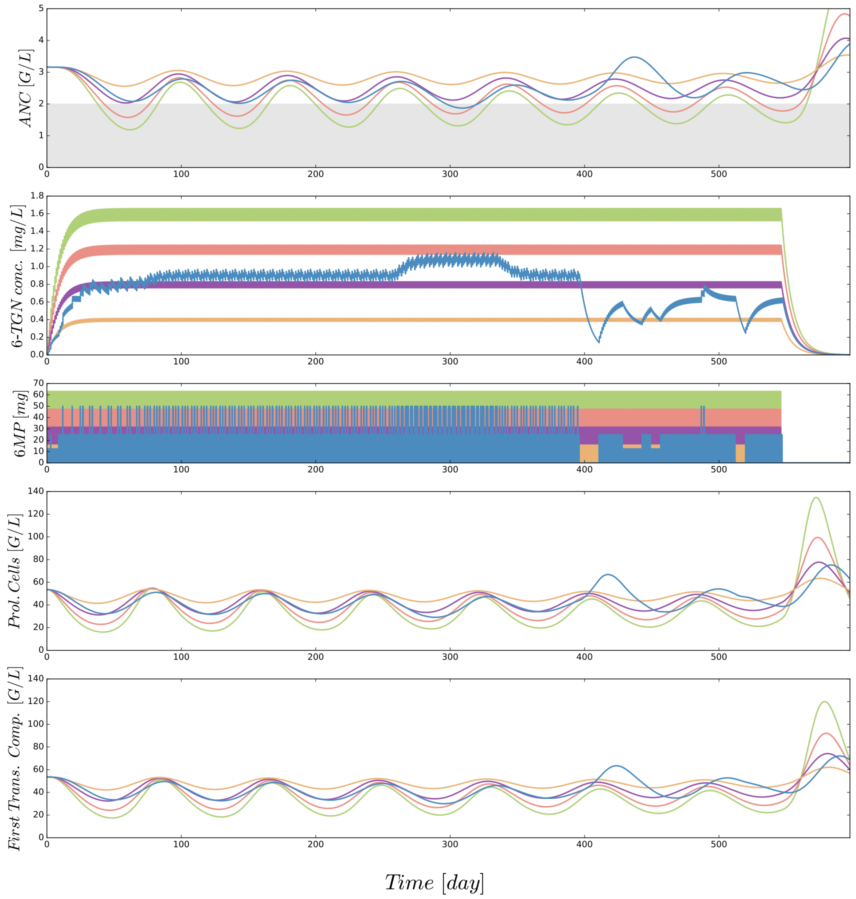

Figure 72: As Figure 2, but for another patient out of 116 patients.

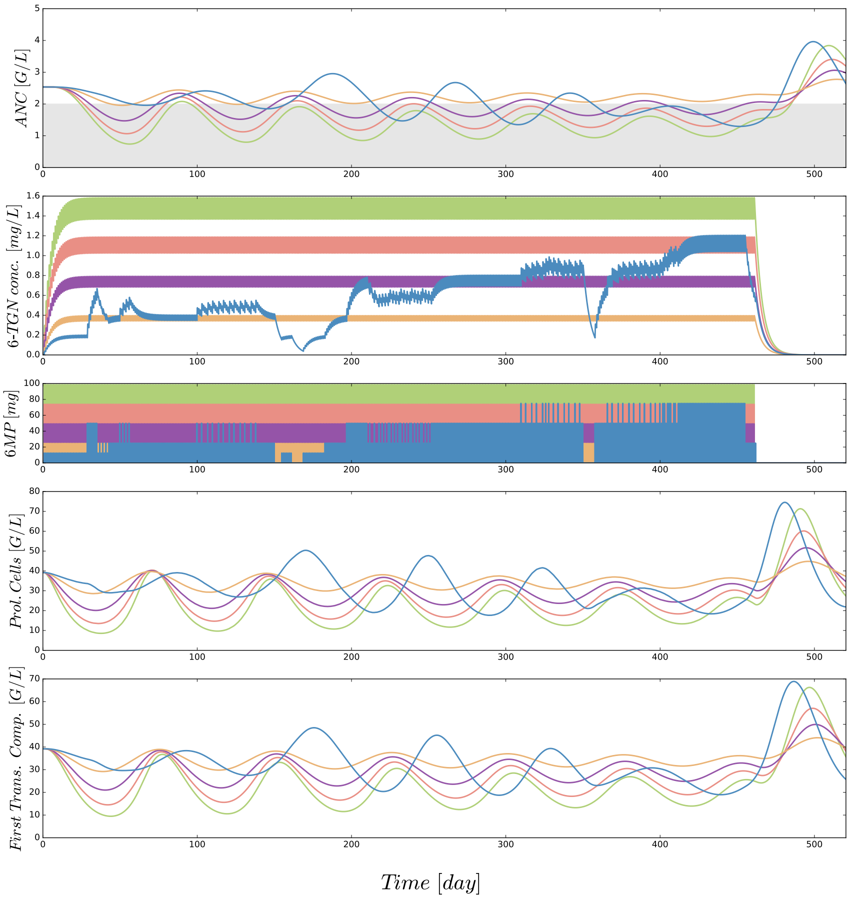

Figure 73: As Figure 2, but for another patient out of 116 patients.

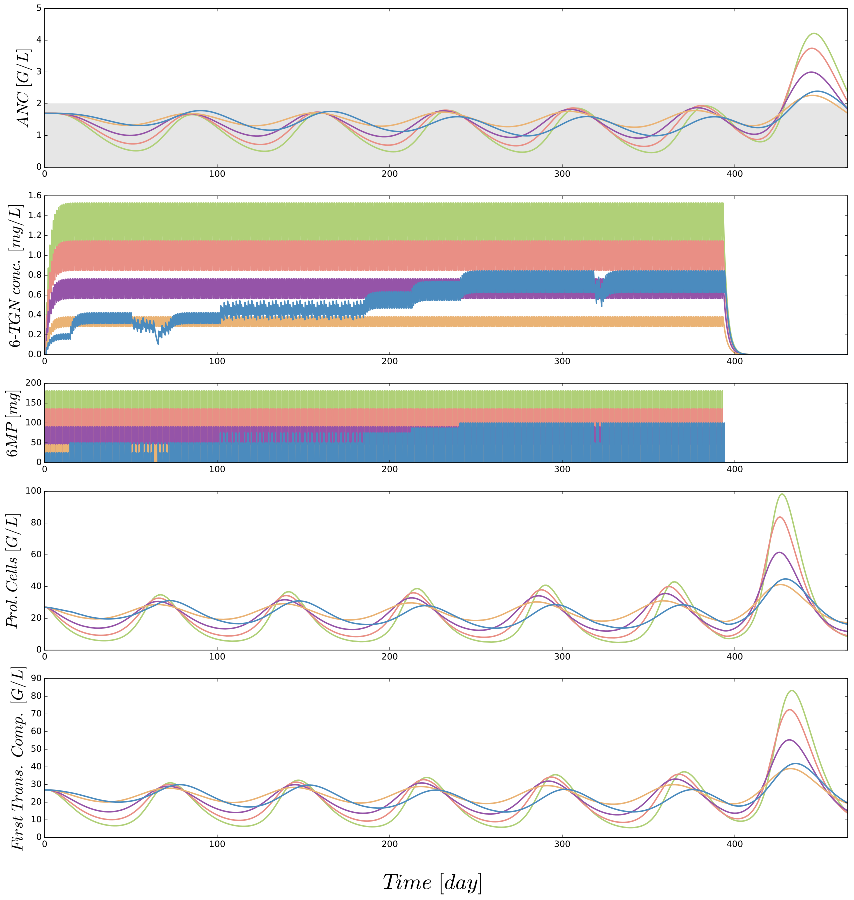

Figure 74: As Figure 2, but for another patient out of 116 patients.

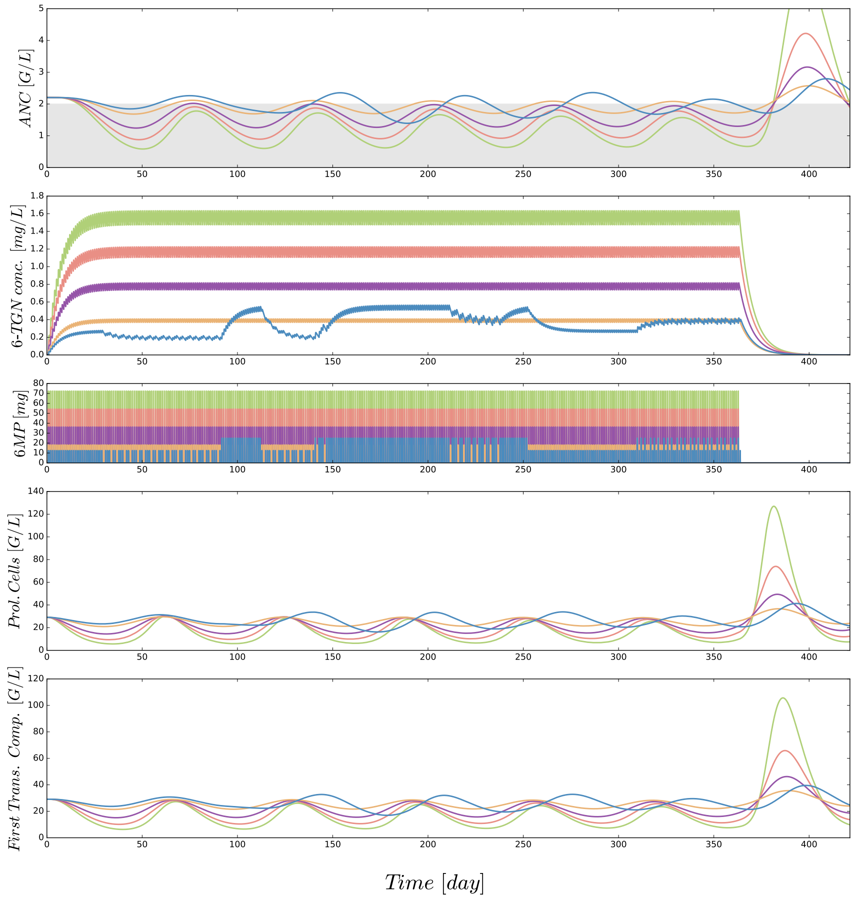

Figure 75: As Figure 2, but for another patient out of 116 patients.

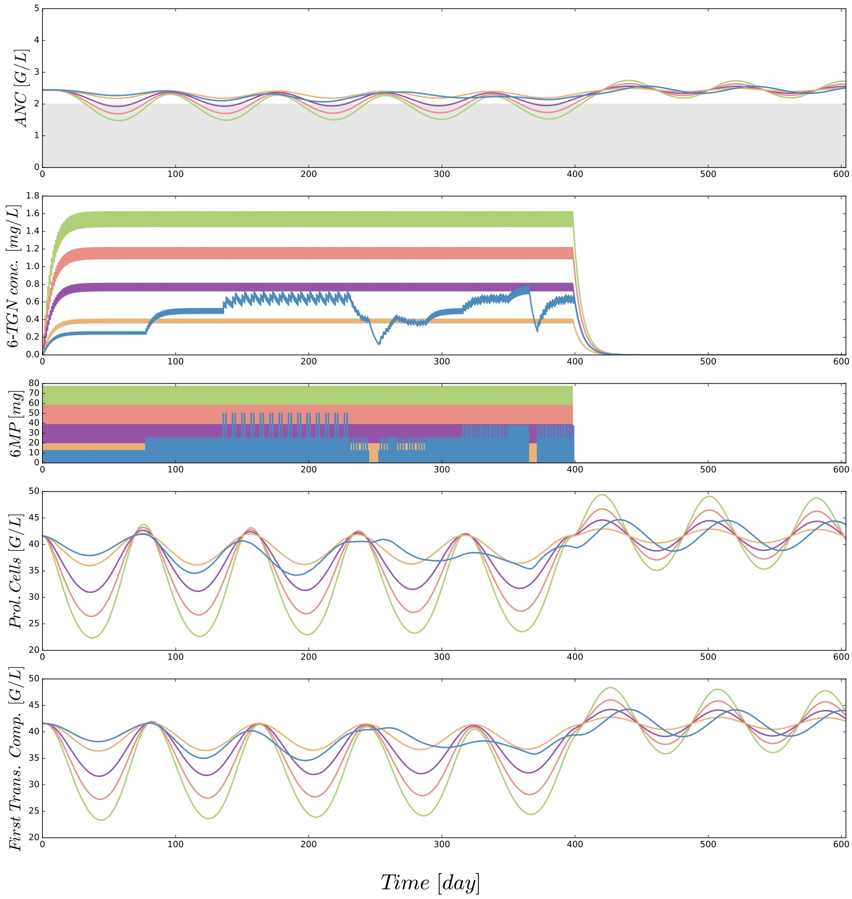

Figure 76: As Figure 2, but for another patient out of 116 patients.

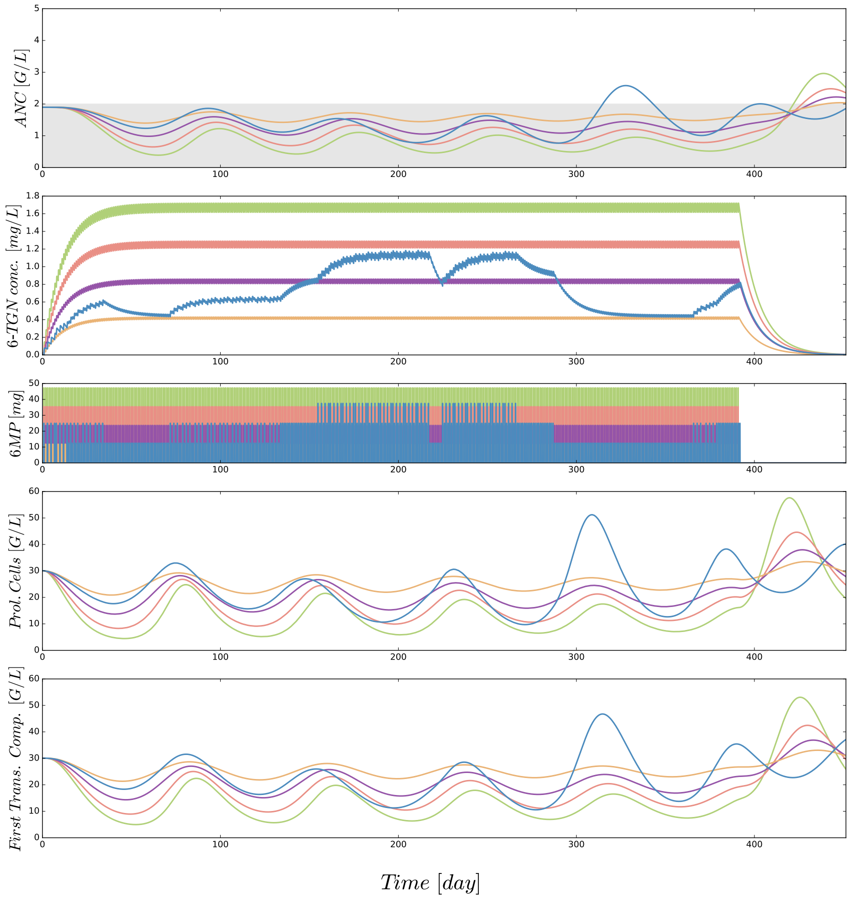

Figure 77: As Figure 2, but for another patient out of 116 patients.

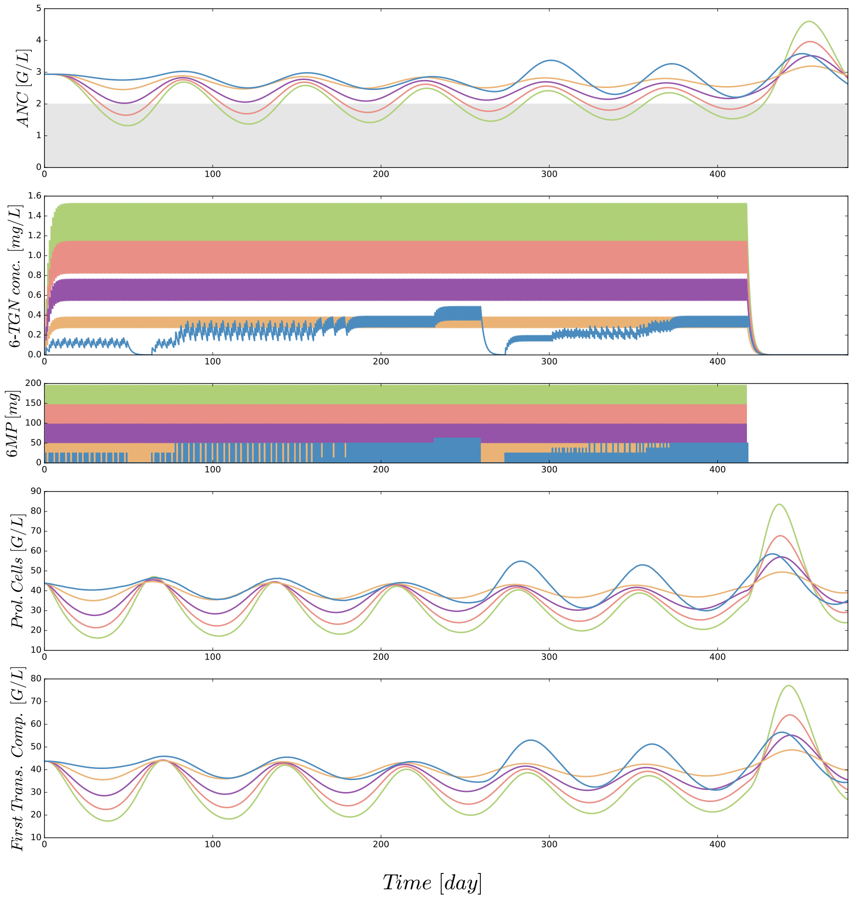

Figure 78: As Figure 2, but for another patient out of 116 patients.

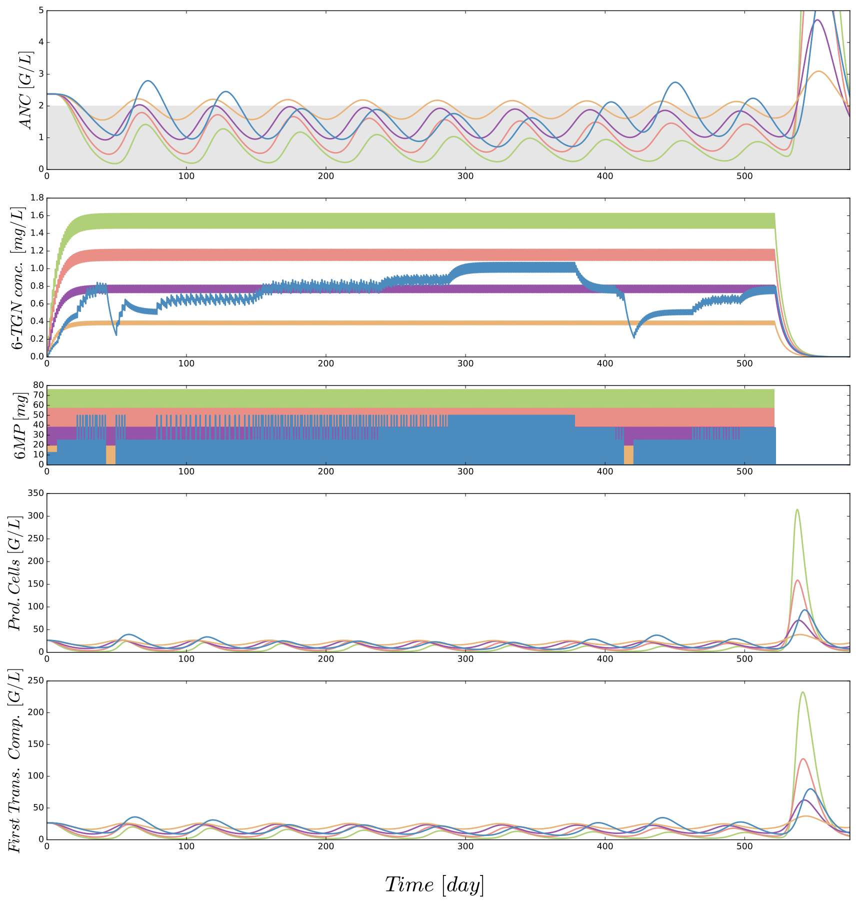

Figure 79: As Figure 2, but for another patient out of 116 patients.

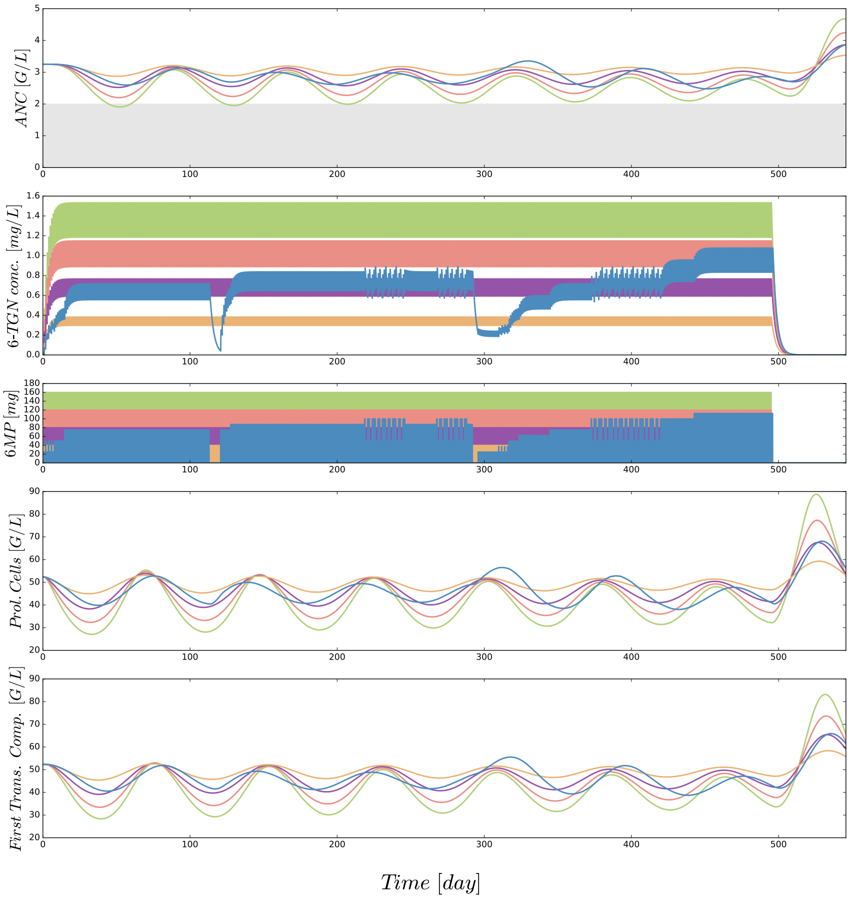

Figure 80: As Figure 2, but for another patient out of 116 patients.

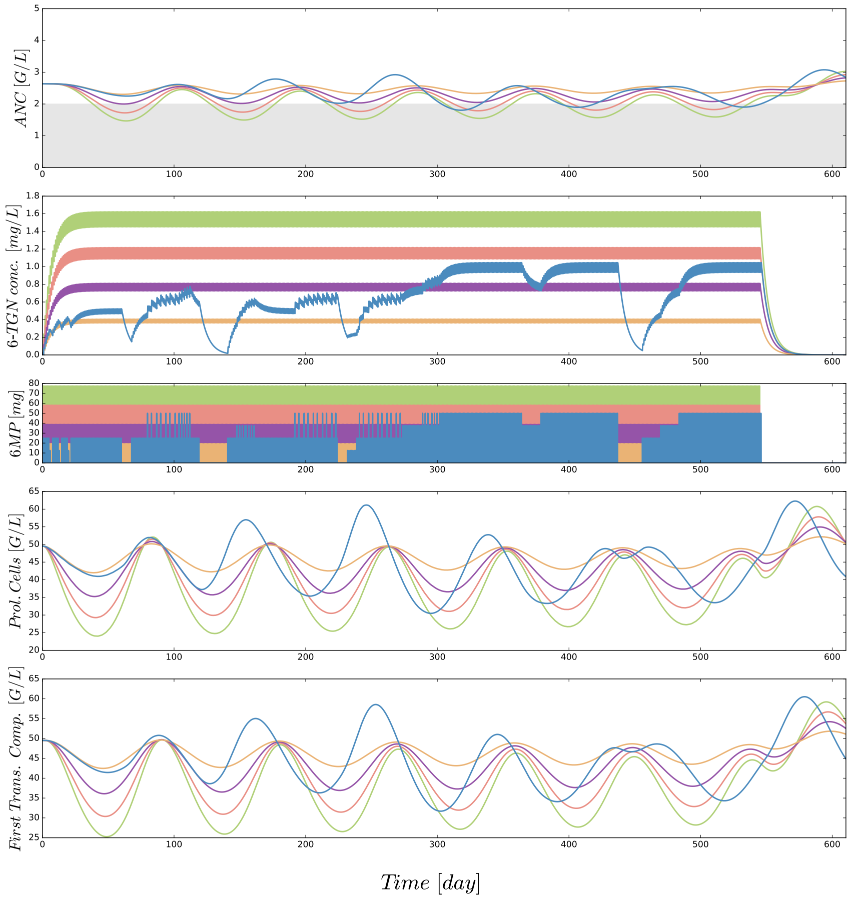

Figure 81: As Figure 2, but for another patient out of 116 patients.

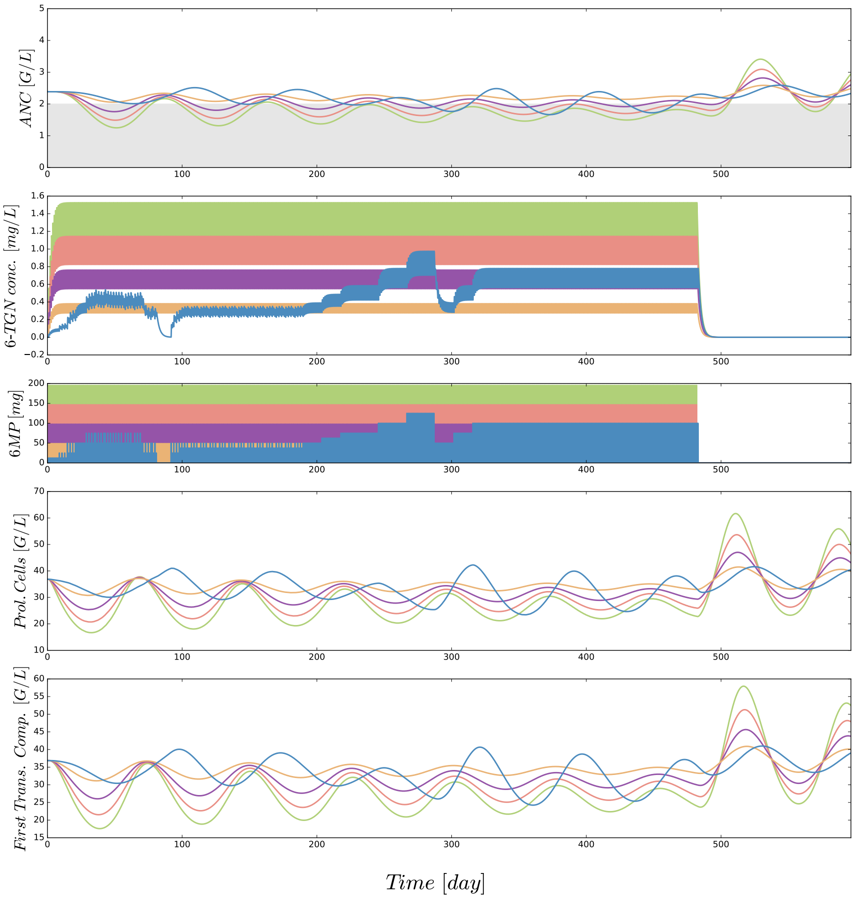

Figure 82: As Figure 2, but for another patient out of 116 patients.

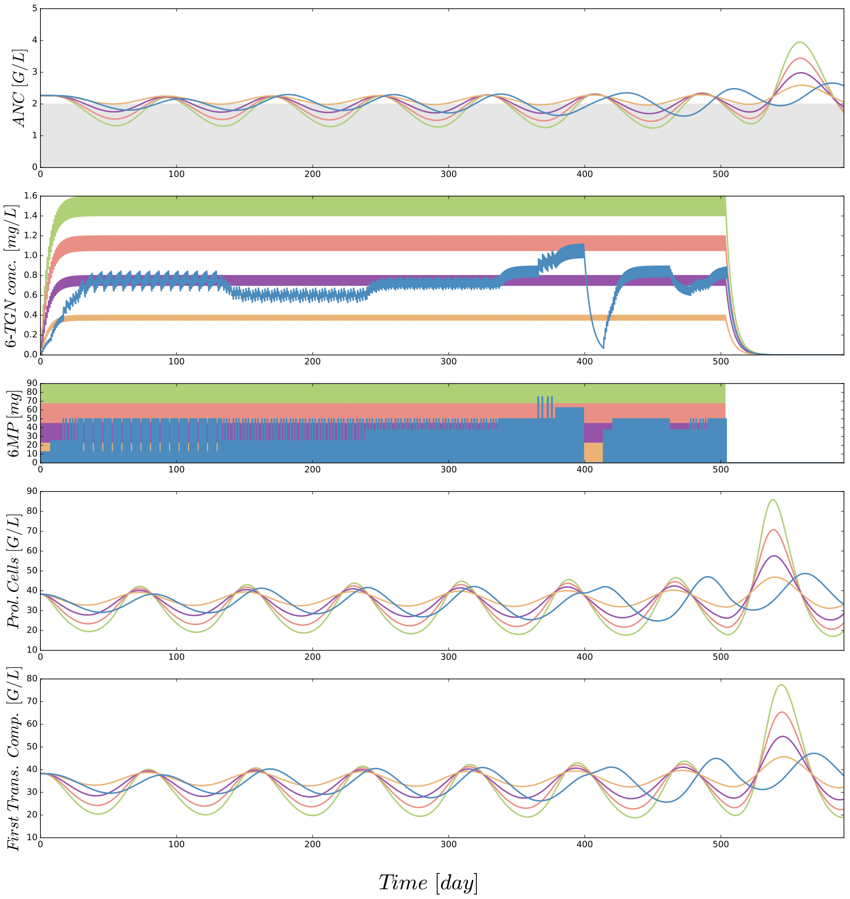

Figure 83: As Figure 2, but for another patient out of 116 patients.

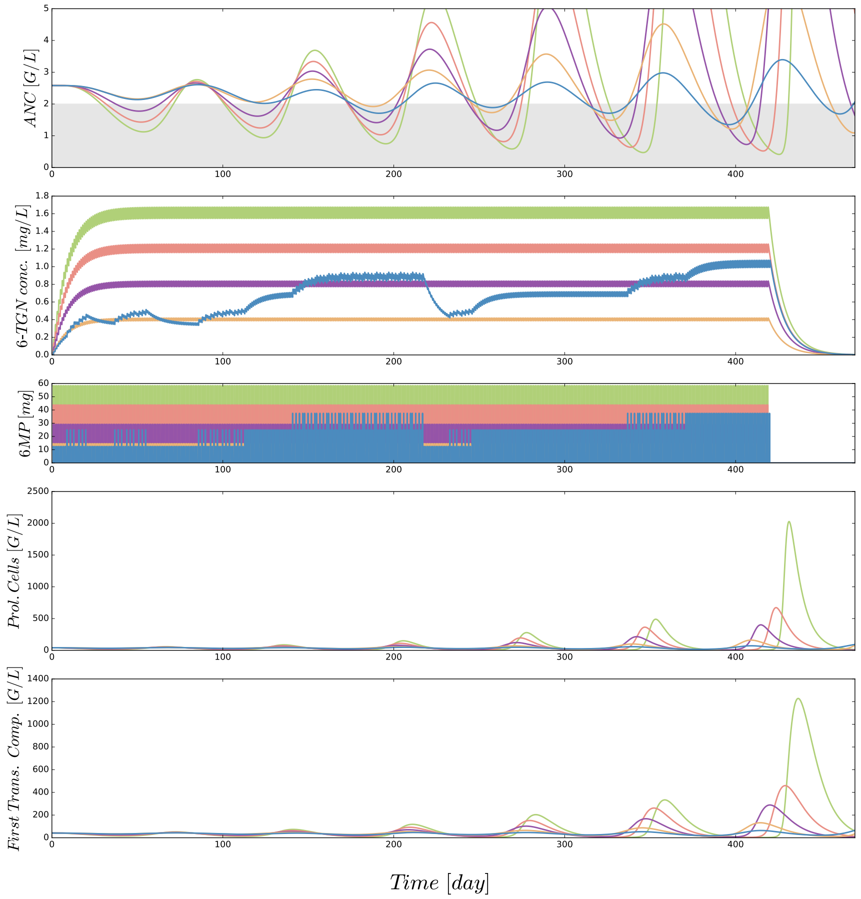

Figure 84: As Figure 2, but for another patient out of 116 patients.

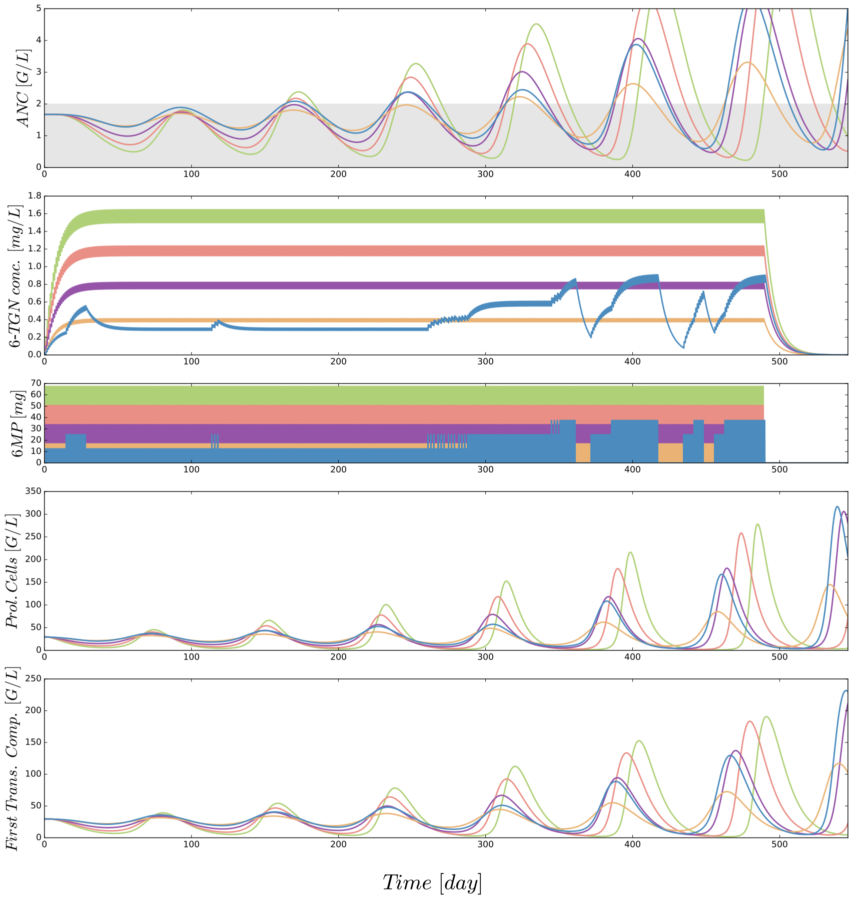

Figure 85: As Figure 2, but for another patient out of 116 patients.

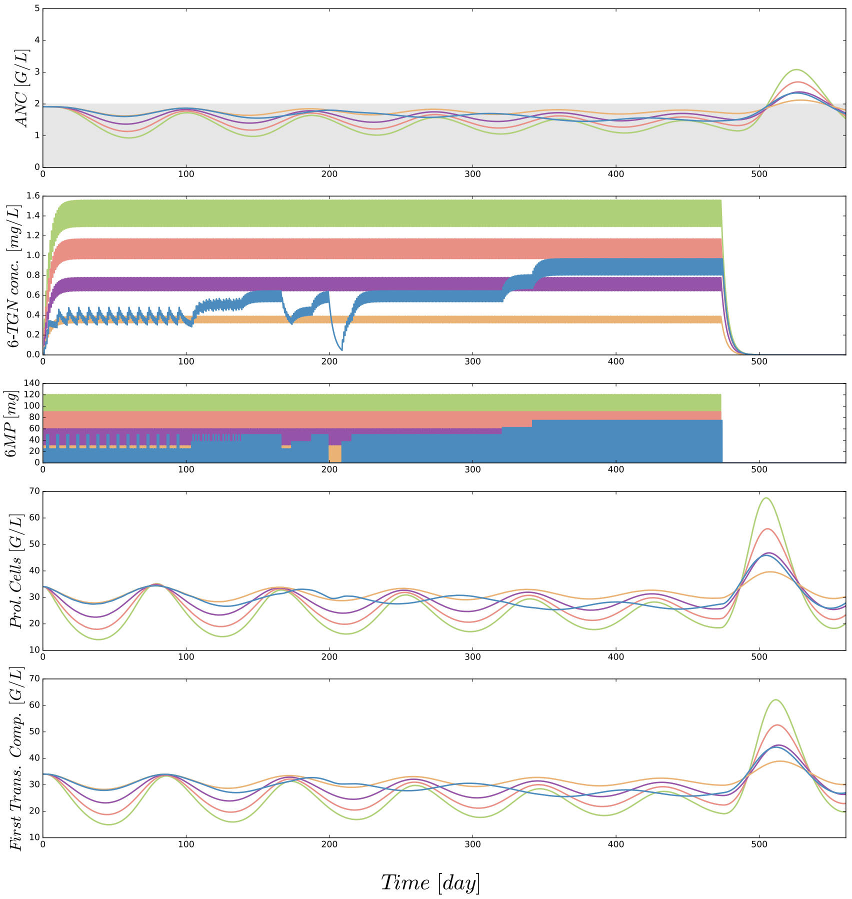

Figure 86: As Figure 2, but for another patient out of 116 patients.

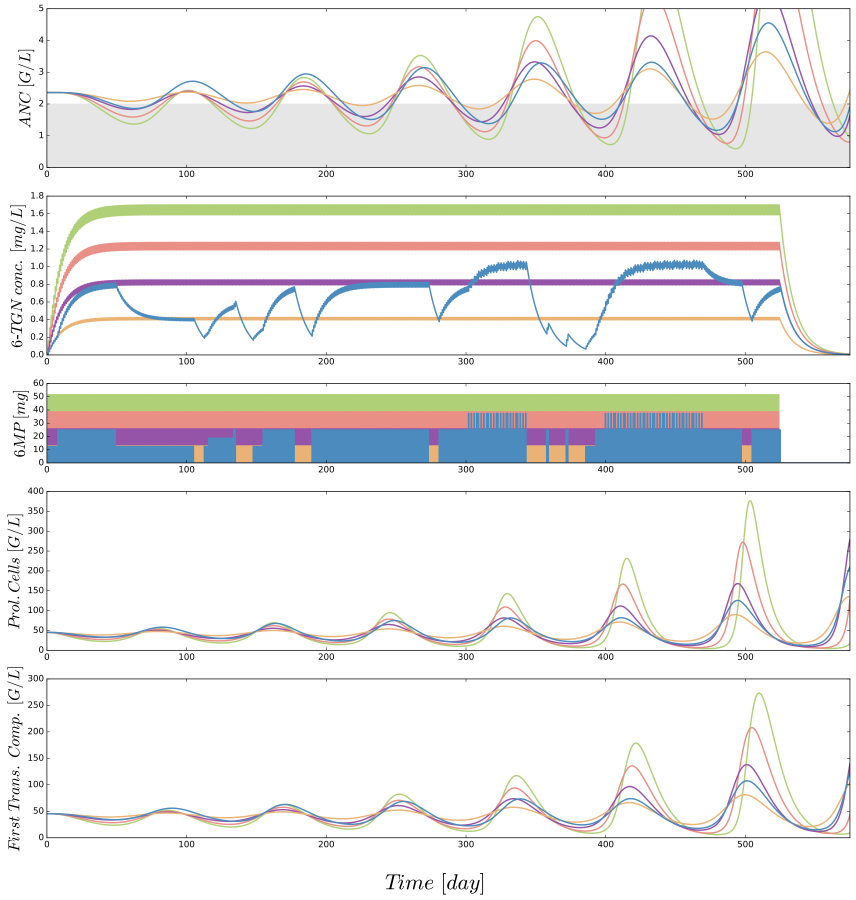

Figure 87: As Figure 2, but for another patient out of 116 patients.

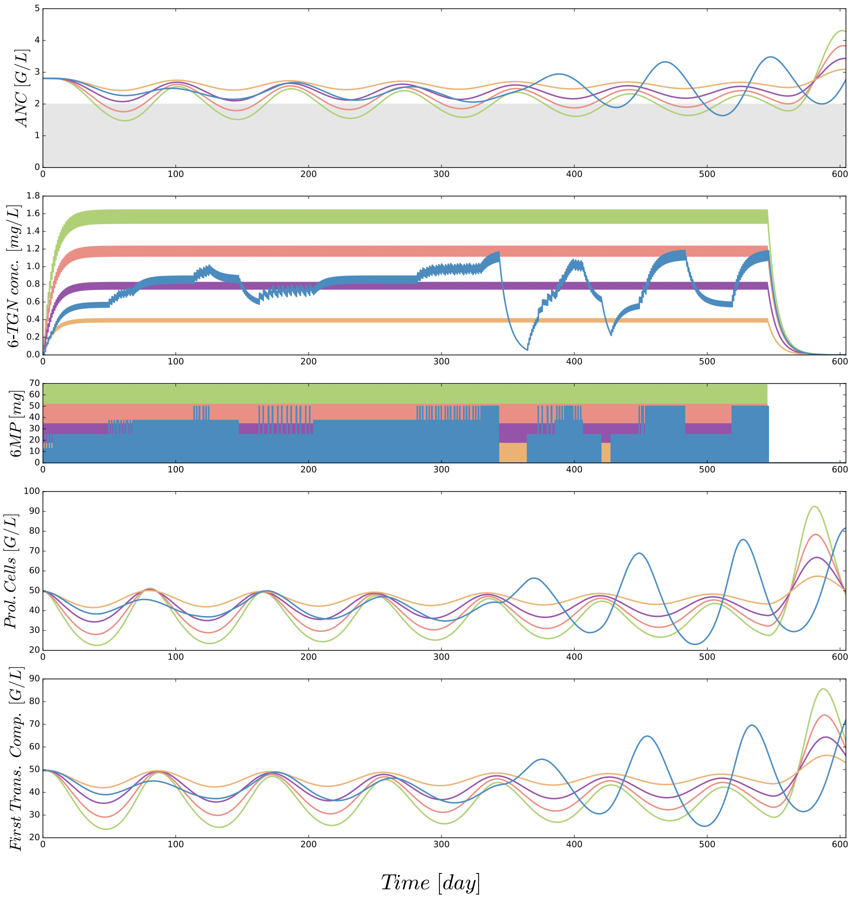

Figure 88: As Figure 2, but for another patient out of 116 patients.

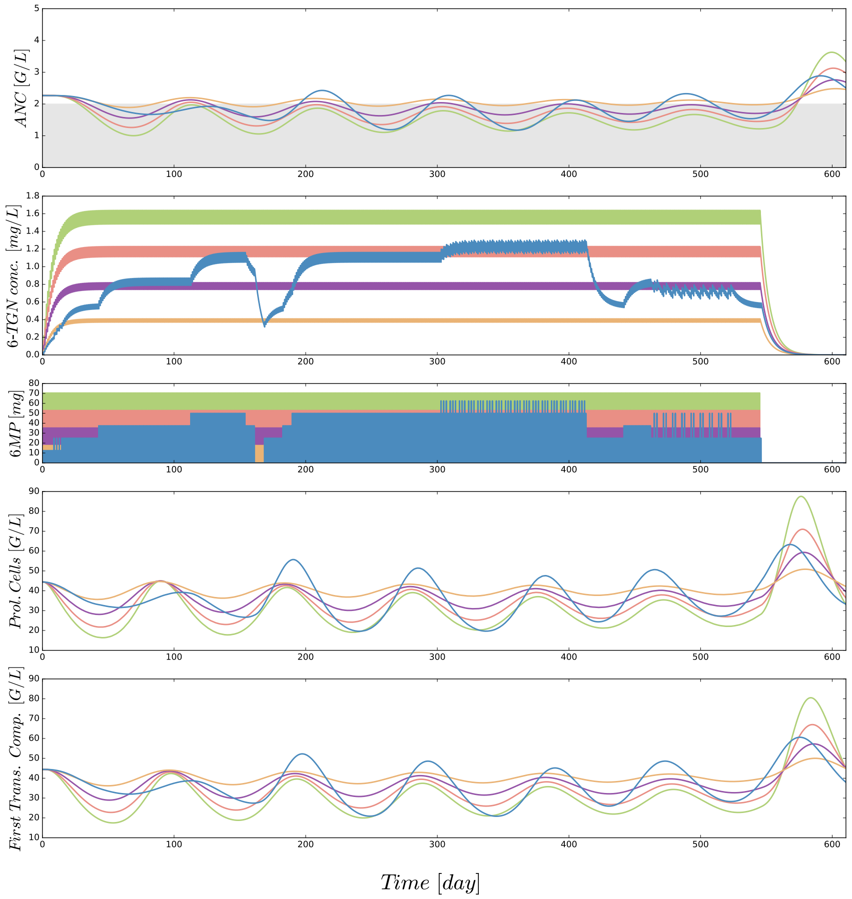

Figure 89: As Figure 2, but for another patient out of 116 patients.

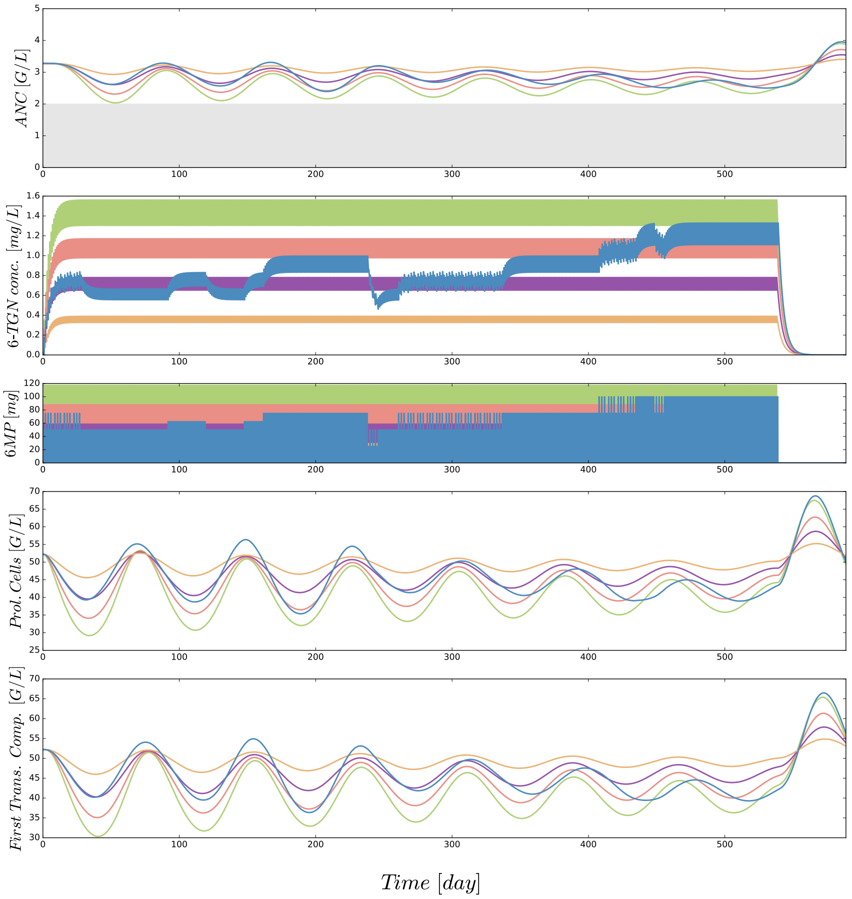

Figure 90: As Figure 2, but for another patient out of 116 patients.

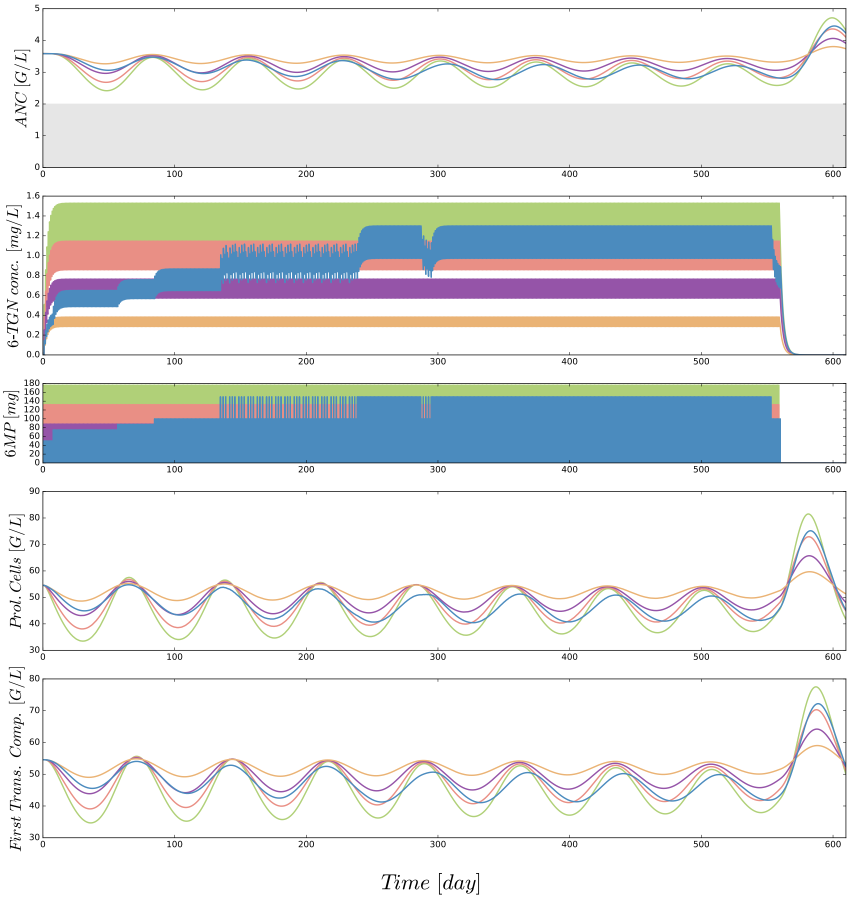

Figure 91: As Figure 2, but for another patient out of 116 patients.

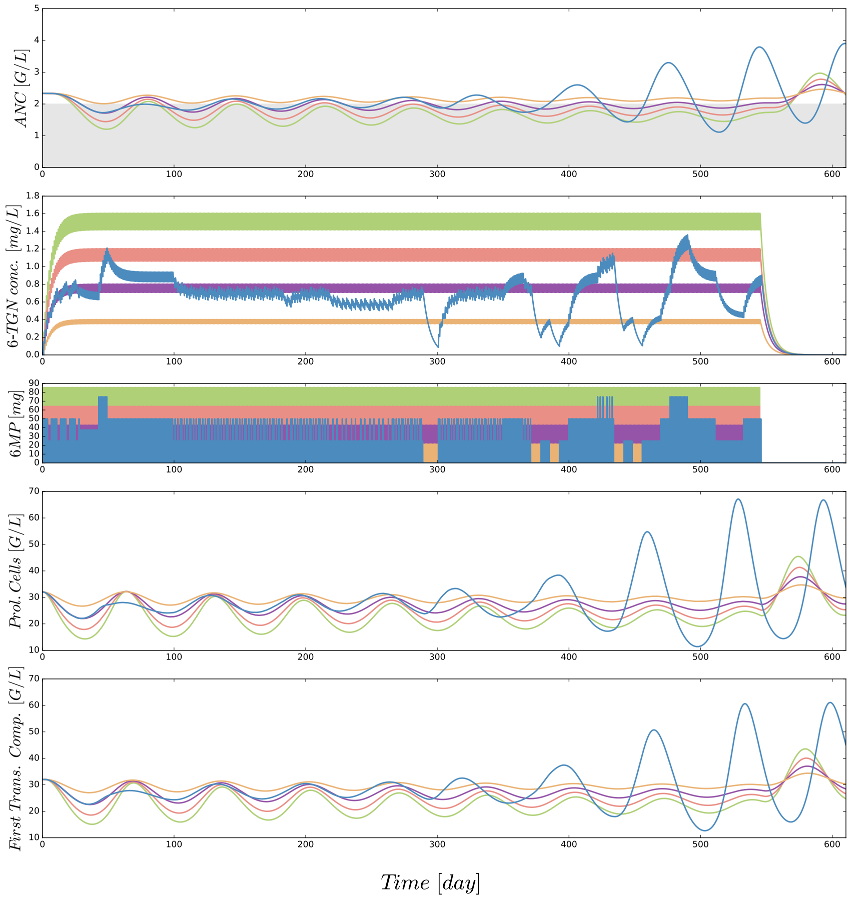

Figure 92: As Figure 2, but for another patient out of 116 patients.

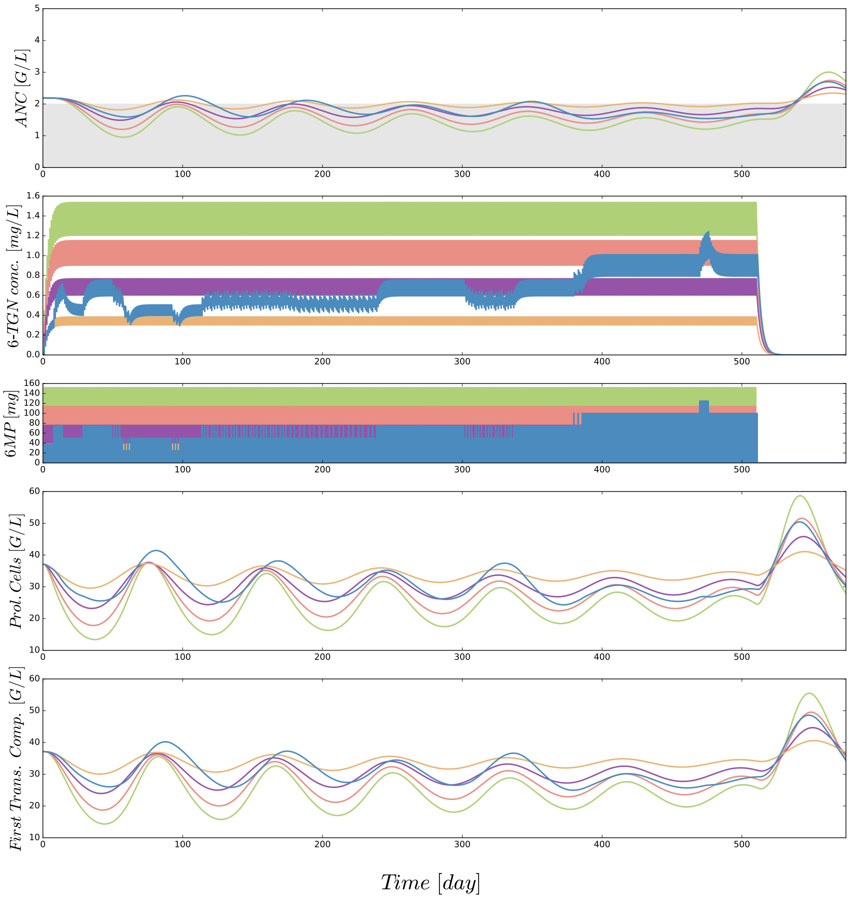

Figure 93: As Figure 2, but for another patient out of 116 patients.

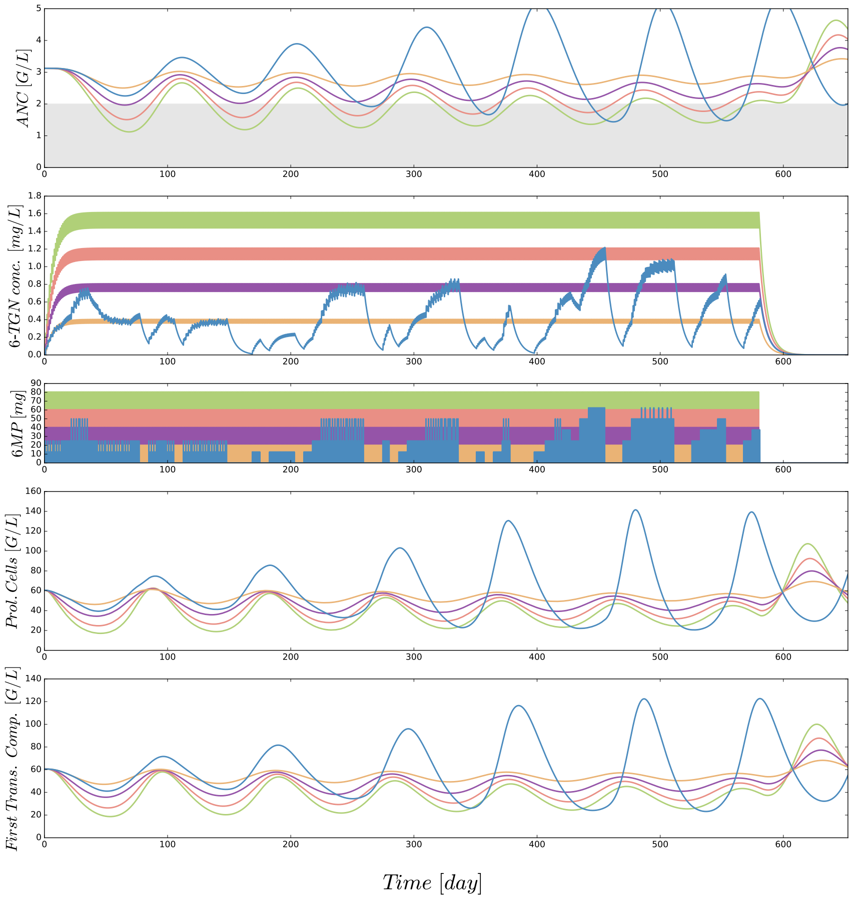

Figure 94: As Figure 2, but for another patient out of 116 patients.

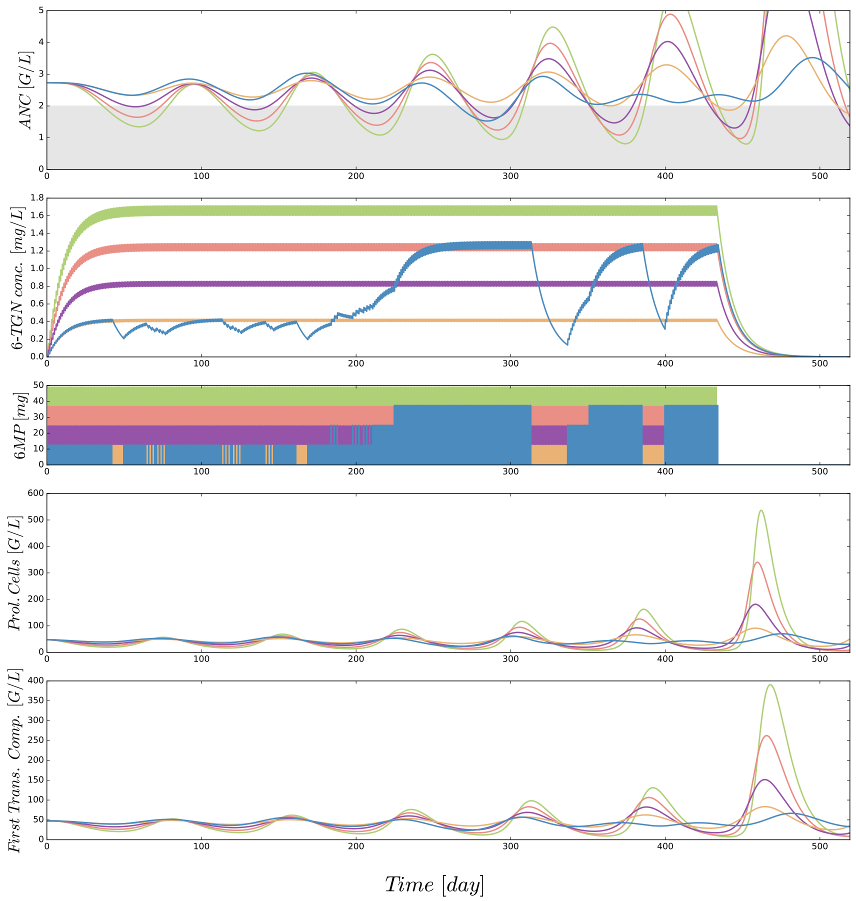

Figure 95: As Figure 2, but for another patient out of 116 patients.

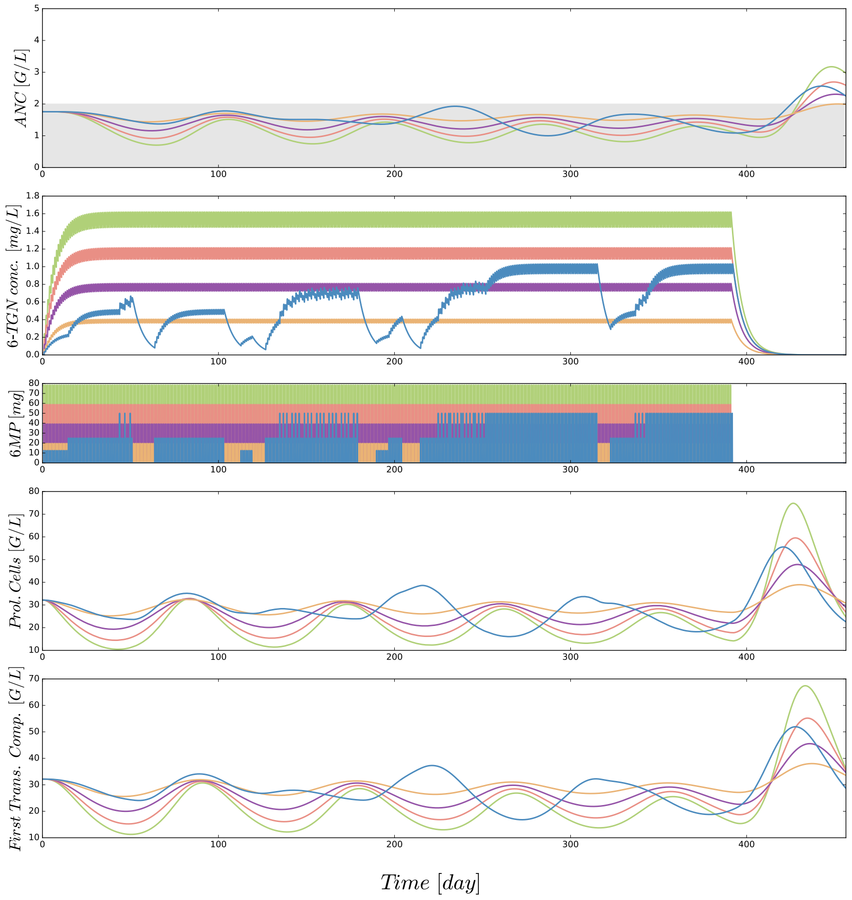

Figure 96: As Figure 2, but for another patient out of 116 patients.

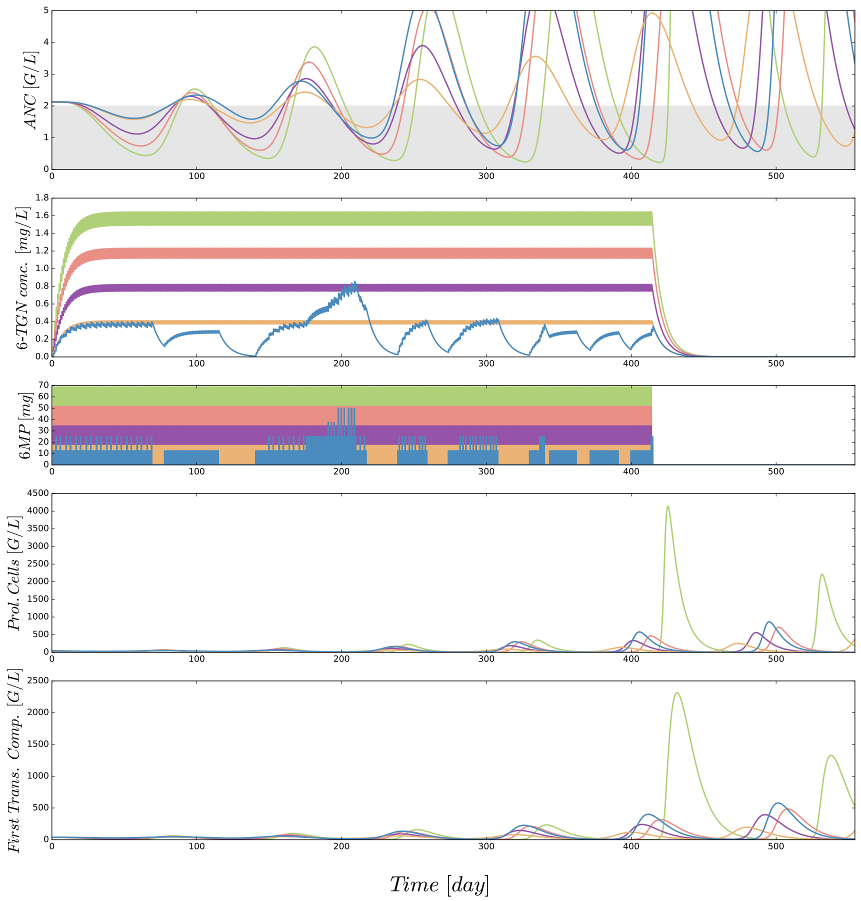

Figure 97: As Figure 2, but for another patient out of 116 patients.

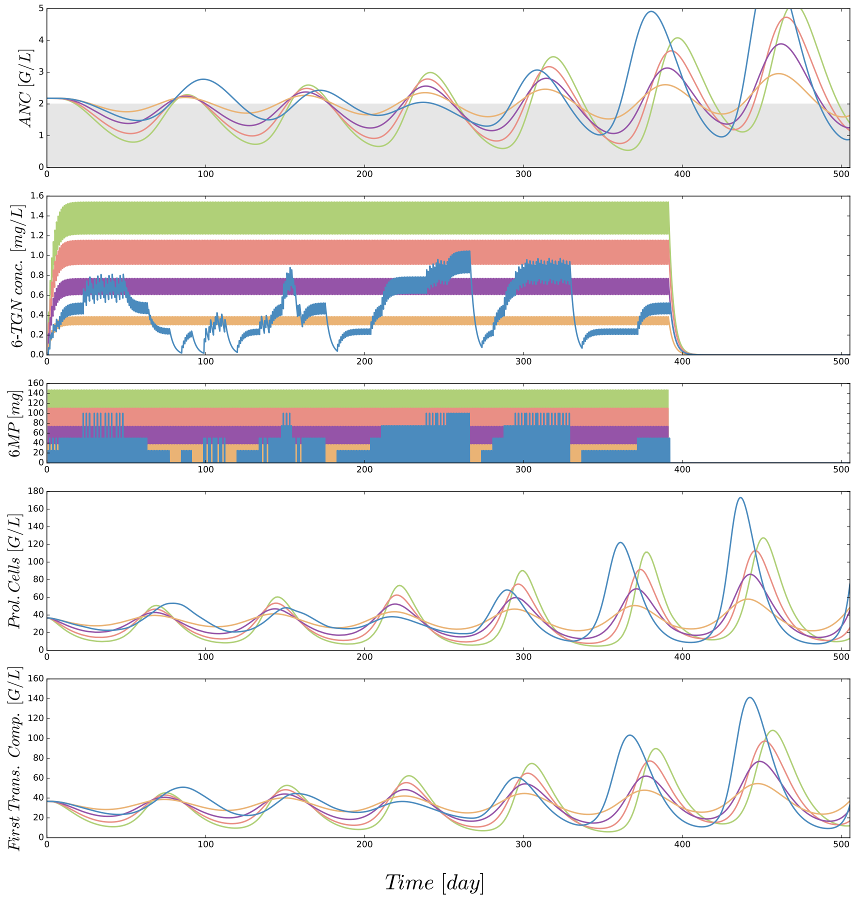

Figure 98: As Figure 2, but for another patient out of 116 patients.

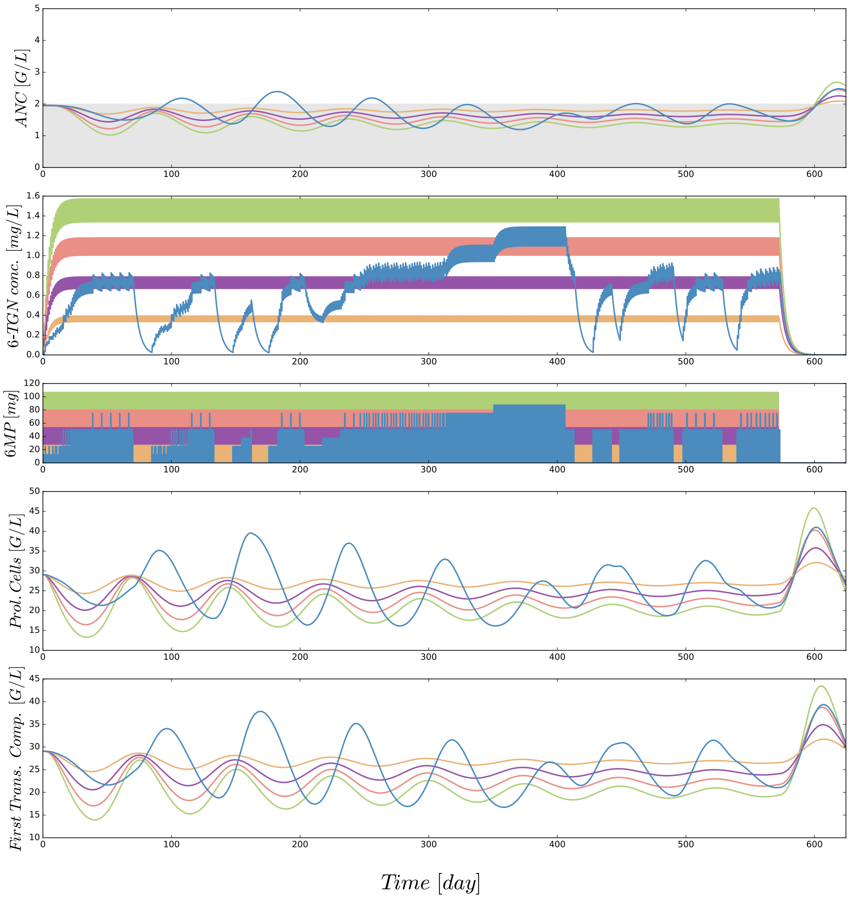

Figure 99: As Figure 2, but for another patient out of 116 patients.

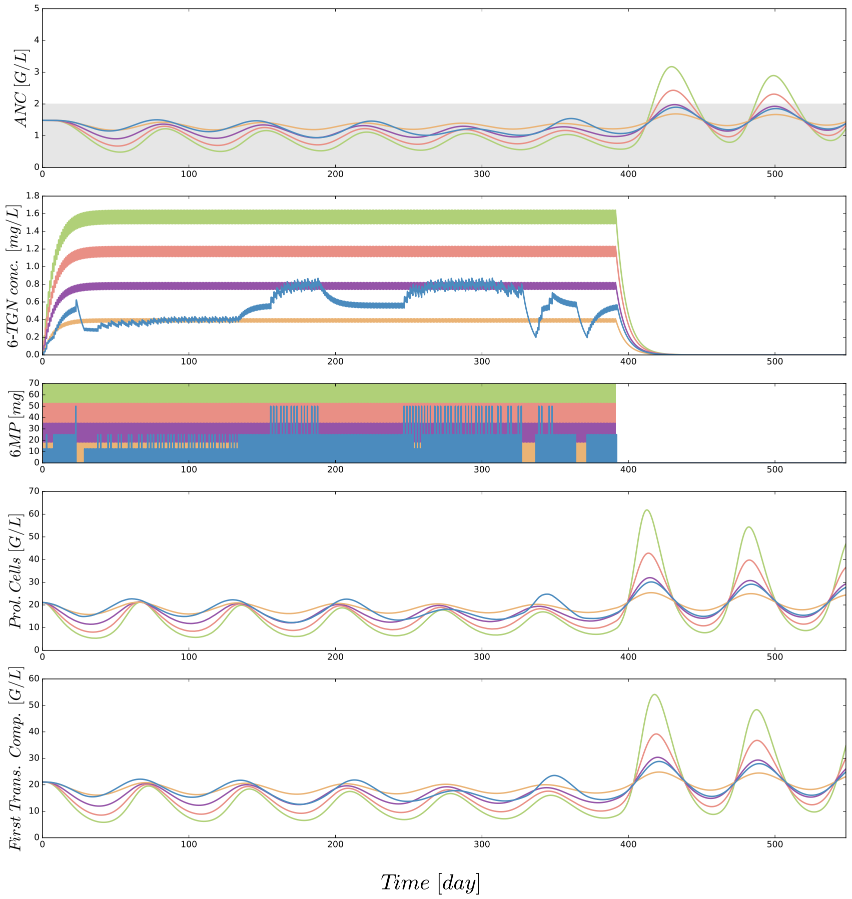

Figure 100: As Figure 2, but for another patient out of 116 patients.

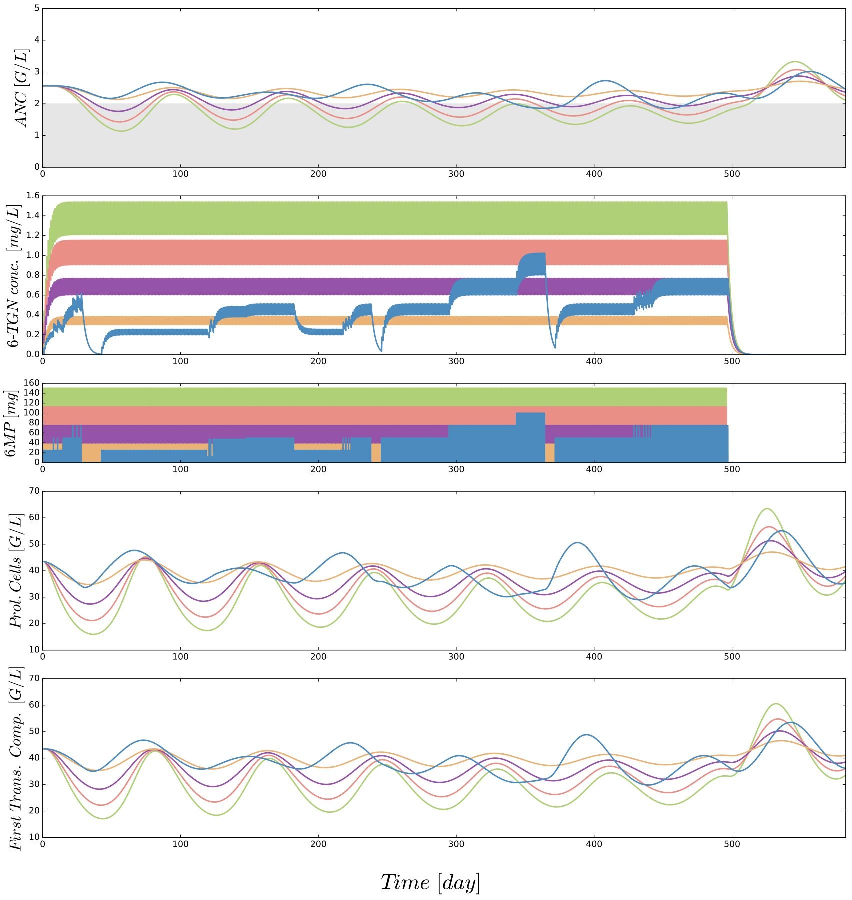

Figure 101: As Figure 2, but for another patient out of 116 patients.

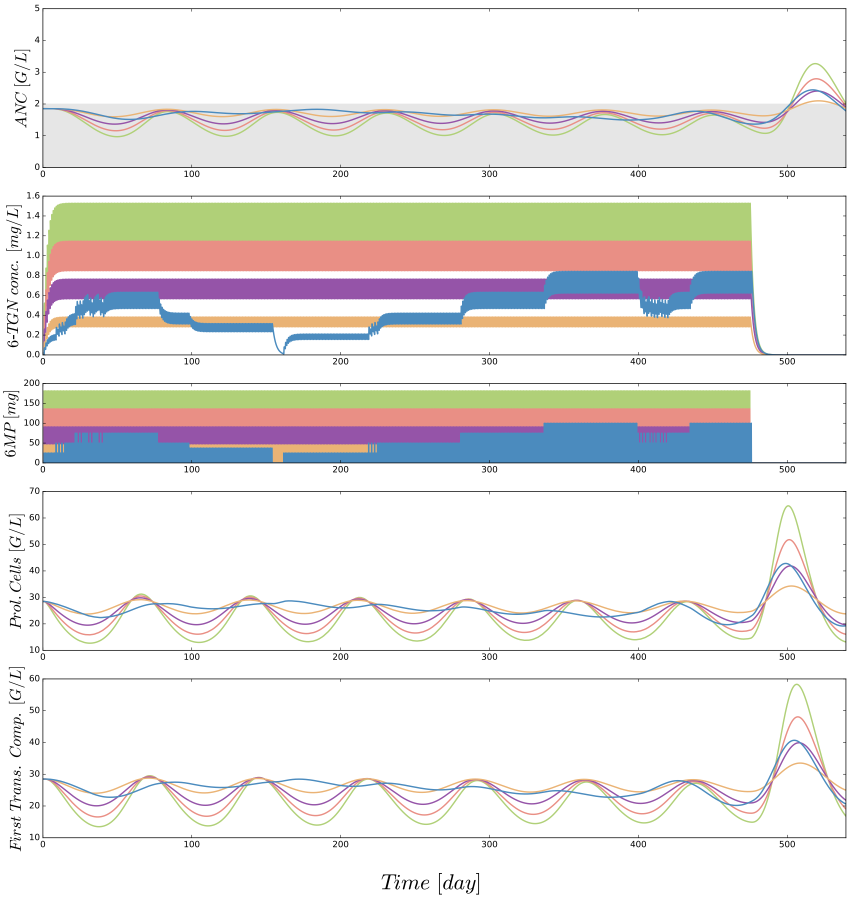

Figure 102: As Figure 2, but for another patient out of 116 patients.

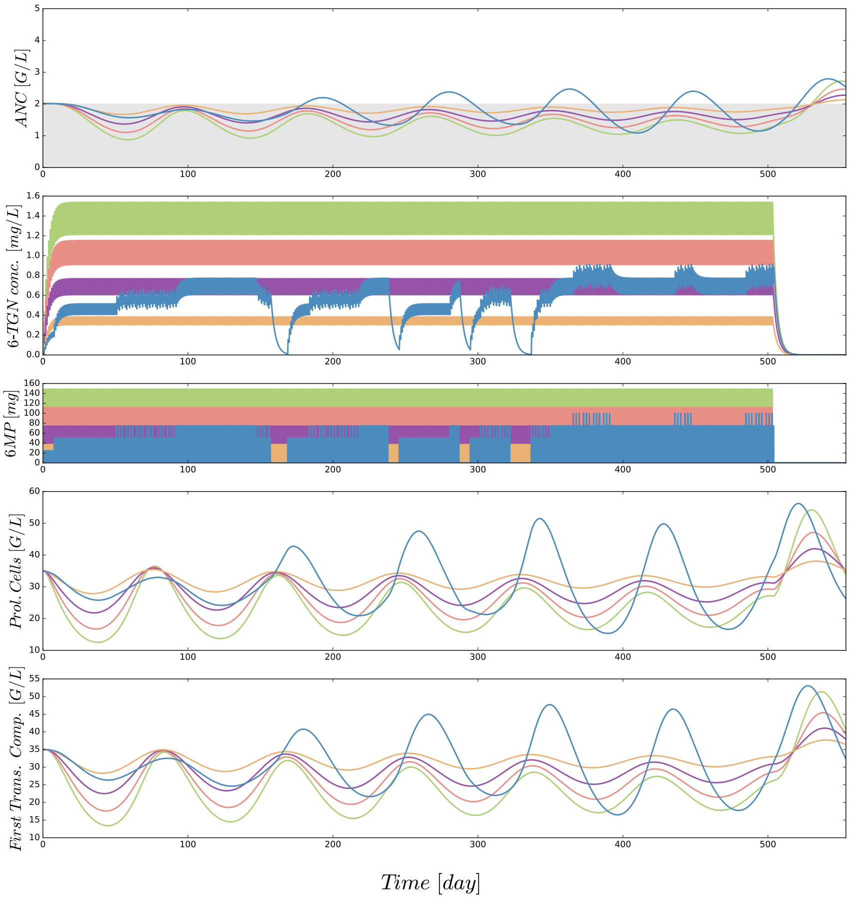

Figure 103: As Figure 2, but for another patient out of 116 patients.

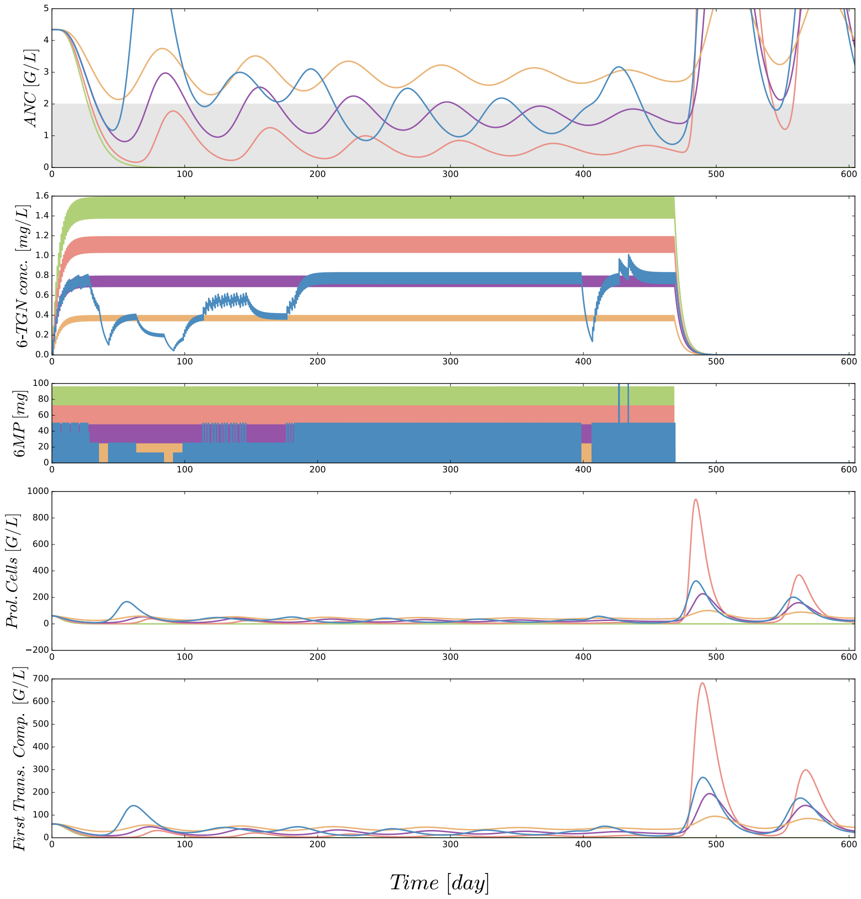

Figure 104: As Figure 2, but for another patient out of 116 patients.

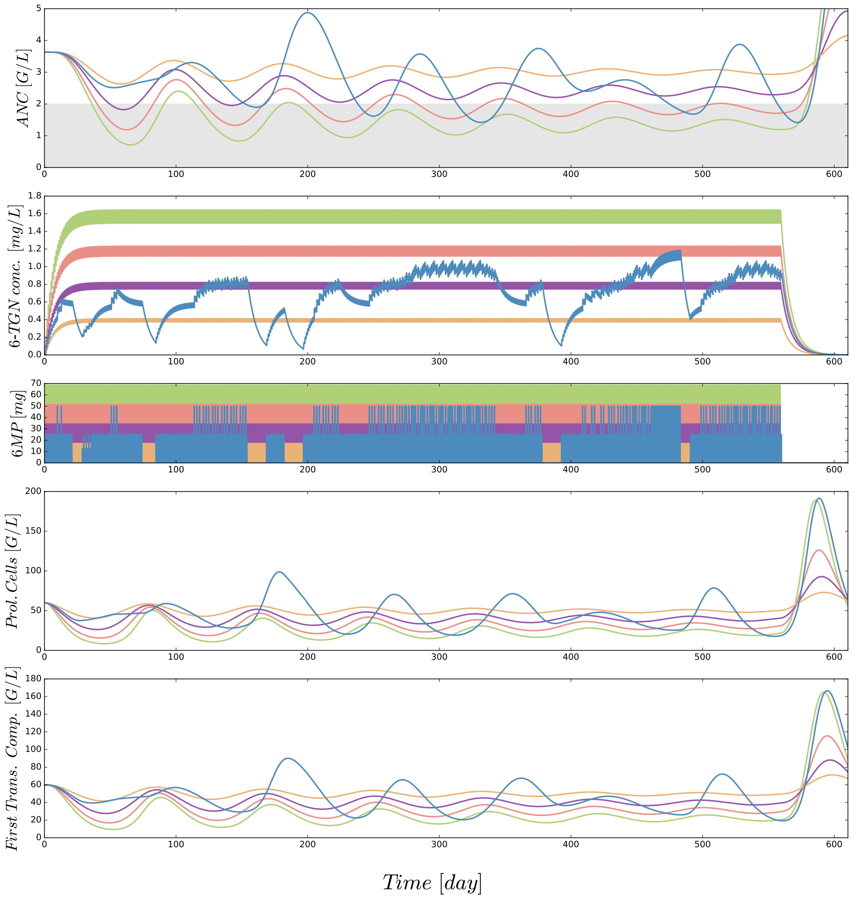

Figure 105: As Figure 2, but for another patient out of 116 patients.

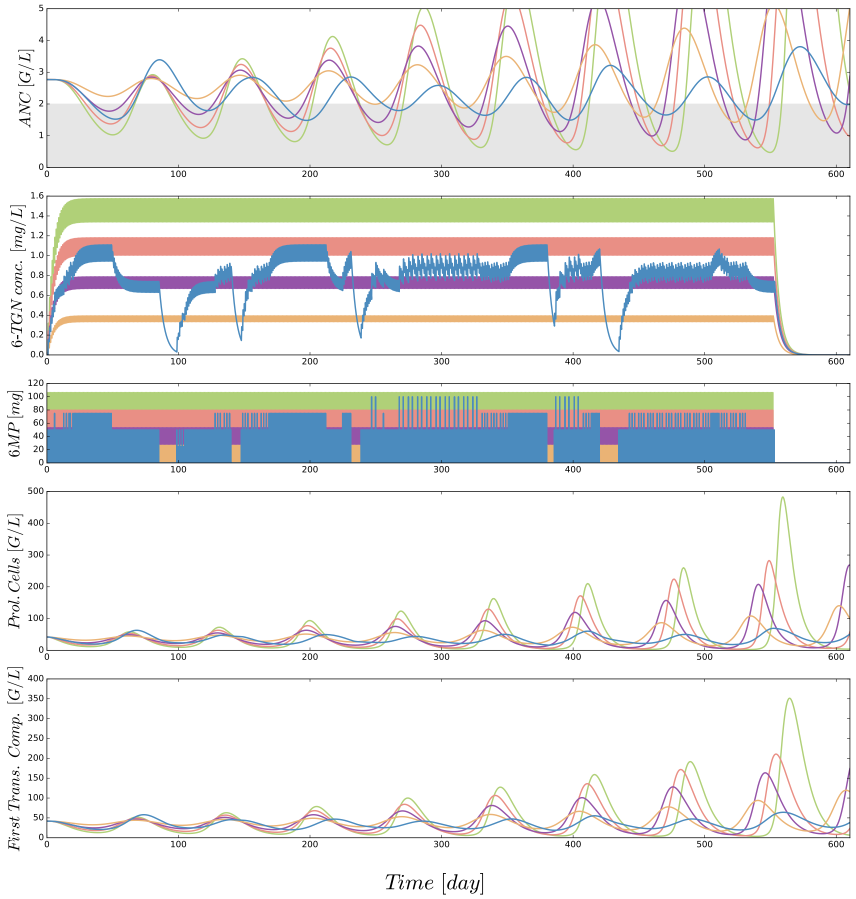

Figure 106: As Figure 2, but for another patient out of 116 patients.

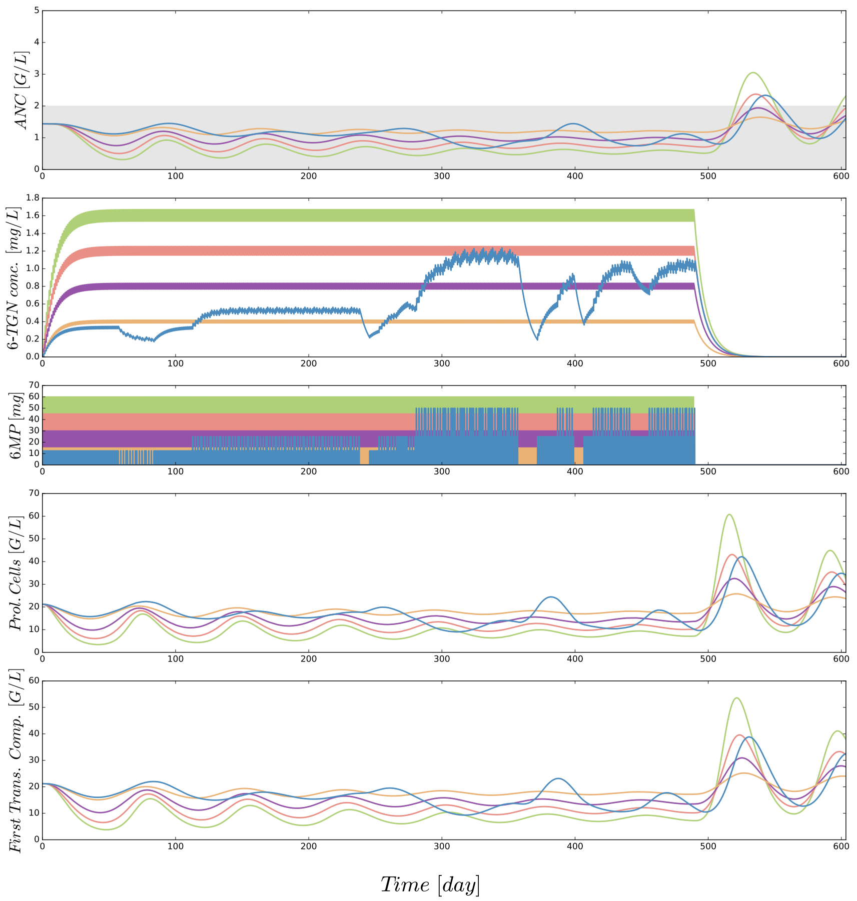

Figure 107: As Figure 2, but for another patient out of 116 patients.

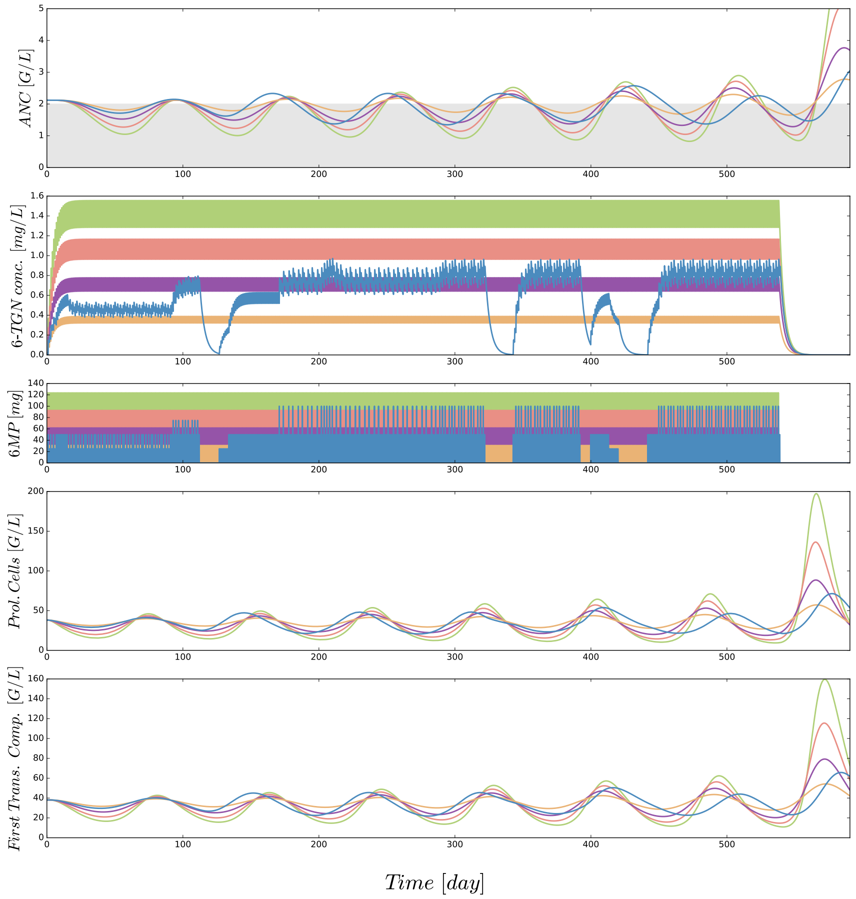

Figure 108: As Figure 2, but for another patient out of 116 patients.

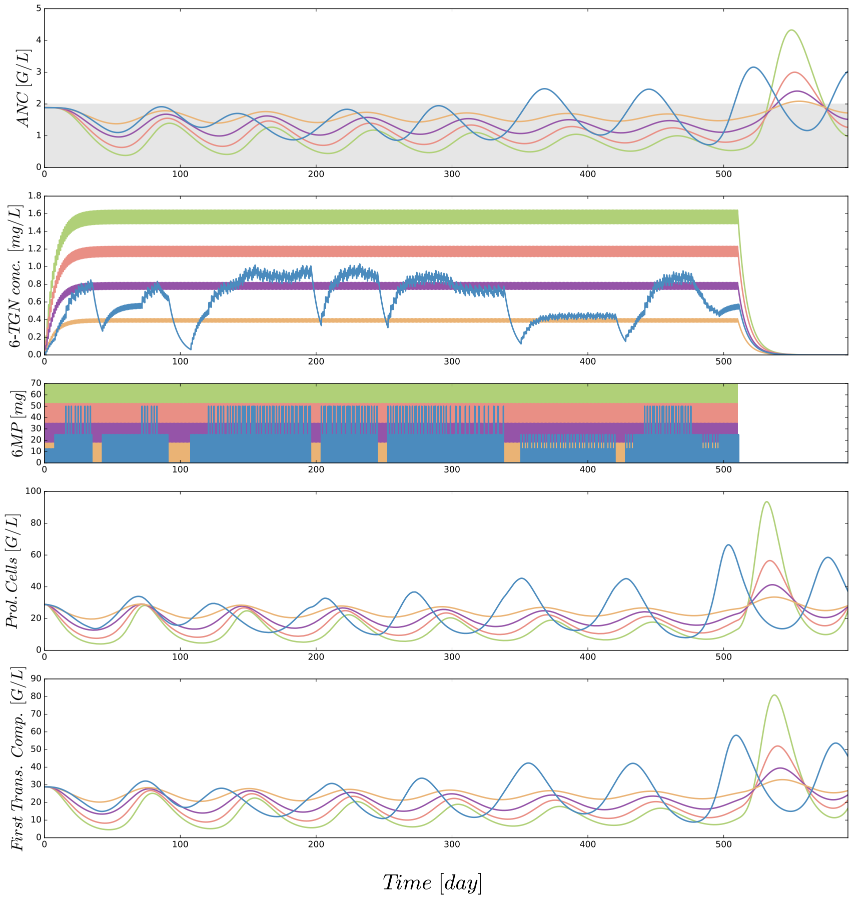

Figure 109: As Figure 2, but for another patient out of 116 patients.

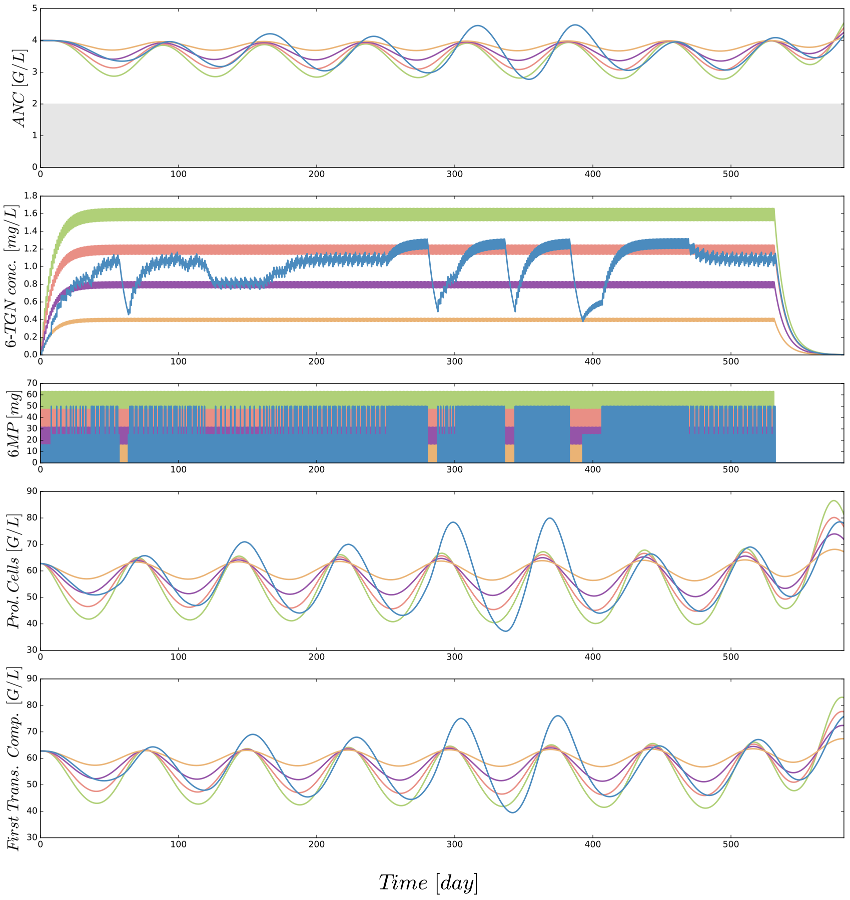

Figure 110: As Figure 2, but for another patient out of 116 patients.

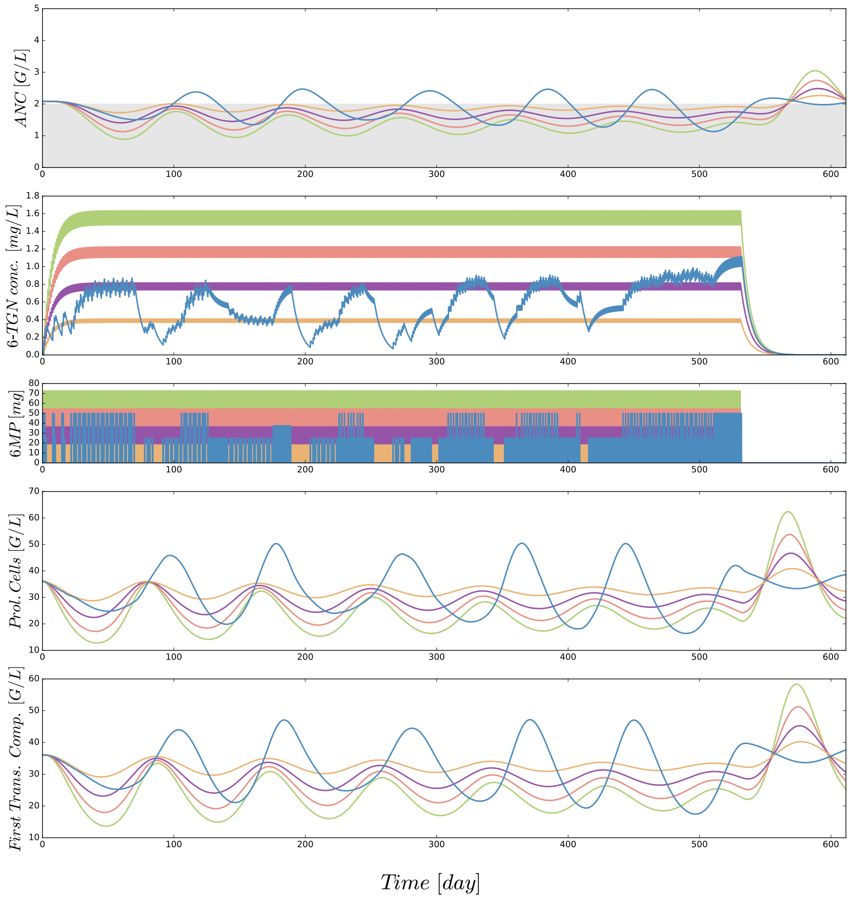

Figure 111: As Figure 2, but for another patient out of 116 patients.

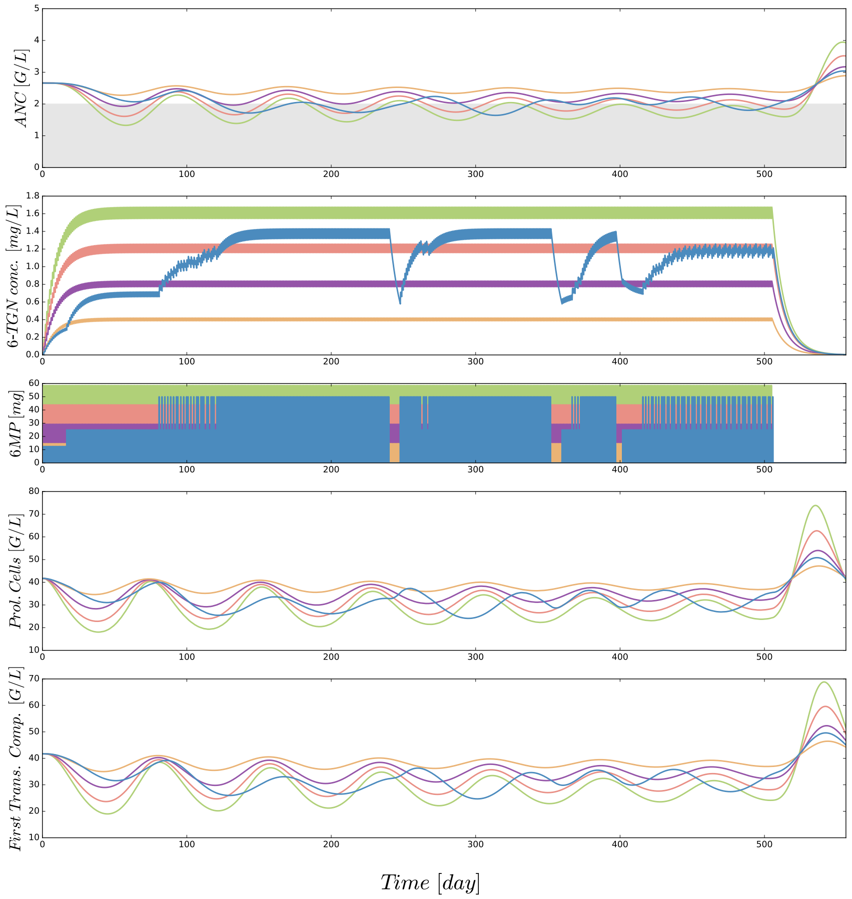

Figure 112: As Figure 2, but for another patient out of 116 patients.

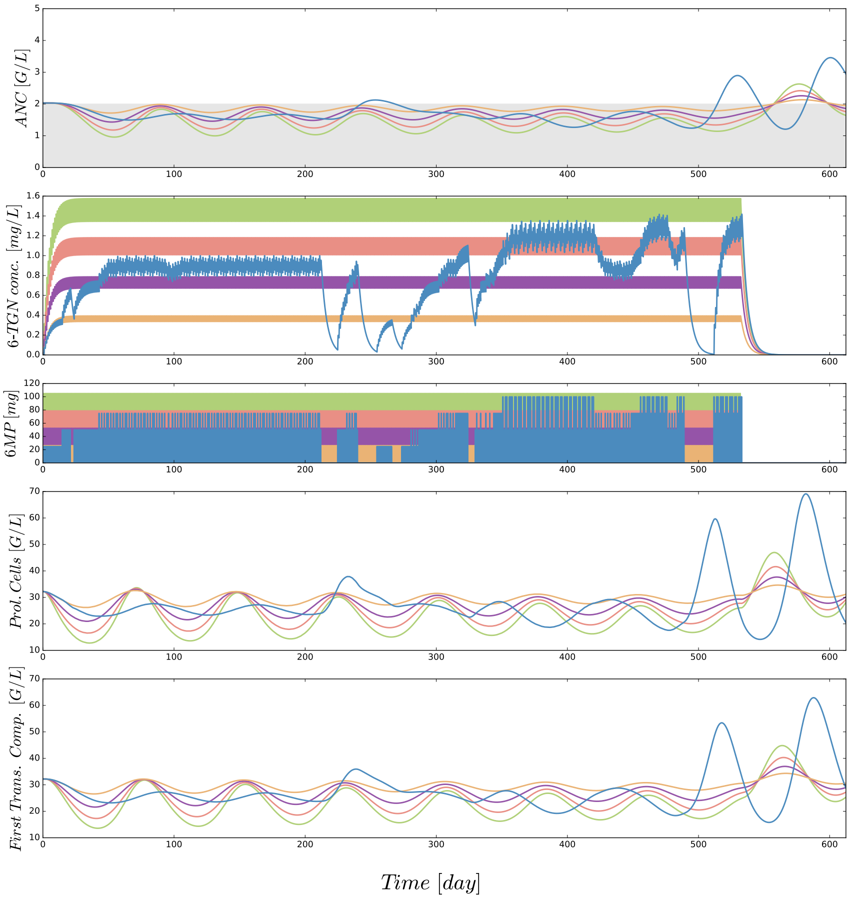

Figure 113: As Figure 2, but for another patient out of 116 patients.

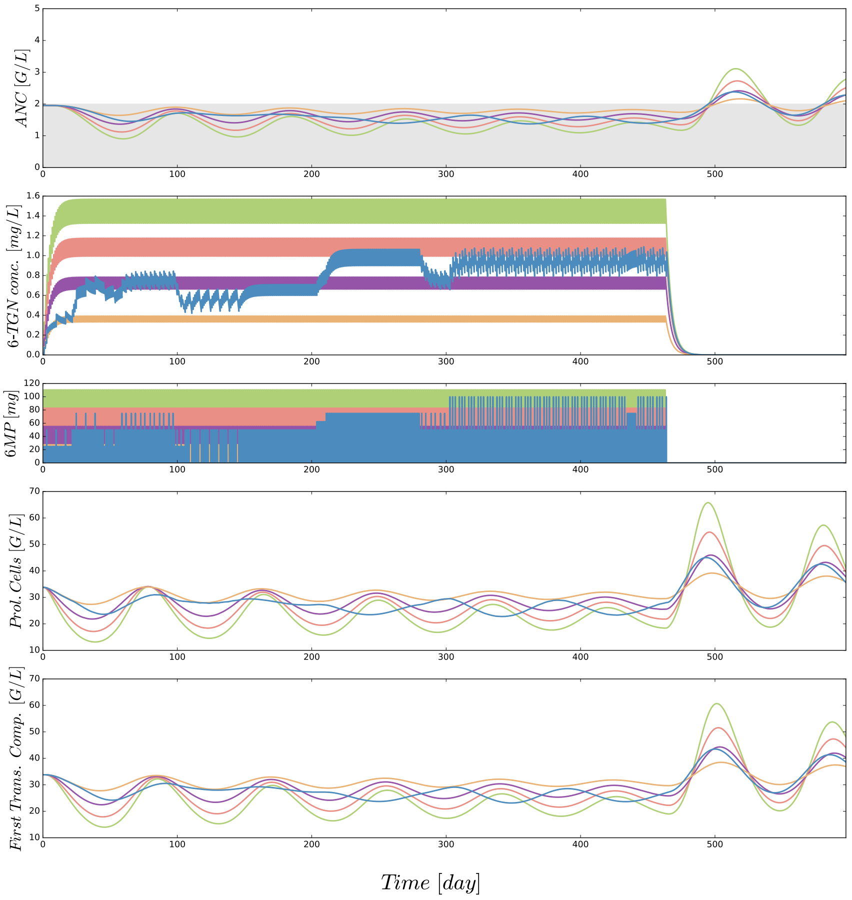

Figure 114: As Figure 2, but for another patient out of 116 patients.

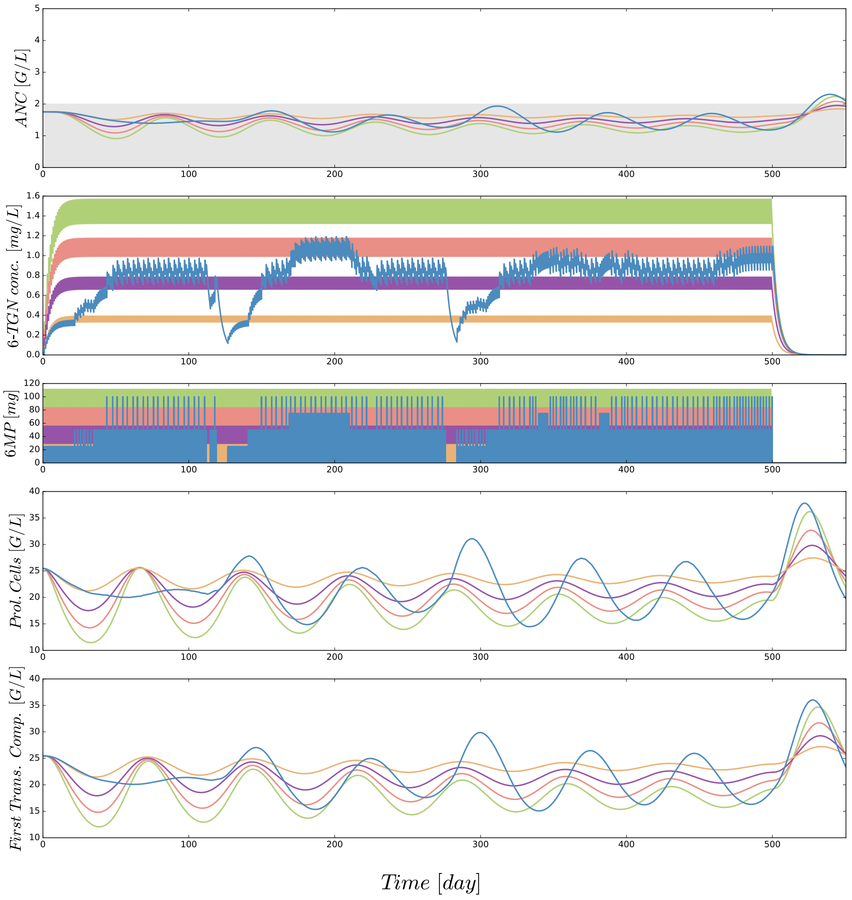

Figure 115: As Figure 2, but for another patient out of 116 patients.

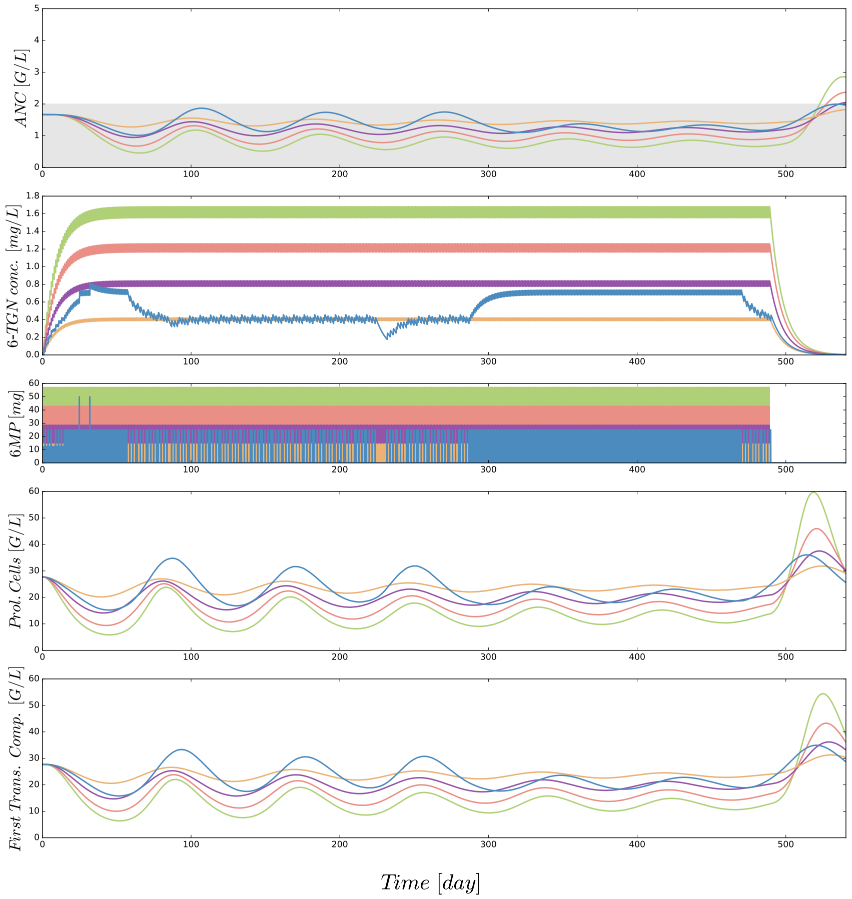

Figure 116: As Figure 2, but for another patient out of 116 patients.

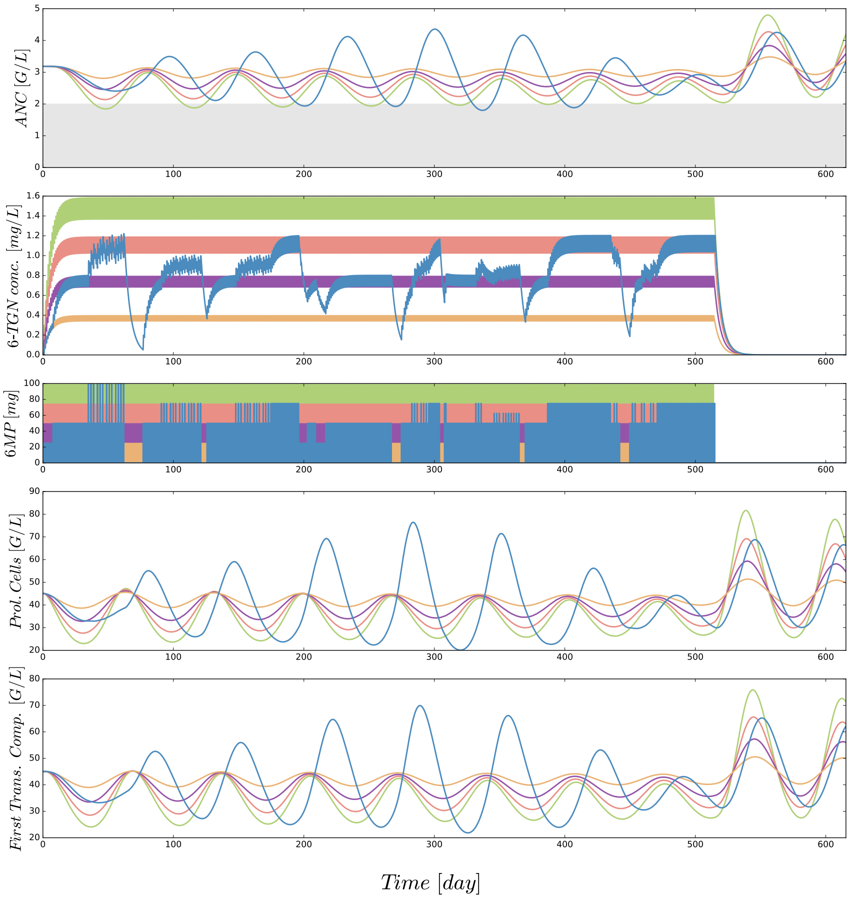

Figure 117: As Figure 2, but for another patient out of 116 patients.
