# Supplementary material for: Model-Based Simulation of Maintenance Therapy of Childhood Acute Lymphoblastic Leukemia
Source: Front Physiol. 2020 Mar 18;11:217. doi: 10.3389/fphys.2020.00217 (PMC7093595; doi:10.3389/fphys.2020.00217)
Supplement: Supplementary file 4 [file Data_Sheet_4.PDF]

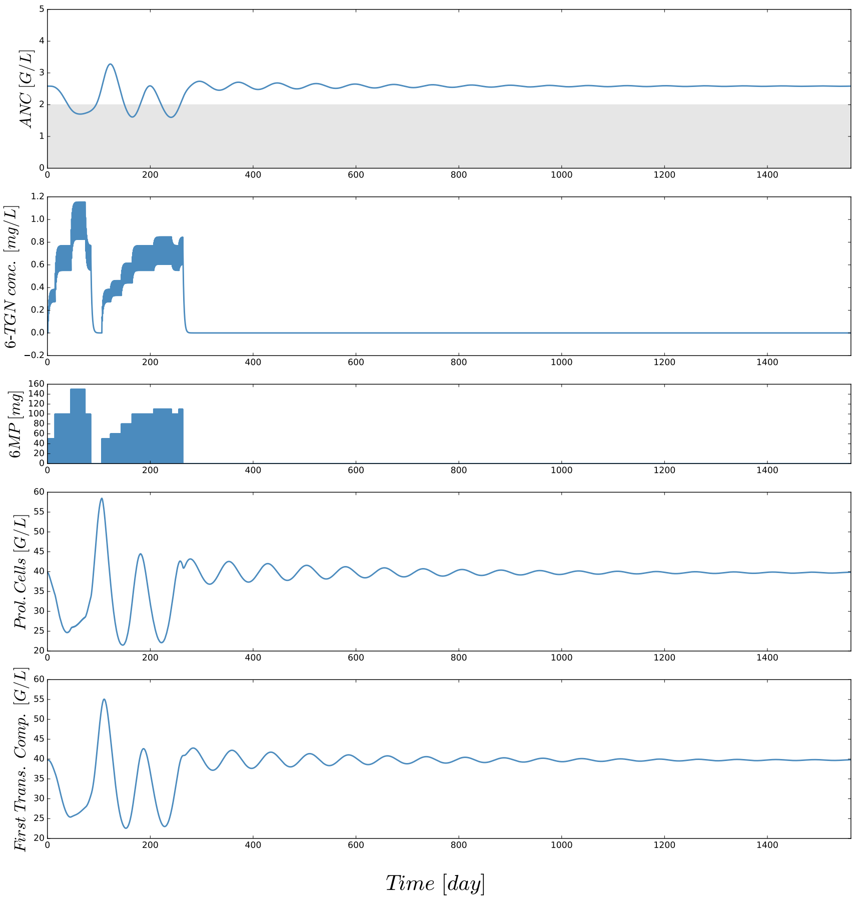

Figure 1: Simulated trajectory of the first patient for protocol 2 from Table 2 with a longer time horizon to determine the steady state behavior after the end of the maintenance therapy. The color of the trajectory is identical to the one used in Figure 4.

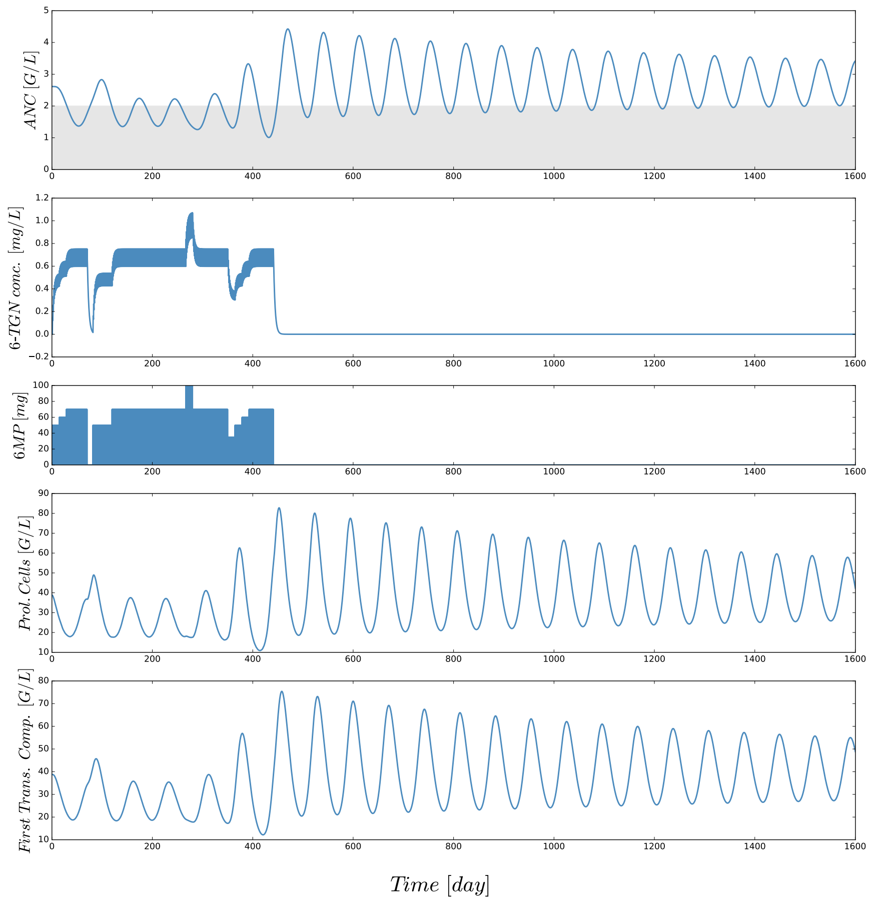

Figure 2: As Figure 1, but for another patient out of 116 patients.

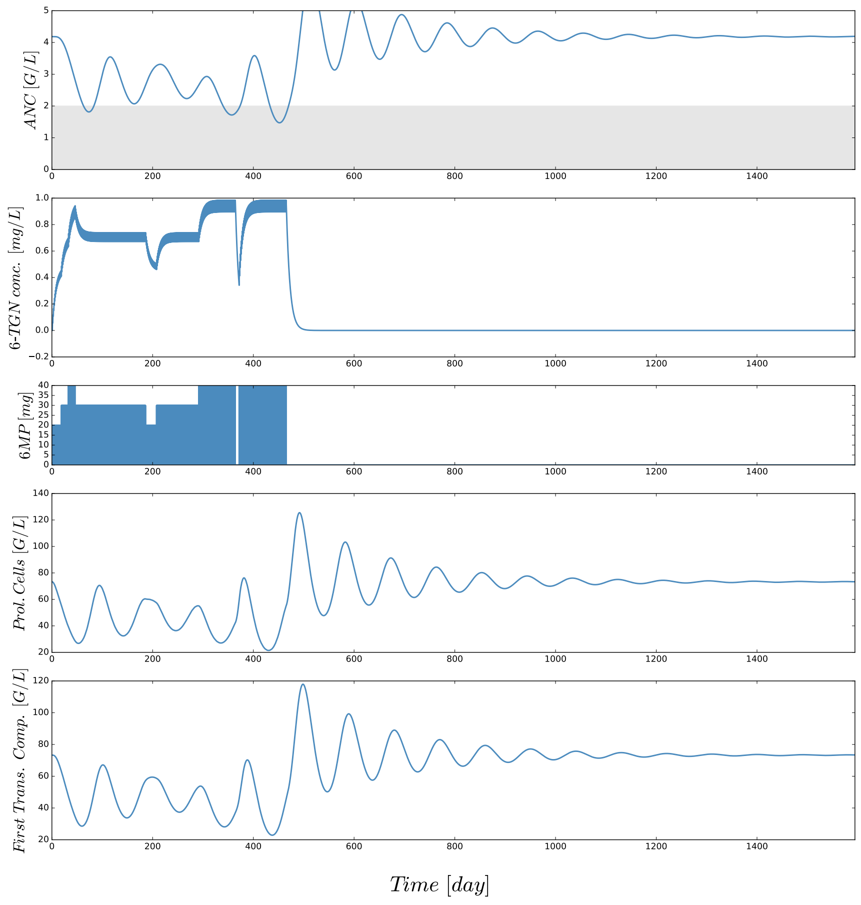

Figure 3: As Figure 1, but for another patient out of 116 patients.

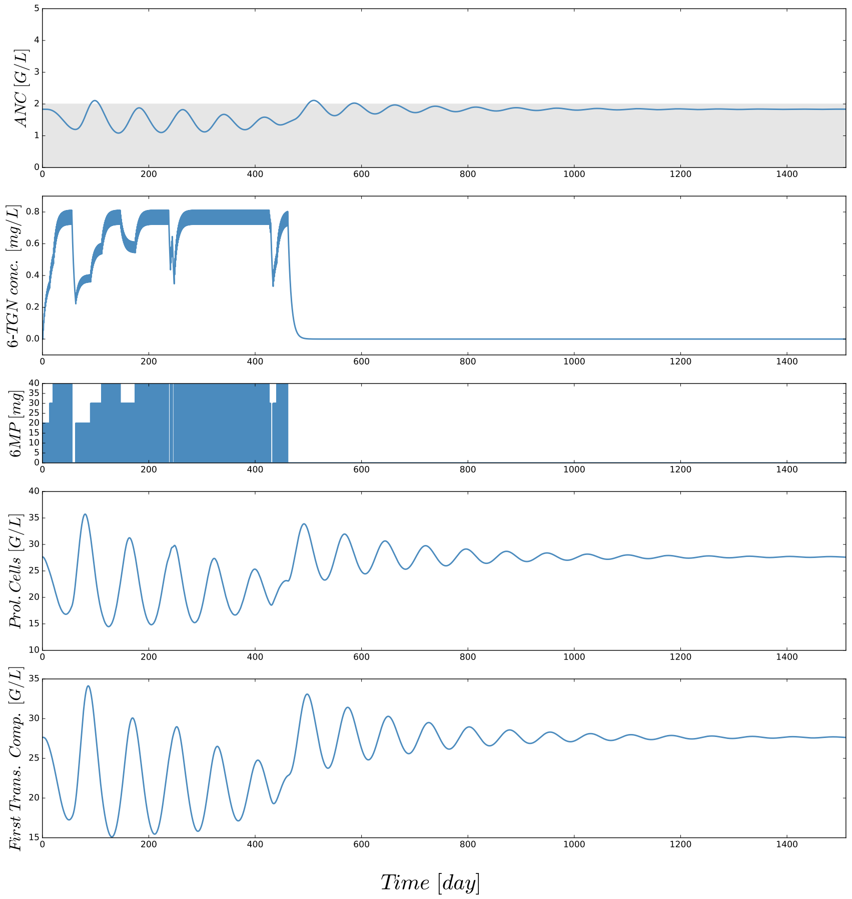

Figure 4: As Figure 1, but for another patient out of 116 patients.

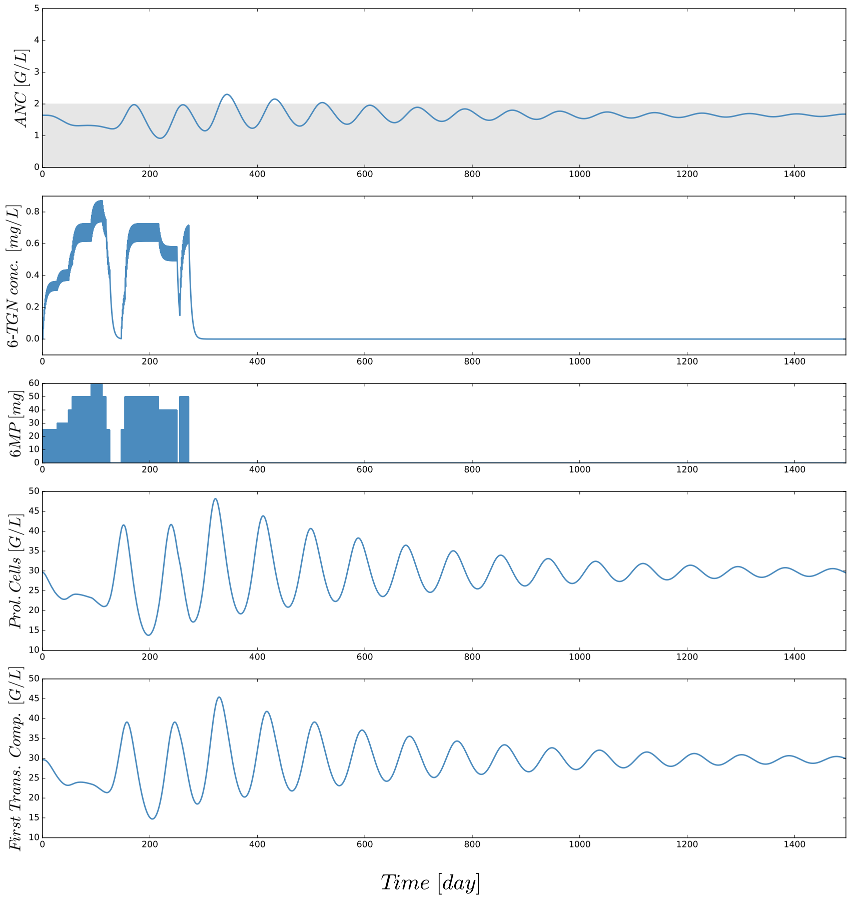

Figure 5: As Figure 1, but for another patient out of 116 patients.

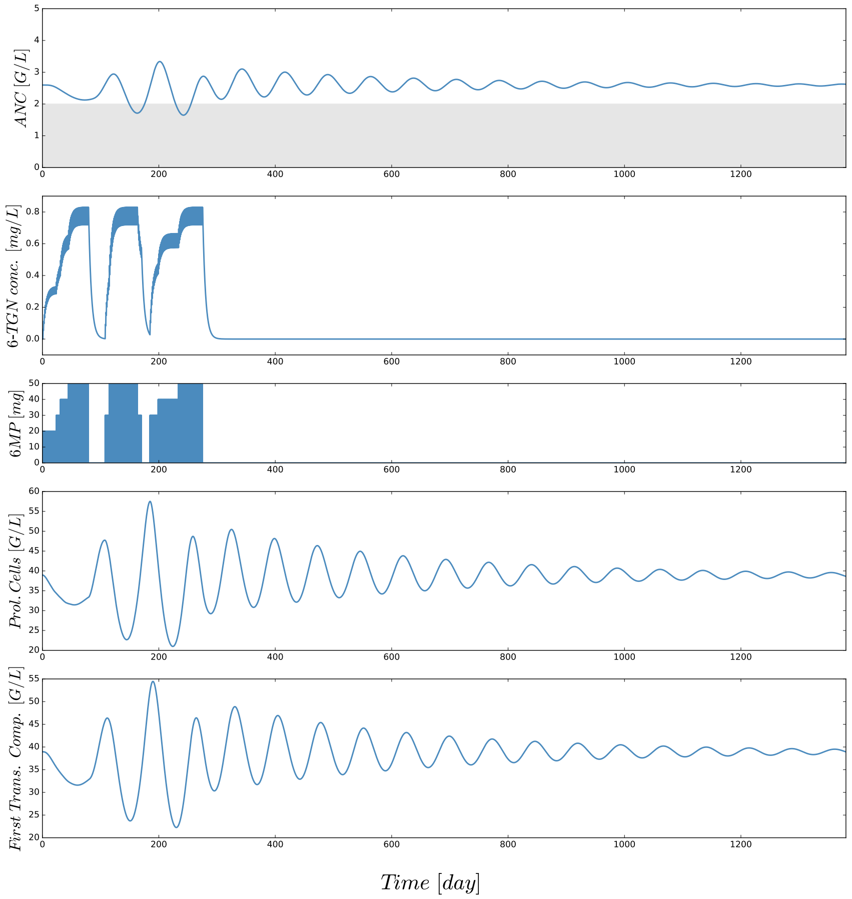

Figure 6: As Figure 1, but for another patient out of 116 patients.

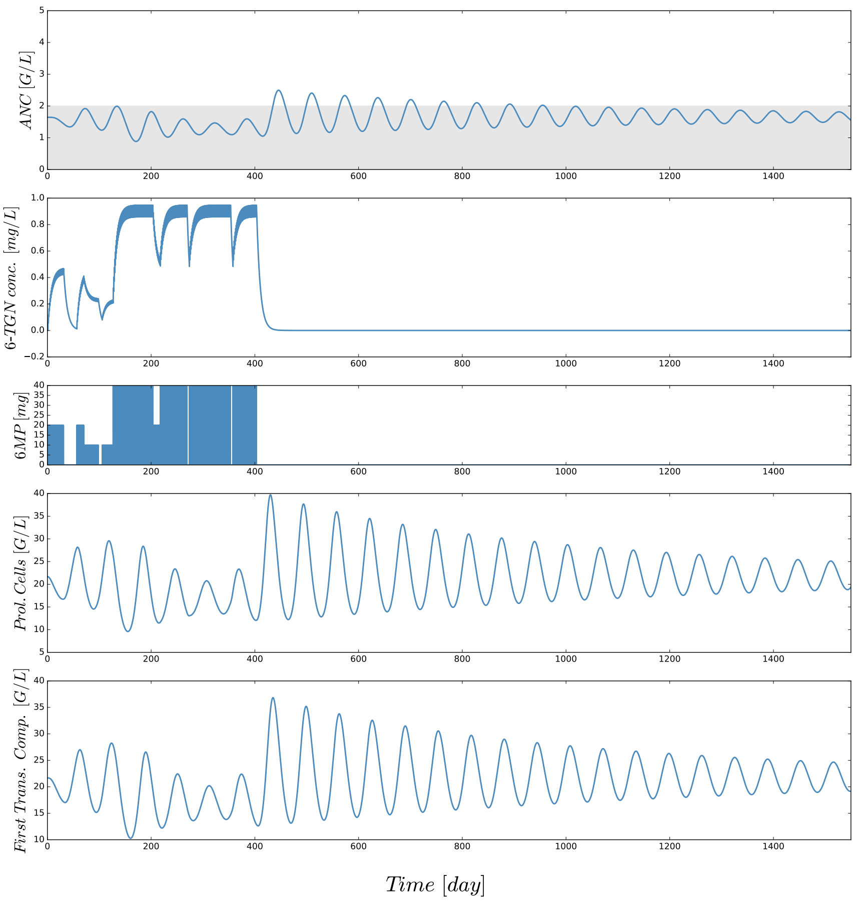

Figure 7: As Figure 1, but for another patient out of 116 patients.

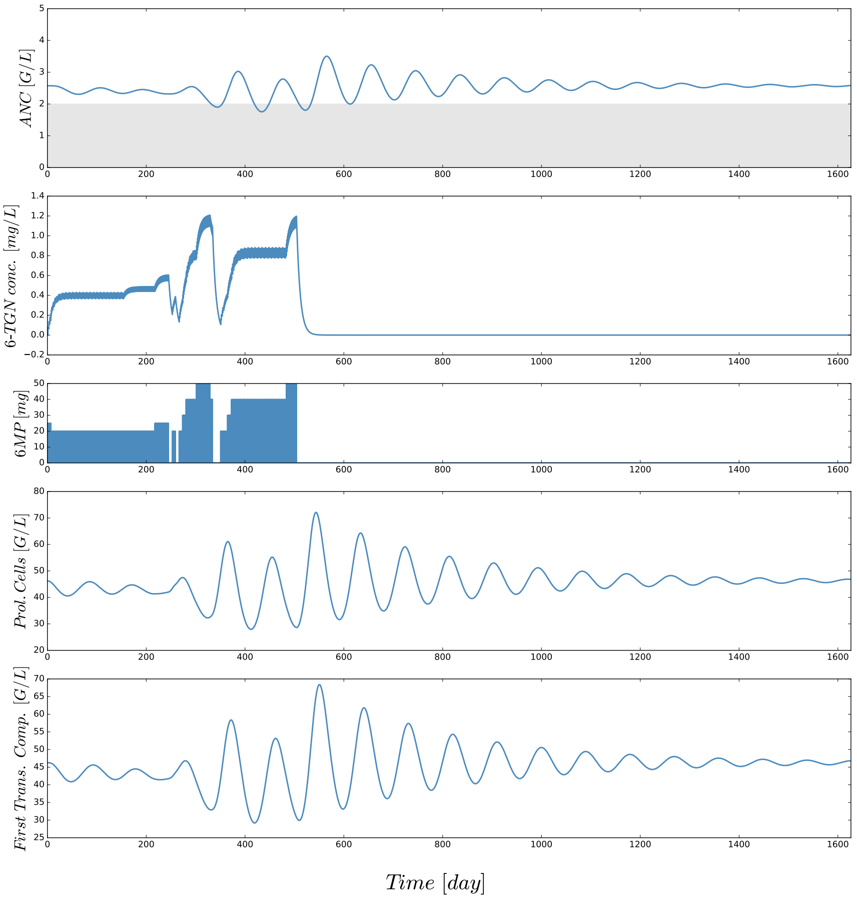

Figure 8: As Figure 1, but for another patient out of 116 patients.

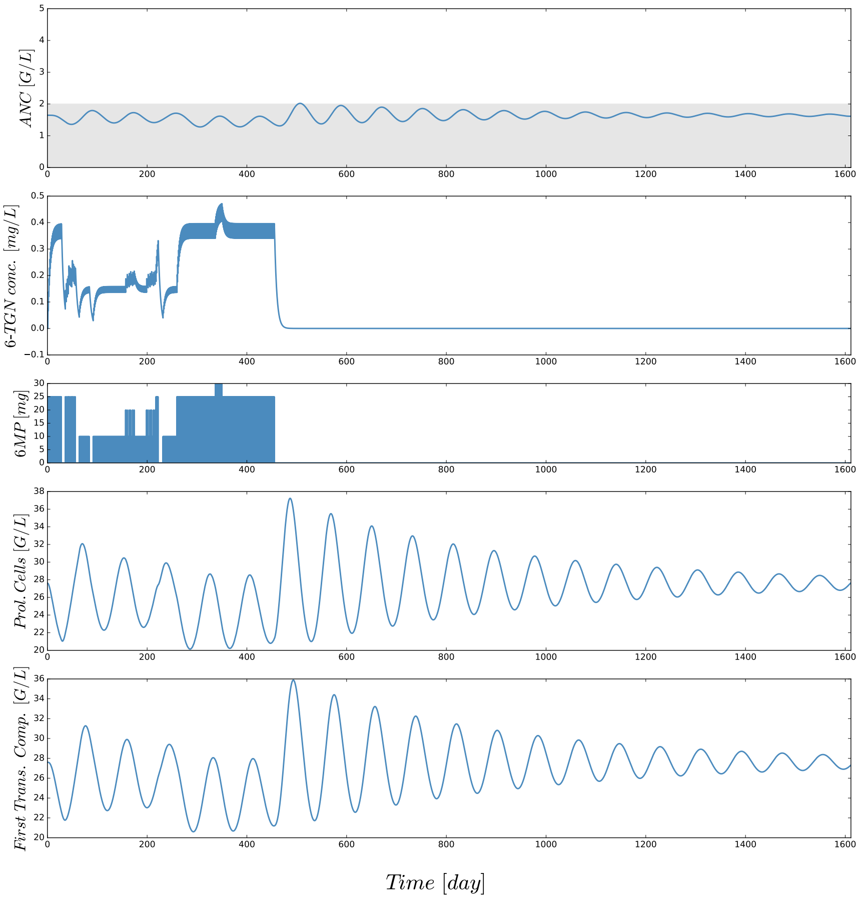

Figure 9: As Figure 1, but for another patient out of 116 patients.

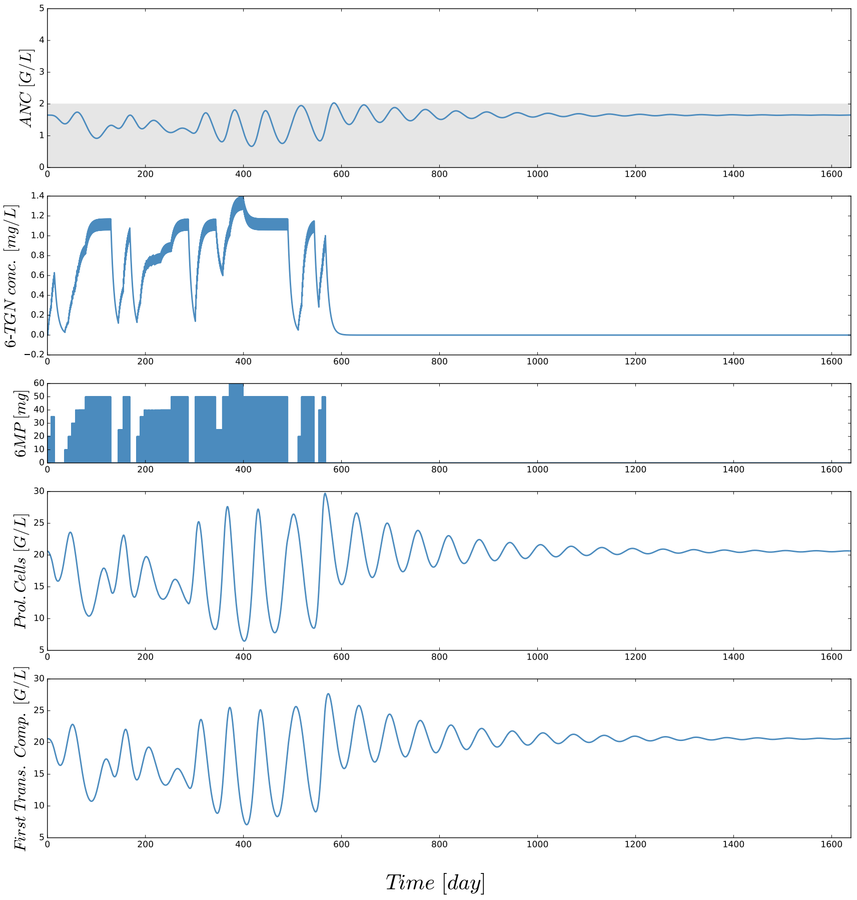

Figure 10: As Figure 1, but for another patient out of 116 patients.

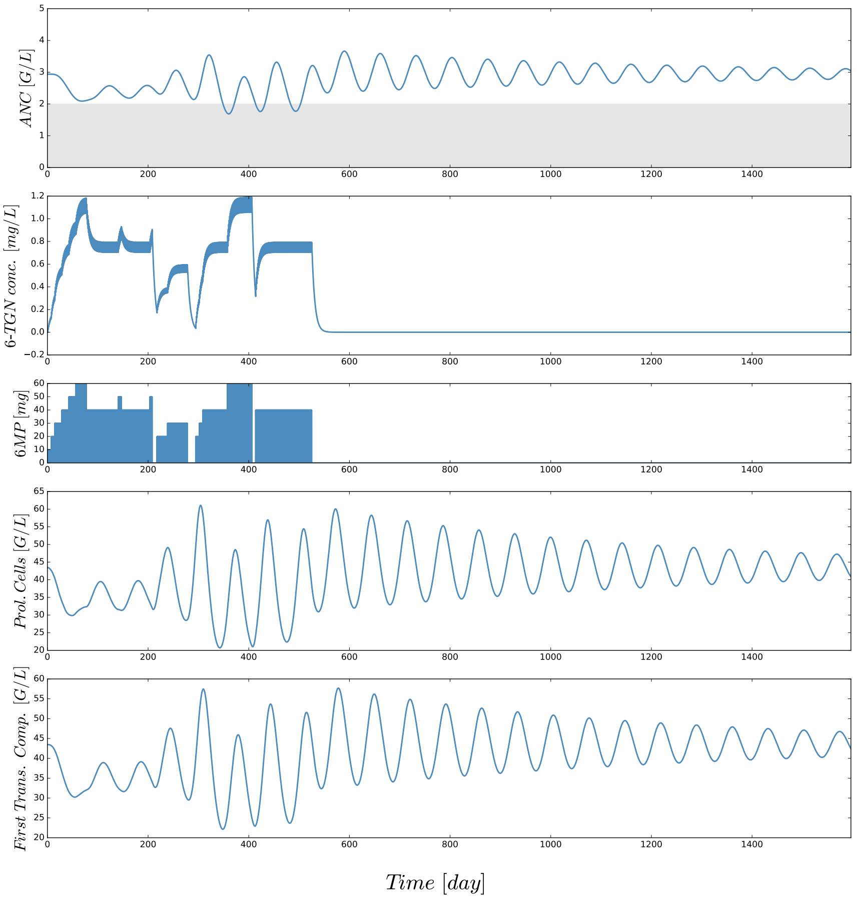

Figure 11: As Figure 1, but for another patient out of 116 patients.

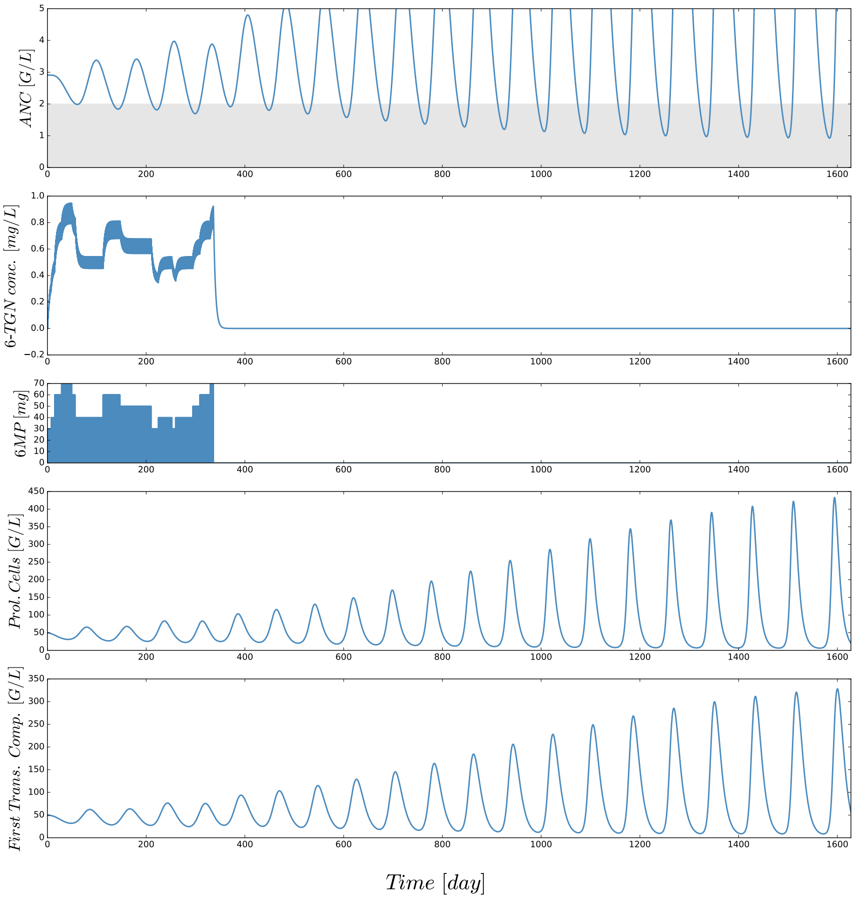

Figure 12: As Figure 1, but for another patient out of 116 patients.

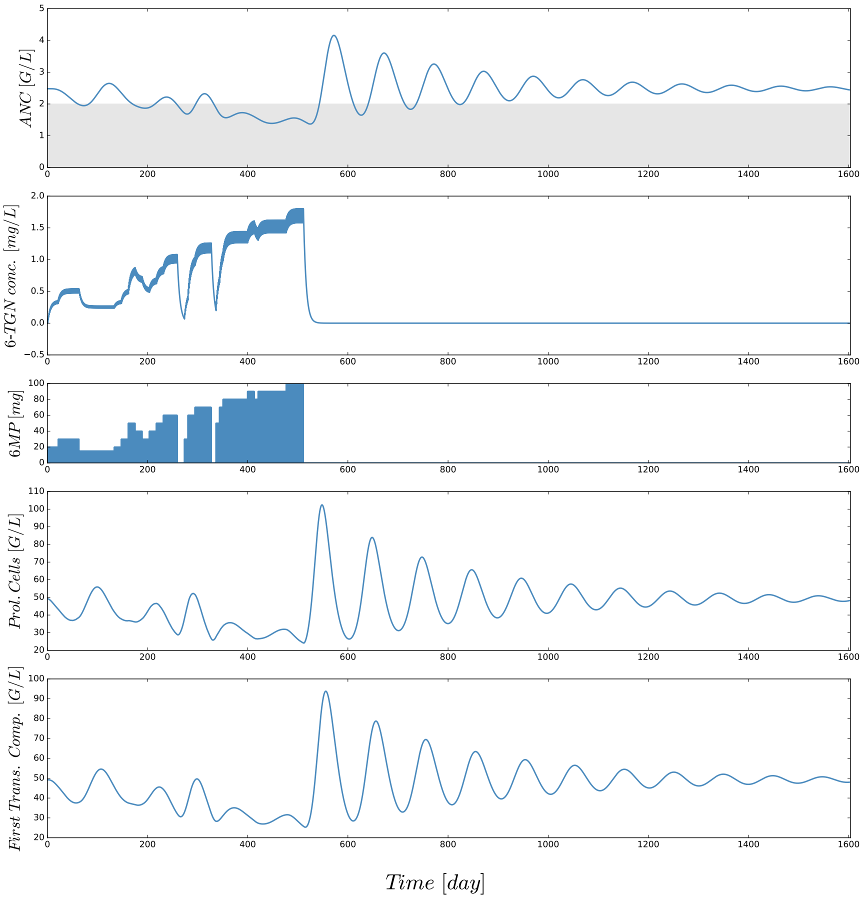

Figure 13: As Figure 1, but for another patient out of 116 patients.

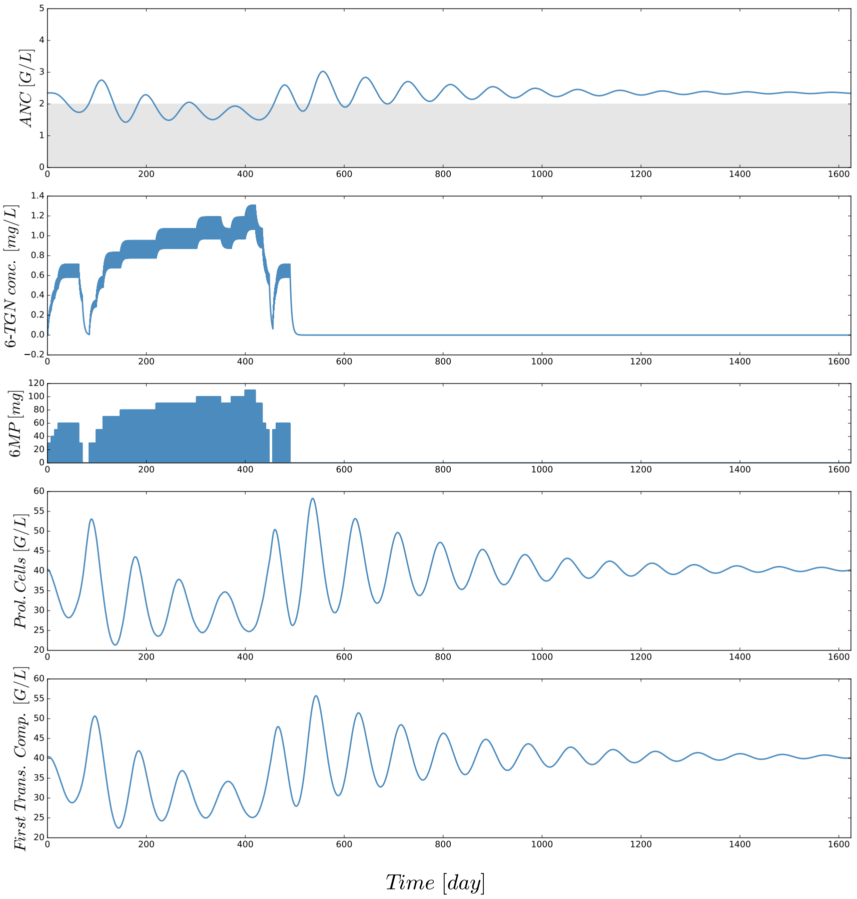

Figure 14: As Figure 1, but for another patient out of 116 patients.

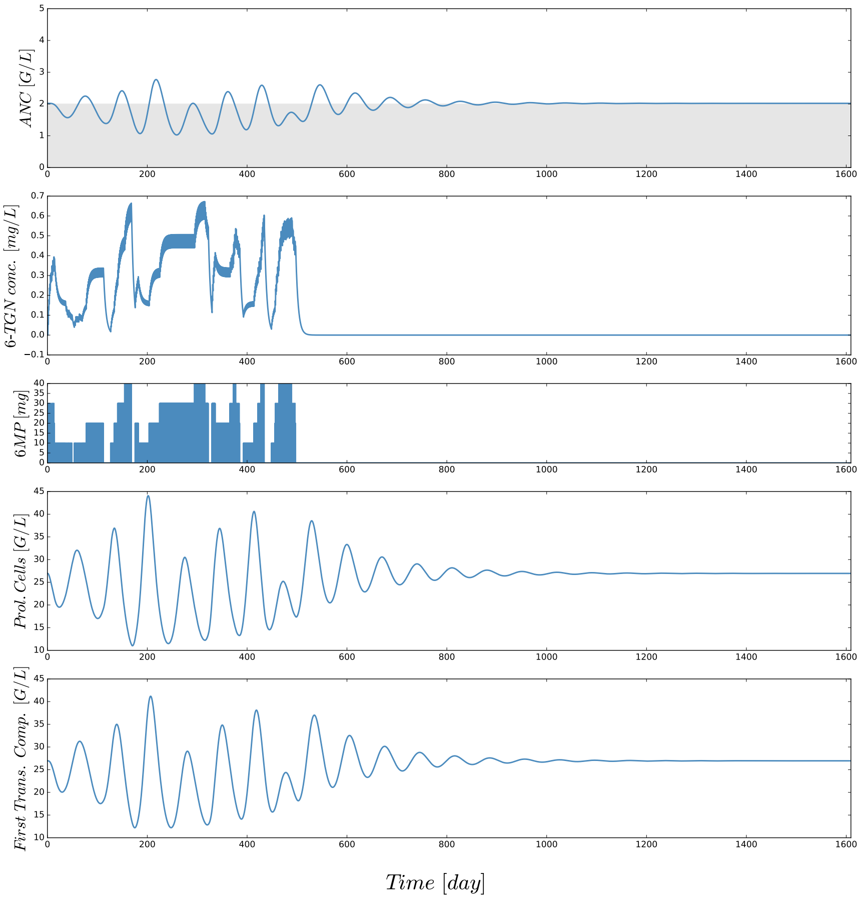

Figure 15: As Figure 1, but for another patient out of 116 patients.

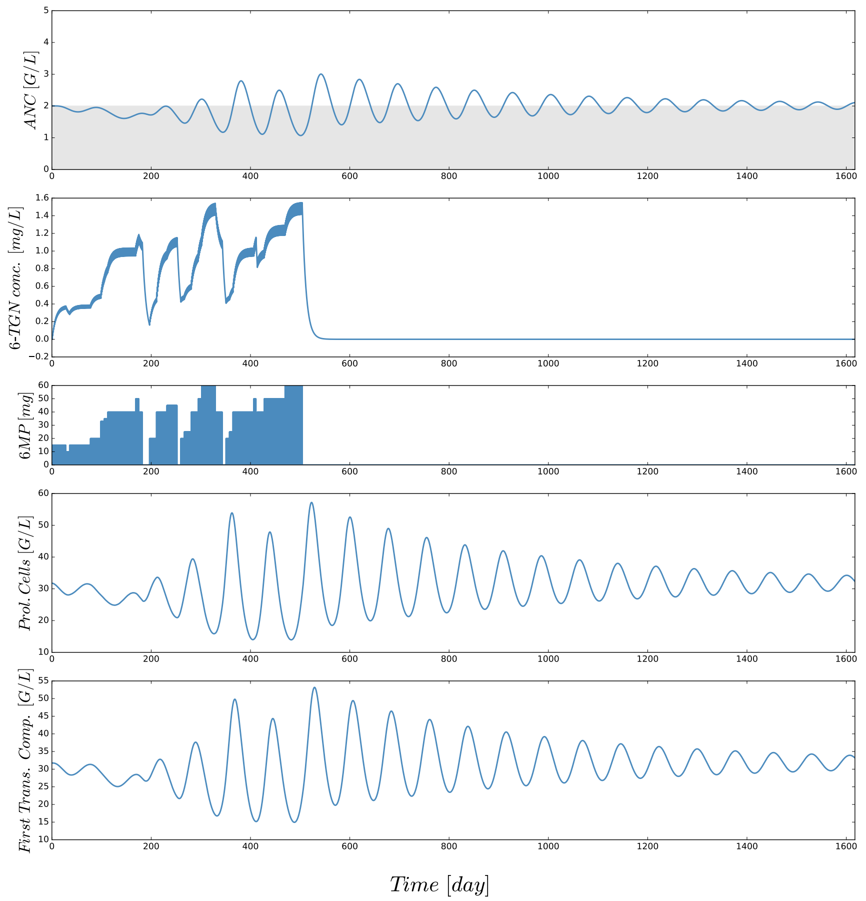

Figure 16: As Figure 1, but for another patient out of 116 patients.

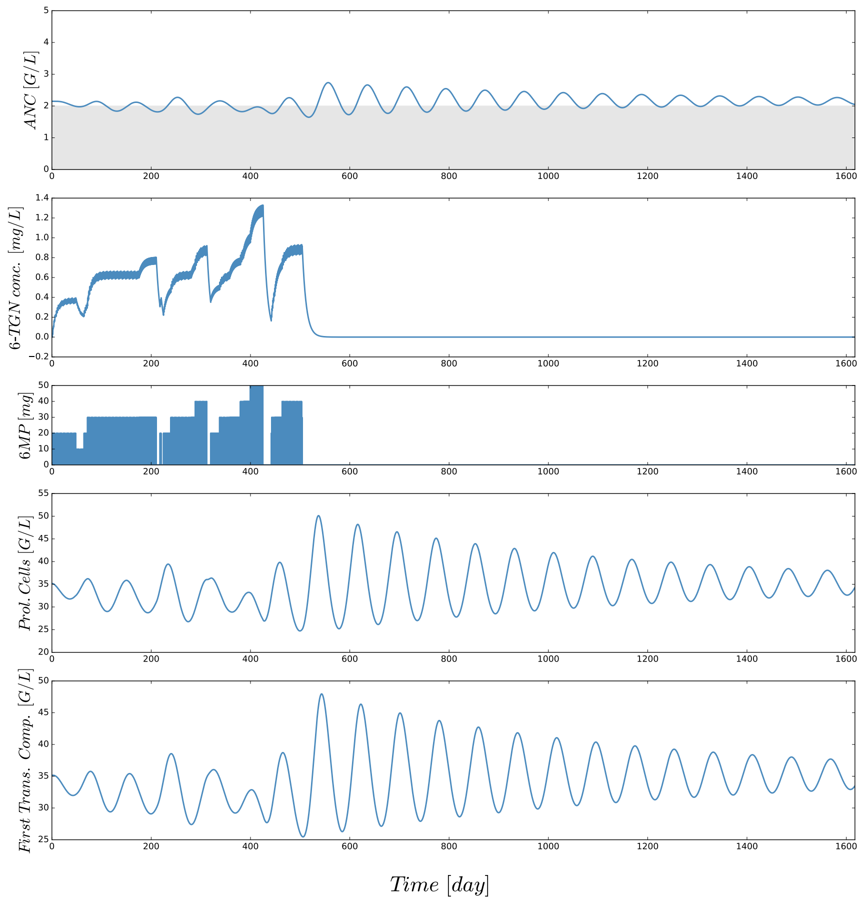

Figure 17: As Figure 1, but for another patient out of 116 patients.

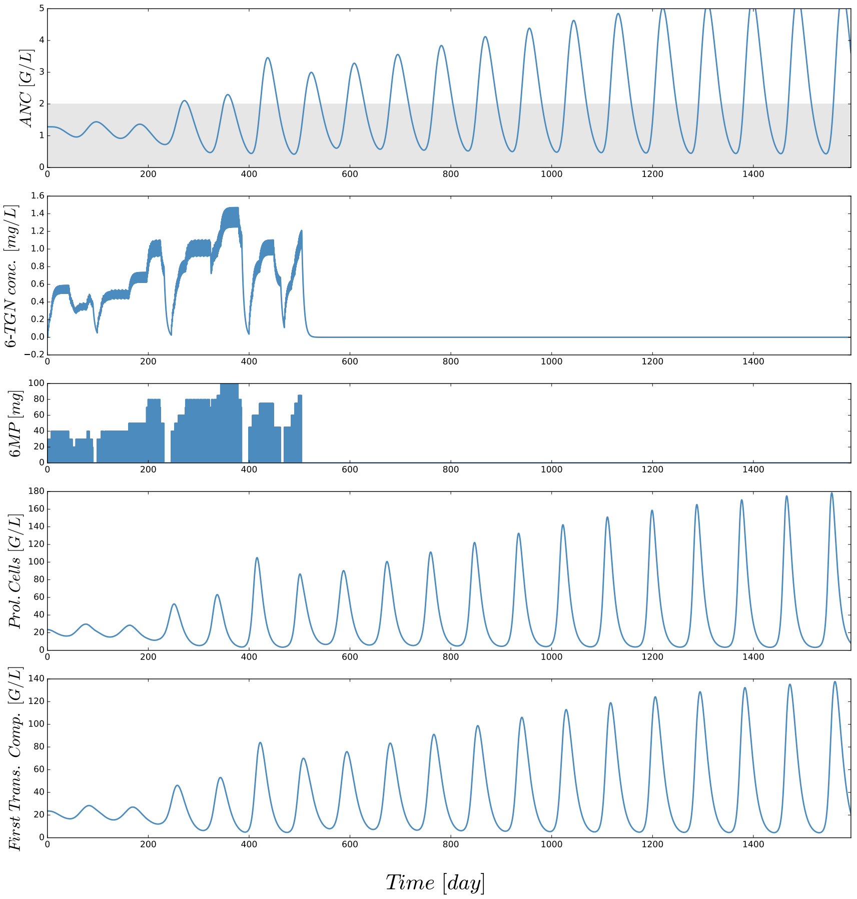

Figure 18: As Figure 1, but for another patient out of 116 patients.

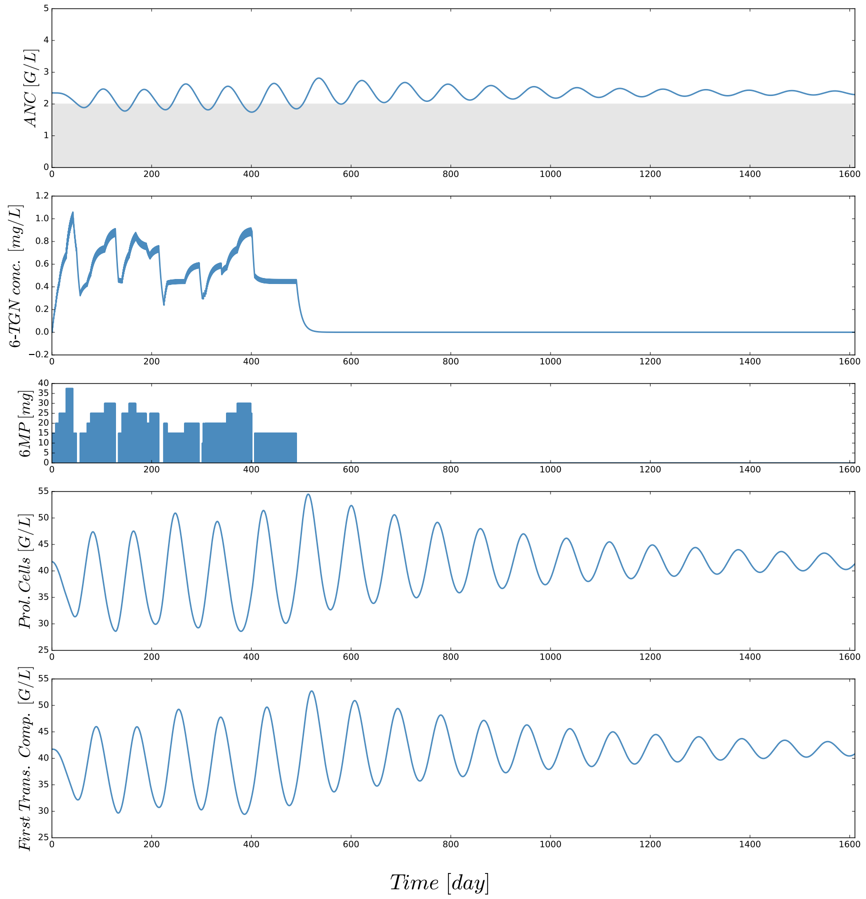

Figure 19: As Figure 1, but for another patient out of 116 patients.

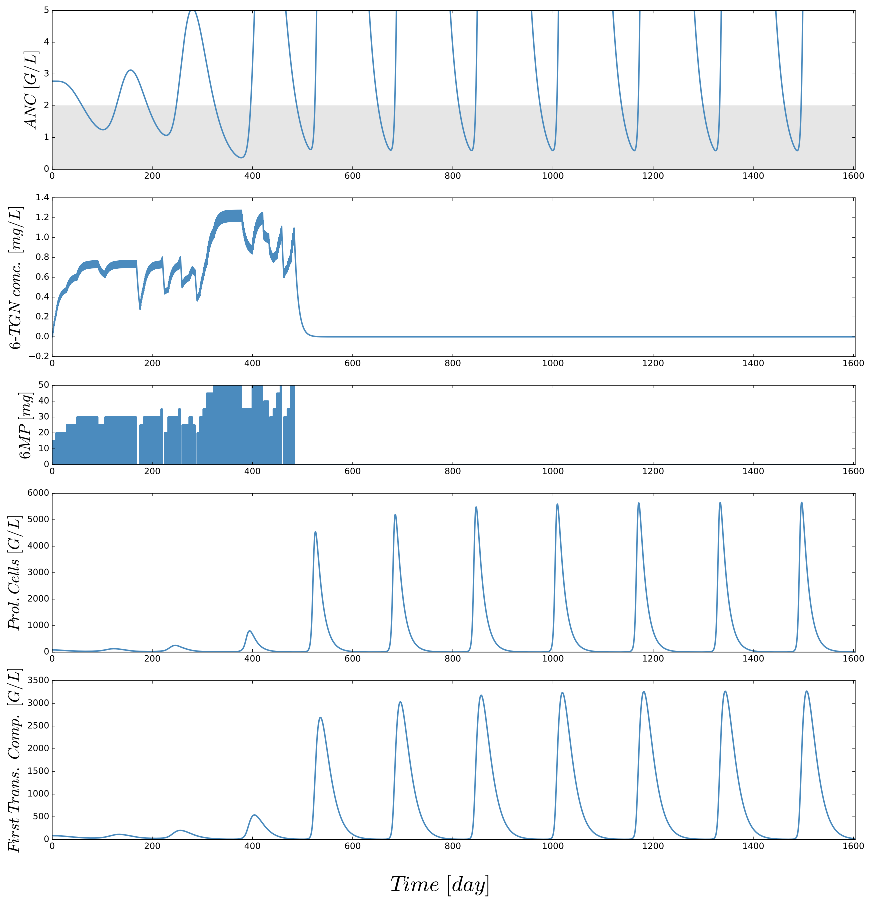

Figure 20: As Figure 1, but for another patient out of 116 patients.

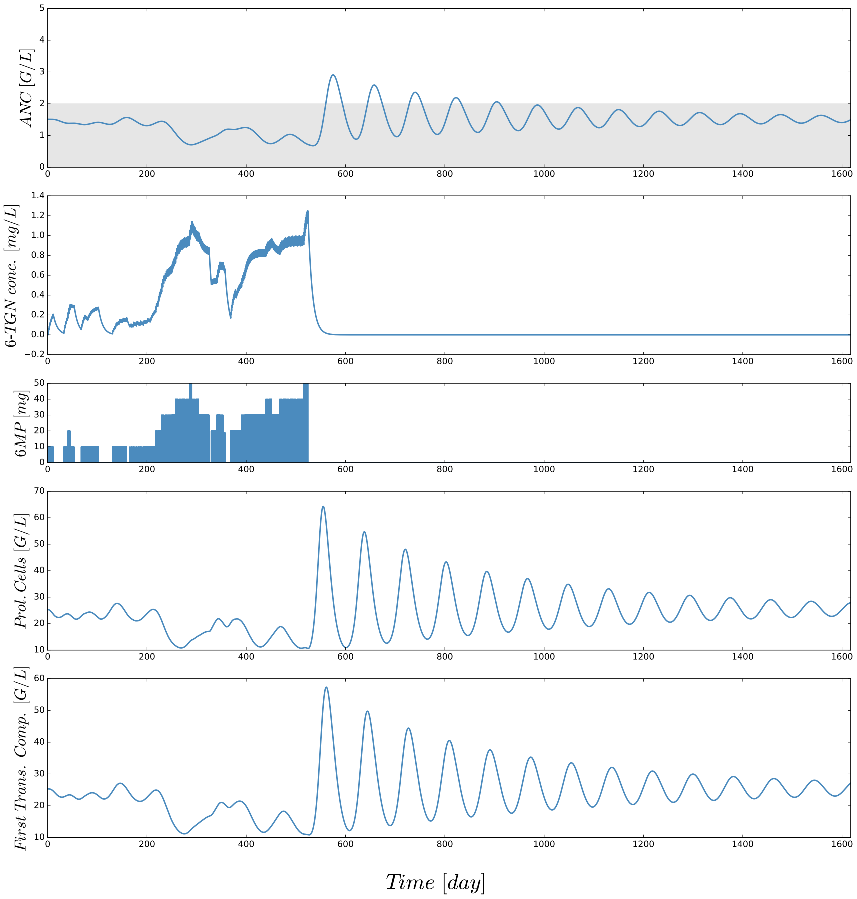

Figure 21: As Figure 1, but for another patient out of 116 patients.

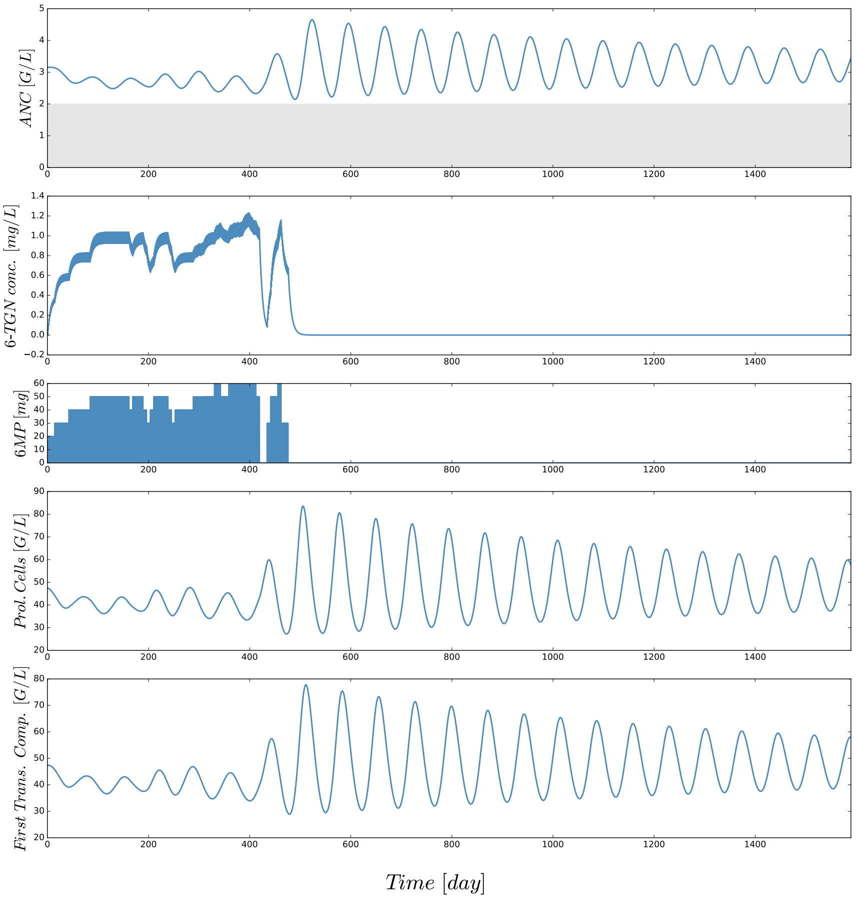

Figure 22: As Figure 1, but for another patient out of 116 patients.

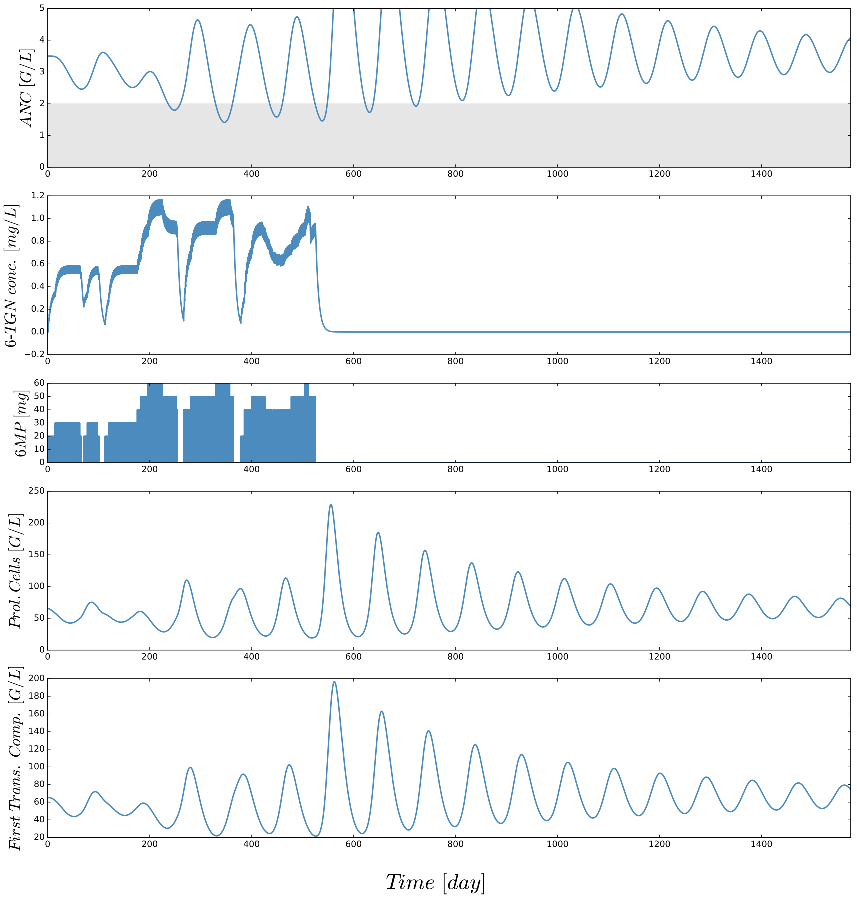

Figure 23: As Figure 1, but for another patient out of 116 patients.

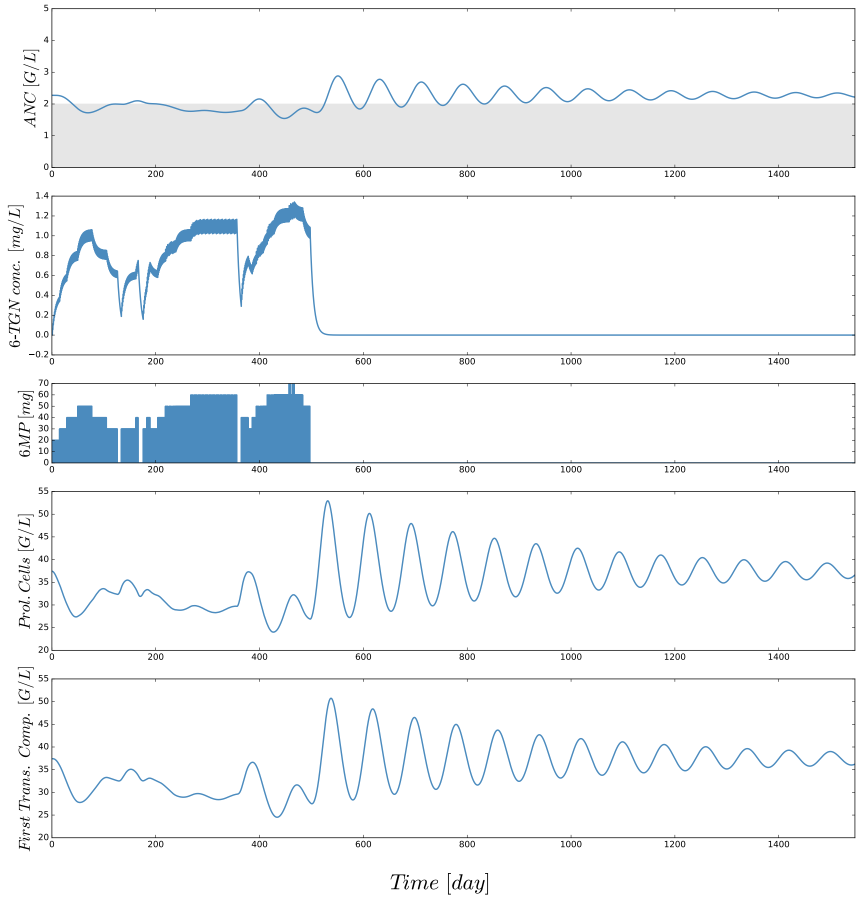

Figure 24: As Figure 1, but for another patient out of 116 patients.

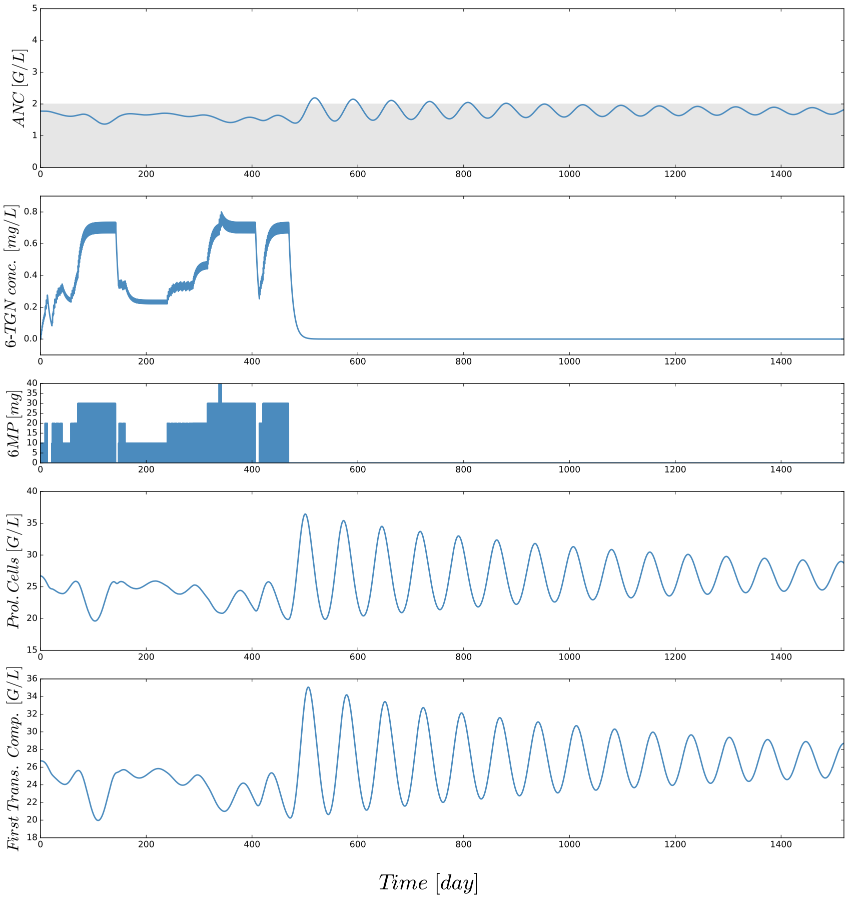

Figure 25: As Figure 1, but for another patient out of 116 patients.

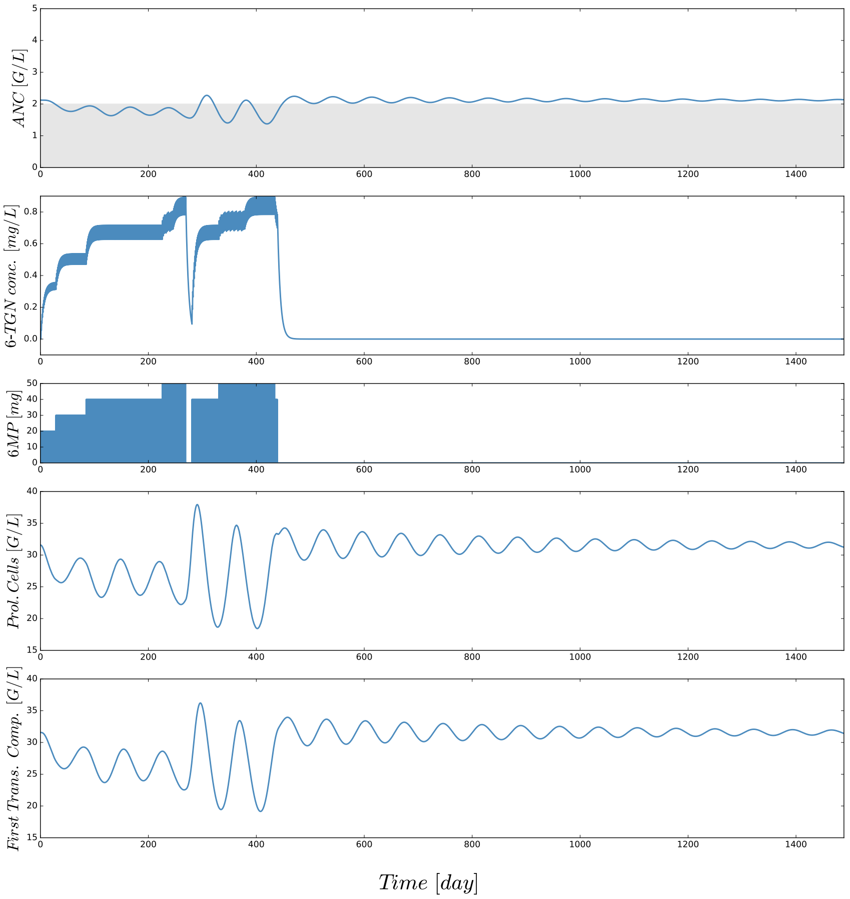

Figure 26: As Figure 1, but for another patient out of 116 patients.

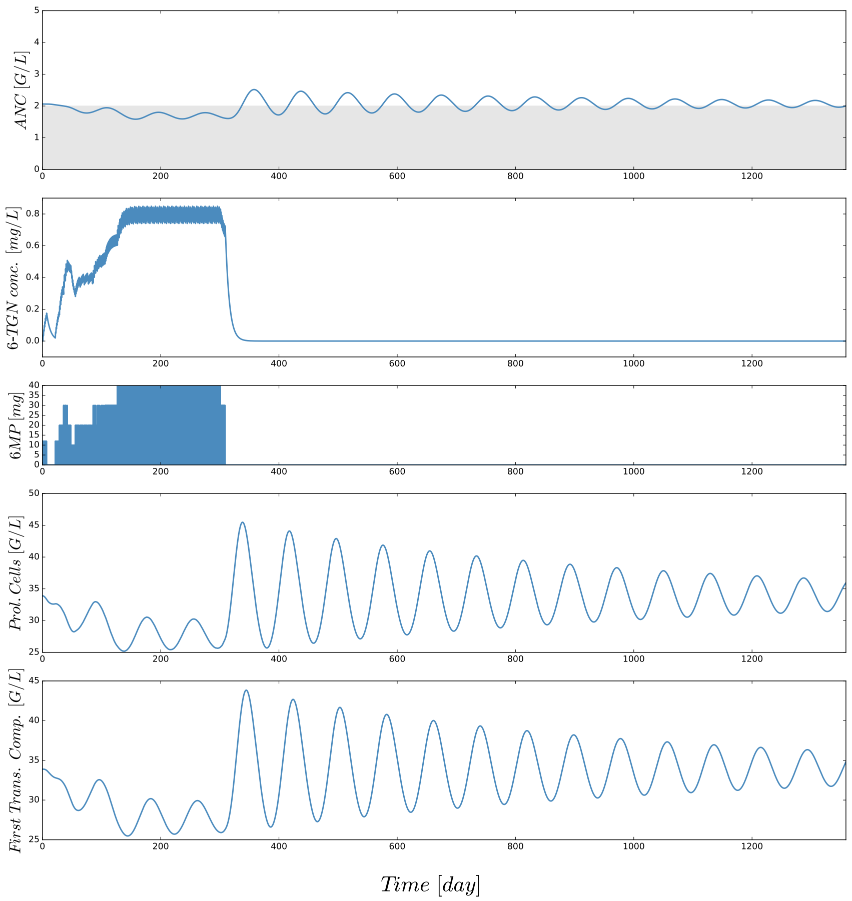

Figure 27: As Figure 1, but for another patient out of 116 patients.

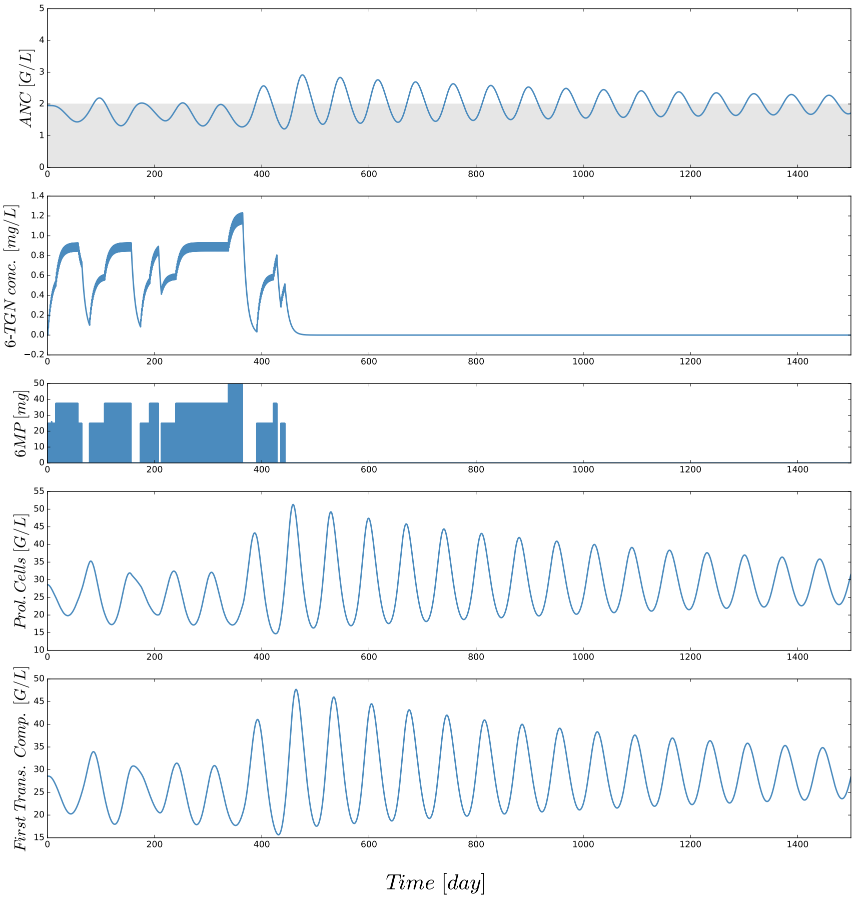

Figure 28: As Figure 1, but for another patient out of 116 patients.

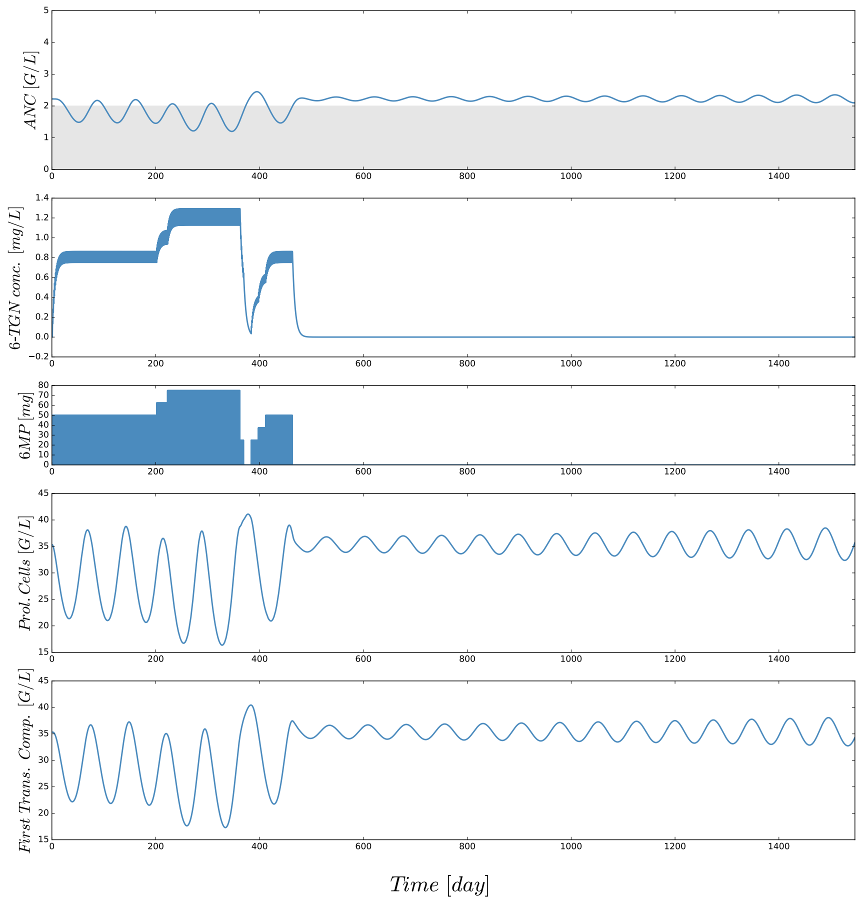

Figure 29: As Figure 1, but for another patient out of 116 patients.

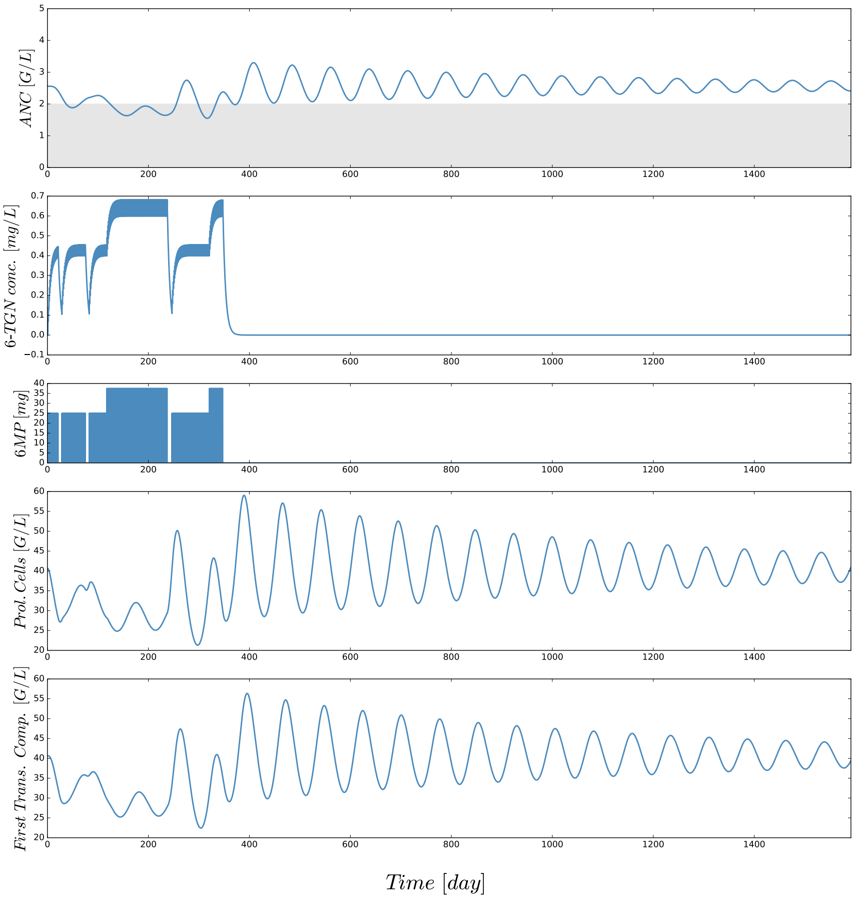

Figure 30: As Figure 1, but for another patient out of 116 patients.

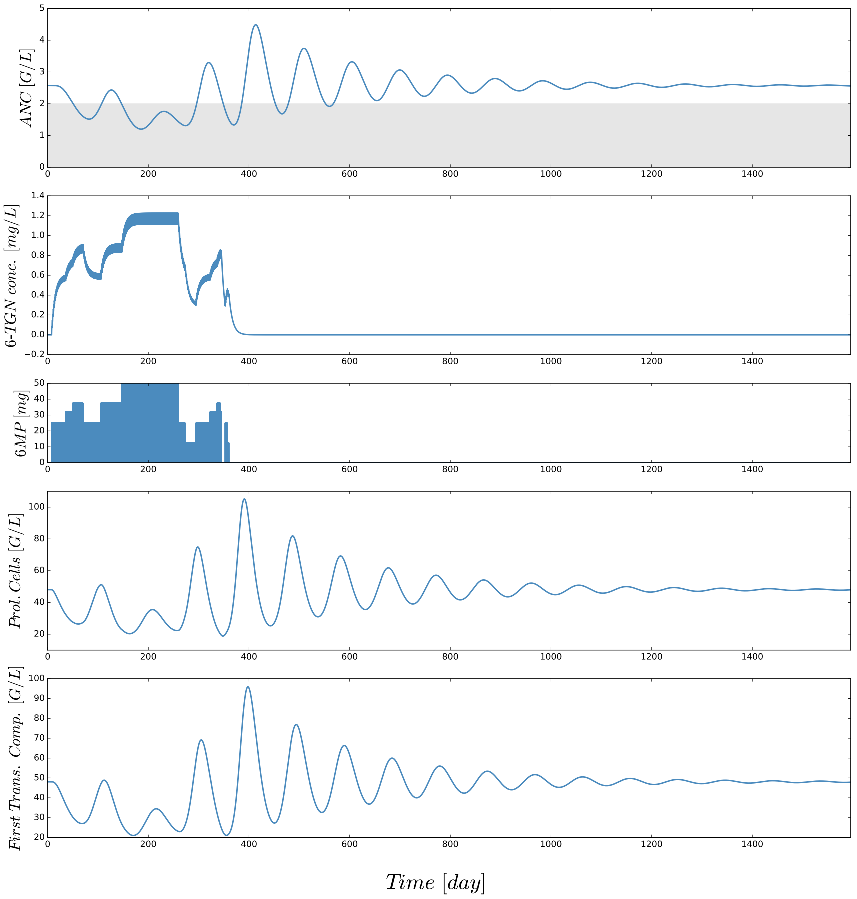

Figure 31: As Figure 1, but for another patient out of 116 patients.

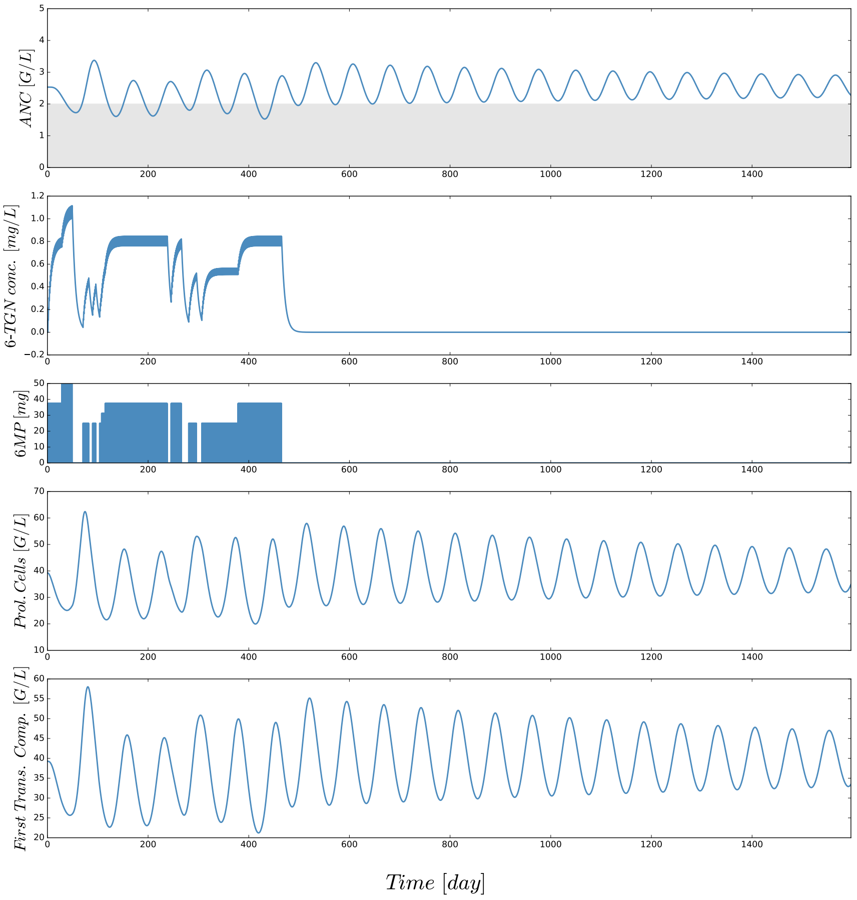

Figure 32: As Figure 1, but for another patient out of 116 patients.

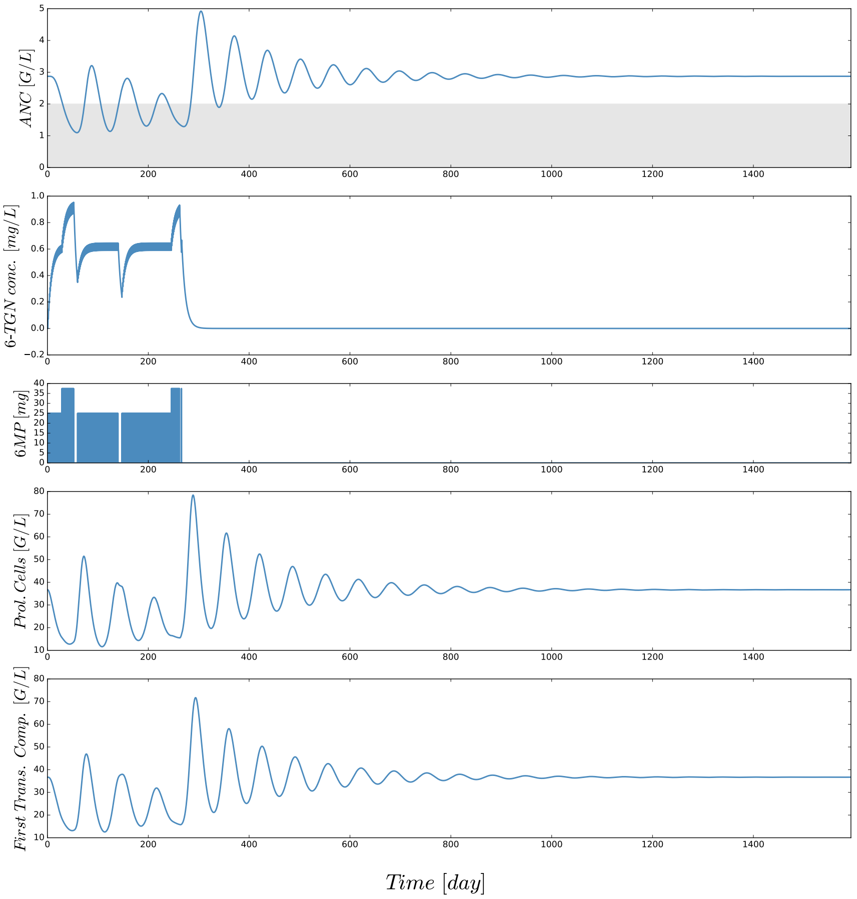

Figure 33: As Figure 1, but for another patient out of 116 patients.

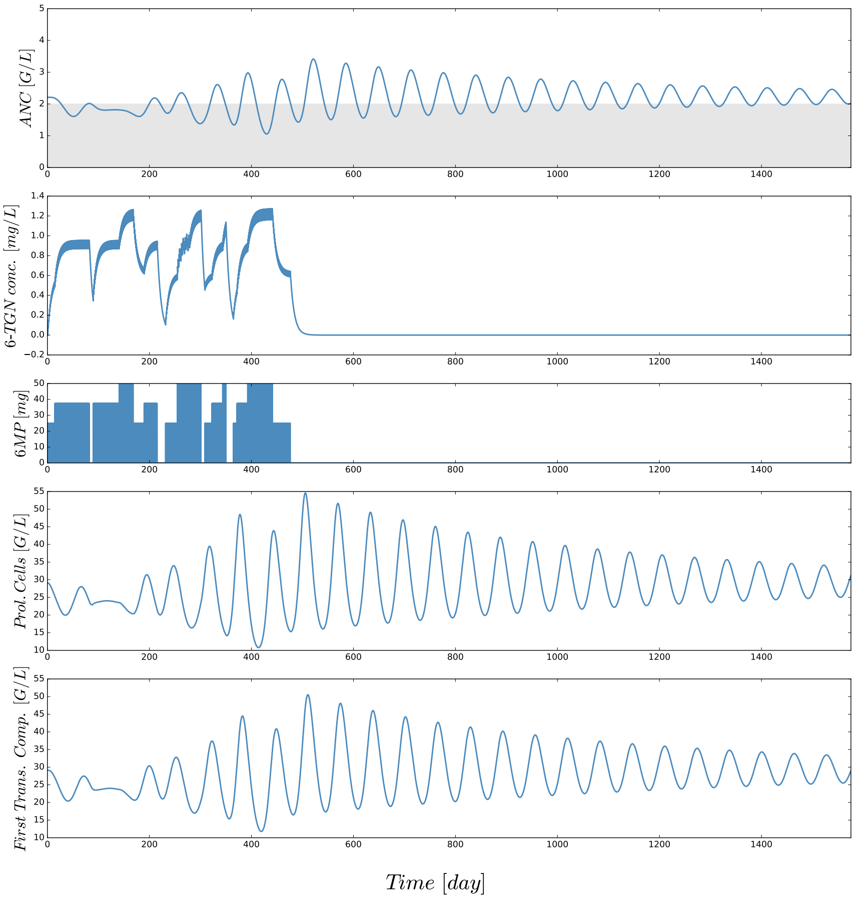

Figure 34: As Figure 1, but for another patient out of 116 patients.

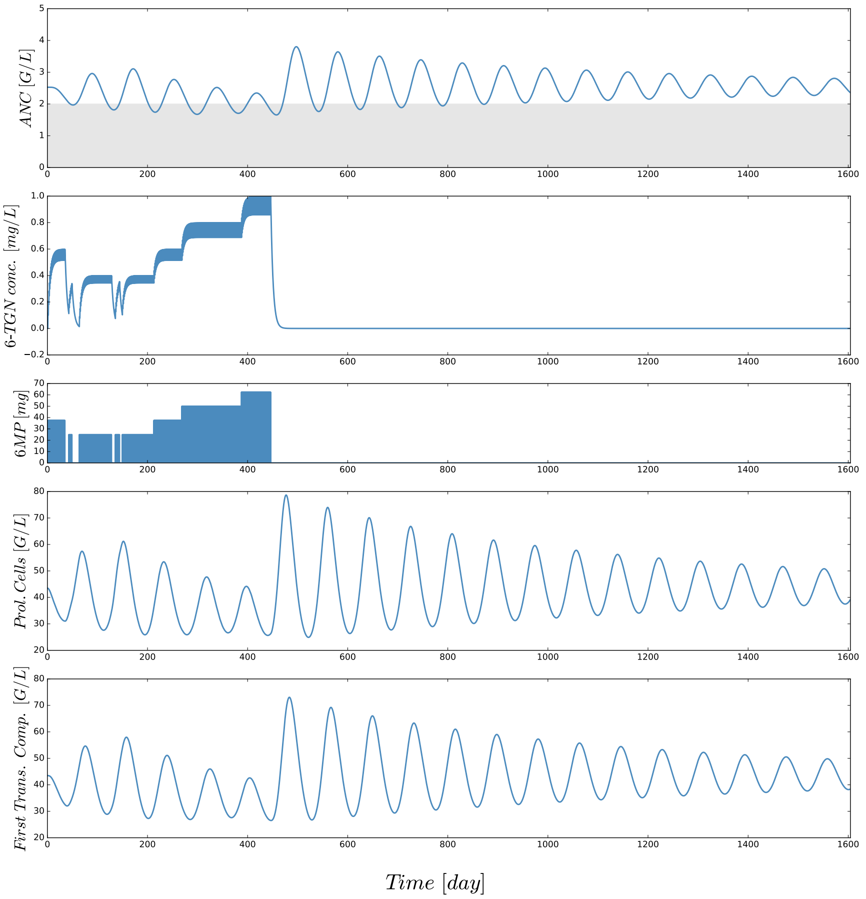

Figure 35: As Figure 1, but for another patient out of 116 patients.

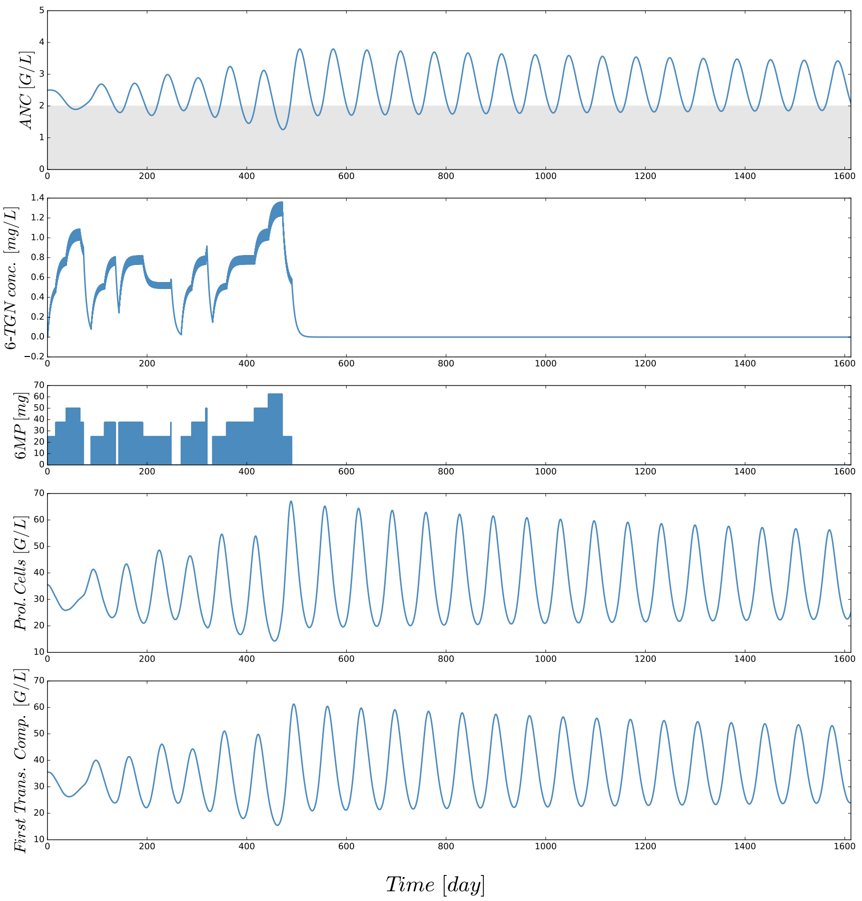

Figure 36: As Figure 1, but for another patient out of 116 patients.

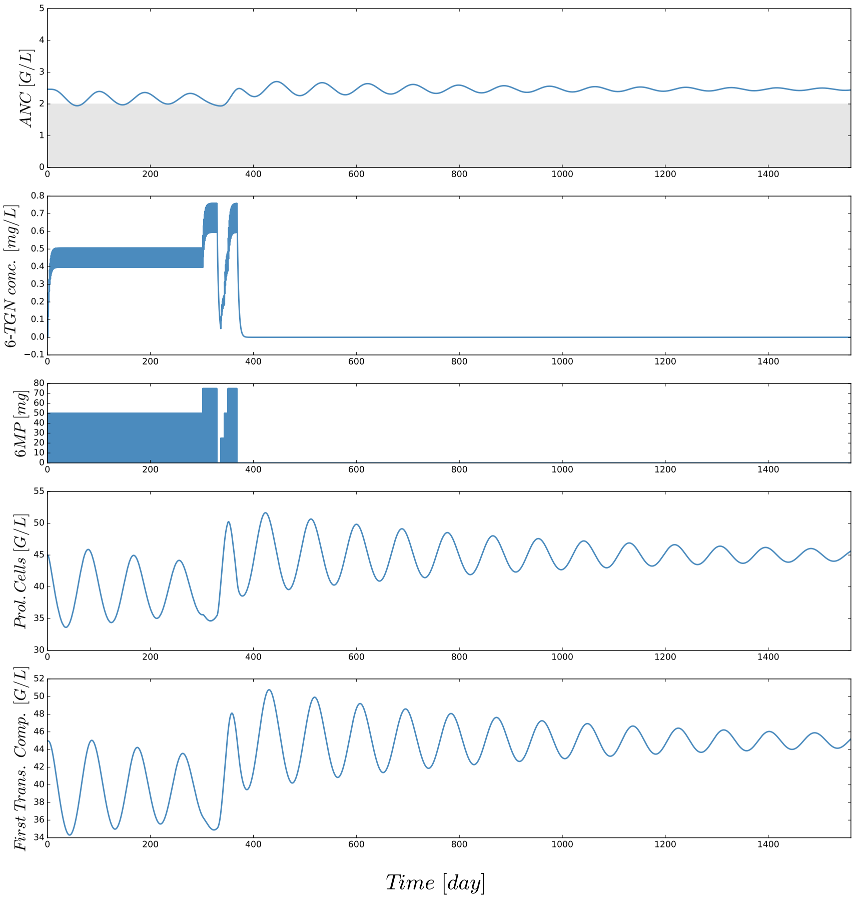

Figure 37: As Figure 1, but for another patient out of 116 patients.

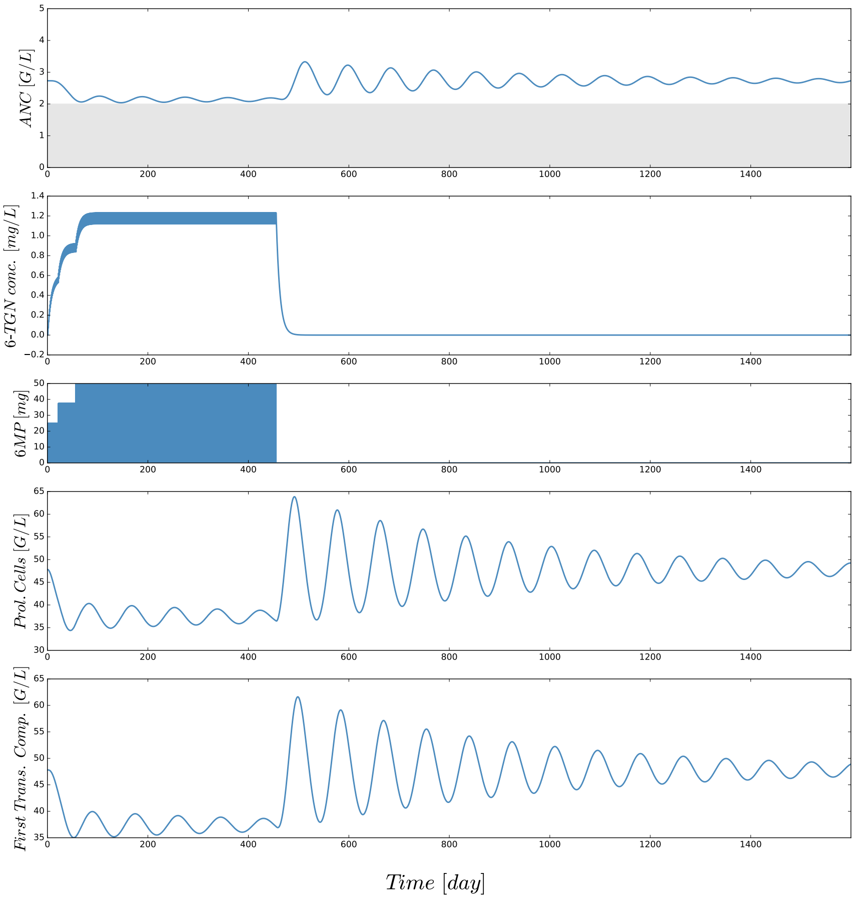

Figure 38: As Figure 1, but for another patient out of 116 patients.

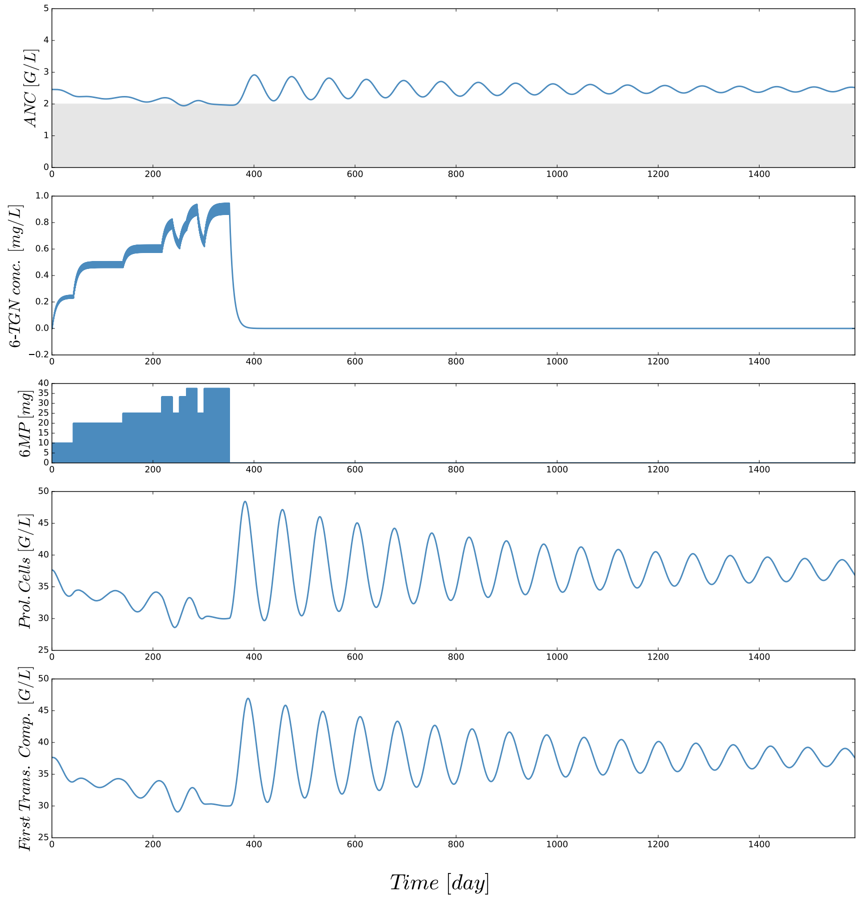

Figure 39: As Figure 1, but for another patient out of 116 patients.

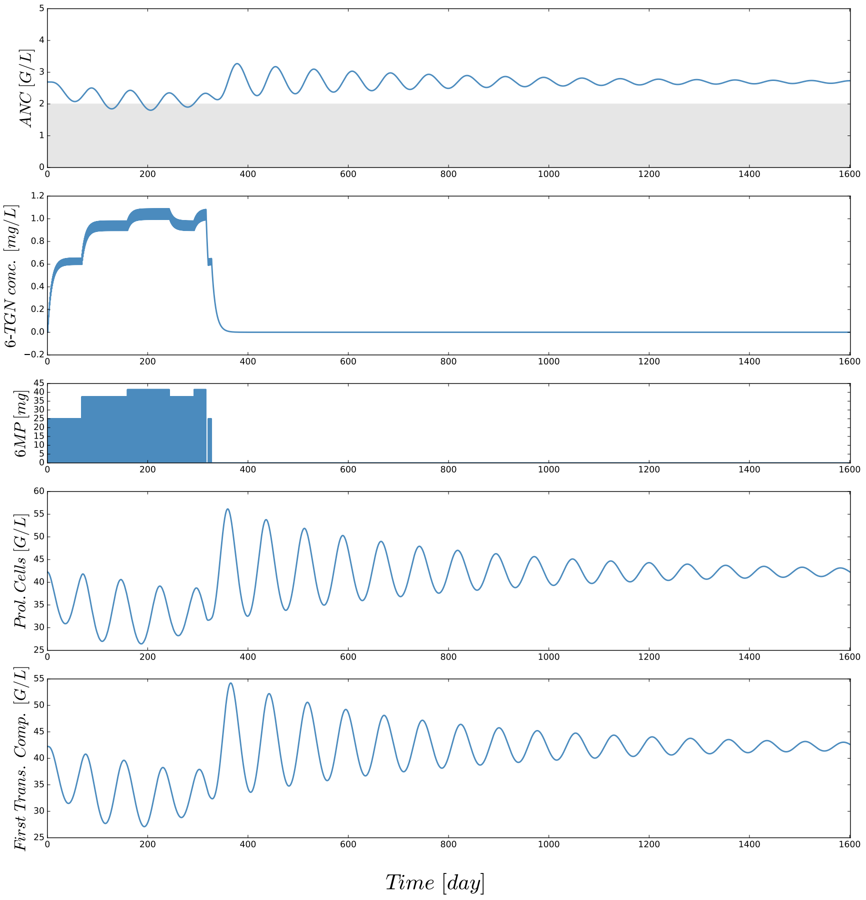

Figure 40: As Figure 1, but for another patient out of 116 patients.

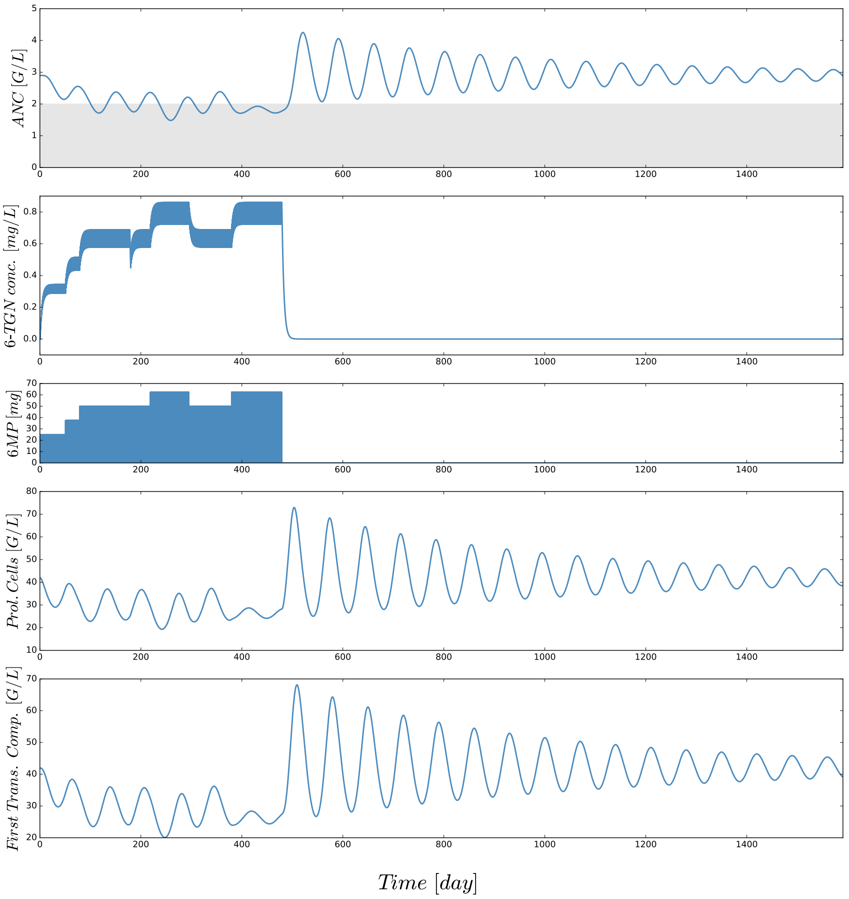

Figure 41: As Figure 1, but for another patient out of 116 patients.

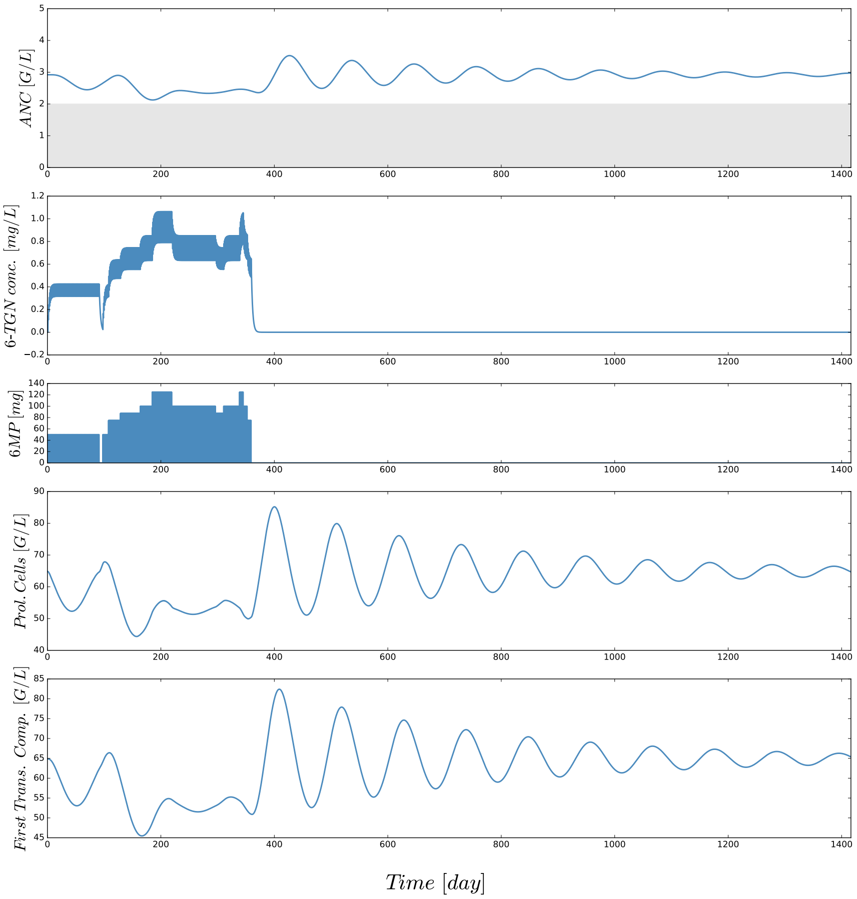

Figure 42: As Figure 1, but for another patient out of 116 patients.

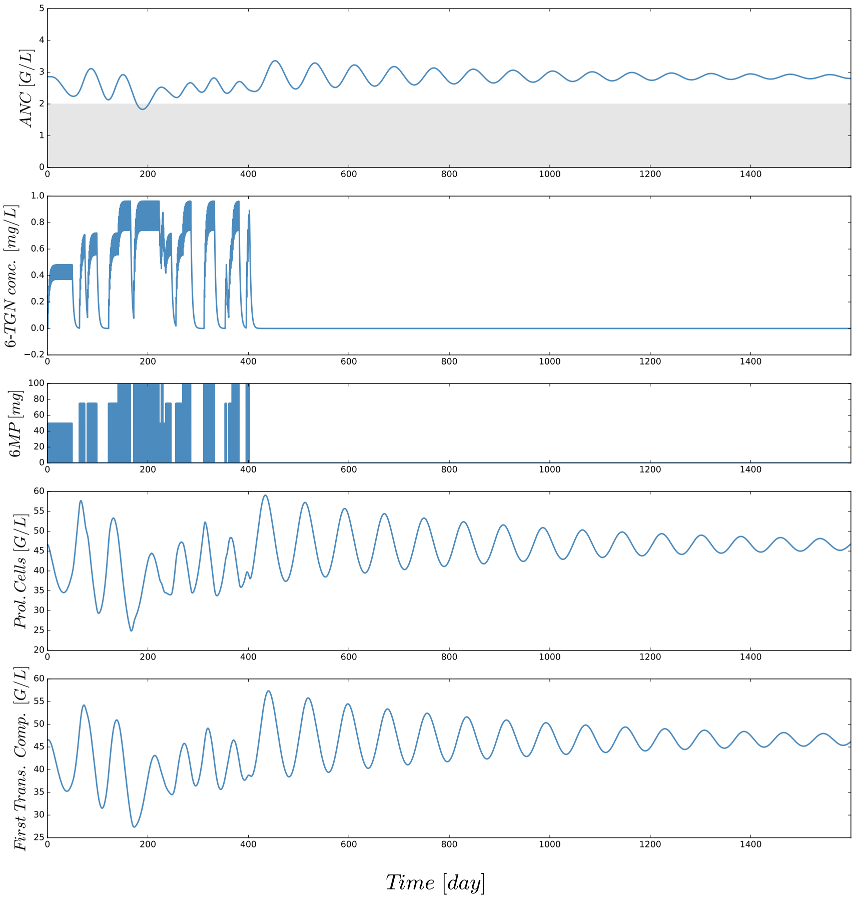

Figure 43: As Figure 1, but for another patient out of 116 patients.

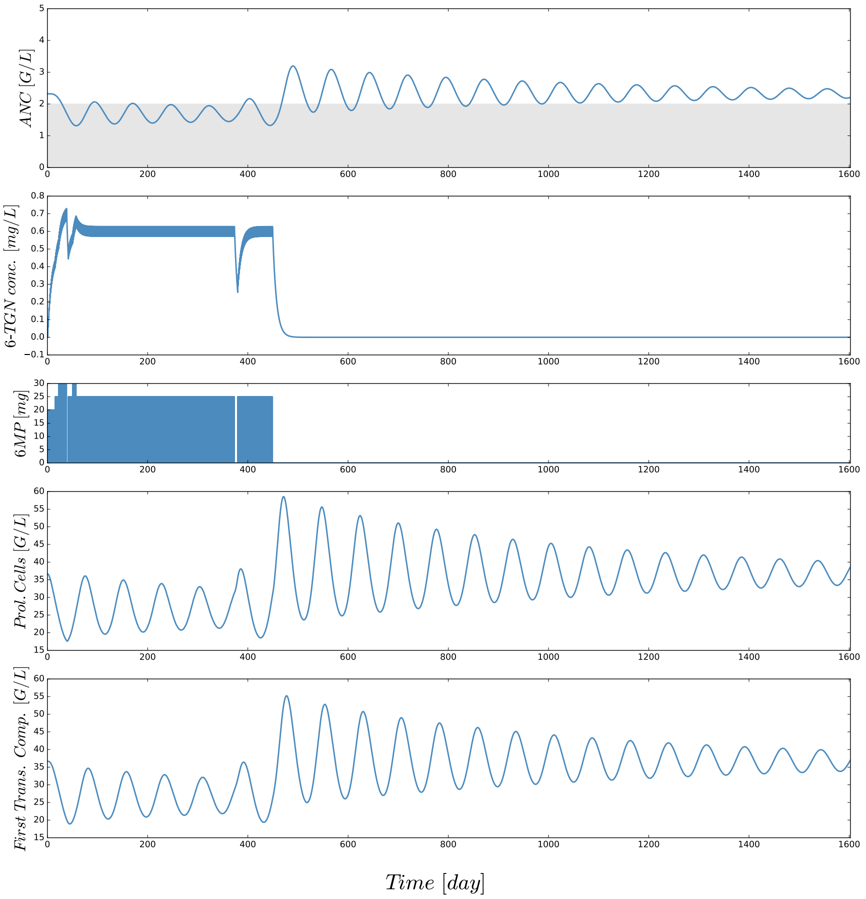

Figure 44: As Figure 1, but for another patient out of 116 patients.

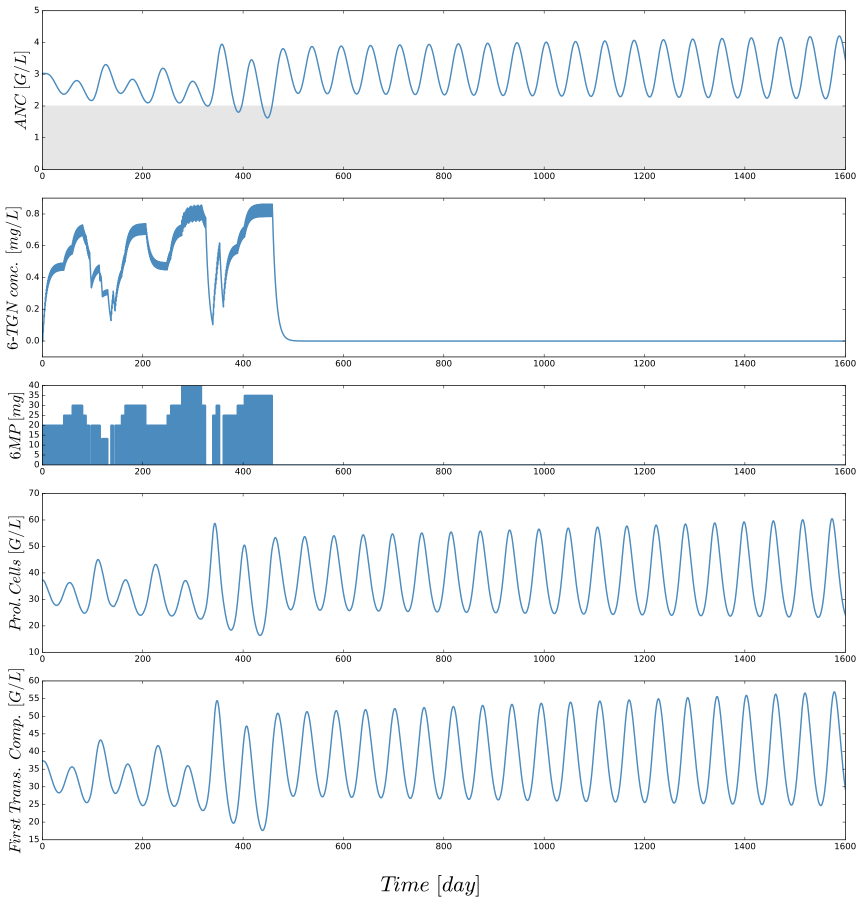

Figure 45: As Figure 1, but for another patient out of 116 patients.

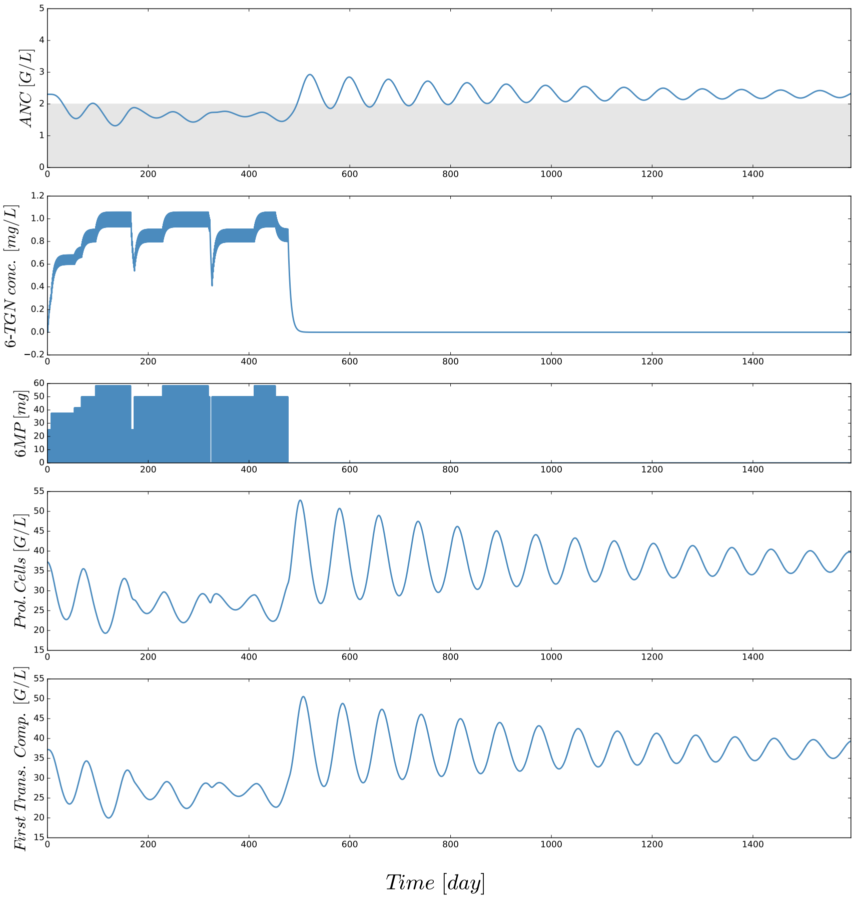

Figure 46: As Figure 1, but for another patient out of 116 patients.

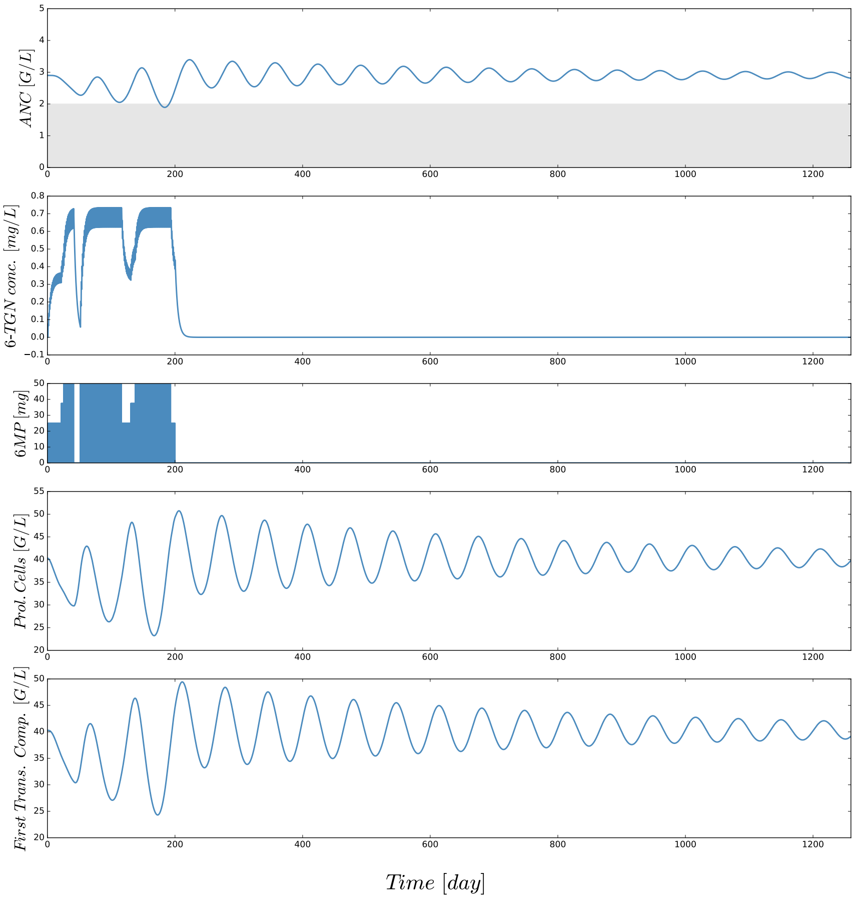

Figure 47: As Figure 1, but for another patient out of 116 patients.

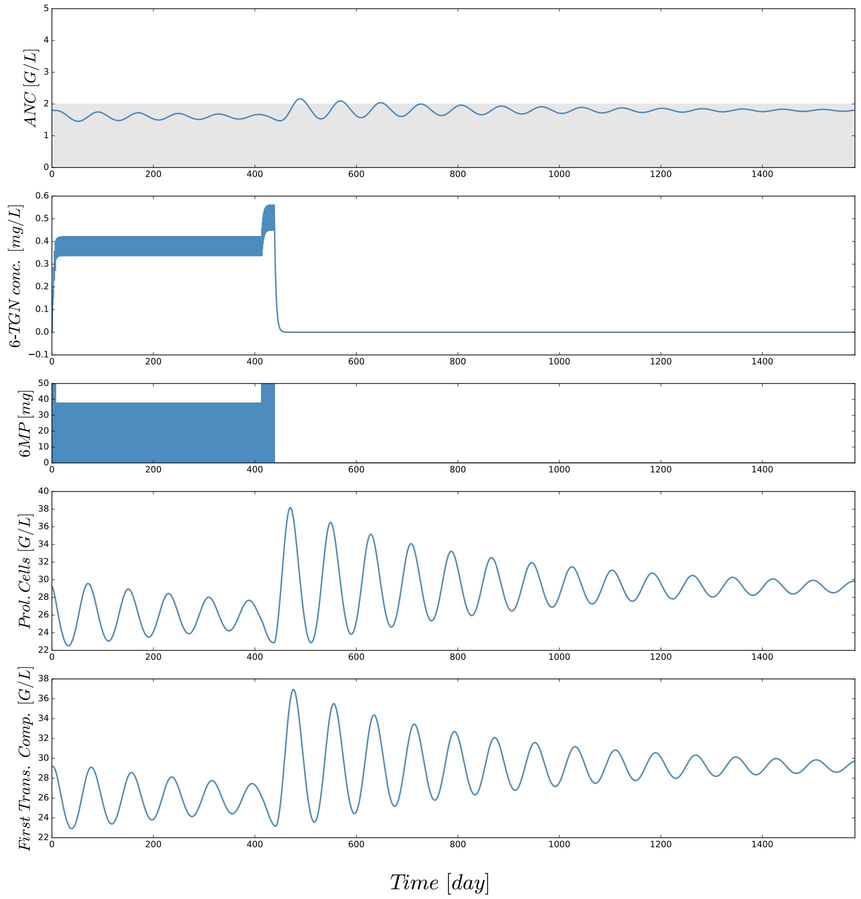

Figure 48: As Figure 1, but for another patient out of 116 patients.

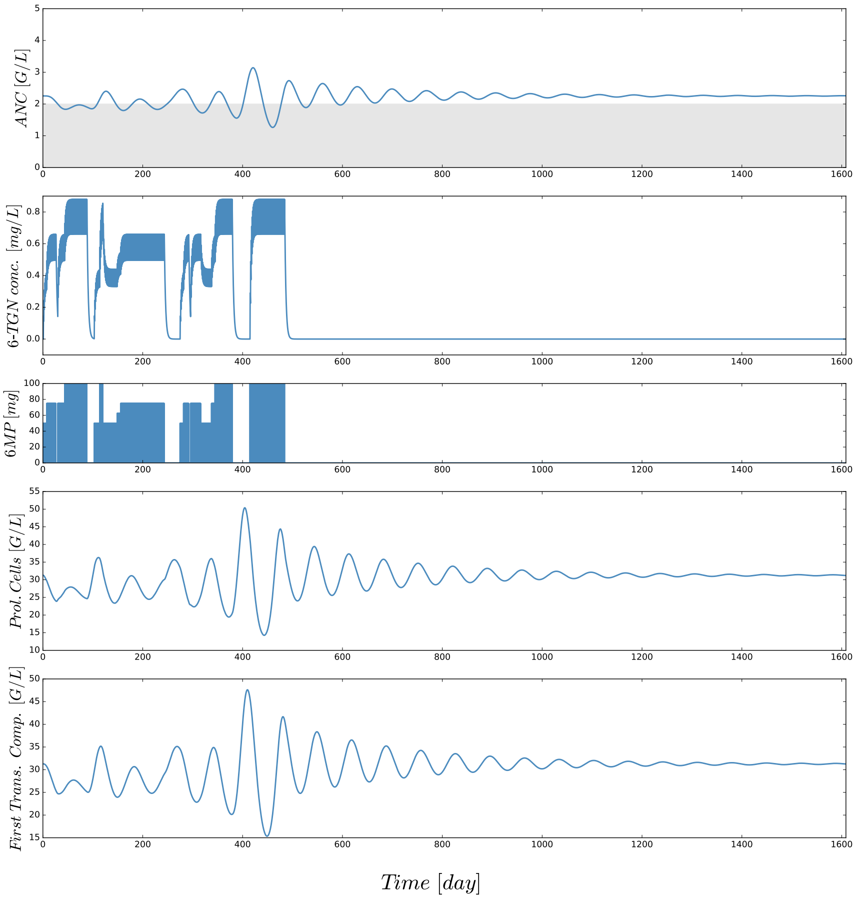

Figure 49: As Figure 1, but for another patient out of 116 patients.

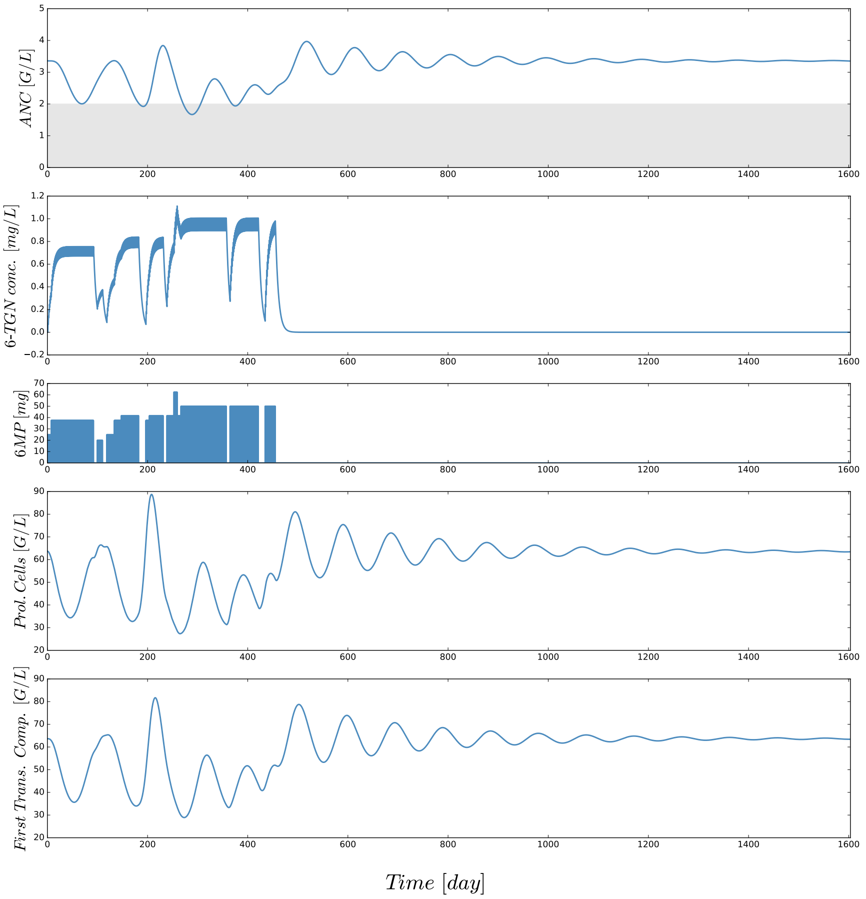

Figure 50: As Figure 1, but for another patient out of 116 patients.

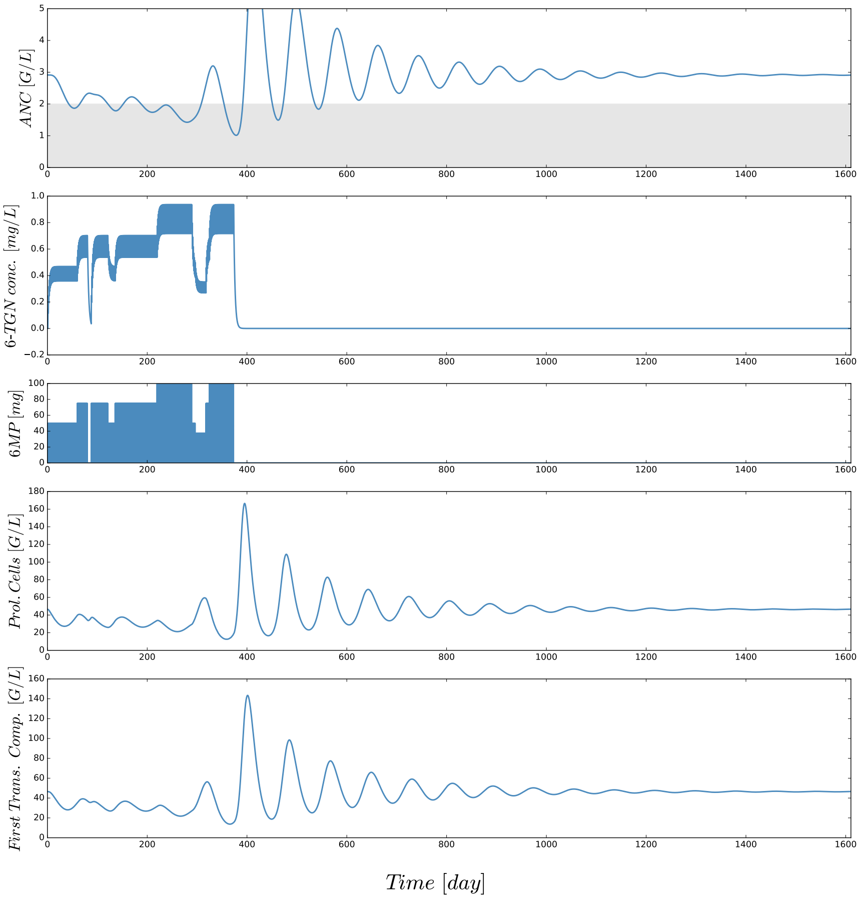

Figure 51: As Figure 1, but for another patient out of 116 patients.

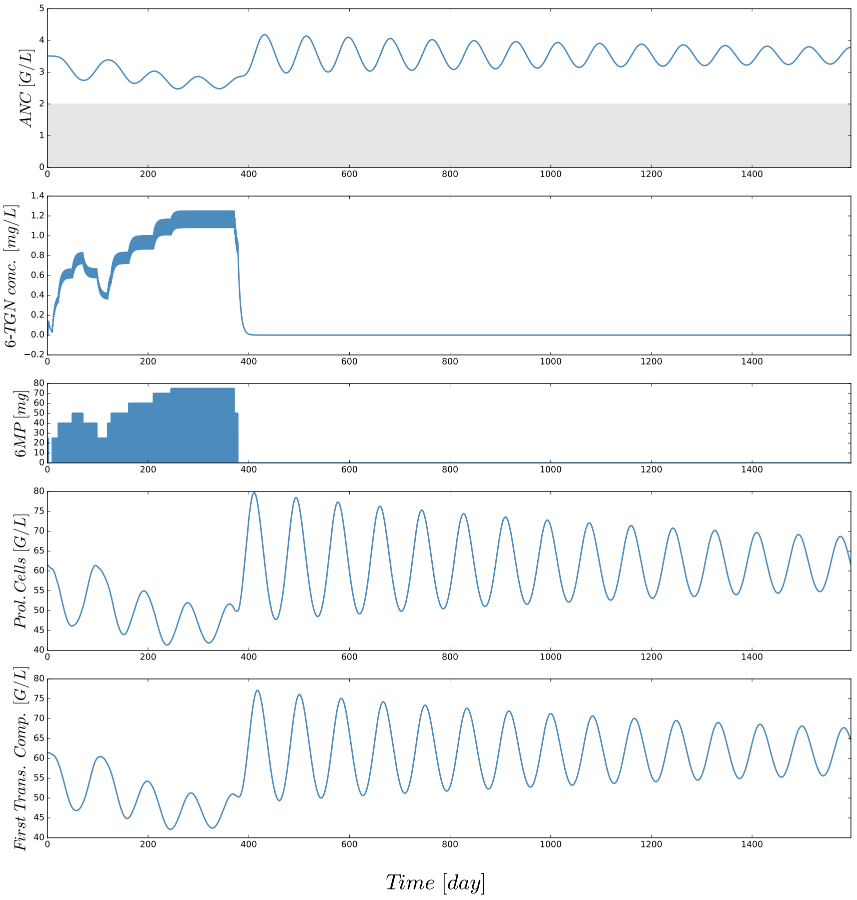

Figure 52: As Figure 1, but for another patient out of 116 patients.

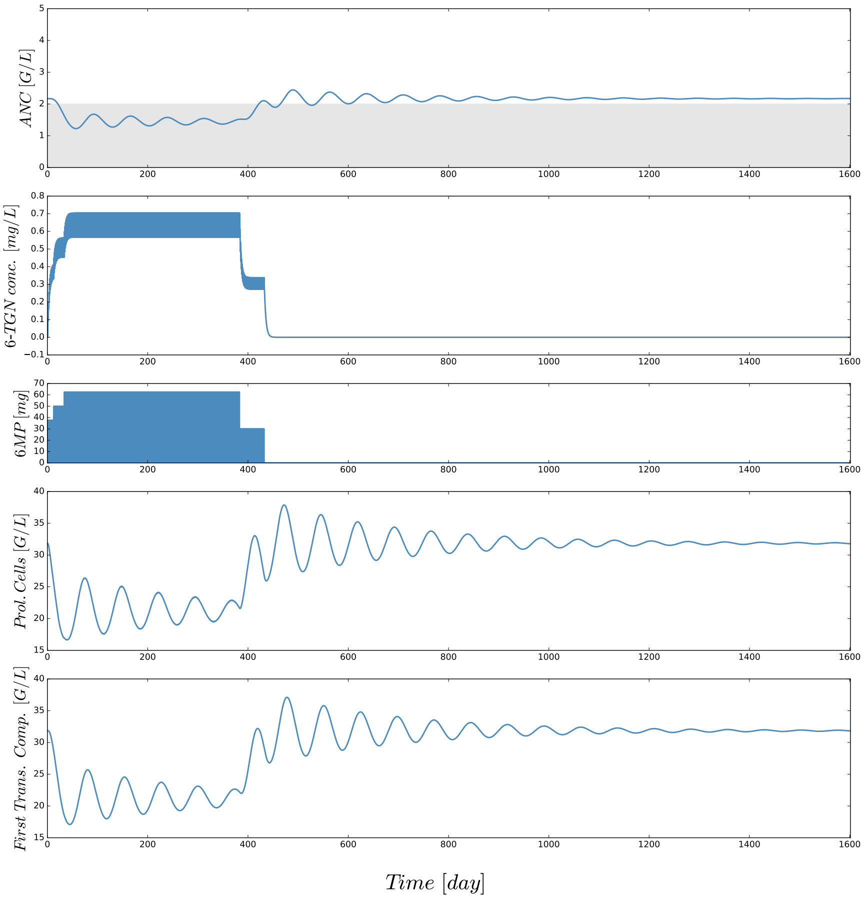

Figure 53: As Figure 1, but for another patient out of 116 patients.

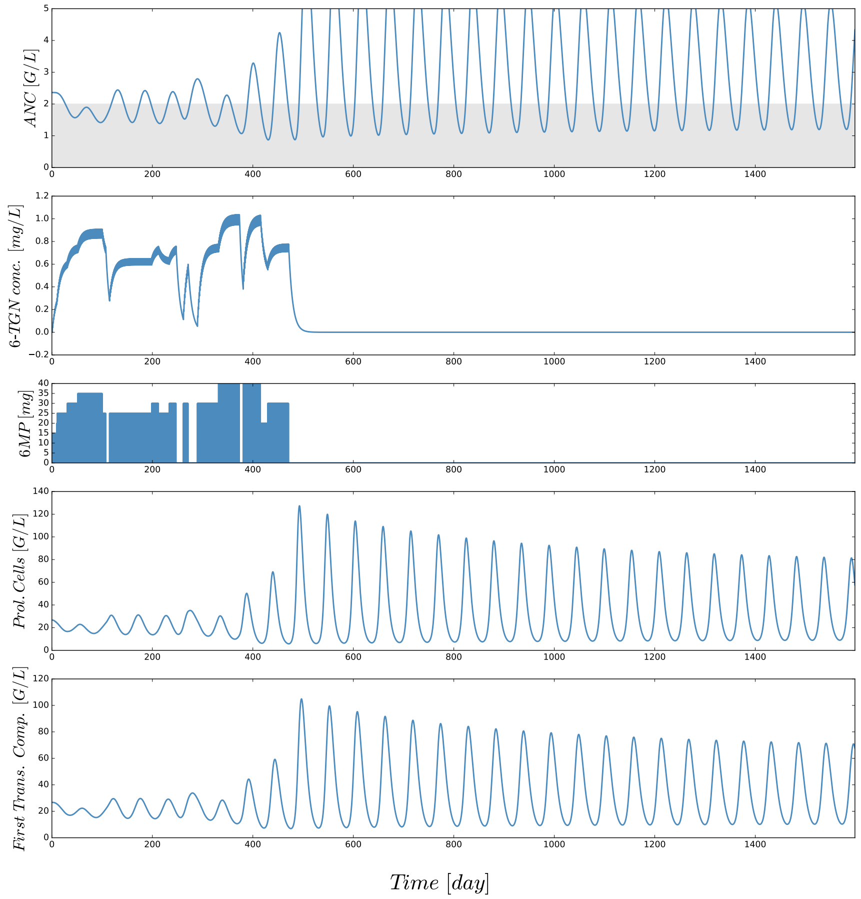

Figure 54: As Figure 1, but for another patient out of 116 patients.

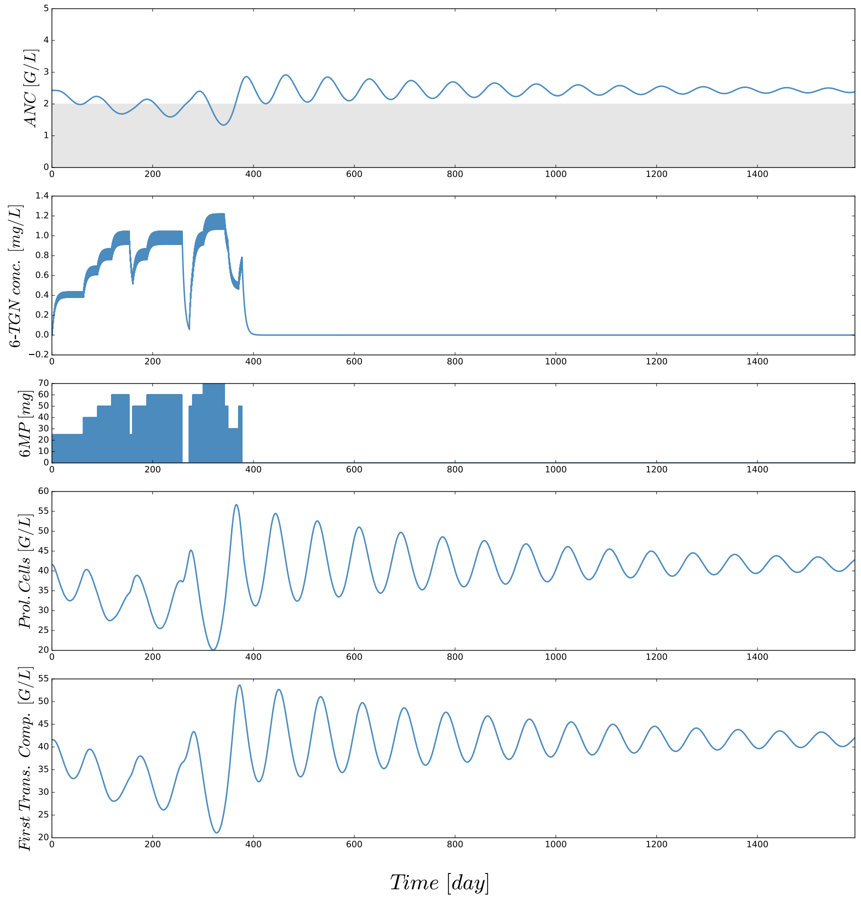

Figure 55: As Figure 1, but for another patient out of 116 patients.

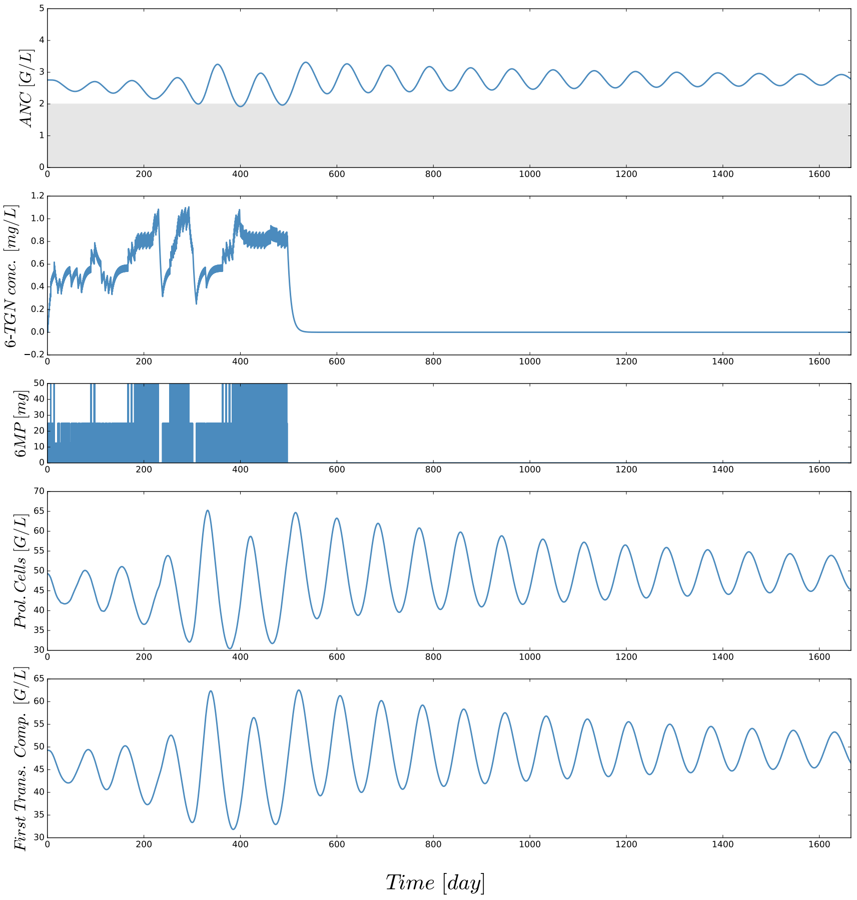

Figure 56: As Figure 1, but for another patient out of 116 patients.

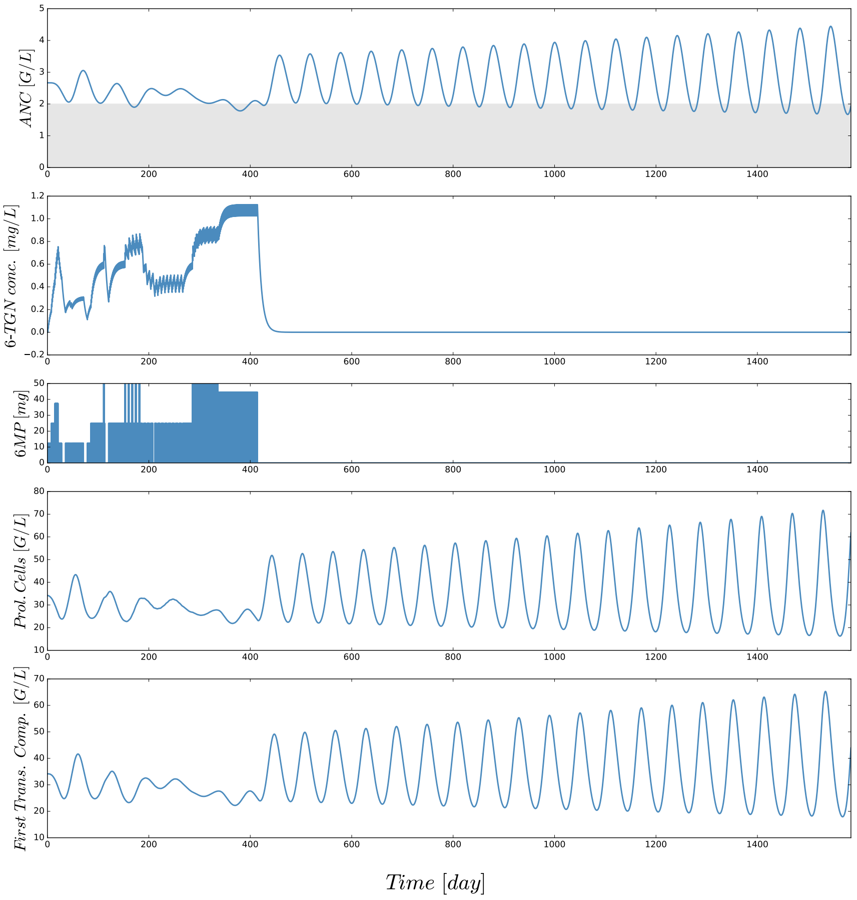

Figure 57: As Figure 1, but for another patient out of 116 patients.

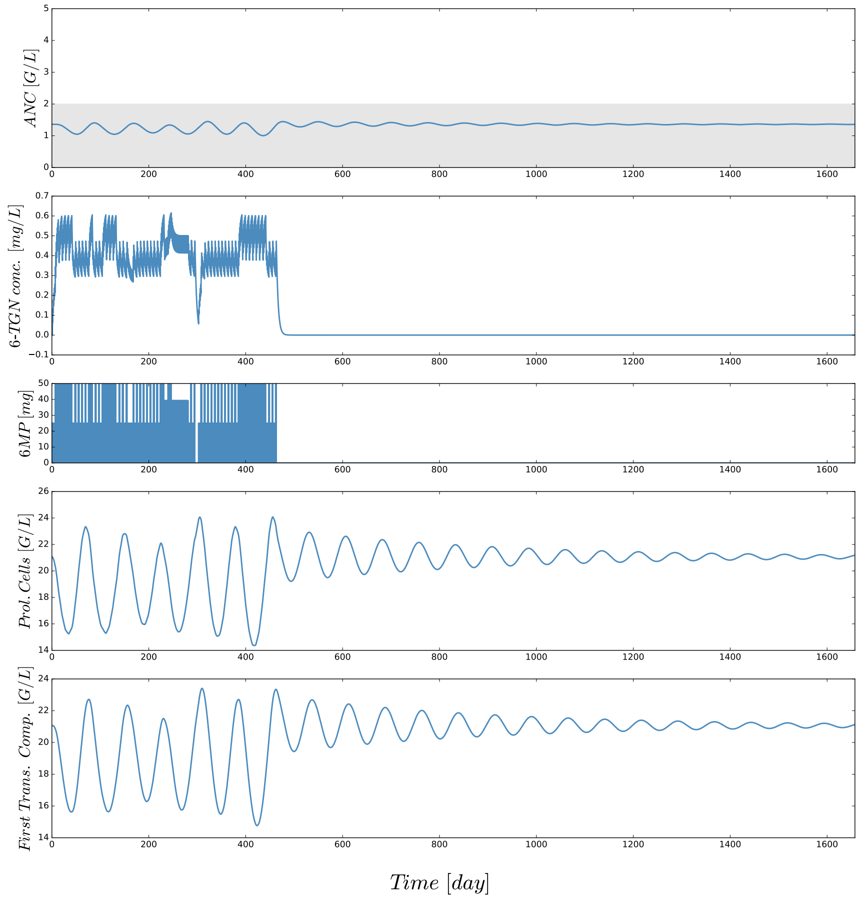

Figure 58: As Figure 1, but for another patient out of 116 patients.

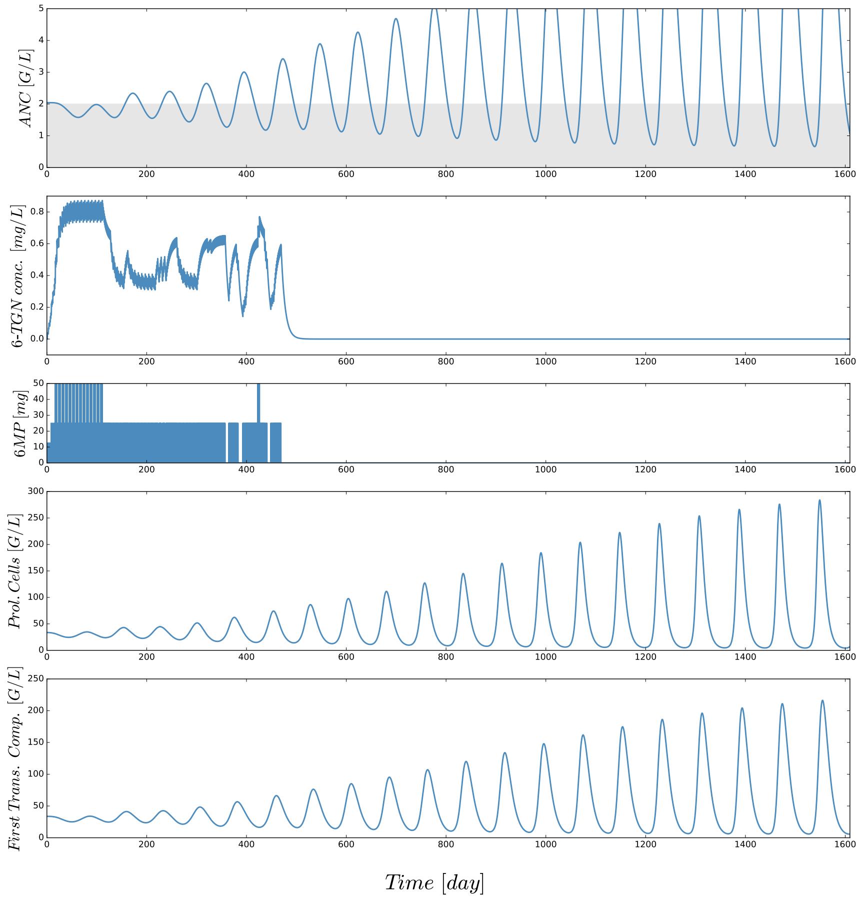

Figure 59: As Figure 1, but for another patient out of 116 patients.

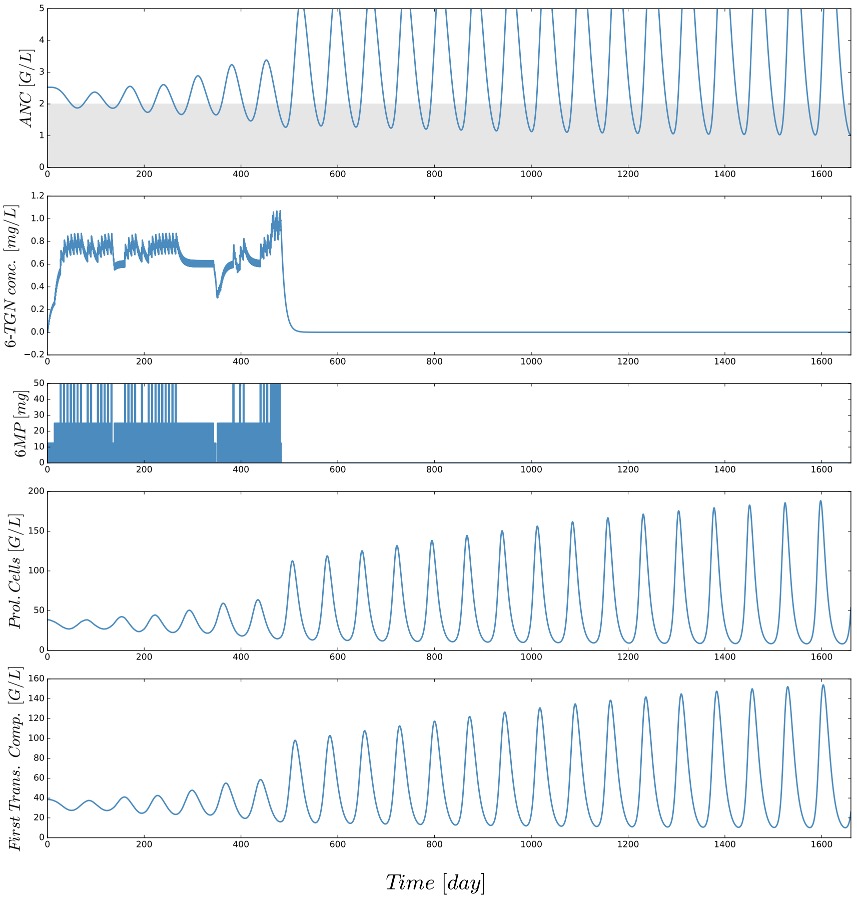

Figure 60: As Figure 1, but for another patient out of 116 patients.

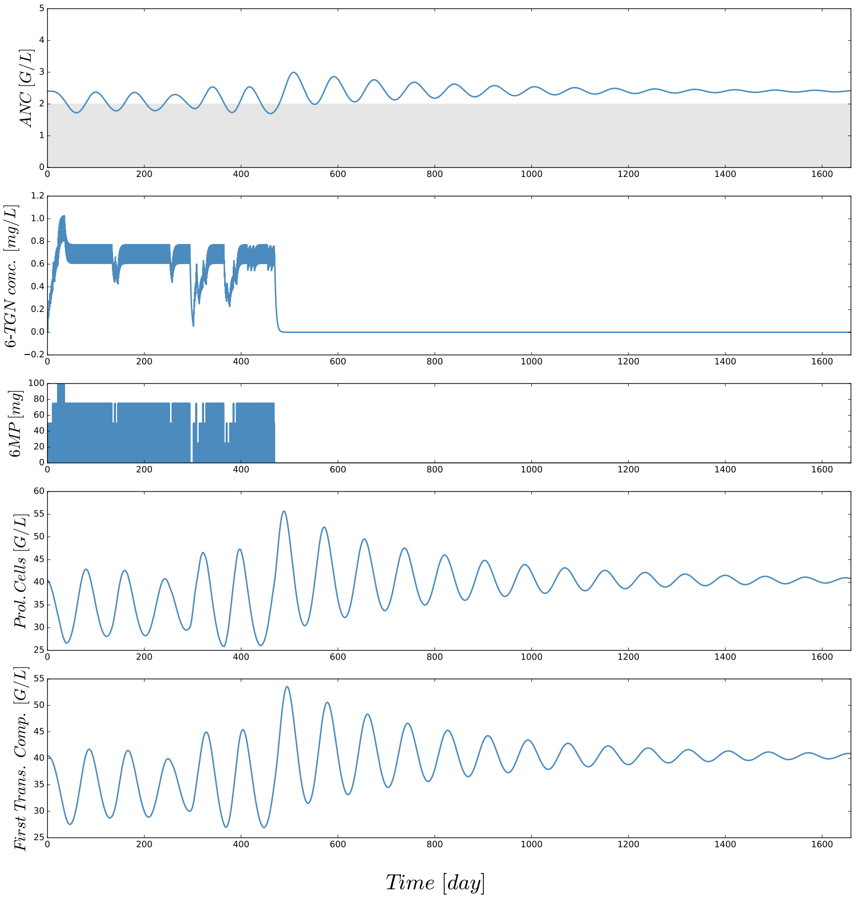

Figure 61: As Figure 1, but for another patient out of 116 patients.

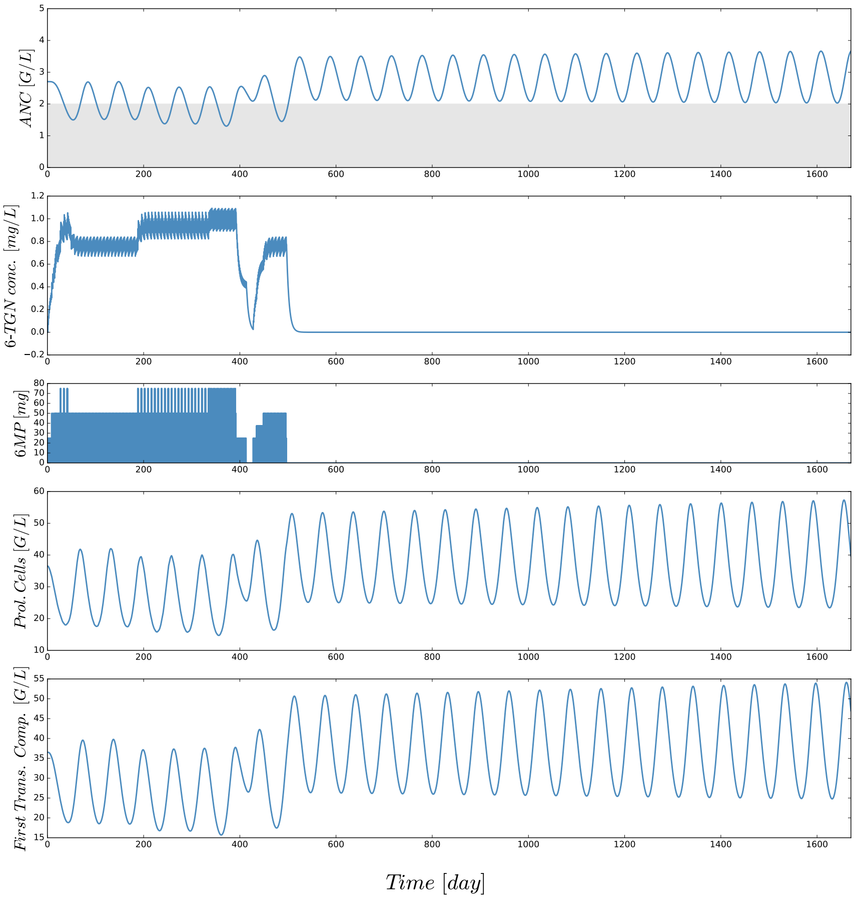

Figure 62: As Figure 1, but for another patient out of 116 patients.

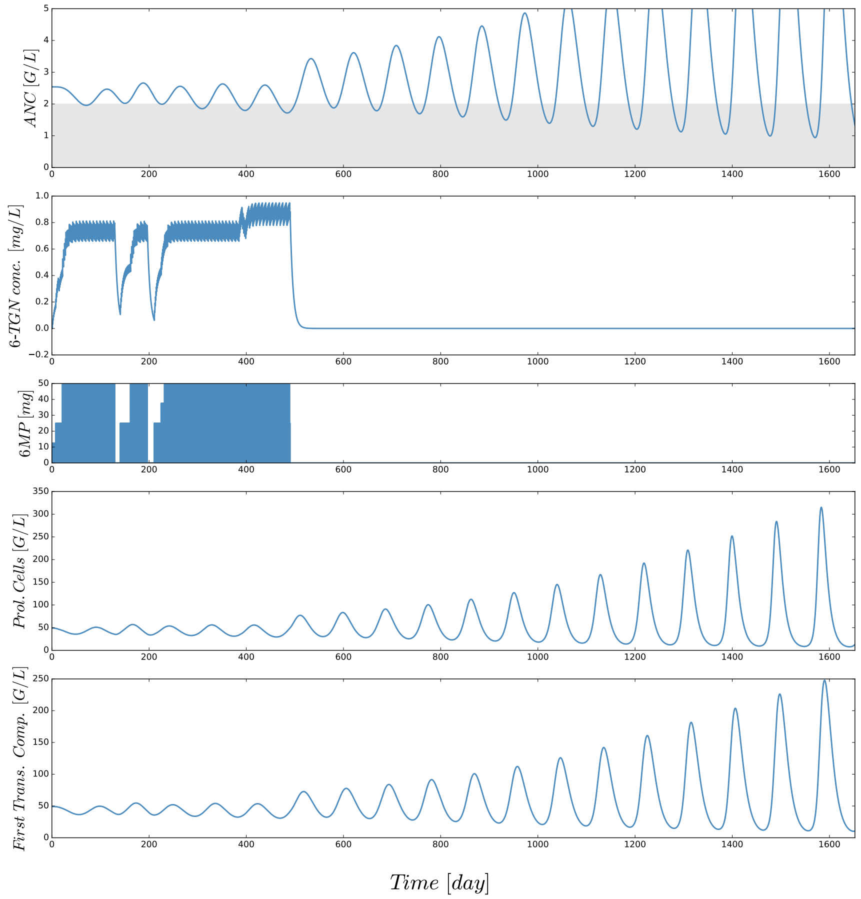

Figure 63: As Figure 1, but for another patient out of 116 patients.

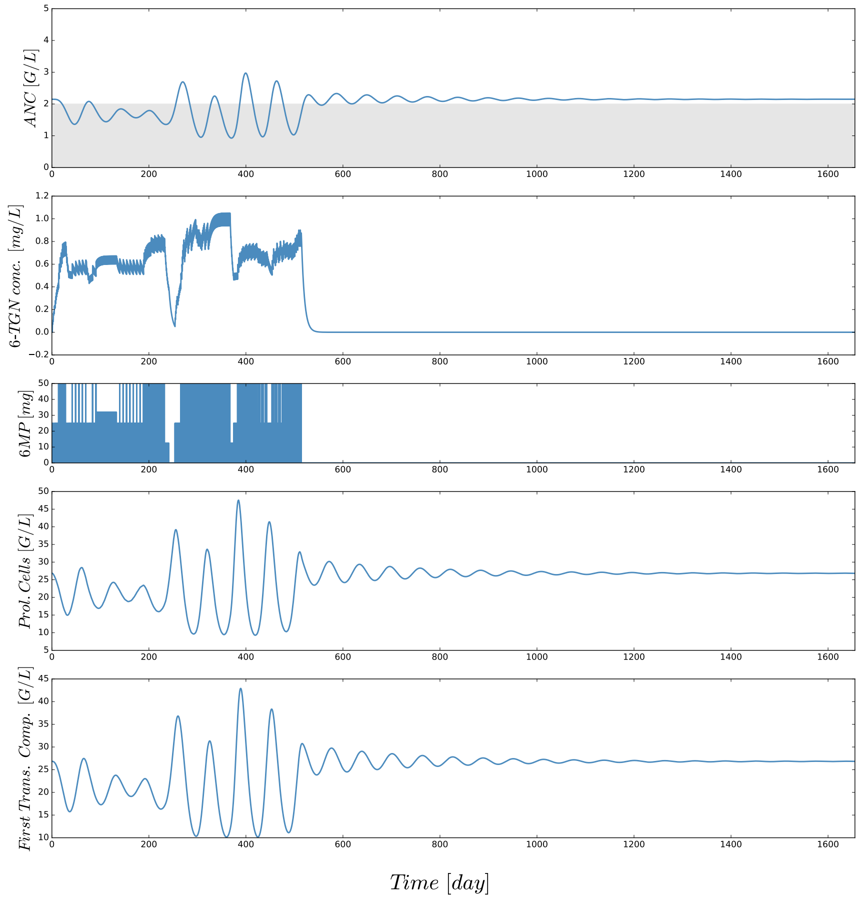

Figure 64: As Figure 1, but for another patient out of 116 patients.

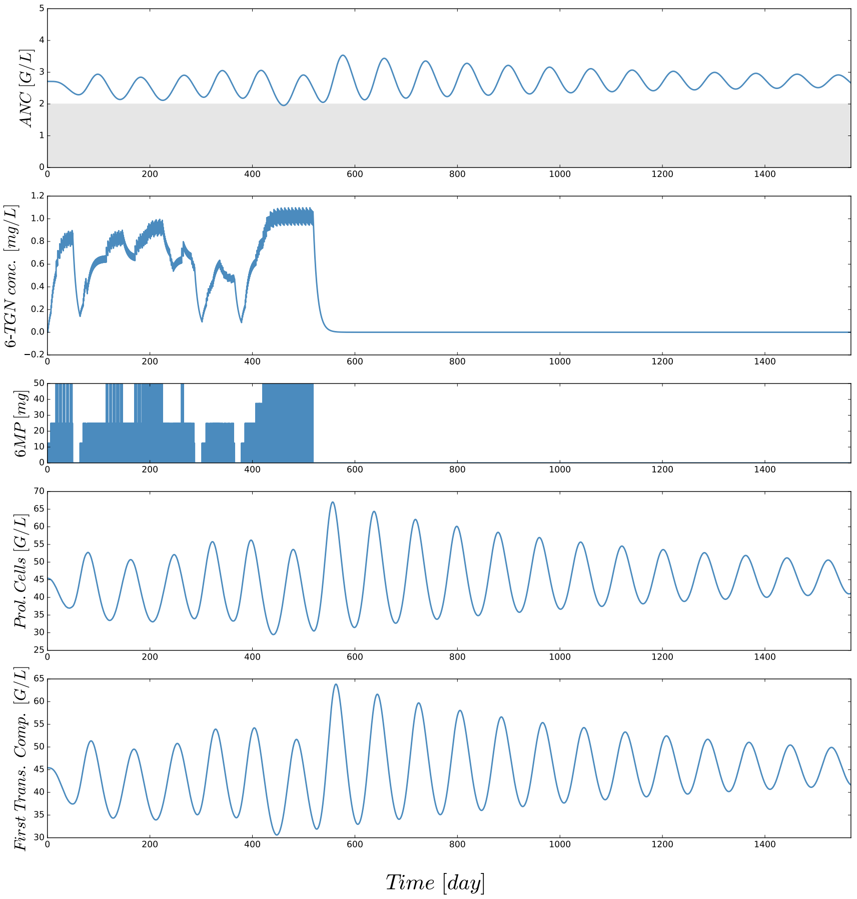

Figure 65: As Figure 1, but for another patient out of 116 patients.

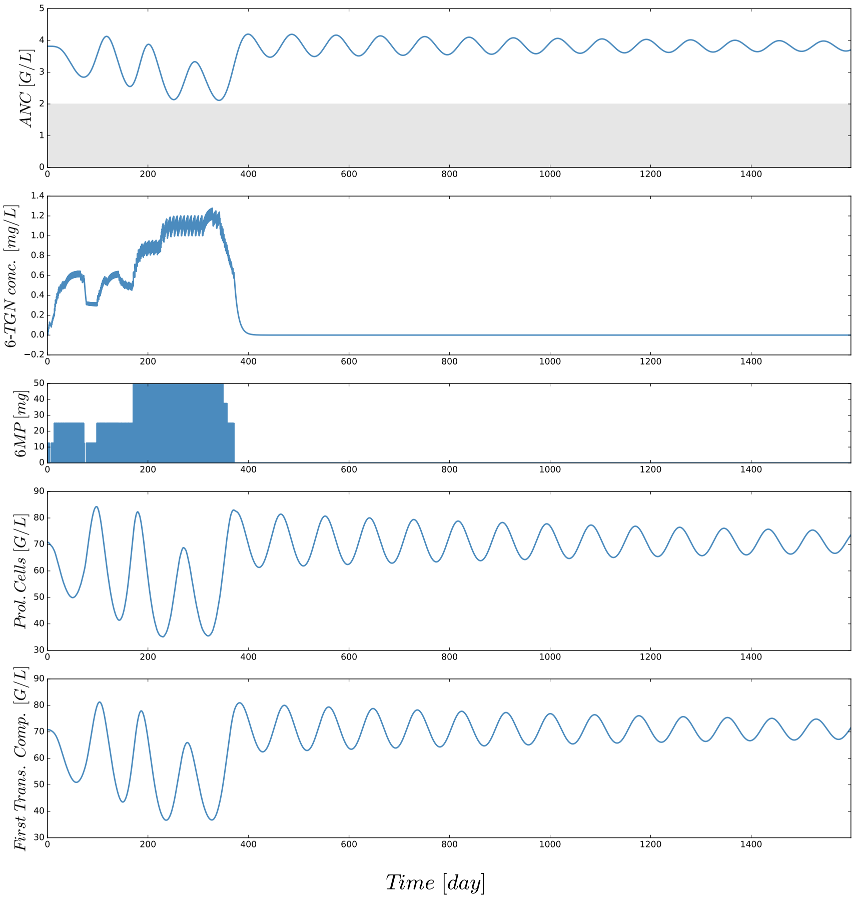

Figure 66: As Figure 1, but for another patient out of 116 patients.

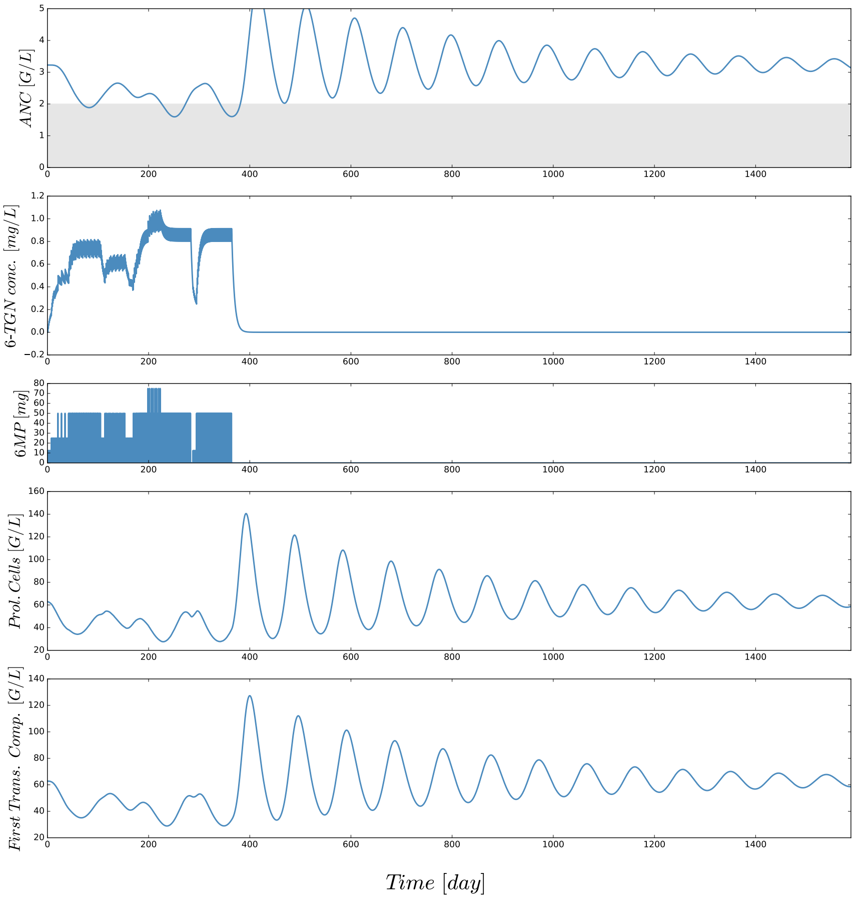

Figure 67: As Figure 1, but for another patient out of 116 patients.

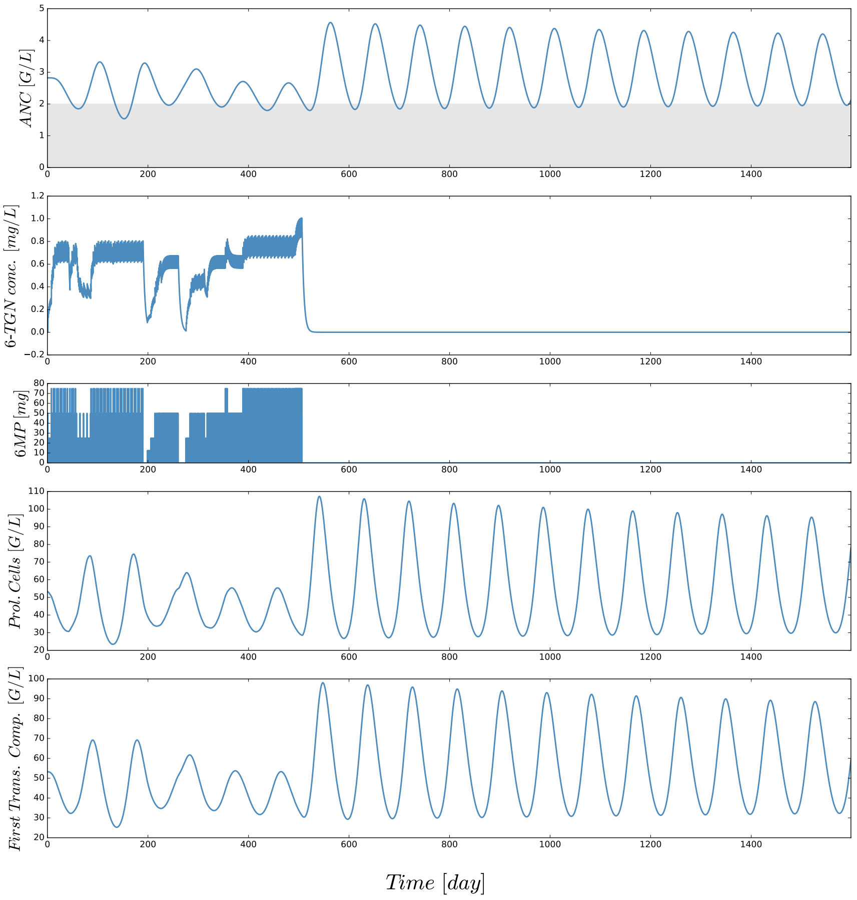

Figure 68: As Figure 1, but for another patient out of 116 patients.

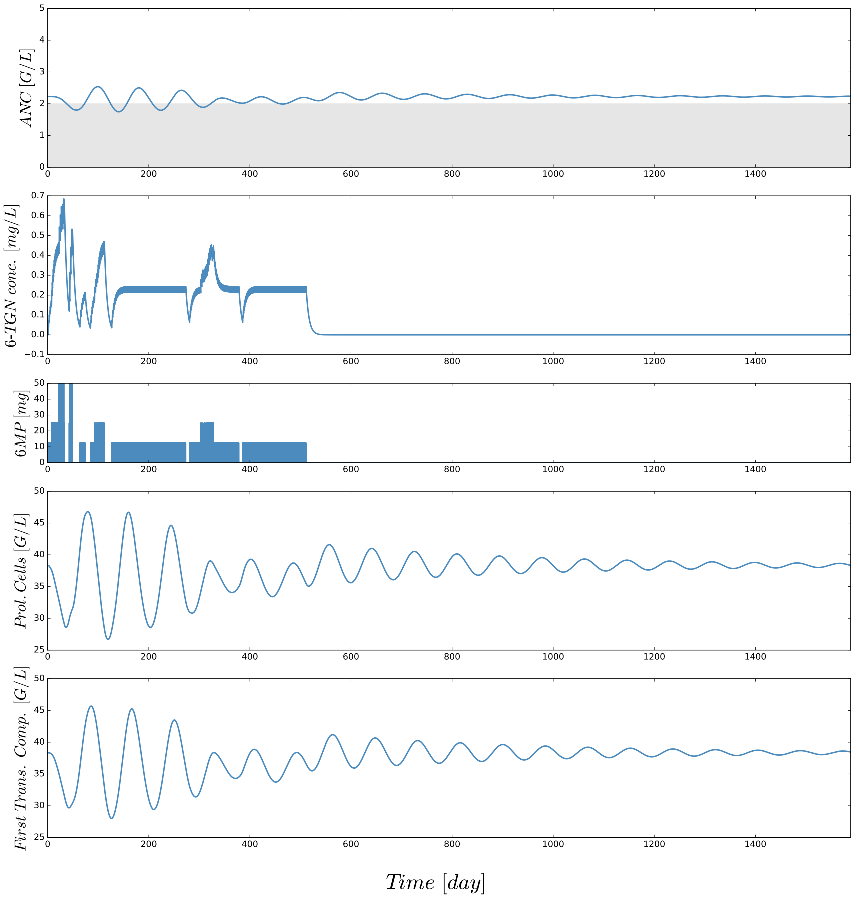

Figure 69: As Figure 1, but for another patient out of 116 patients.

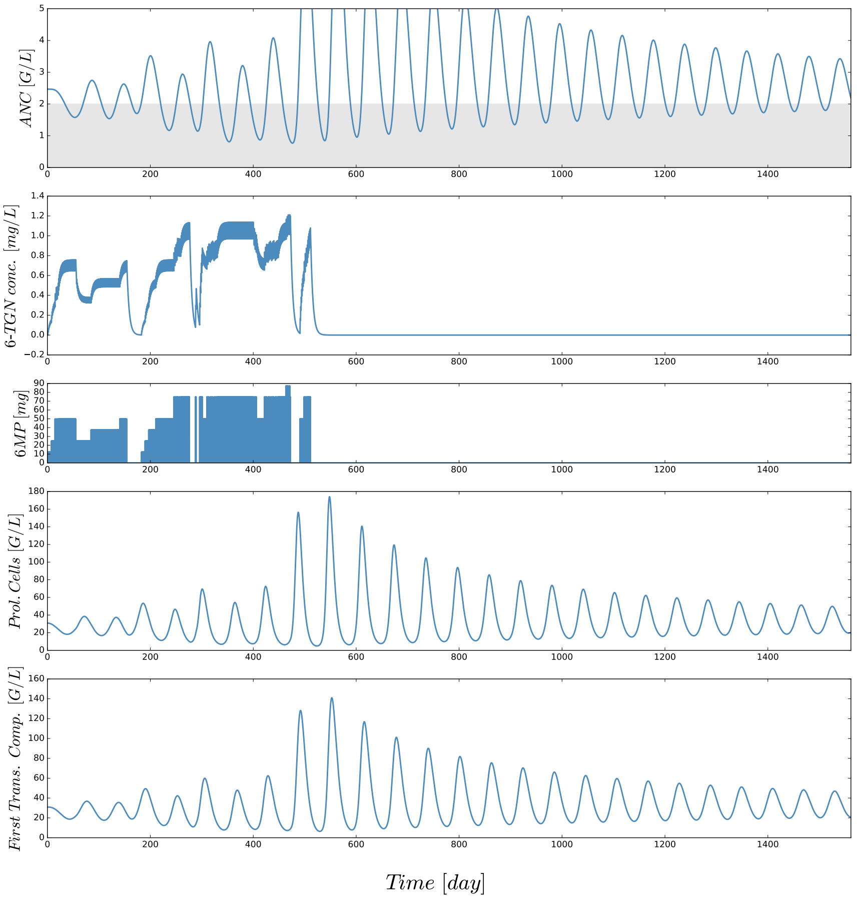

Figure 70: As Figure 1, but for another patient out of 116 patients.

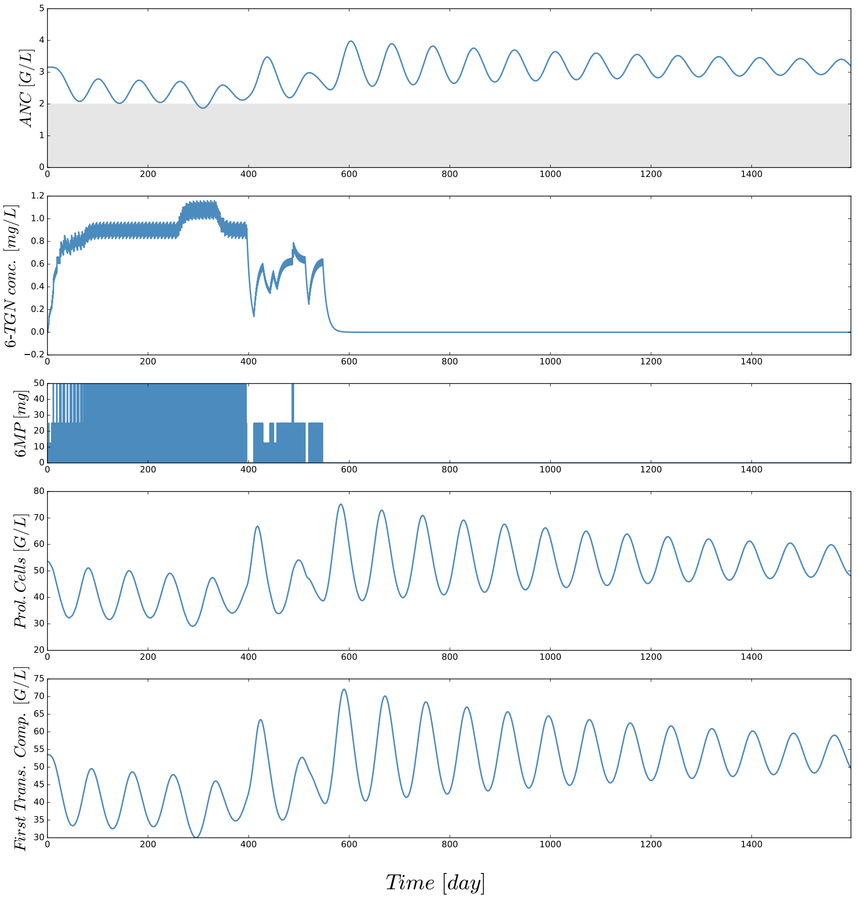

Figure 71: As Figure 1, but for another patient out of 116 patients.

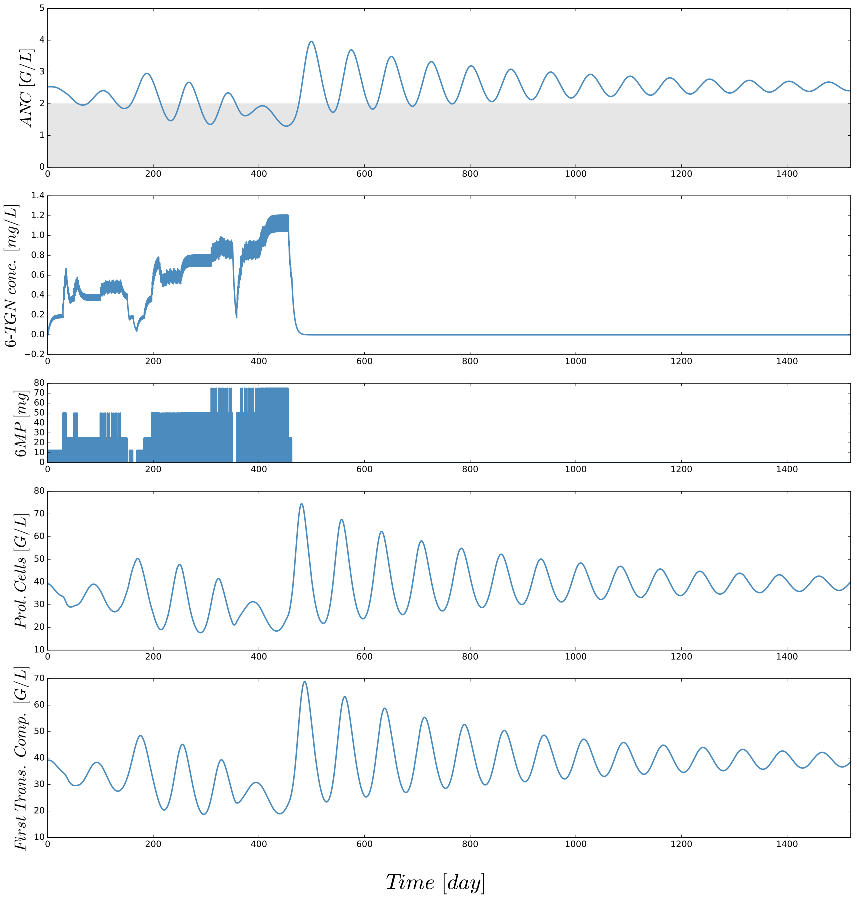

Figure 72: As Figure 1, but for another patient out of 116 patients.

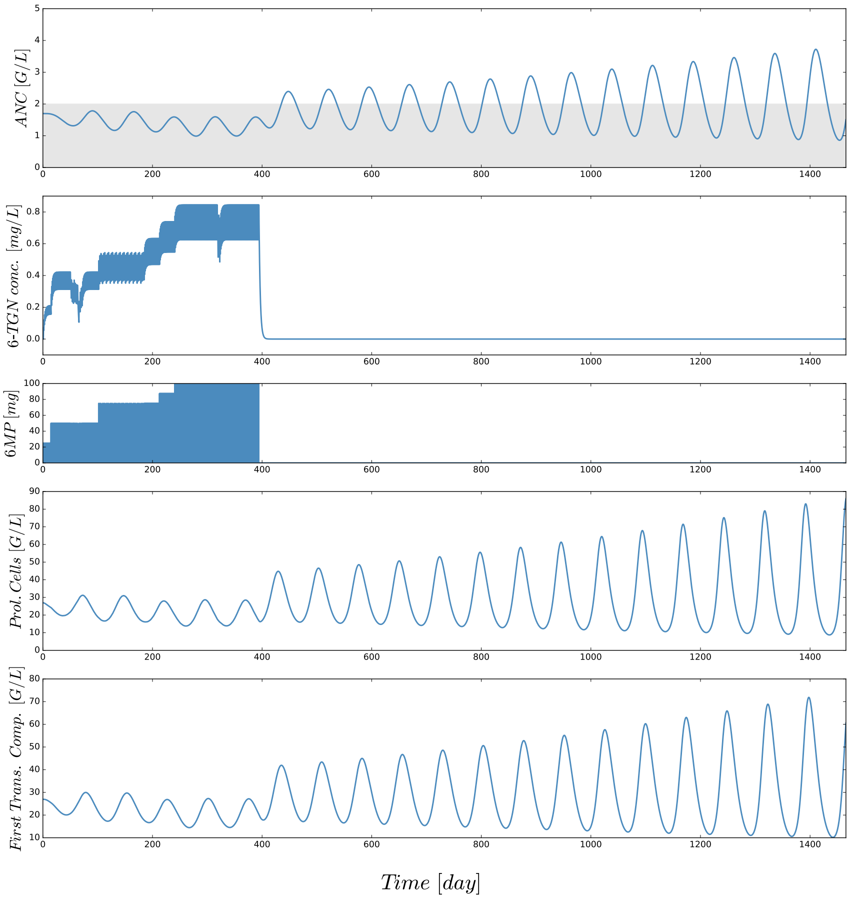

Figure 73: As Figure 1, but for another patient out of 116 patients.

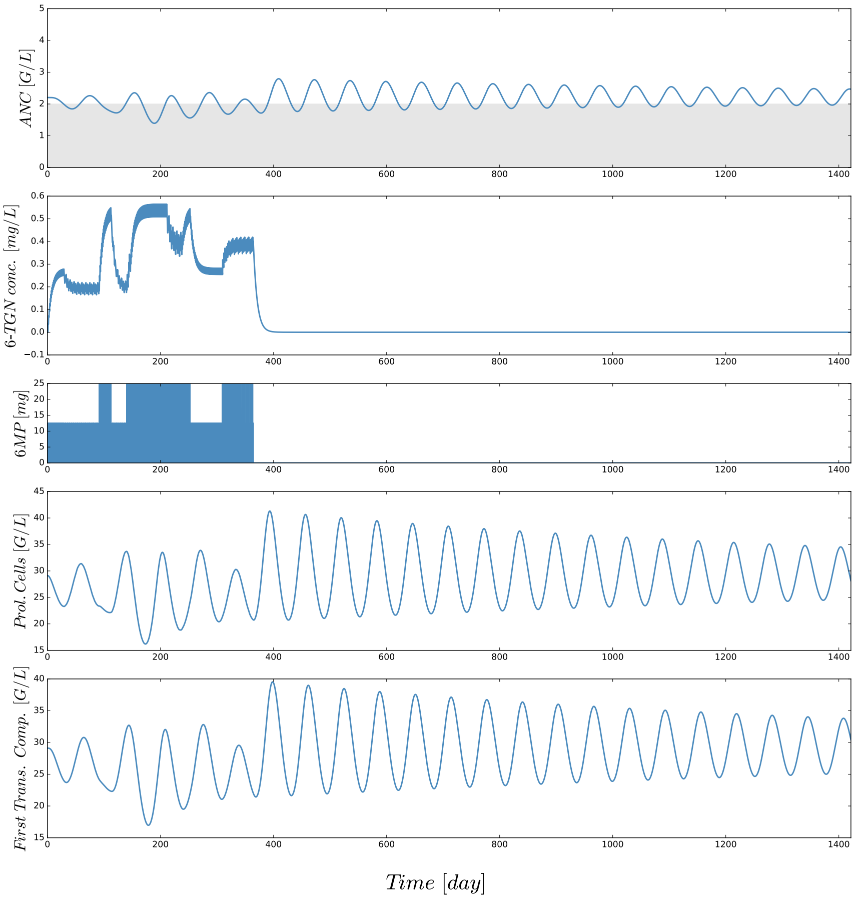

Figure 74: As Figure 1, but for another patient out of 116 patients.

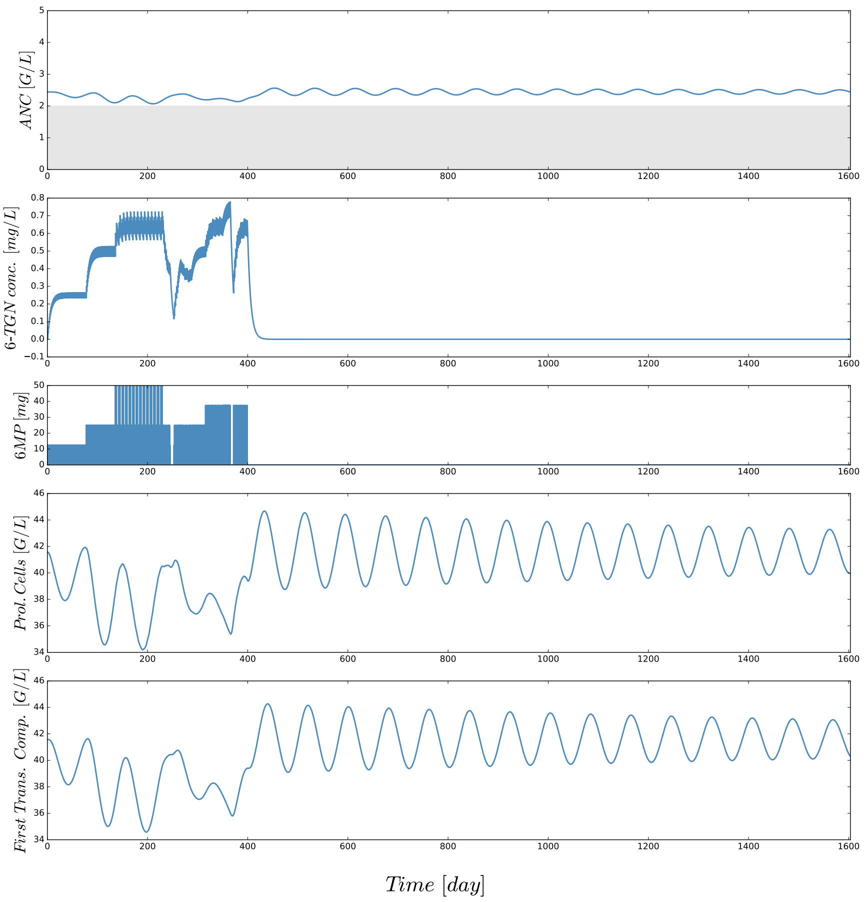

Figure 75: As Figure 1, but for another patient out of 116 patients.

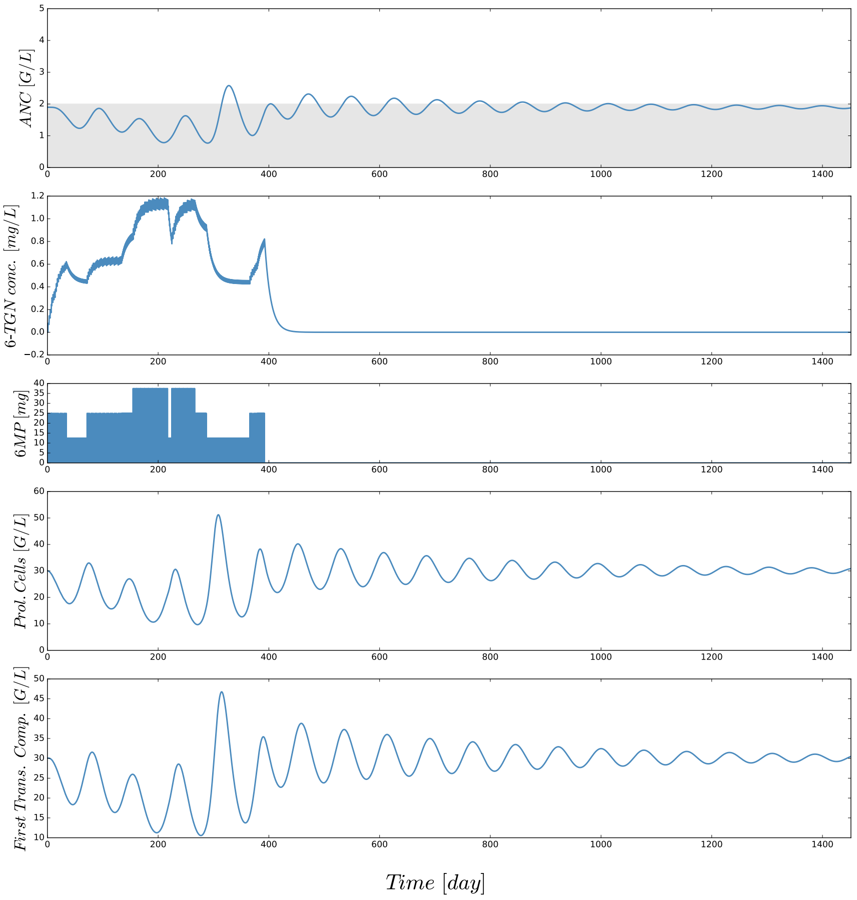

Figure 76: As Figure 1, but for another patient out of 116 patients.

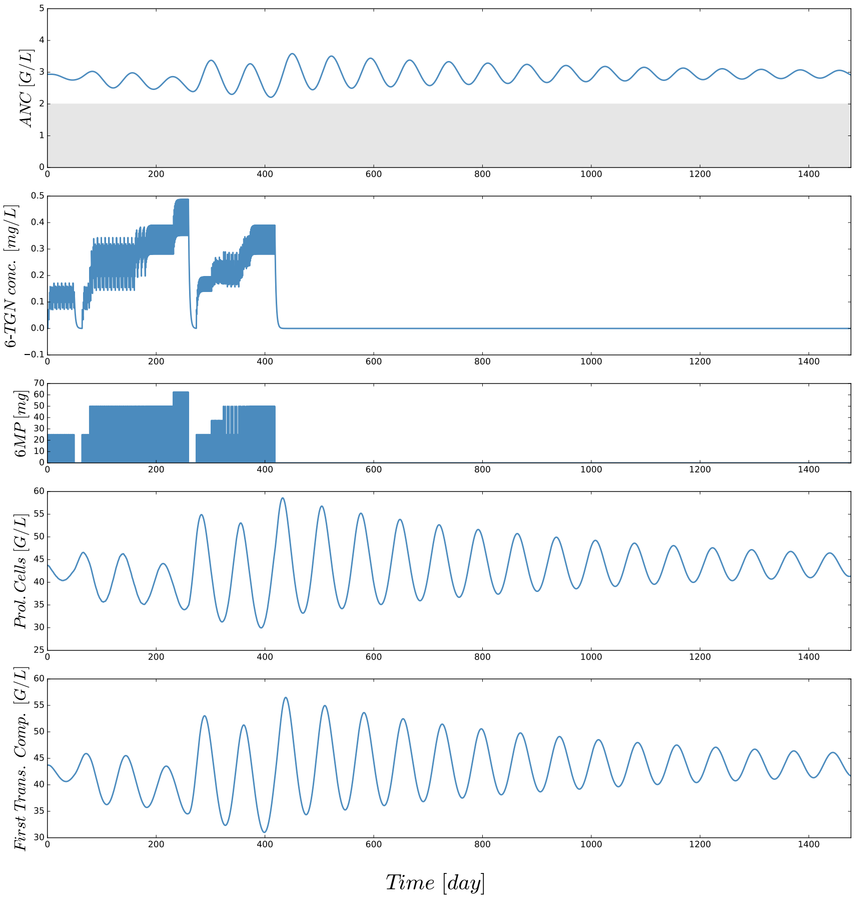

Figure 77: As Figure 1, but for another patient out of 116 patients.

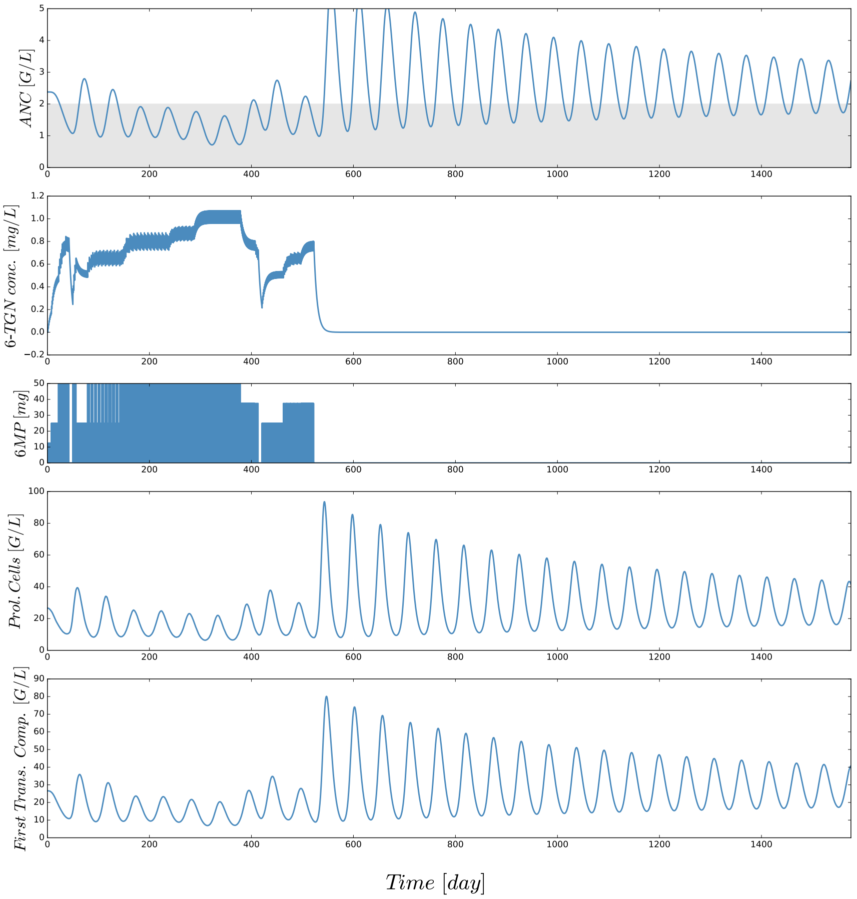

Figure 78: As Figure 1, but for another patient out of 116 patients.

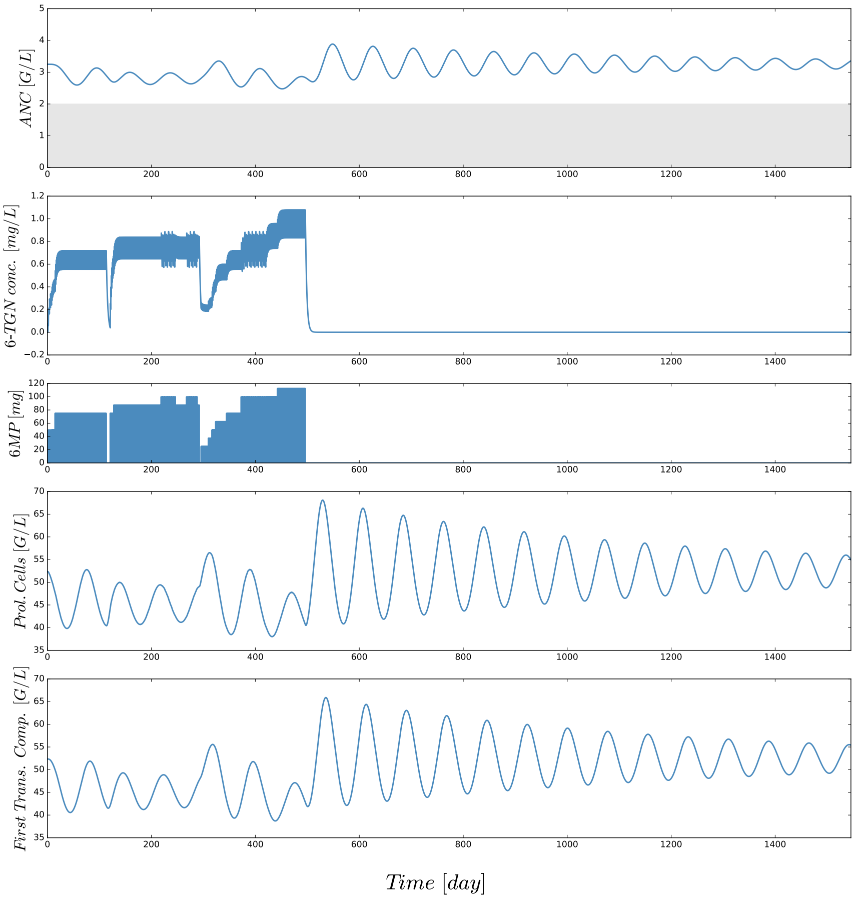

Figure 79: As Figure 1, but for another patient out of 116 patients.

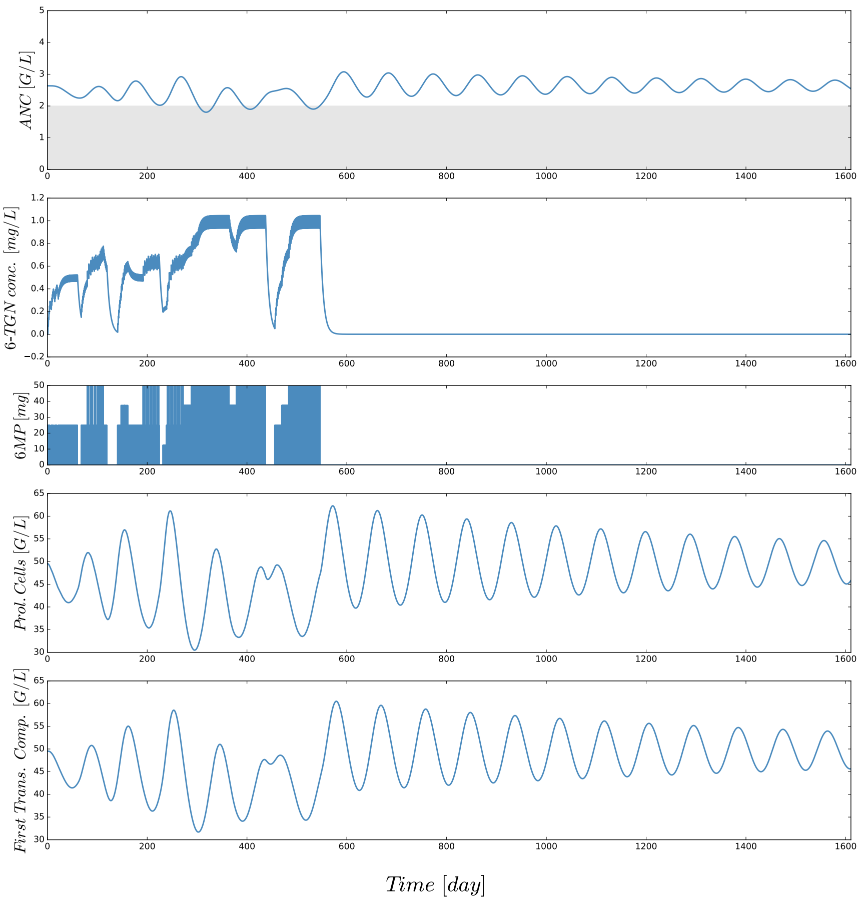

Figure 80: As Figure 1, but for another patient out of 116 patients.

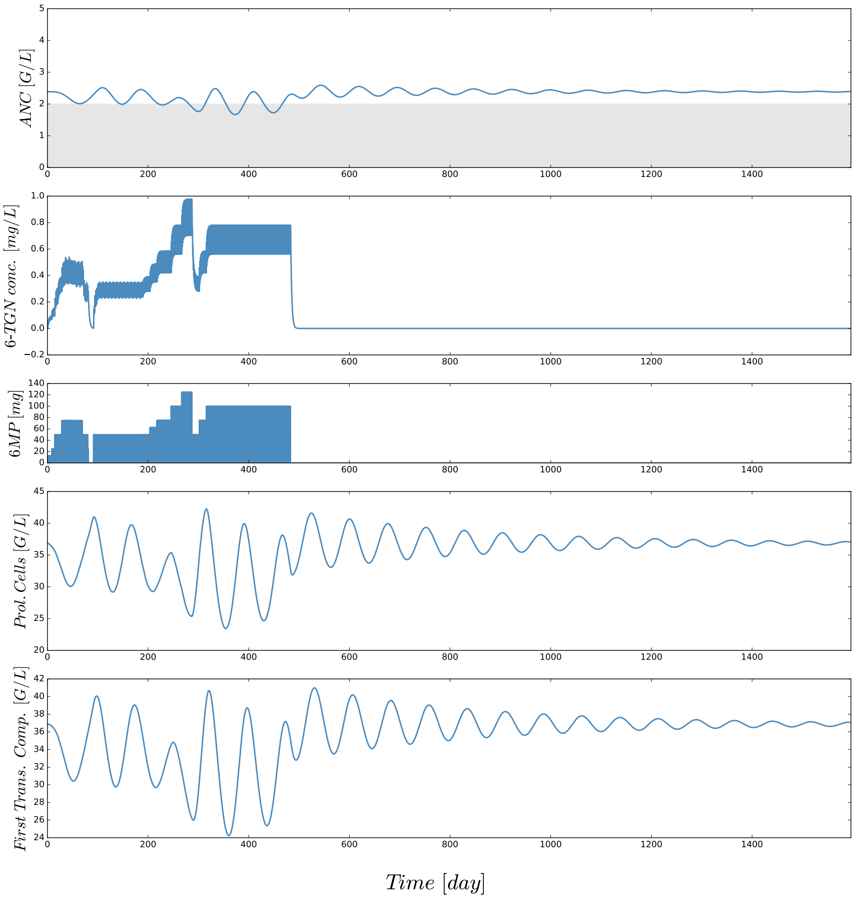

Figure 81: As Figure 1, but for another patient out of 116 patients.

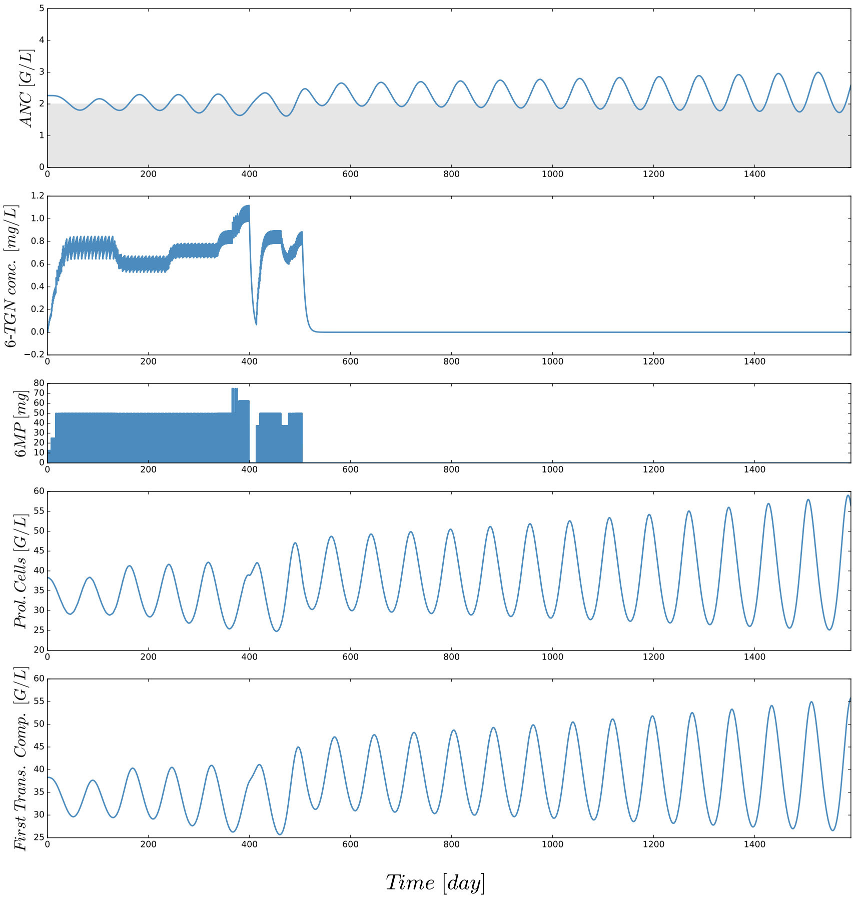

Figure 82: As Figure 1, but for another patient out of 116 patients.

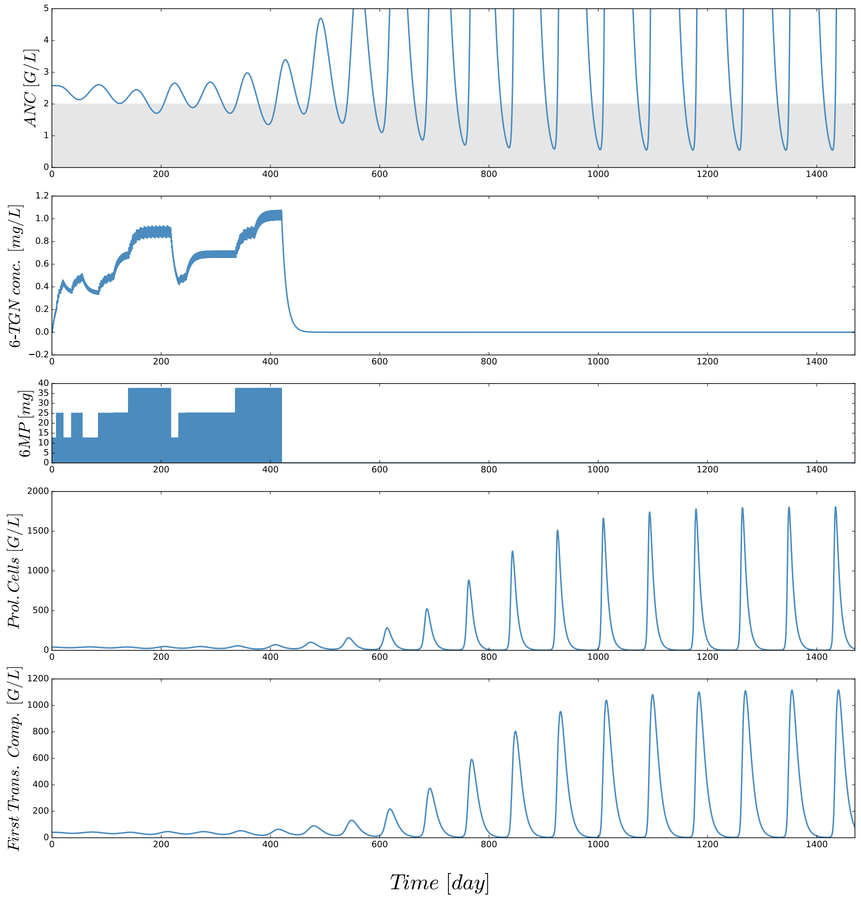

Figure 83: As Figure 1, but for another patient out of 116 patients.

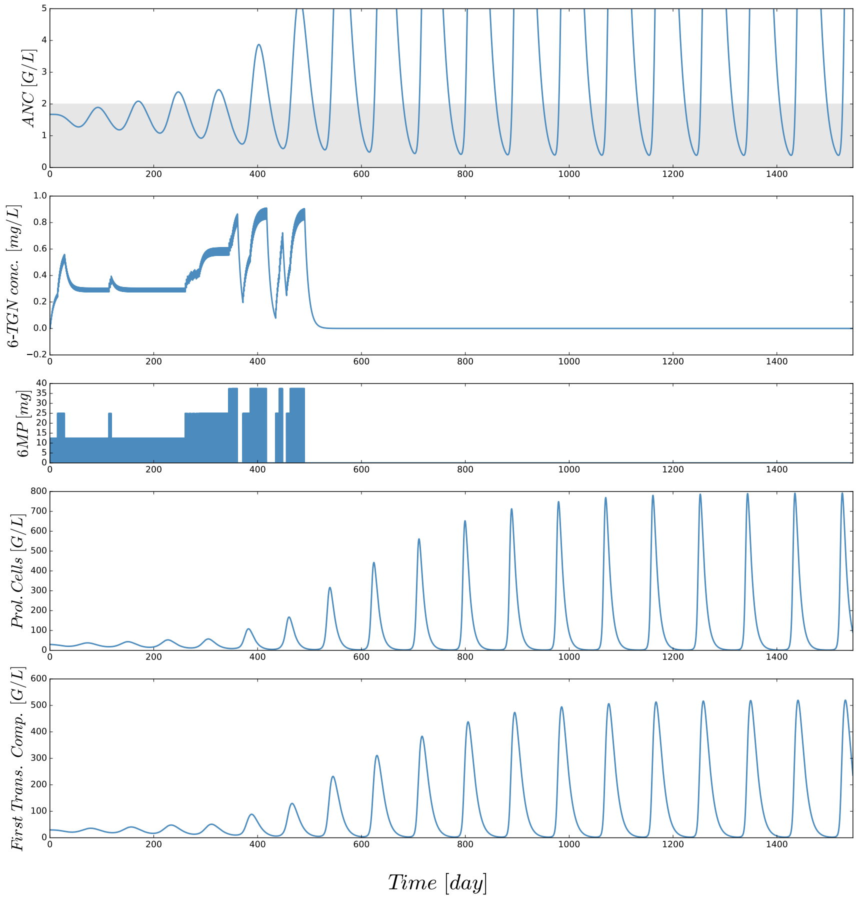

Figure 84: As Figure 1, but for another patient out of 116 patients.

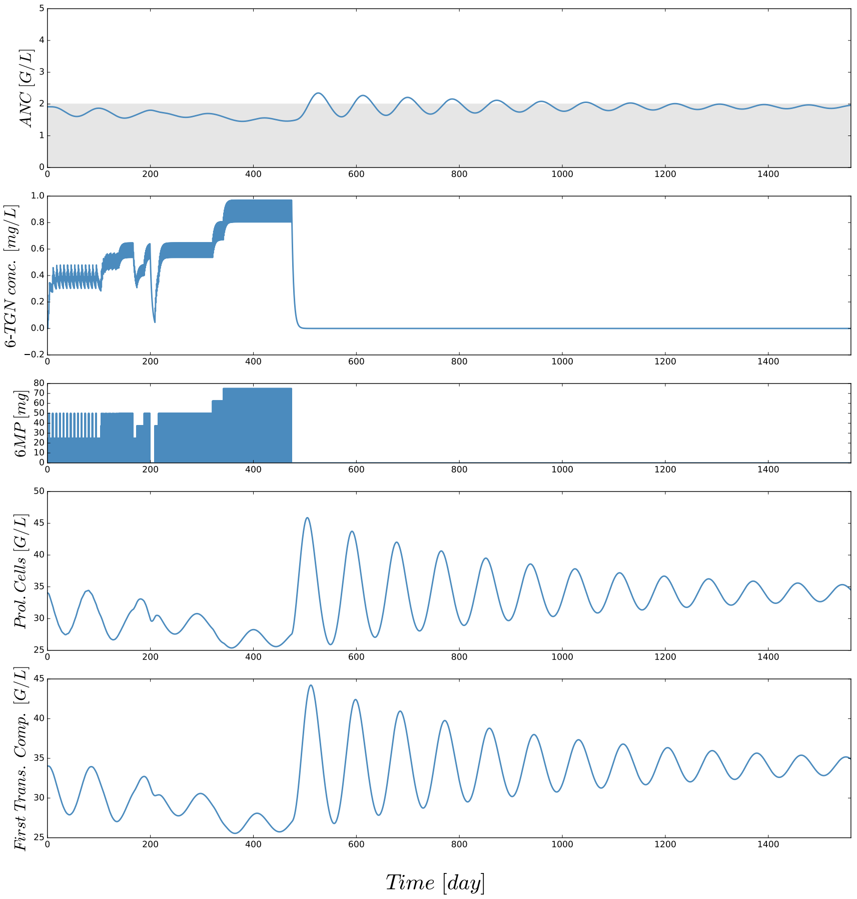

Figure 85: As Figure 1, but for another patient out of 116 patients.

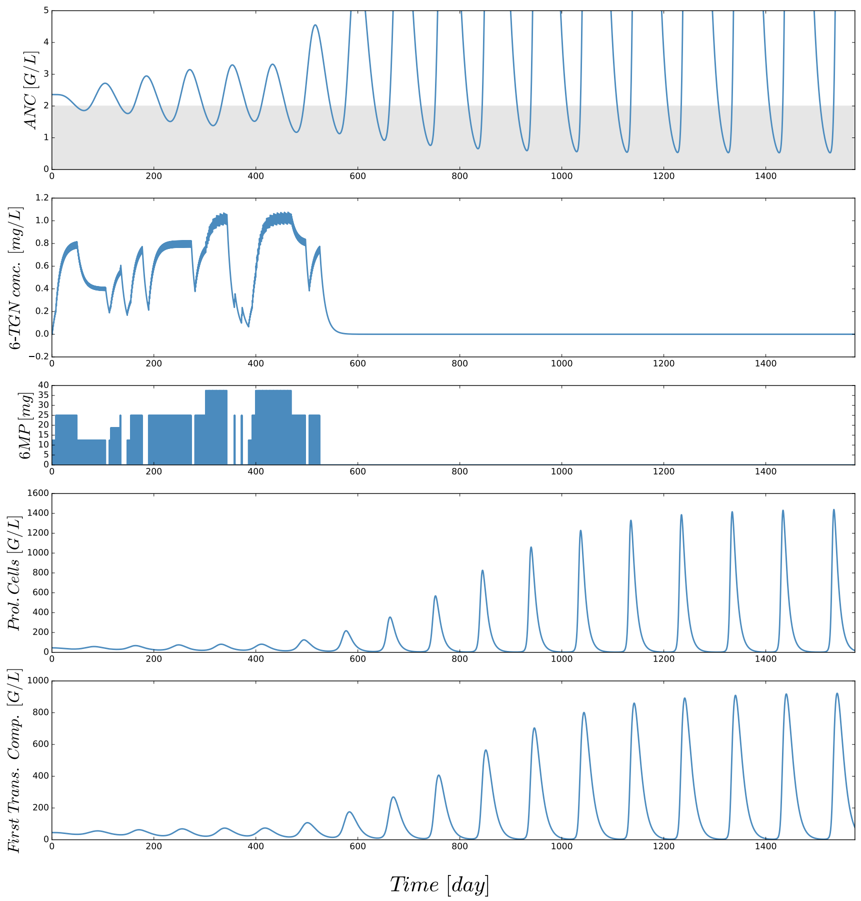

Figure 86: As Figure 1, but for another patient out of 116 patients.

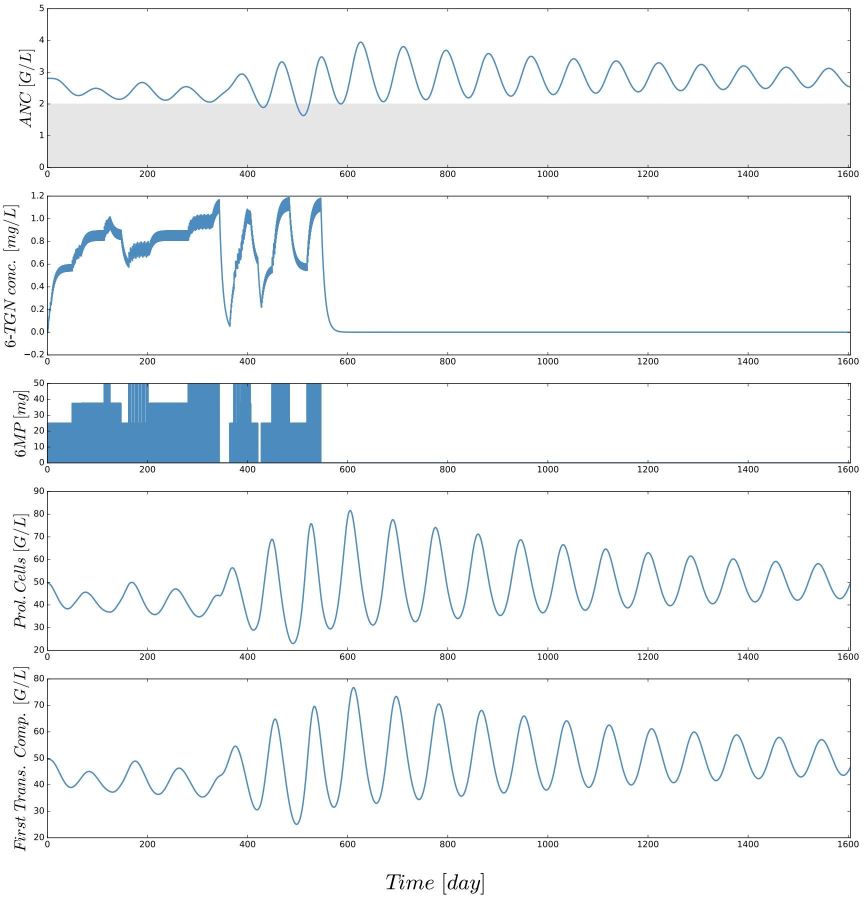

Figure 87: As Figure 1, but for another patient out of 116 patients.

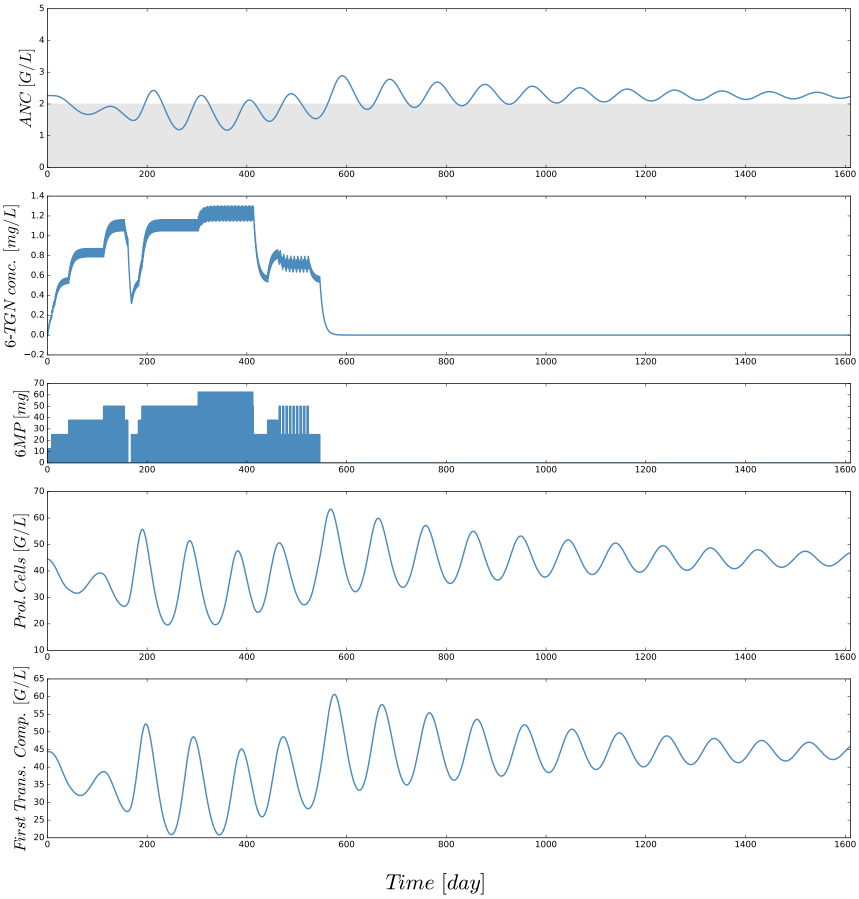

Figure 88: As Figure 1, but for another patient out of 116 patients.

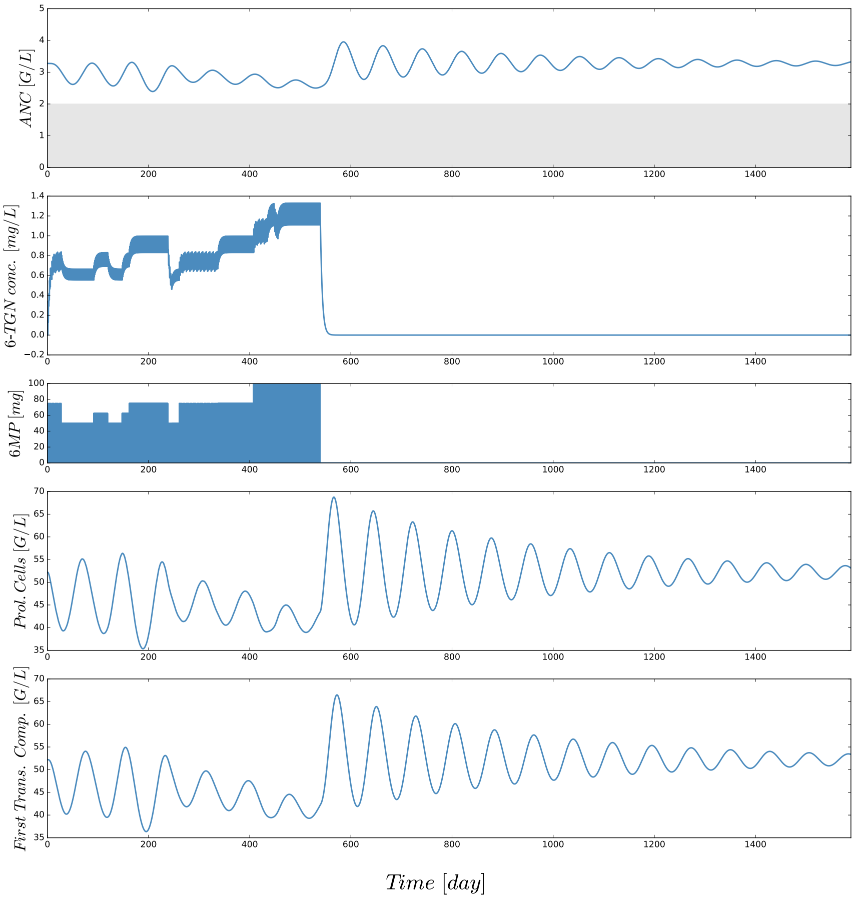

Figure 89: As Figure 1, but for another patient out of 116 patients.

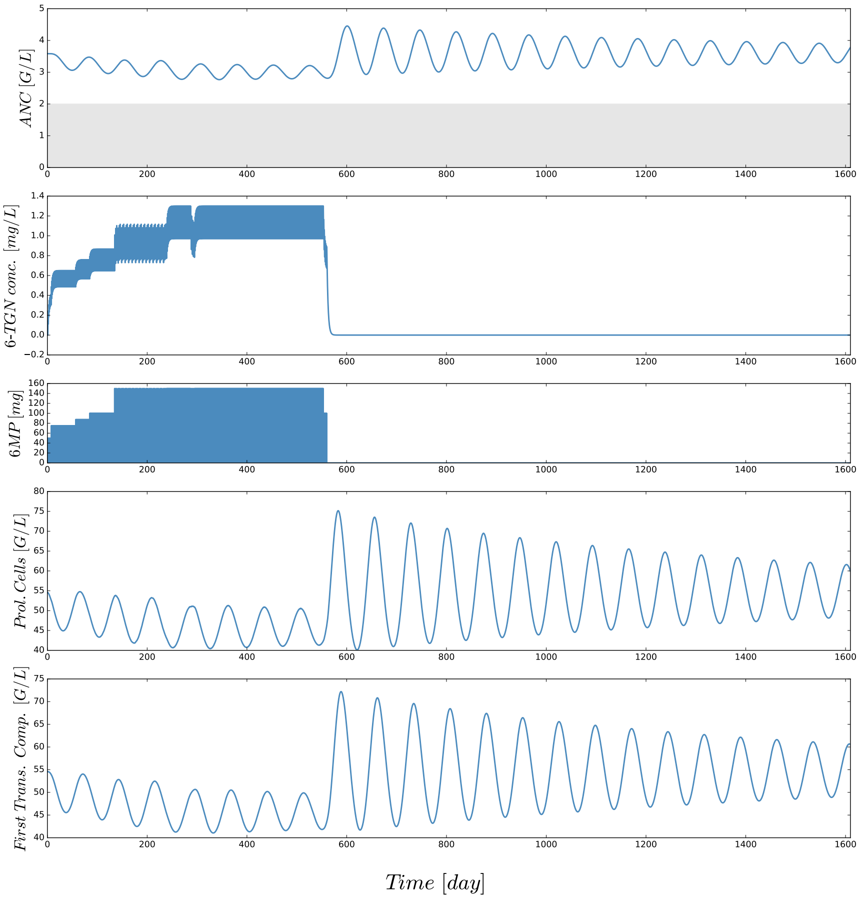

Figure 90: As Figure 1, but for another patient out of 116 patients.

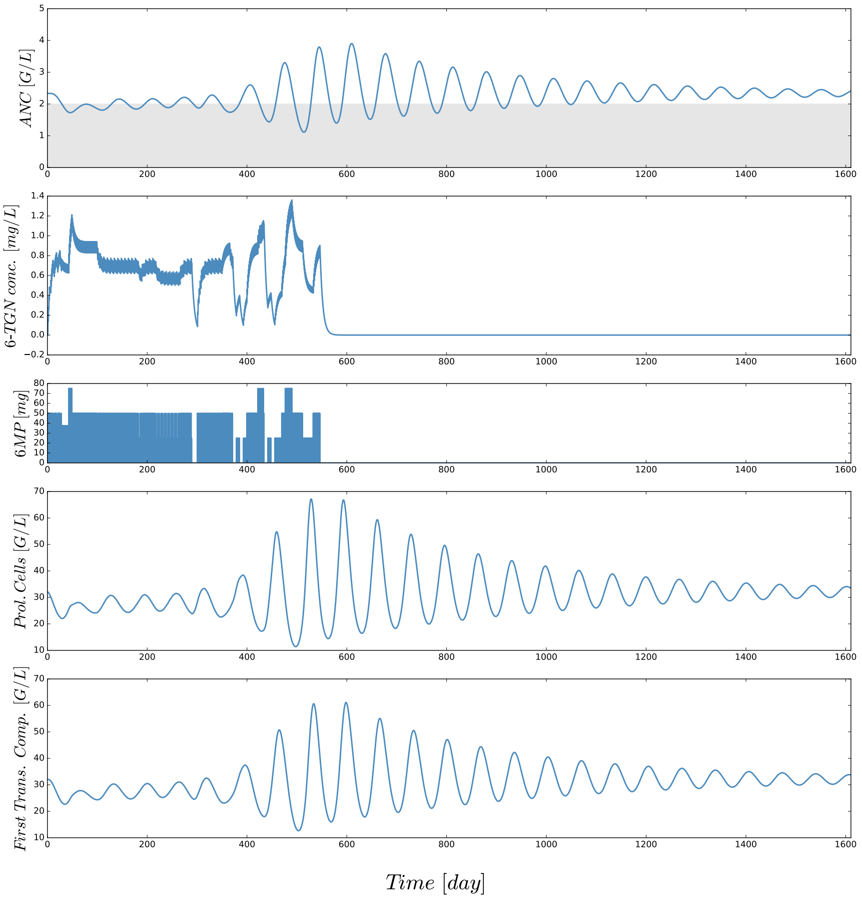

Figure 91: As Figure 1, but for another patient out of 116 patients.

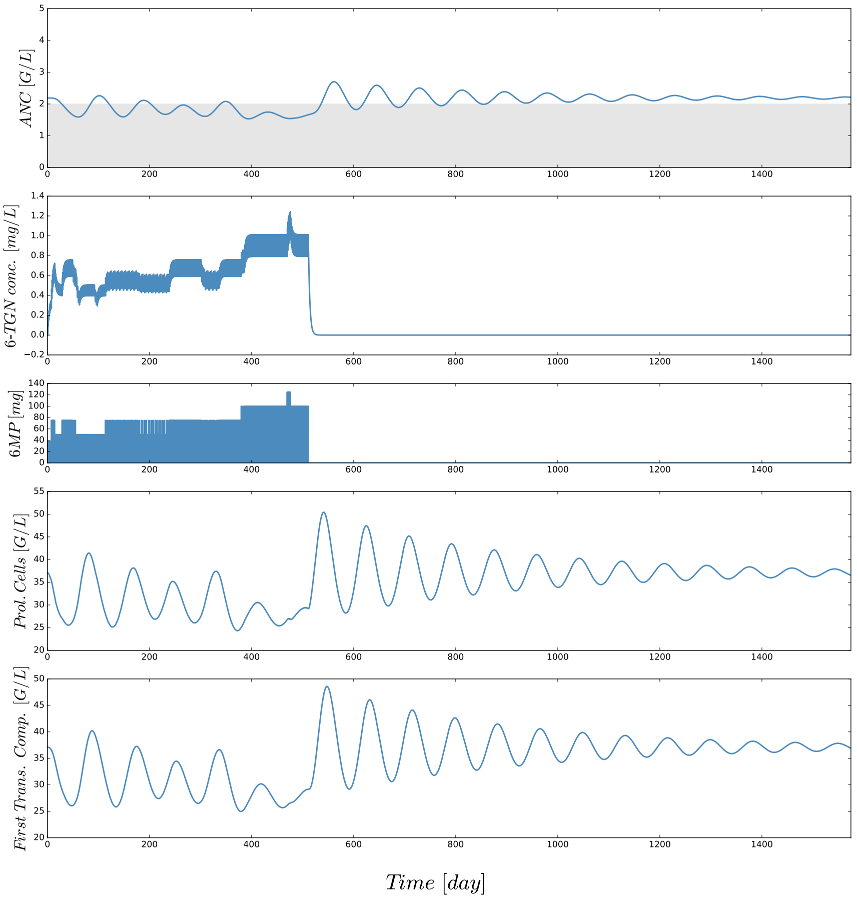

Figure 92: As Figure 1, but for another patient out of 116 patients.

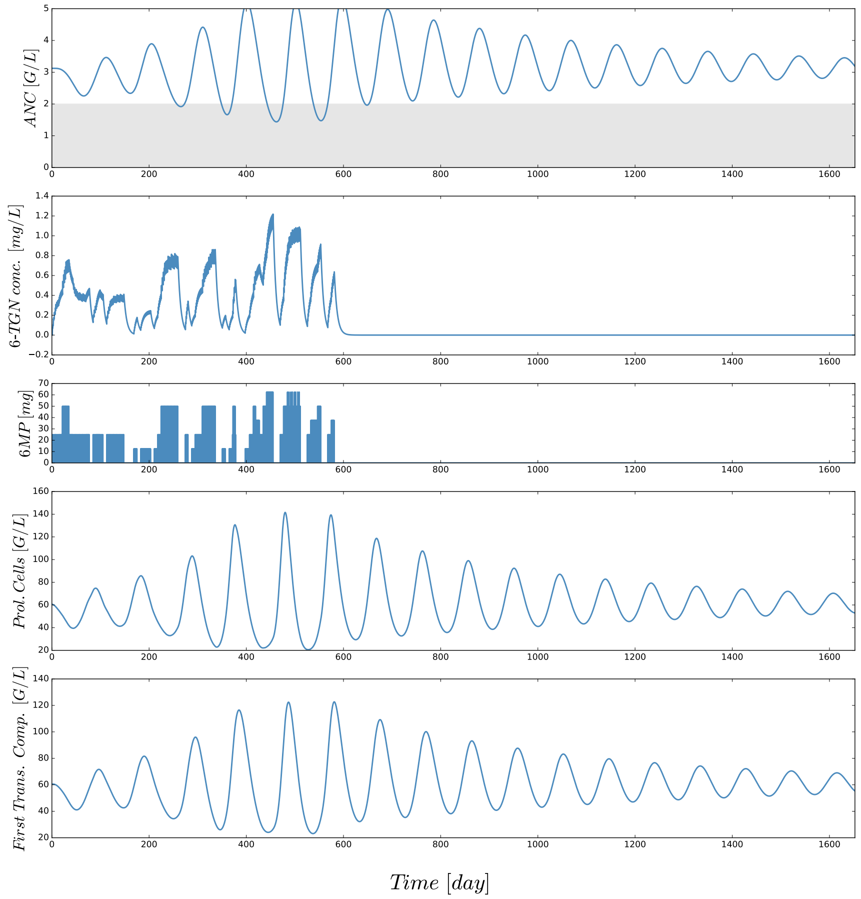

Figure 93: As Figure 1, but for another patient out of 116 patients.

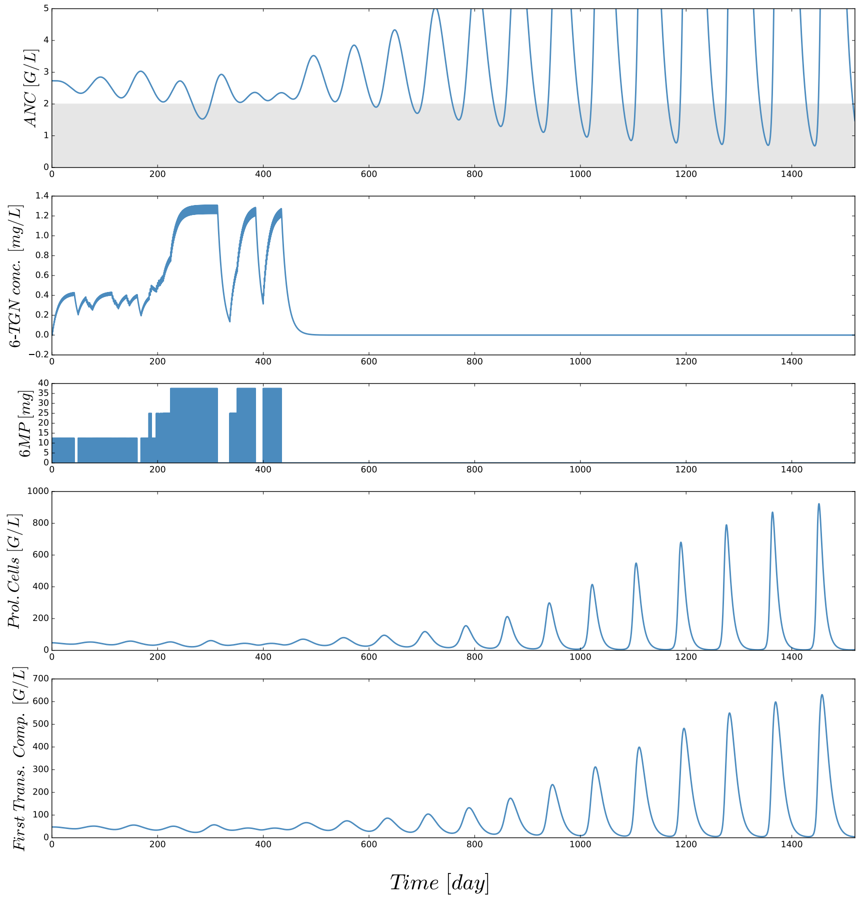

Figure 94: As Figure 1, but for another patient out of 116 patients.

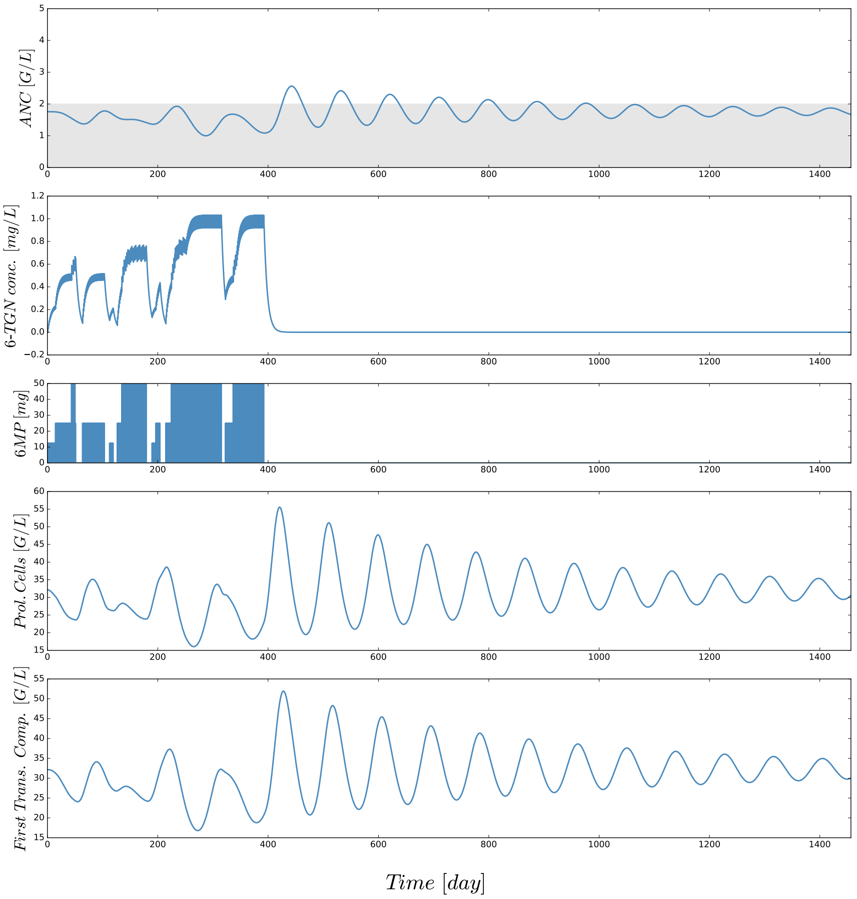

Figure 95: As Figure 1, but for another patient out of 116 patients.

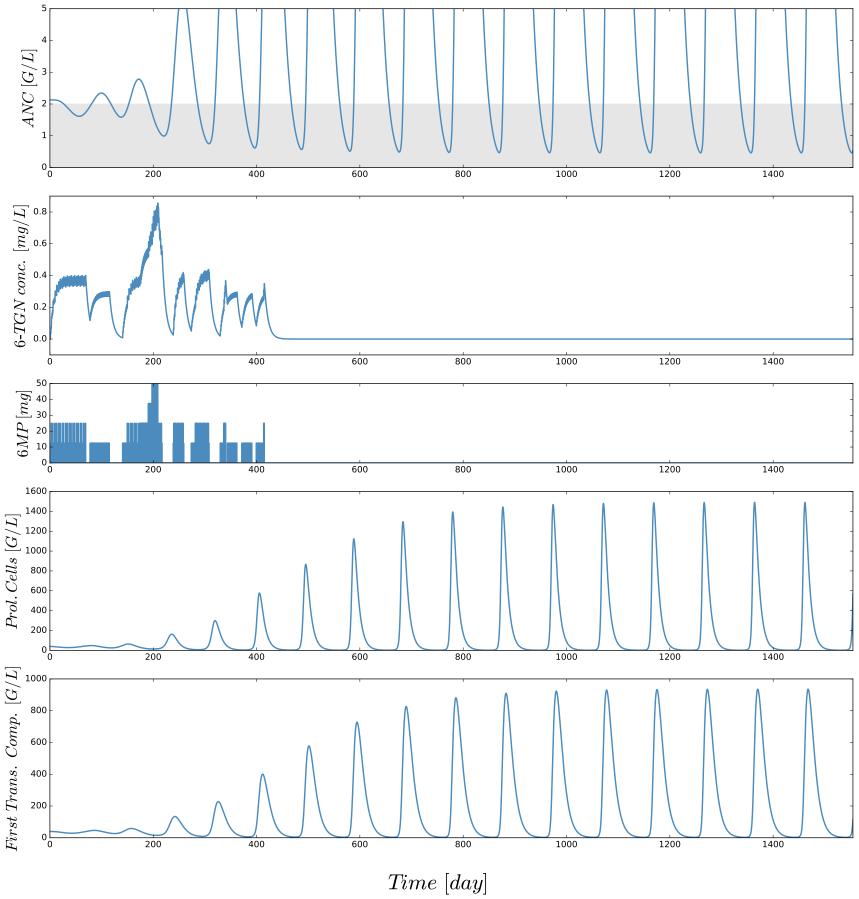

Figure 96: As Figure 1, but for another patient out of 116 patients.

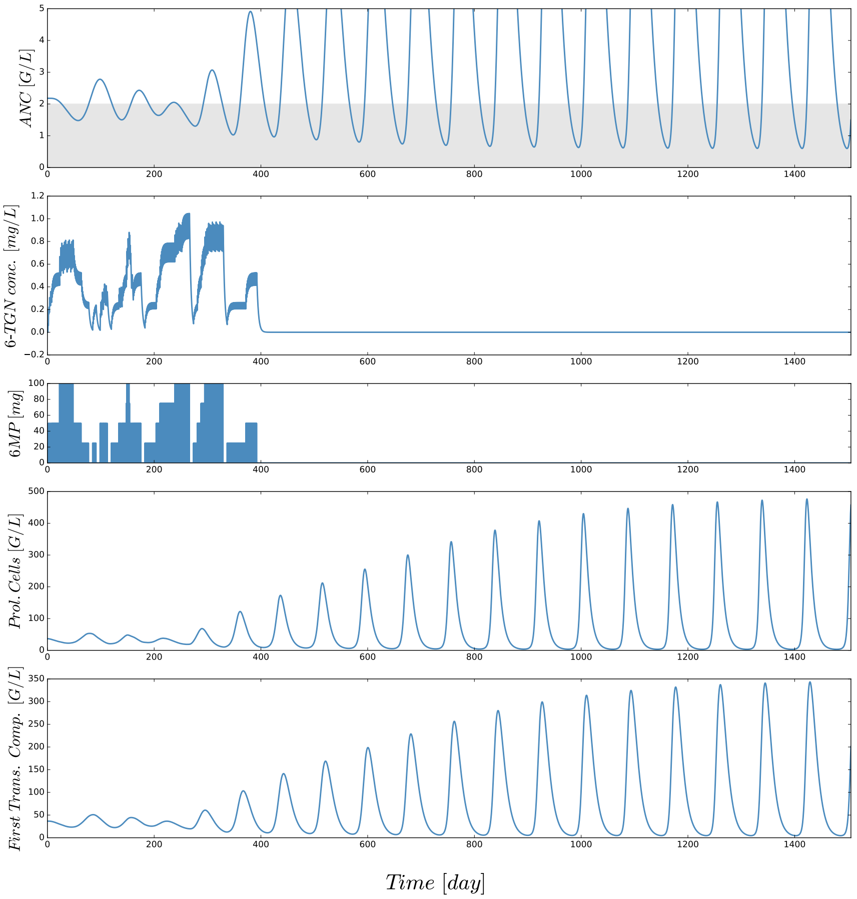

Figure 97: As Figure 1, but for another patient out of 116 patients.

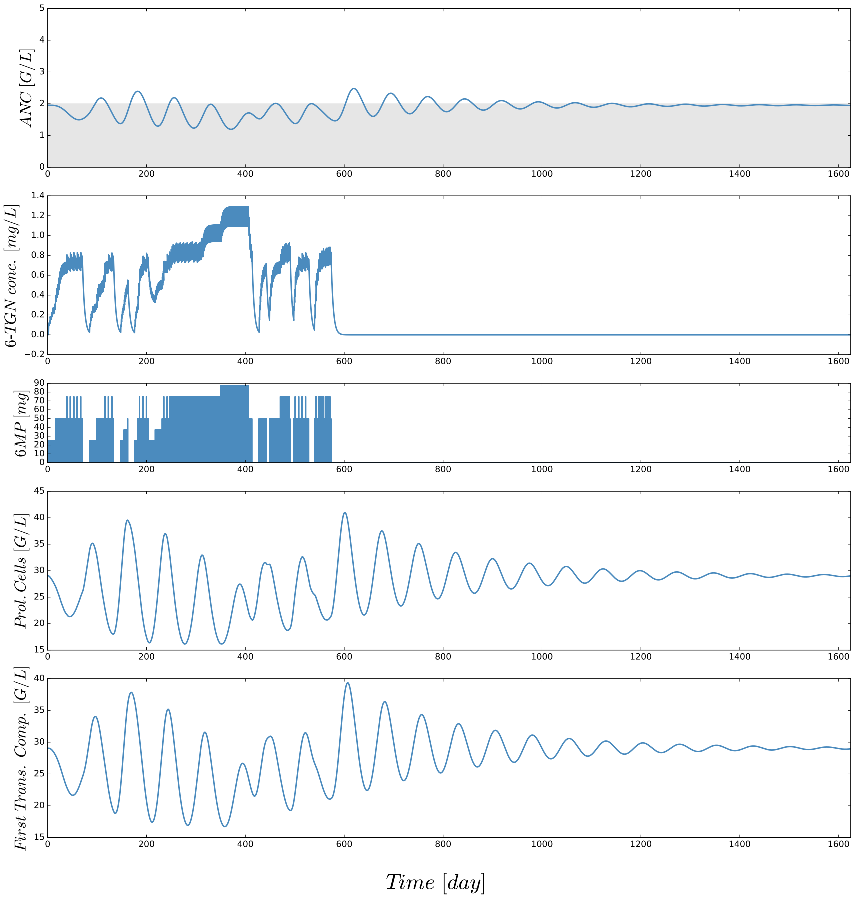

Figure 98: As Figure 1, but for another patient out of 116 patients.

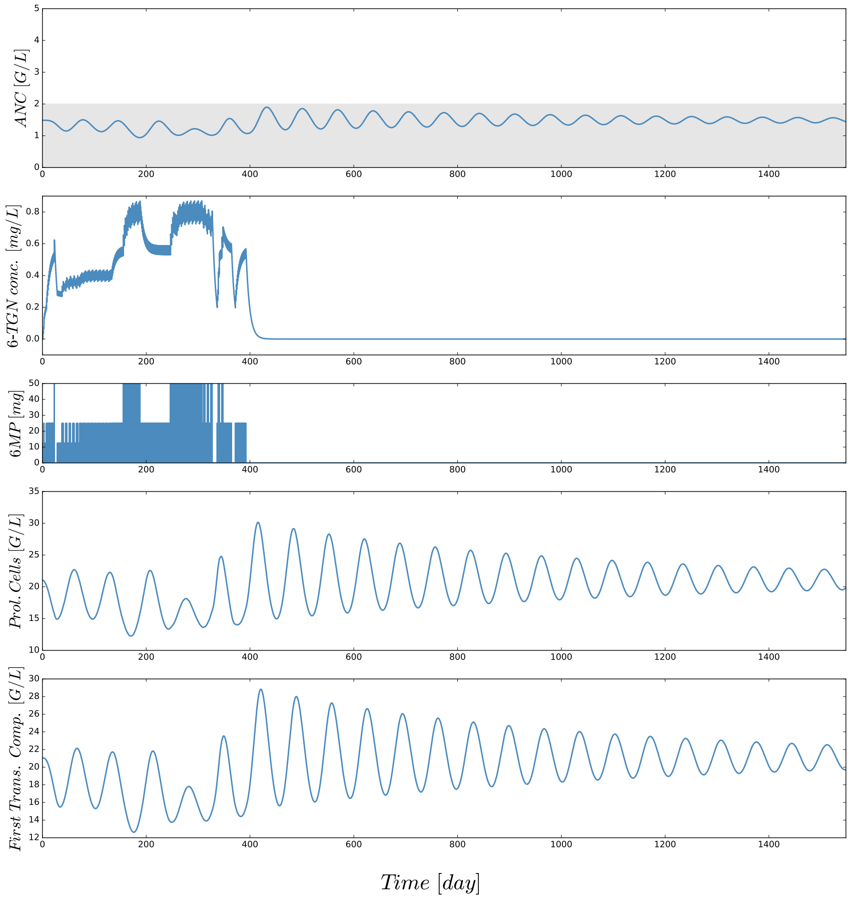

Figure 99: As Figure 1, but for another patient out of 116 patients.

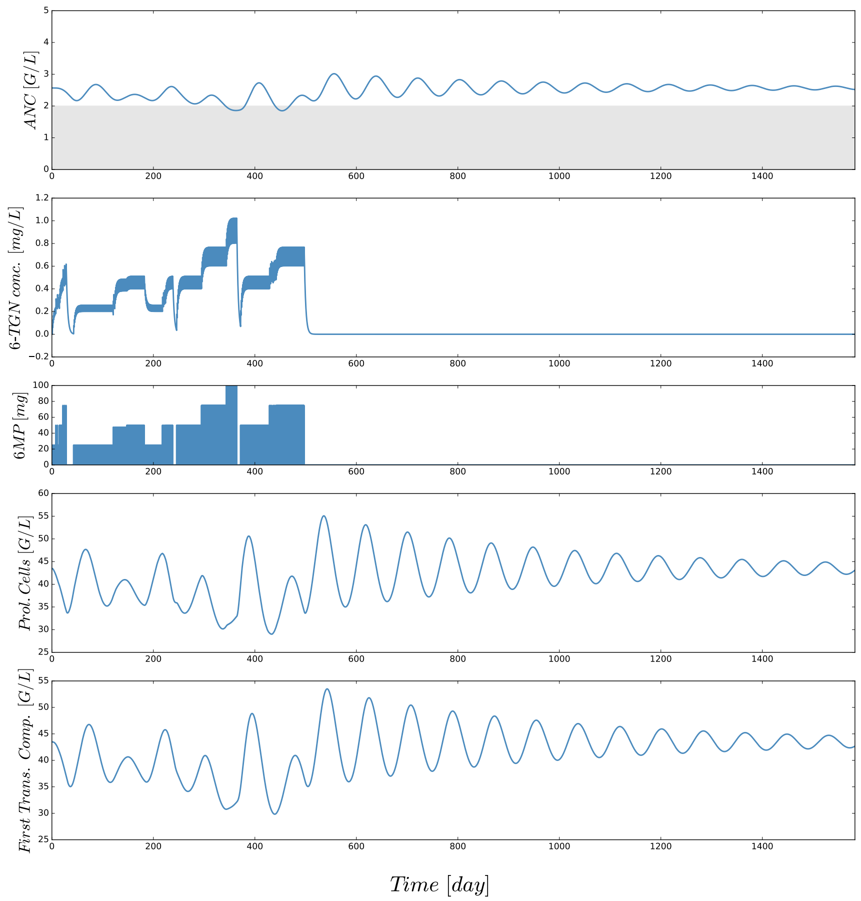

Figure 100: As Figure 1, but for another patient out of 116 patients.

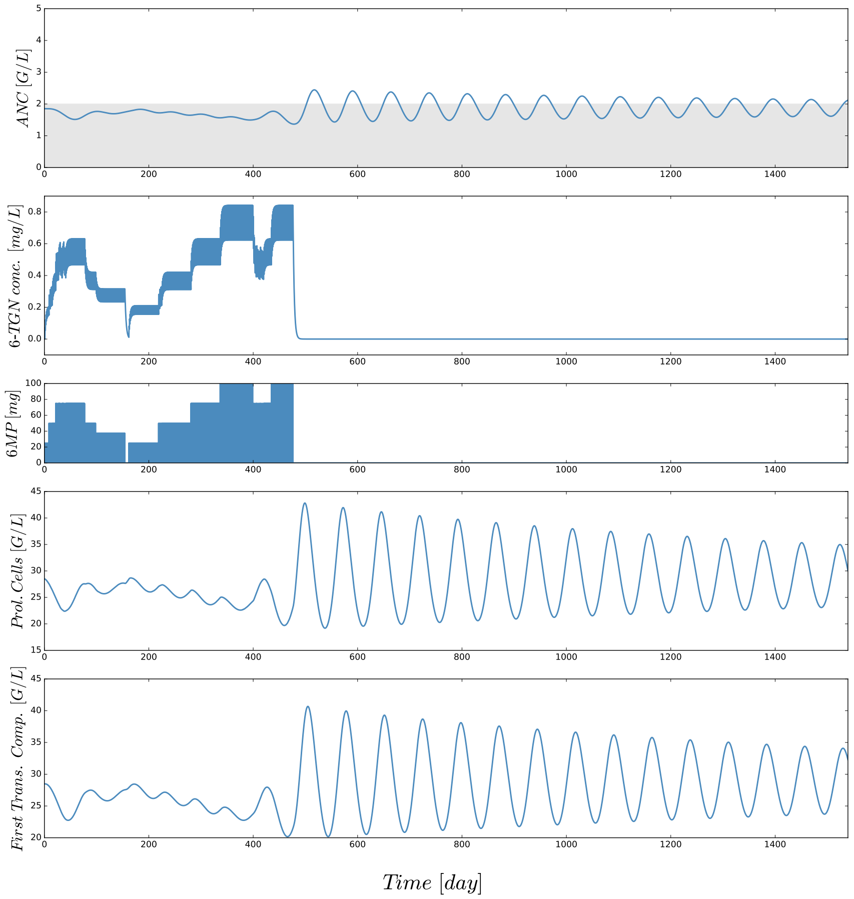

Figure 101: As Figure 1, but for another patient out of 116 patients.

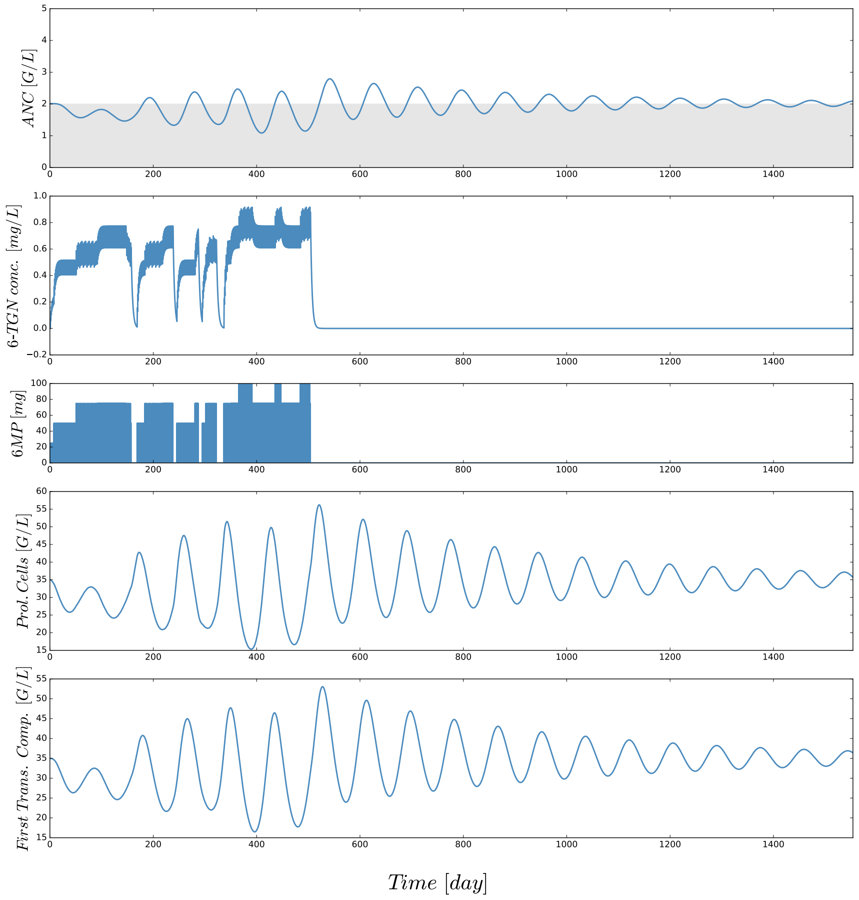

Figure 102: As Figure 1, but for another patient out of 116 patients.

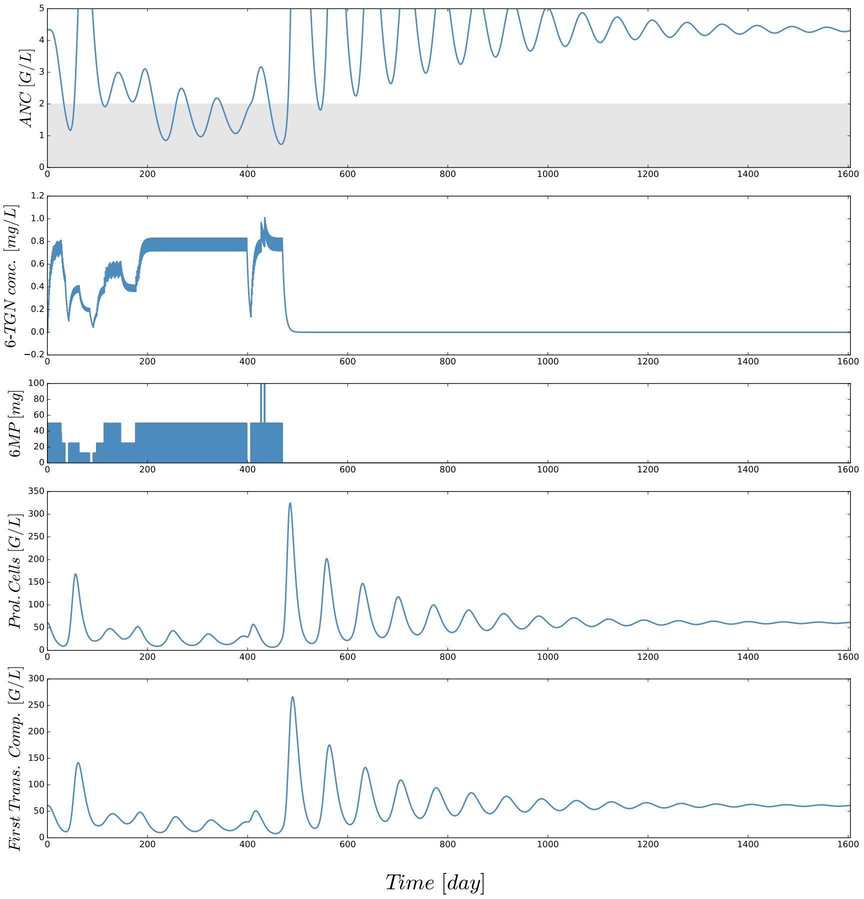

Figure 103: As Figure 1, but for another patient out of 116 patients.

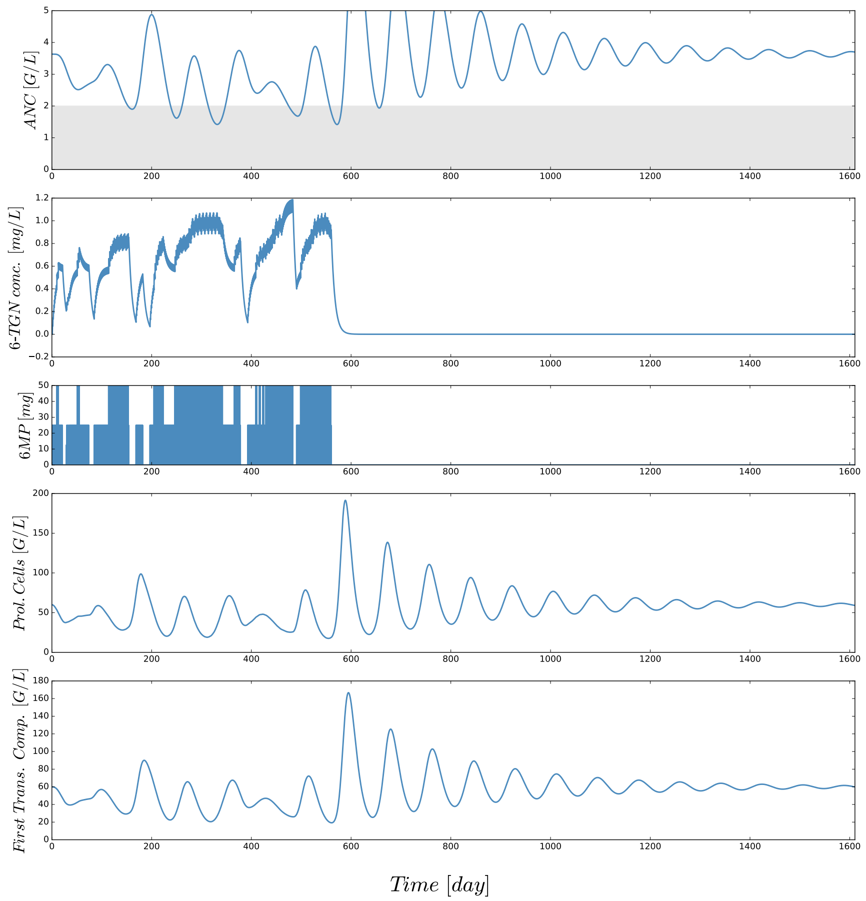

Figure 104: As Figure 1, but for another patient out of 116 patients.

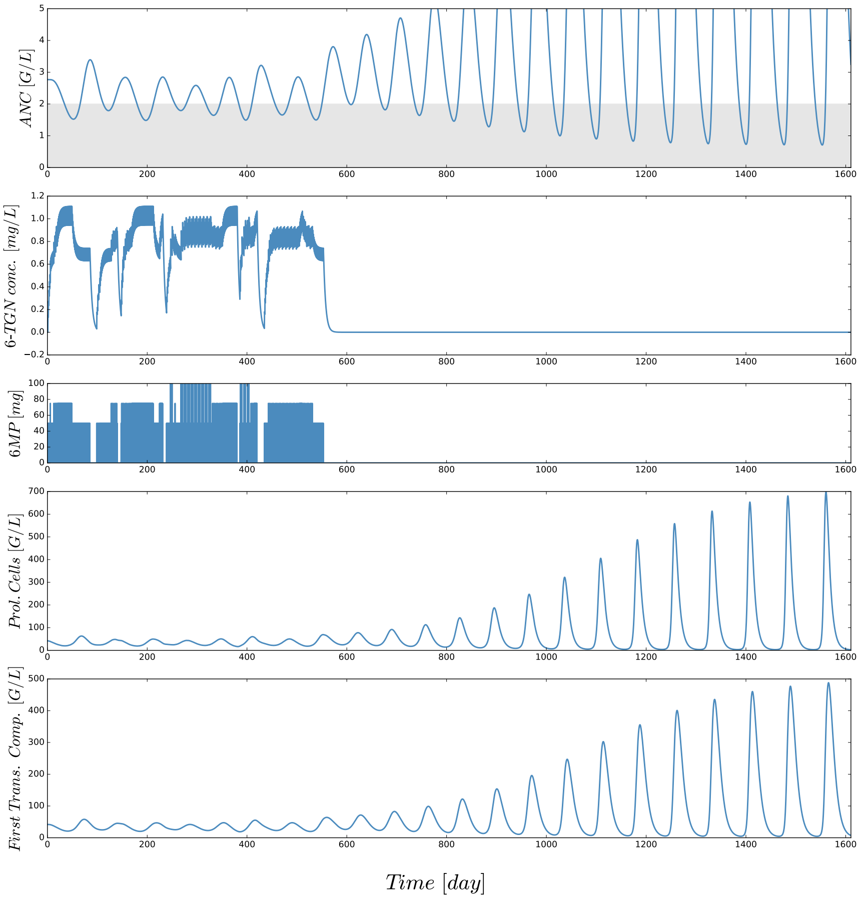

Figure 105: As Figure 1, but for another patient out of 116 patients.

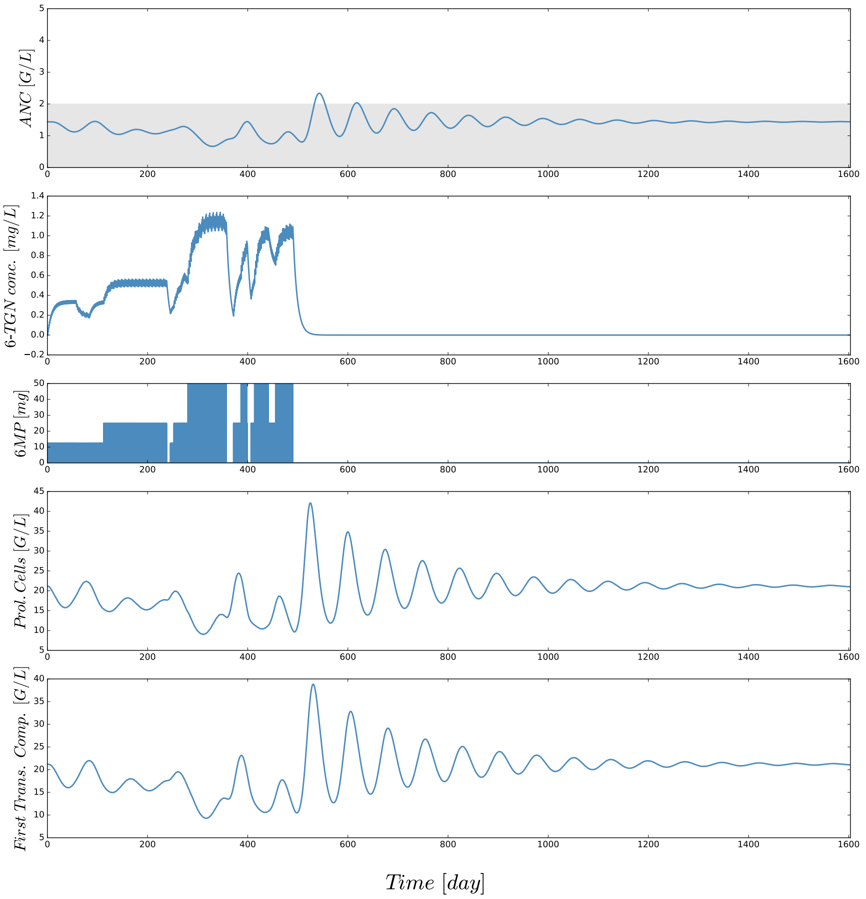

Figure 106: As Figure 1, but for another patient out of 116 patients.

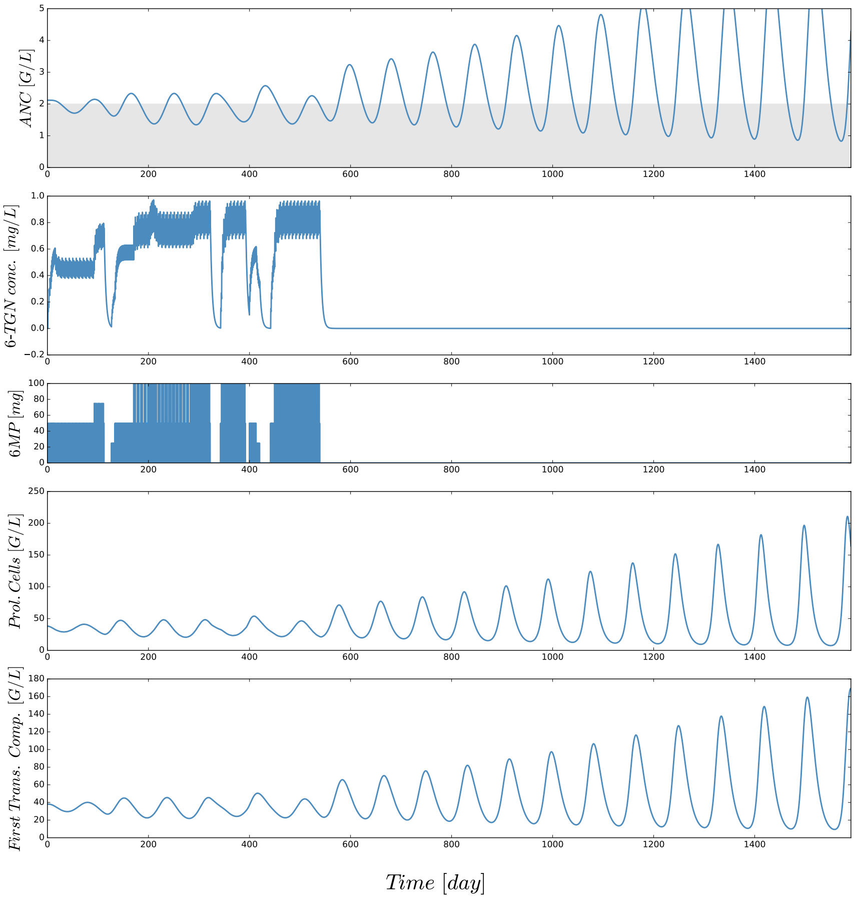

Figure 107: As Figure 1, but for another patient out of 116 patients.

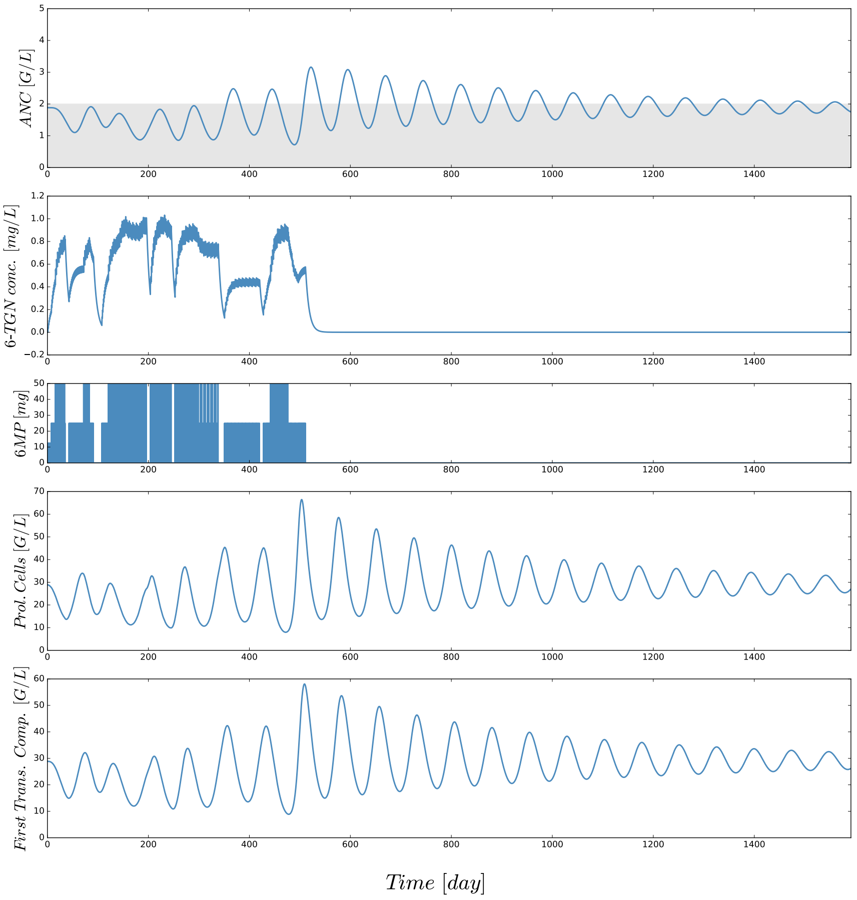

Figure 108: As Figure 1, but for another patient out of 116 patients.

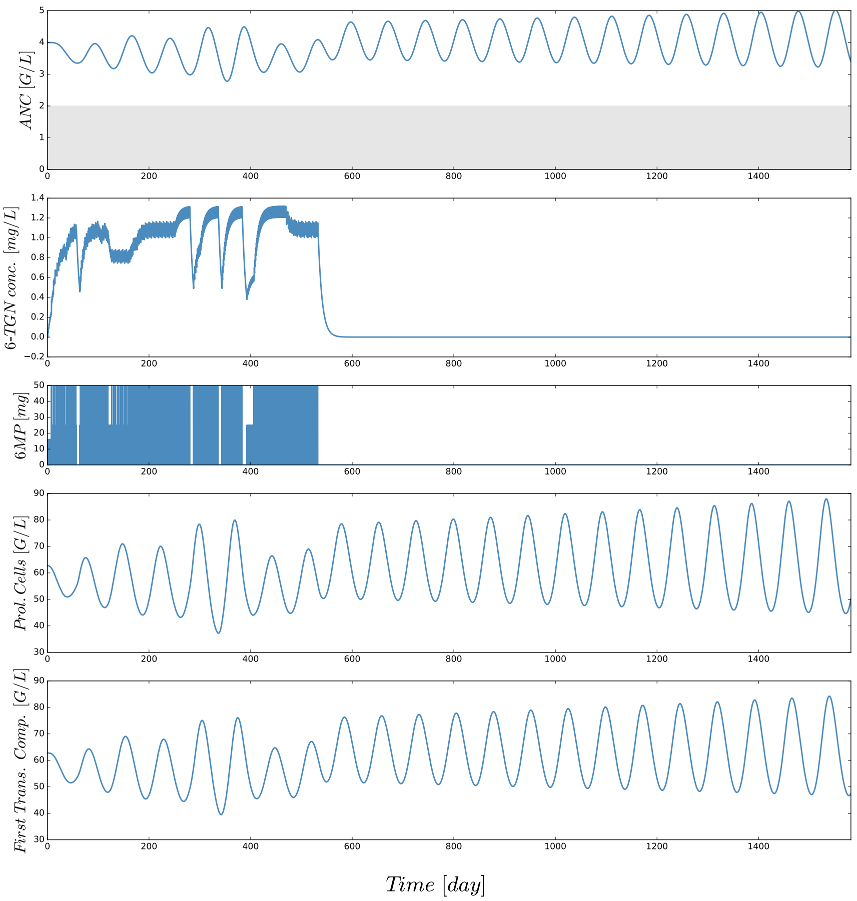

Figure 109: As Figure 1, but for another patient out of 116 patients.

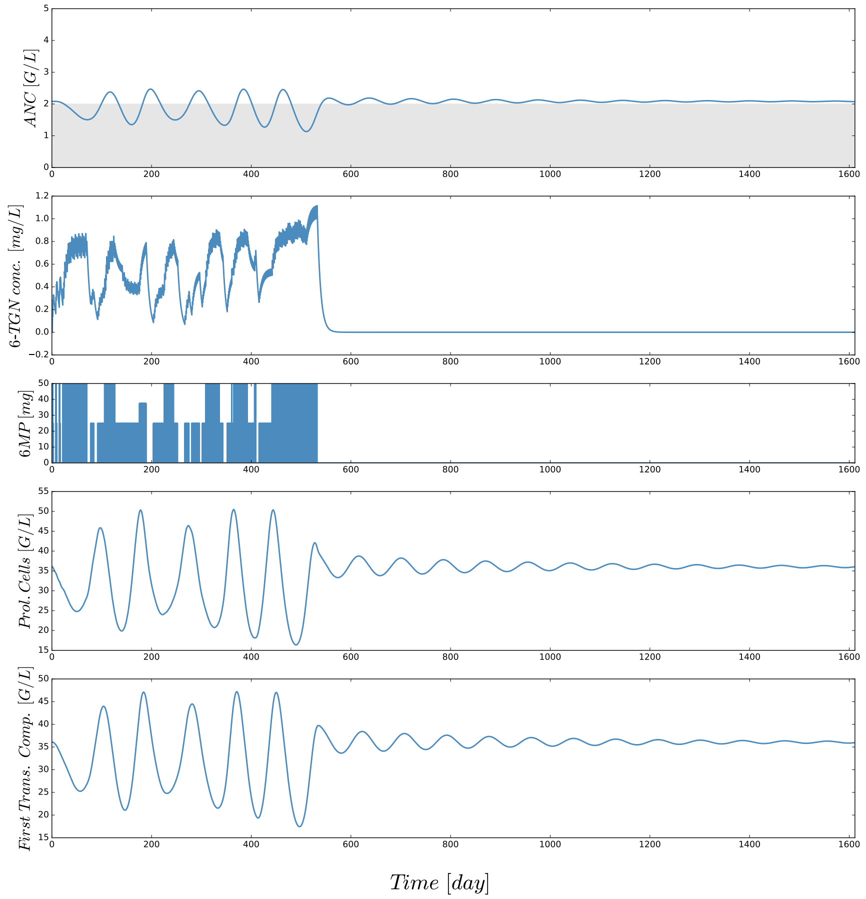

Figure 110: As Figure 1, but for another patient out of 116 patients.

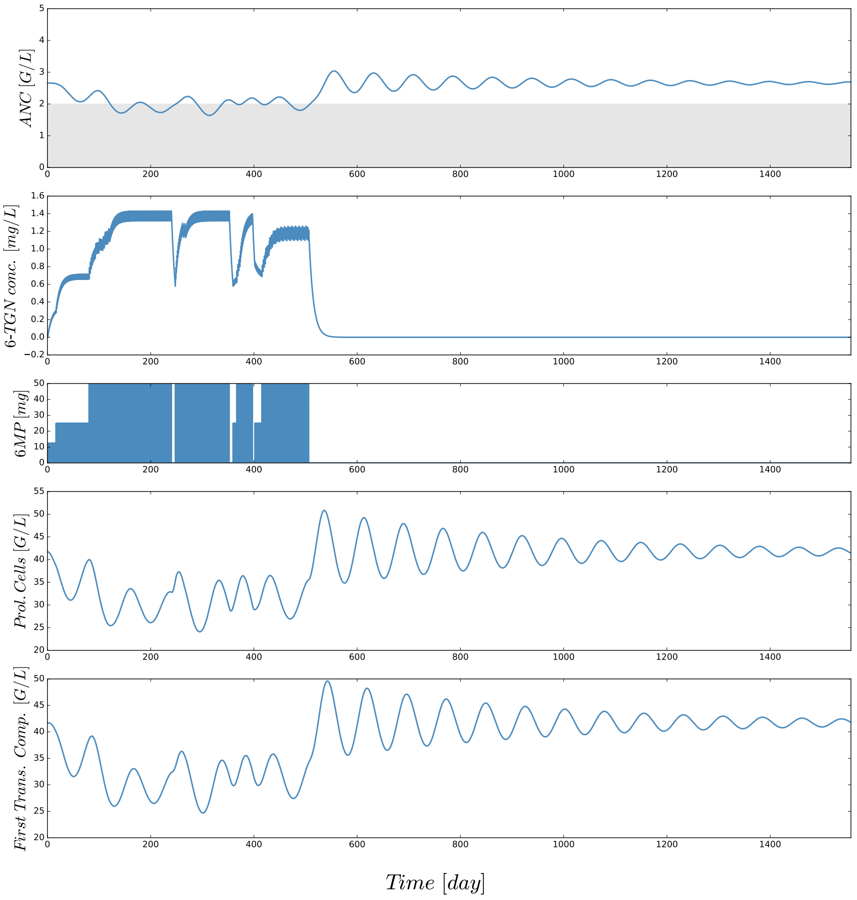

Figure 111: As Figure 1, but for another patient out of 116 patients.

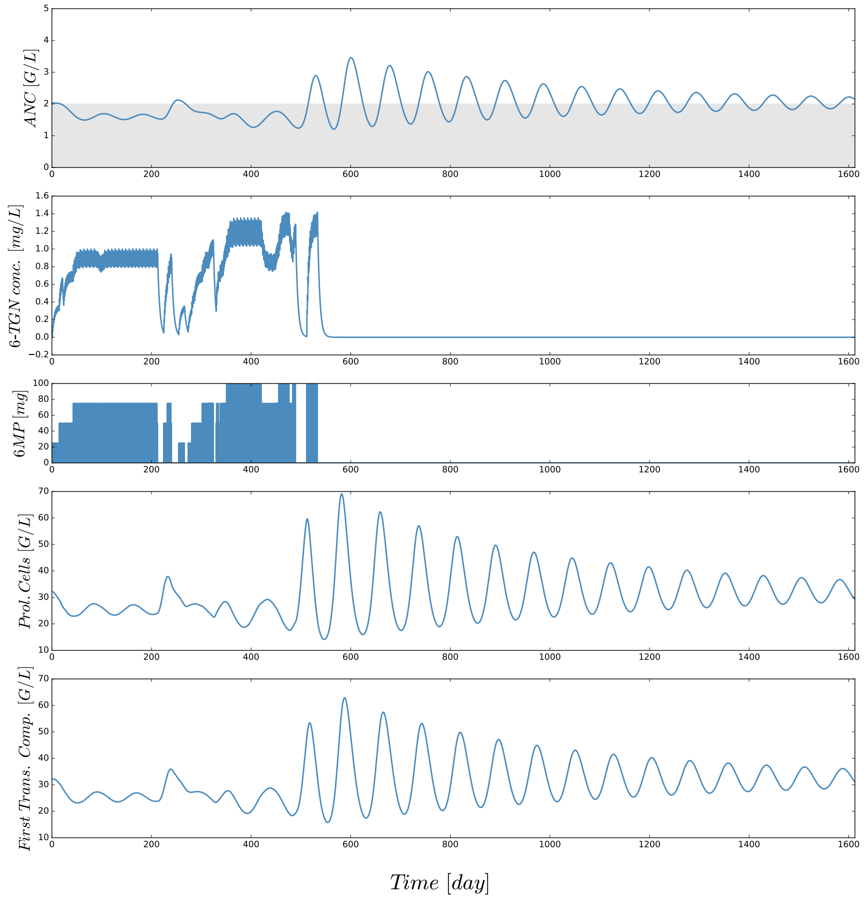

Figure 112: As Figure 1, but for another patient out of 116 patients.

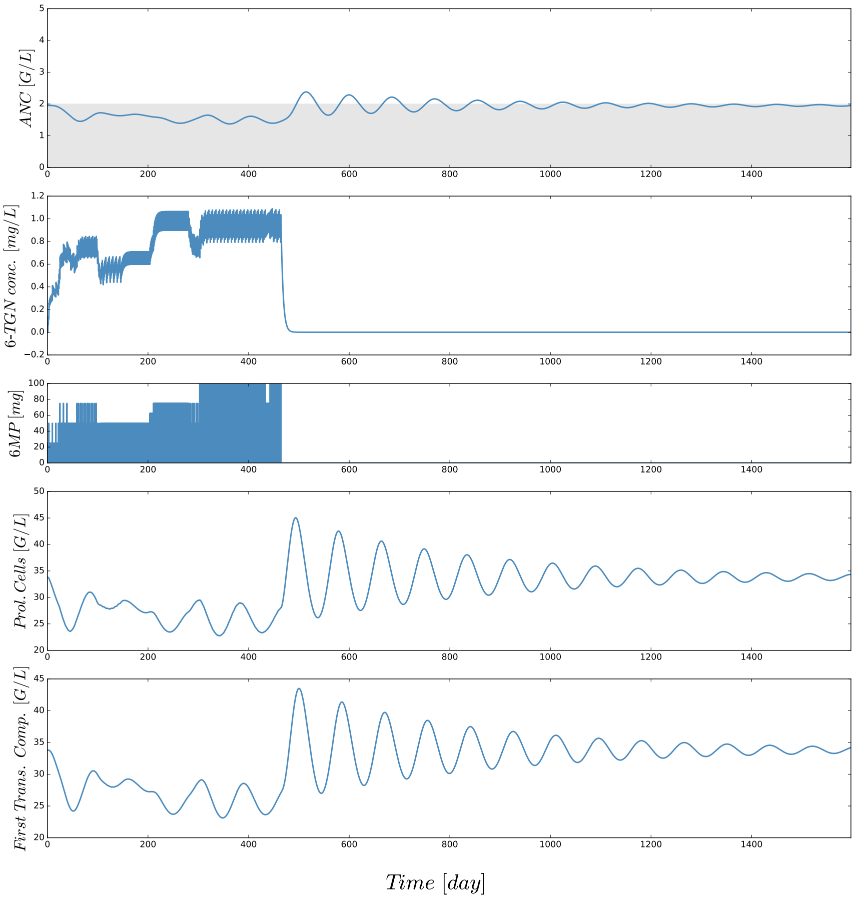

Figure 113: As Figure 1, but for another patient out of 116 patients.

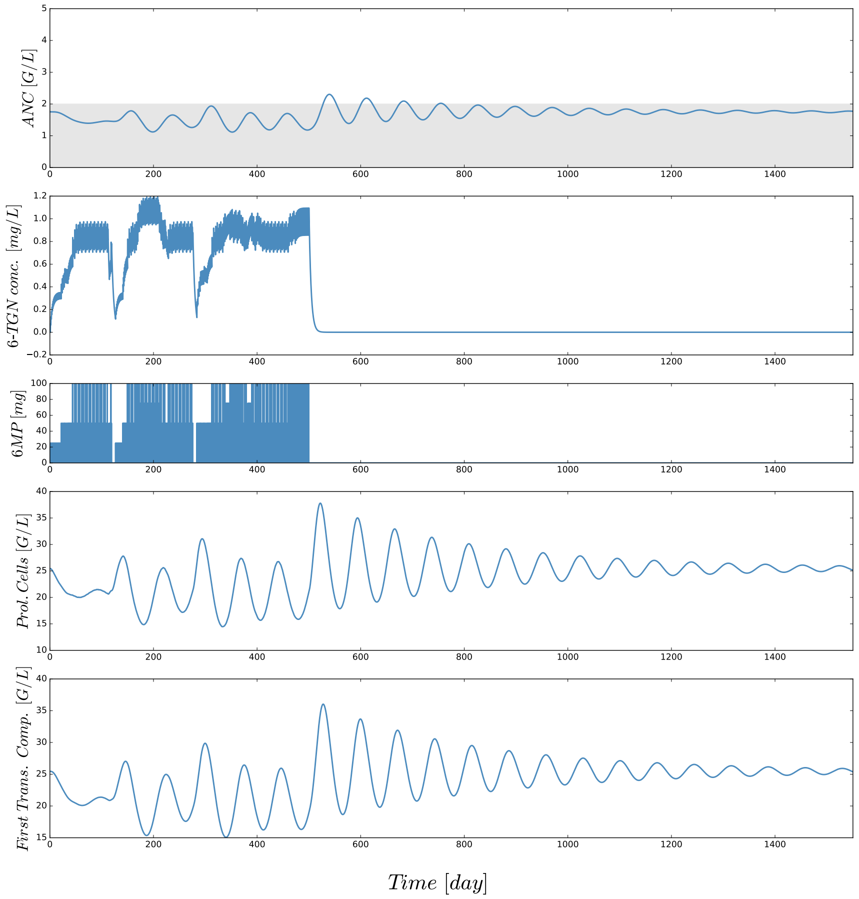

Figure 114: As Figure 1, but for another patient out of 116 patients.

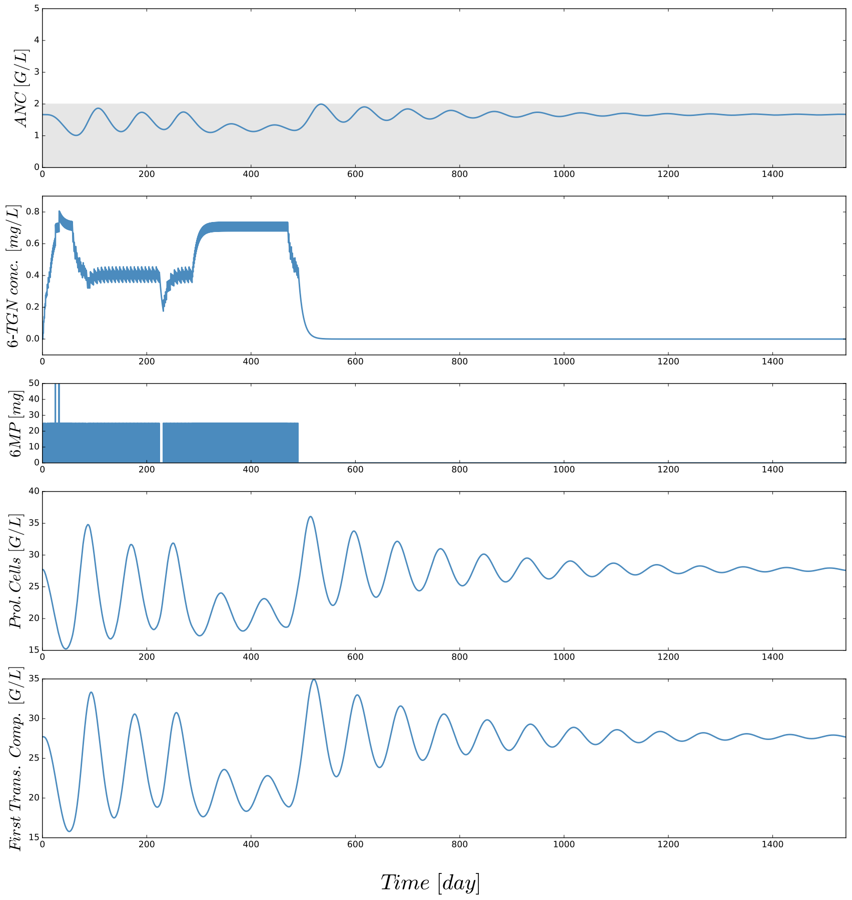

Figure 115: As Figure 1, but for another patient out of 116 patients.

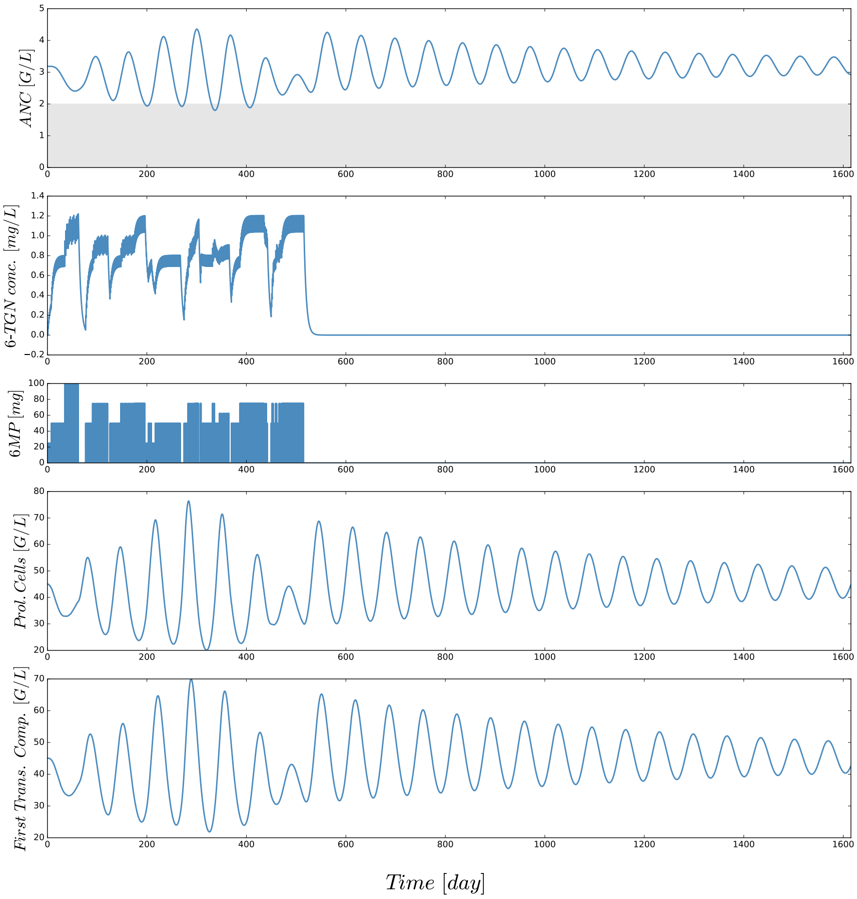

Figure 116: As Figure 1, but for another patient out of 116 patients.
